# Supplementary material for: Catalyst-free carbosilylation of alkenes using silyl boronates and organic fluorides via selective C-F bond activation
Source: Nat Commun. 2021 Jun 18;12:3749. doi: 10.1038/s41467-021-24031-w (PMC8213744; doi:10.1038/s41467-021-24031-w)
Supplement: Supplementary file 1 — SUPPLEMENTARY INFO [file 41467_2021_24031_MOESM1_ESM.pdf]

Supplementary Information

Catalyst-free Carbosilylation of Alkenes using Silyl Boronates and Organic Fluorides via Selective C-F Bond Activation

Jun Zhou,[1] Bingyao Jiang,[2] Yamato Fujihira,[2] Zhengyu Zhao,[1] Takanori Imai,[2] and Norio Shibata*[1,2,3]

[1] *Department of Nanopharmaceutical Sciences, Nagoya Institute of Technology, Gokiso, Showa-ku, Nagoya 466-8555, Japan*

[2] *Department of Life Science and Applied Chemistry, Nagoya Institute of Technology, Gokiso, Showa-ku, Nagoya 466-8555, Japan*

[3] *Institute of Advanced Fluorine-Containing Materials, Zhejiang Normal University, 688 Yingbin Avenue, 321004 Jinhua, China*

**Correspondence and requests for materials should be addressed to N.S. (email: nozshiba@nitech.ac.jp)*

Table of Contents

1. General Information and Materials **3**

2. Supplementary Note 1: Details for Condition Optimization **4**

Supplementary Table 1. Screening catalyst for the carbosilylation of styrene and arylfluoride 4

Supplementary Table 2. Screening base for the carbosilylation of styrene and arylfluoride 5

Supplementary Table 3. Screening solvent for the carbosilylation of styrene and arylfluoride 6

Supplementary Table 4. Screening equivalent of reagents for the carbosilylation of styrene and arylfluoride 7

Supplementary Table 5. Variations after the optimal reaction conditions 8

3. Supplementary Note 2: Experimental Procedures for the Synthesis of Starting Materials **9**

3.1 Synthesis of substituted aryl fluorides **1** 9

3.2 Synthesis of substituted alkyl fluorides **2** 16

3.3 Synthesis of alkenes **3** 20

3.4 Synthesis of trialkyl(4,4,5,5-tetramethyl-1,3,2-dioxaborolan-2-yl)silane 24

4. General Procedure for the Carbosilylation Reactions of Alkenes **25**

5. Unsuccessful Substrates for the Carbosilylation of Alkenes with Organic Fluorides **26**

5.1 Organic fluorides 26

5.2 Alkenes 26

6. Competition between Carbosilylation, Silylation and/or Borylation of Halogen-containing Compounds **27**

6.1 Comparisons of aryl halides with styrene for the carbosilylation 27

6.2 Competitive reaction between aryl halides with styrene for the carbosilylation 27

6.3 Comparisons of alkyl halides with styrene for the carbosilylation 28

6.4 Competitive reaction between alkyl fluoride and alkyl chloride with styrene for the carbosilylation 28

7. Preliminary Mechanistic Investigations **31**

7.1 Reaction with radical scavenger 31

7.2 Radical clock experiments 31

7.3 The reaction process 33

7.4 The NMR spectroscopic studies 34

8. Characterization Data of Products **37**

9. NMR Spectra (^{1}H NMR, ^{13}C NMR and ^{19}F NMR) **56**

10. Supplementary References **170**

1. General Information and Materials

All reactions were performed in oven-dried glassware under a positive pressure of nitrogen or argon. Solvents were transferred via syringe and were introduced into the reaction vessels through a rubber septum. All solvents were dried by standard method. All the reactions were monitored by thin-layer chromatography (TLC) carried out on 0.25 mm Merck silica gel (60-F254). The TLC plates were visualized with UV light. All the reaction products were purified by column chromatography and was carried out on a column packed with silica gel 60N spherical neutral size 50-63 mm. The ^{1}H NMR (300 MHz and 500 MHz) and ^{19}F NMR (282 MHz) spectra as for solution in $CDCl_3$ or Acetone-d^6 were recorded on a Varian Mercury 300 and BRUKER 500 Ultra Shield TR. ^{13}C NMR (126 MHz and 75 MHz) spectra for solution in $CDCl_3$ was recorded on a BRUKER 500 Ultra Shield TR and a Varian Mercury 300. The chemical shifts (δ) are expressed in ppm downfield from internal TMS (δ = 0.00) and coupling constants (*J*) are reported in hertz (Hz). The hexafluorobenzene (C_6F_6) [δ = -162.2 ($CDCl_3$)] was used as internal standard for ^{19}F NMR. The following abbreviations were used to explain the multiplicities: s = singlet, d = doublet, t = triplet, q = quartet, m = multiplet, br = broad. Mass spectra were recorded on a SHIMADZU GCMS-QP5050A (EI-MS) and SHIMADZU LCMS-2020 (ESI-MS). High resolution mass spectrometry (HRMS) was carried out on an electron impact ionization mass spectrometer with a micro-TOF analyzer and recorded on a Waters, GCT Premier (EI-MS) with a TOF analyzer. Infrared spectra were recorded on a JASCO FT/IR-4100 spectrometer. Melting points were recorded on a BUCHI M-565.

Commercially available chemicals were obtained from Aldrich Chemical Co., Alfa Aesar, TCI and used as received unless otherwise noted. Solvents such as acetonitrile, ethyl acetate, Dioxane, DMF, DCM and THF were dried and distilled before use.

2. Supplementary Note 1: Details for Condition Optimization

Supplementary Table 1. Screening catalyst for the carbosilylation of styrene and arylfluoride[a]

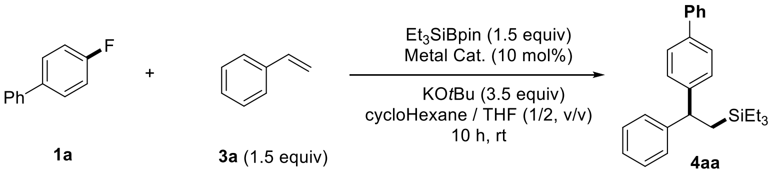

1a **3a** (1.5 equiv) **4aa**

Entry	Metal Cat.	Conversion[b]	4aa (%)[b]
**1**	**$Ni(COD)_2$**	**92**	**38**
**2**	$NiBr_2$·DME	68	36
**3**	$NiBr_2$·diglyme	81	31
**4**	--	59	30
**5**	$CuCl_2$	62	32
**6**	CuI	41	15
**7**	$NiCl_2(PPh_3)_2$	72	27
**8**	$PdCl_2$·dppf	47	23

[a] Reactions were attempted with **1a** (0.1 mmol), **3a** (0.15 mmol), $Et_3SiBpin$ (0.15 mmol), Nickel catalyst (10 mol%), KO*t*Bu (3.5 equiv) in binary solvent of cyclohexane/THF (0.75 mL, 1/2, v/v) at room temperature for 10 hours.

[b] Yields were determined by ^1H NMR and ^{19}F NMR analysis of the crude reaction mixture using 3-fluoropyridine as an internal standard.

Supplementary Table 2. Screening base for the carbosilylation of styrene and arylfluoride[a]

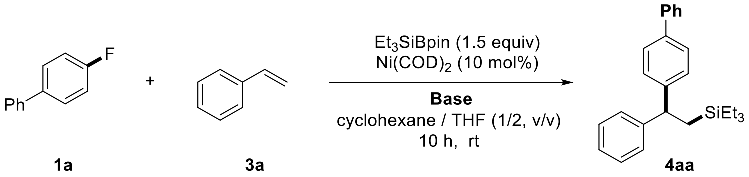

Entry	Base (equiv)	4aa (%)[b]
**1**	KO*t*Bu (3.5)	38
**2**	NaO*t*Bu (3.5)	14
**3**	KHMDS (3.5)	trace
**4**	LiHMDS (3.5)	N.R.
**5**	KOMe (3.5)	trace
**6**	--	N.R.
**7**	KO*t*Bu (2.5)	32
**8**	KO*t*Bu (1.5)	27
**9**	KO*t*Bu (4.0)	52
**10**	KO*t*Bu (5.0)	41

[a] Reactions were carried with **1a** (0.1 mmol), **3a** (0.15 mmol), $Et_3SiBpin$ (0.15 mmol), $Ni(COD)_2$ (10 mol%) and Base in binary solvent of cyclohexane/THF (0.75mL, 1/2, v/v) at room temperature for 10 hours.

[b] Yields were determined by ^{1}H NMR and ^{19}F NMR analysis of the crude reaction mixture using 3-fluoropyridine as an internal standard.

Supplementary Table 3. Screening solvent for the carbosilylation of styrene and arylfluoride[a]

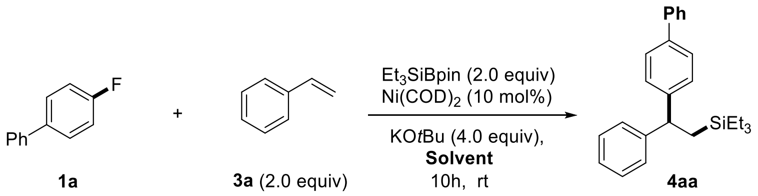

Entry	Solvent	4aa (%)[b]
**1**	cyclohexane/THF(1/2, v/v)	81
**2**	cyclohexane/THF(2/1, v/v)	87
**3**	cyclohexane/THF(1/4, v/v)	84
**4**[c]	cyclohexane/THF(4/1, v/v)	91 (87)
**5**	**cyclohexane/THF(8/1**, v/v)	**98 (95)**
**6**	cyclohexane	71
**7**	THF	79
**8**	Toluene	75
**9**	CH_3CN	trace
**10**	Dioxane	70
**11**	DME	38

[a] Reactions were carried with **1a** (0.1 mmol), **3a** (0.2 mmol), $Et_3SiBpin$ (0.2 mmol), $Ni(COD)_2$ (10 mol%) and KO*t*Bu (4.0 equiv) in corresponding solvent (0.75 mL) at room temperature for 10 hours.

[b] Yields were determined by ^1H NMR and ^{19}F NMR analysis of the crude reaction mixture using 3-fluoropyridine as an internal standard.

[c] The isolated yield was shown in the parenthesis.

Supplementary Table 4. Screening equivalent of reagents for the carbosilylation of styrene and arylfluoride[a]

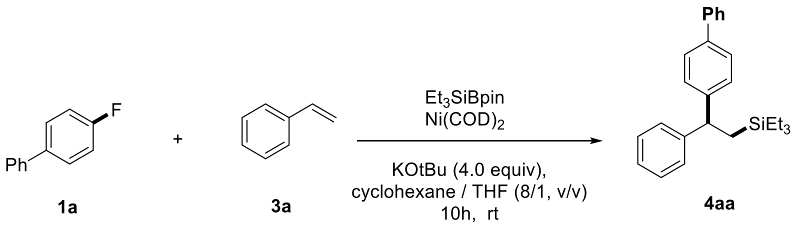

Entry	$Ni(COD)_2$	3a	$Et_3SiBpin$	t (h)	4aa (%)[b]
**1**	10 mol%	2.0	2.0	10	98
**2**	10 mol%	1.8	2.0	10	78
**3**	10 mol%	1.5	2.0	10	66
**4**	10 mol%	1.2	2.0	10	60
**5**	10 mol%	5.0	2.0	10	45
**6**	10 mol%	10.0	2.0	10	9
**7**	10 mol%	2.0	1.5	10	65
**8**	5 mol%	2.0	2.0	10	95
**9**	1 mol%	2.0	2.0	10	96
**10**[c]	1 mol%	2.0	2.0	2.5	99 (94)
**11**[c]	--	2.0	2.0	2.5	94 (91)

[a] Reactions were carried with **1a** (0.1 mmol), **3a**, $Et_3SiBpin$, $Ni(COD)_2$ and KO*t*Bu (4.0 equiv) in a binary solvent of cyclohexane/THF (0.75 mL, 8/1, v/v) at room temperature.

[b] Yields were determined by ^1H NMR and ^{19}F NMR analysis of the crude reaction mixture using 3-fluoropyridine as an internal standard.

[c] The isolated yield was shown in the parenthesis.

Supplementary Table 5. Variations after the optimal reaction conditions[a]

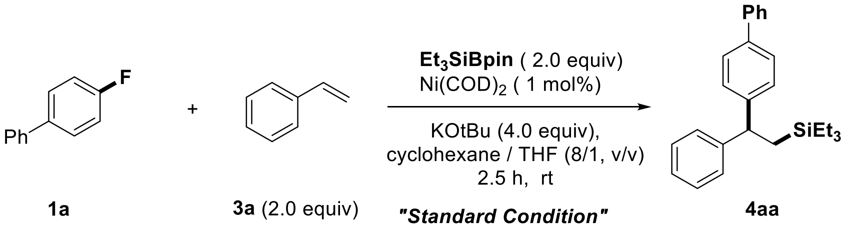

Entry	Variations from "***Standard Condition***"	**4aa** (%)[b]
**1**[c]	none	99(94)
**2**[c]	Without $Ni(COD)_2$	94(91)
**3**	Without KO*t*Bu	0
**4**	NaO*t*Bu instead of KO*t*Bu	15
**5**	LiO*t*Bu, KOMe or KHMDS instead of KO*t*Bu	0
**6**	3.5 equiv of KO*t*Bu	90
**7**	Without $Et_3SiBpin$	0
**8**	1.5 equiv of $Et_3SiBpin$ instead of 2.0 equiv	65
**9**	cyclohexane instead of cyclohexane/THF (8/1, v/v)	76
**10**	THF instead of cyclohexane/THF (8/1, v/v)	81
**11**	1,4-Dioxane instead of cyclohexane/THF (8/1, v/v)	71
**12**	Toluene instead of cyclohexane/THF (8/1, v/v)	73
**13**	DME instead of cyclohexane/THF (8/1, v/v)	38
**14**	CH_3CN, DMF or DCE instead of cyclohexane/THF (8/1, v/v)	0
**15**[c]	0.2 mmol **1a** was used	98(96)

[a] Standard Reaction conditions were carried with **1a** (0.1 mmol), **3a** (0.2 mmol), $Et_3SiBpin$ (0.2 mmol), $Ni(COD)_2$ (1 mol%) and KO*t*Bu (4.0 equiv.) in a binary solvent of cyclohexane/THF (0.75 mL, 8/1, v/v) at room temperature.

[b] Yields were determined by ^{1}H NMR and ^{19}F NMR analysis of the crude reaction mixture using 3-fluoropyridine as an internal standard.

[c] The isolated yield was shown in the parenthesis.

3. Supplementary Note 2: Experimental Procedures for the Synthesis of Starting Materials

3.1 Synthesis of substituted aryl fluorides 1

Fluoroarenes **1a, 1c, 1d**, **1f, 1g** and **1z** were purchased from TCI or Sigma Aldrich. **1b**, **1e**, **1h**, **1j**, **1o**, **1s**, **1t, 1y** and **6a** were used prepared according to known methods. A typical experimental procedure for the preparation of **1i**, **1k**, **1l**, **1m**, **1n**, **1p**, **1q**, **1r, 1u, 1v, 1w, 1x** and **6b, 6c, 6d, 6e** were described below.

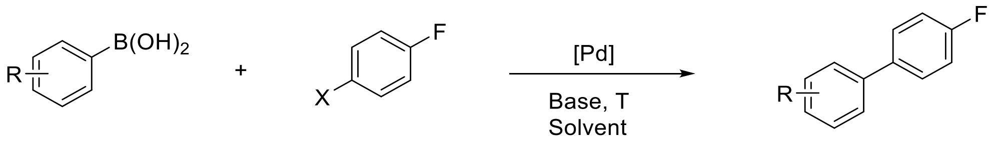

4-(Benzyloxy)-4'-fluoro-1,1'-biphenyl (1i)[1]

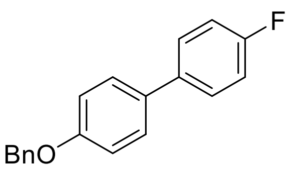

A dried flask was charged with 1-Fluoro-4-iodobenzene (0.55 mL, 5.0 mmol), 4-Benzyloxyphenylboronic acid (1.37 g, 6.0 mmol), Pd(dppf)Cl_2 (81.7 mg, 0.1 mmol) and *N,N*-Diisopropylethylamine (2.55 mL, 15 mmol) in ^{i}PrOH/H_2O (30 mL, v/v = 2/1). The mixture was stirred at 100 °C and the reaction progress was monitored by TLC. After stirring for 20 h, the mixture was cooled to room temperature. To this mixture was added water 5 mL, and then extracted with EtOAc (15 mL × 3). The combined organic phases were washed with brine (10mL), and dried over Na_2SO_4. After filtration, the filtrate was concentrated under reduced pressure. The residue was purified by column chromatography on silica gel (Hexane) to give title compound as white solid (0.612 g, 44%). **^{1}H NMR** (300 MHz, $CDCl_3$) δ 7.59 – 7.30 (m, 8H), 7.16 – 6.98 (m, 4H), 5.11 (s, 2H). **^{19}F NMR** (282 MHz, $CDCl_3$) δ -117.22(s, 1F). **MS(EI)**: *m/z* 278 $[M]^+$. The chemical shifts were consistent with those reported in the literature.

4-Fluoro-3'-(trifluoromethyl)-1,1'-biphenyl (1k)[2]

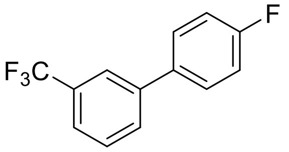

A dried flask was charged with 1-Fluoro-4-bromobenzene (0.77 mL, 7.0 mmol), 3-(Trifluoromethyl)phenylboronic acid (1.60 g, 8.4 mmol), $Pd(OAc)_2$ (41 mg, 0.182 mmol), Ph_3P (184 mg, 0.7 mmol) and KOH (786 mg, 14 mmol) in THF/H_2O (36 mL, v/v = 5/1). The mixture was stirred at 60 °C and the reaction progress was monitored by TLC. After stirring for 16 h, the mixture was cooled to room temperature. To this mixture was added water 5 mL, and then extracted with EtOAc (15 mL × 3). The combined organic phases were washed with brine (10mL), and dried over Na_2SO_4. After filtration, the filtrate was concentrated under reduced pressure. The residue was purified by column chromatography on silica gel (Hexane) to give title compound as white solid (1.26 g, 75 %). **^{1}H NMR** (300 MHz, $CDCl_3$) δ 7.88 – 7.68 (m, 2H), 7.57

(dt, J = 12.1, 4.9 Hz, 4H), 7.16 (t, J = 8.6 Hz, 2H). **^{19}F NMR** (282 MHz, $CDCl_3$) δ -63.10(s, 3F), -114.90 (tt, J = 9.1, 5.3 Hz, 1F). **MS(EI)**: *m/z* 240 $[M]^+$. The chemical shifts were consistent with those reported in the literature.

4-Fluoro-4'-(trifluoromethyl)-1,1'-biphenyl (1l)[3]

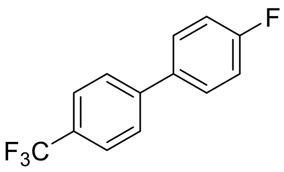

A dried flask was charged with 1-Fluoro-4-bromobenzene (0.77 mL, 7.0 mmol), 4-(Trifluoromethyl)phenylboronic acid (1.60 g, 8.4 mmol), $Pd(OAc)_2$ (41 mg, 0.182 mmol), Ph_3P (184 mg, 0.7 mmol) and KOH (786 mg, 14 mmol) in THF/H_2O (36 mL, v/v = 5/1). The mixture was stirred at 60 ºC and the reaction progress was monitored by TLC. After stirring for 16 h, the mixture was cooled to room temperature. To this mixture was added water 5 mL, and then extracted with EtOAc (15 mL × 3). The combined organic phases were washed with brine (10 mL), and dried over Na_2SO_4. After filtration, the filtrate was concentrated under reduced pressure. The residue was purified by column chromatography on silica gel (Hexane) to give title compound as white solid (1.60 g, 95 %). **^{1}H NMR** (300 MHz, $CDCl_3$) δ 7.76 – 7.60 (m, 4H), 7.62 – 7.51 (m, 2H), 7.17 (t, J = 8.7 Hz, 2H). **^{19}F NMR** (282 MHz, $CDCl_3$) δ -62.92(s, 3F), -114.66 (dt, J = 8.8, 3.5 Hz, 1F). **MS(EI)**: *m/z* 240 $[M]^+$. The chemical shifts were consistent with those reported in the literature.

4-Chloro-4'-fluoro-1,1'-biphenyl (1m)[4]

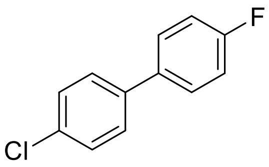

A dried flask was charged with 1-Fluoro-4-iodobenzene (0.81 mL, 7.0 mmol), 4-Chlorophenylboronic acid (1.20 g, 7.7 mmol), $Pd(OAc)_2$ (16 mg, 0.07 mmol), and K_3PO_4 (2.97g, 14 mmol) in THF/H_2O (30 mL, v/v = 2/1). The mixture was stirred at room temperature for 2 h, and the reaction progress was monitored by TLC. To this mixture was added water 5 mL, and then extracted with EtOAc (15 mL × 3). The combined organic phases were washed with brine (10 mL), and dried over Na_2SO_4. After filtration, the filtrate was concentrated under reduced pressure. The residue was purified by column chromatography on silica gel (Hexane) to give title compound clear oil (0.72 g, 50%). **^{1}H NMR** (300 MHz, $CDCl_3$) δ 7.57 – 7.42 (m, 4H), 7.45 – 7.35 (m, 2H), 7.13 (t, J = 8.6 Hz, 2H). **^{19}F NMR** (282 MHz, $CDCl_3$) δ -115.70 (d, J = 9.9 Hz, 1F). **MS(EI)**: *m/z* 206 $[M]^+$. The chemical shifts were consistent with those reported in the literature.

4-(4-Fluorophenyl)pyridine (1p)[5]

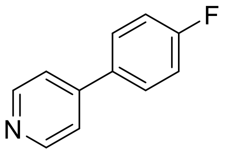

A dried flask was charged with 1-Fluoro-4-bromobenzene (0.55 mL, 5.0 mmol), 4-Pyridylboronic acid (0.74 g, 6 mmol), $Pd(PPh_3)Cl_2$ (351 mg, 0.5 mmol), and K_2CO_3 (2.76g, 20 mmol) in DMF/H_2O (25 mL, v/v = 4/1). The mixture was stirred at 100 ºC overnight, and the reaction progress was monitored by TLC. To this mixture was added water 5 mL, and then extracted with EtOAc (15 mL × 3). The combined organic phases were washed with brine (10 mL), and dried over Na_2SO_4. After filtration, the filtrate was concentrated under reduced pressure. The residue was purified by column chromatography on silica gel (Hexane/EtOAc 5/1) to give title compound as a white solid (0.856 g, 99%). **^{1}H NMR** (300 MHz, $CDCl_3$) δ 8.64 (d, J = 4.4 Hz, 2H), 7.74 – 7.53 (m, 2H), 7.50 – 7.39 (m, 2H), 7.23 – 7.05 (m, 2H). **^{19}F NMR** (282 MHz, $CDCl_3$) δ -113.08 (q, J = 7.2,

6.6 Hz, 1F). **MS(EI)**: *m/z* 173 $[M]^+$. The chemical shifts were consistent with those reported in the literature.

5-Fluoro-2-phenylpyridine (1q)[6]

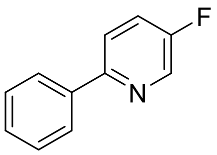

A dried flask was charged with 2-bromo-5-fluoropyridine (0.88 g, 5.0 mmol), Phenylboronic acid (0.92 g, 7.5 mmol), $Pd(OAc)_2$ (56 mg, 0.25 mmol), and K_2CO_3 (1.38 g, 10 mmol) in EtOH/H_2O (20 mL, v/v = 3/1). The mixture was stirred at 80 °C overnight, and the reaction progress was monitored by TLC. To this mixture was added water 5 mL, and then extracted with EtOAc (15 mL × 3). The combined organic phases were washed with brine (10 mL), and dried over Na_2SO_4. After filtration, the filtrate was concentrated under reduced pressure. The residue was purified by column chromatography on silica gel (Hexane/EtOAc 10/1) to give title compound as a white solid (0.80 g, 93%). **^{1}H NMR** (300 MHz, $CDCl_3$) δ 8.55 (d, *J* = 3.0 Hz, 1H), 7.94 (d, *J* = 6.7 Hz, 2H), 7.72 (dd, *J* = 8.7, 4.3 Hz, 1H), 7.46 (qd, *J* = 9.8, 8.6, 5.0 Hz, 4H). **^{19}F NMR** (282 MHz, $CDCl_3$) δ -130.32 (dd, *J* = 8.3, 4.4 Hz, 1F). **MS(EI)**: *m/z* 173 $[M]^+$. The chemical shifts were consistent with those reported in the literature.

2-Fluoro-5-phenylpyridine (1r)[7]

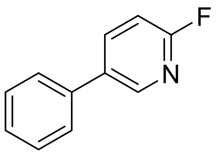

A dried flask was charged with 2-Fluoro-5-bromopyridine (0.5 mL, 5.0 mmol), Phenylboronic acid (0.61 g, 5 mmol), $Pd(PPh_3)_4$ (289 mg, 0.25 mmol), and Na_2CO_3 (1.06 g, 10 mmol) in MeCN/H_2O (25 mL, v/v = 4/1). The mixture was stirred at 90 °C for 3h, and the reaction progress was monitored by TLC. To this mixture was added water 5 mL, and then extracted with EtOAc (15 mL × 3). The combined organic phases were washed with brine (10 mL), and dried over Na_2SO_4. After filtration, the filtrate was concentrated under reduced pressure. The residue was purified by column chromatography on silica gel (Hexane/EtOAc 100/1) to give title compound as a colorless oil (0.75 g, 88%). **^{1}H NMR** (300 MHz, $CDCl_3$) δ 8.42 (d, *J* = 2.7 Hz, 1H), 7.97 (td, *J* = 8.0, 2.7 Hz, 1H), 7.60 – 7.36 (m, 5H), 7.01 (dd, *J* = 8.4, 3.2 Hz, 1H). **^{19}F NMR** (282 MHz, $CDCl_3$) δ -71.11 (d, *J* = 7.9 Hz, 1F). **MS(EI)**: *m/z* 173 $[M]^+$. The chemical shifts were consistent with those reported in the literature.

3-(4-Fluorophenyl)benzofuran (1u)[8]

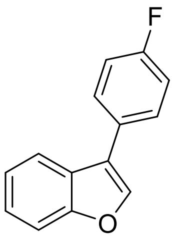

A dried flask was charged with 1-Fluoro-4-bromobenzene (0.2 mL, 2.0 mmol), Benzofuran-3-boronic acid (0.36 g, 2.2 mmol), $Pd(PPh_3)_4$ (116 mg, 0.1 mmol), and saturated $NaHCO_3$ (3 mL) in 1,4-Dioxane (15 mL). The mixture was stirred at 110 °C for 2h, and the reaction progress was monitored by TLC. To this mixture was added water 5 mL, and then extracted with EtOAc (10 mL × 3). The combined organic phases were washed with brine (10 mL), and dried over Na_2SO_4. After filtration, the filtrate was concentrated under reduced pressure. The residue was purified by column chromatography on silica gel (Hexane) to give title compound as a white solid (0.33 g, 77%). **^{1}H NMR** (300 MHz, $CDCl_3$) δ 7.89 – 7.78 (m, 1H), 7.78 (d, *J* = 3.3 Hz, 1H), 7.69 – 7.54 (m, 3H), 7.49 – 7.31 (m, 2H), 7.22 (td, *J* = 8.7, 3.0 Hz, 2H). **^{19}F NMR** (282 MHz, $CDCl_3$) δ -114.80 – -115.15 (m, 1F). **MS(EI)**: *m/z* 278 $[M]^+$. The chemical shifts were consistent with those

reported in the literature.

6-Fluoro-1-methyl-*1H*-indole (1n)[9]

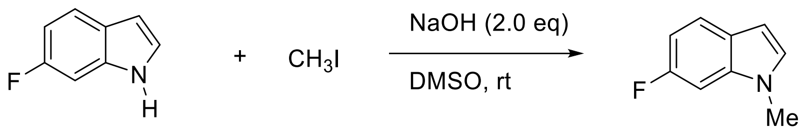

To a solution of 6-Fluoroindole (0.36 g, 2.7 mmol) in DMSO (10 mL) was added NaOH (0.22 g, 5.3 mmol, 60% in mineral oil) at room temperature. After stirring for 30 min, then CH_3I (0.76 g, 5.3 mmol) was added and the reaction was then stirred at room temperature overnight. After check TLC, the reaction was quenched with saturated NH_4Cl (5 mL) and extracted with Et_2O (15mL × 3). The combined organic extract was washed with brine (15 mL) and dried over Na_2SO_4. After filtration, the filtrate was concentrated under reduced pressure. The crude reaction mixture was purified by column chromatography on silica gel (Hexane/DCM 50:1) to give title compound as clear oil (0.33 g, 81%). **^{1}H NMR** (300 MHz, $CDCl_3$) δ 7.69 – 7.48 (m, 1H), 7.11 – 6.97 (m, 2H), 6.93 (ddt, *J* = 9.6, 8.6, 2.3 Hz, 1H), 6.56 – 6.46 (m, 1H), 3.75 (s, 3H). **^{19}F NMR** (282 MHz, $CDCl_3$) δ -121.07 – -121.37 (m, 1F). **MS(EI)**: *m/z* 149 $[M]^+$. The chemical shifts were consistent with those reported in the literature.

1-Fluoro-3-(3-methoxypropyl)benzene (1v)

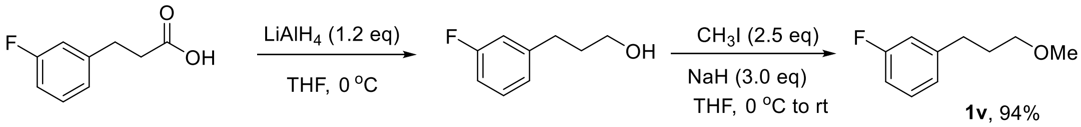

A flame dried flask was charged with $LiAlH_4$ (319 mg, 8.4 mmol) and 10 mL anhydrous THF, then cooled to 0 °C. A solution of 3-(3-Fluorophenyl)propanoic acid (1.18 g, 7.0 mmol)in 5 mL anhydrous THF was then added under 0 °C. The mixture was stirred overnight, then mixture was quenched with water (0.32 mL), and then 10% NaOH (0.32 mL) was added slowly, water (0.96 mL), then filtered with Celite to get the filtration, followed extracted with EtOAc (15 mL × 3). The combined organic phases were washed with brine (10 mL), and dried over Na_2SO_4. Concentrated under reduced pressure to give crude product. The crude product was dissolved in 30 mL dry THF, stirred at 0 °C, then treated with NaH (0.7 g, 17.5 mmol, 60% in mineral oil). After stirring 30 min, CH_3I (1.10 mL, 17.5 mmol) was then added slowly. The mixture was stirred at room temperature for 2h, and the reaction progress was monitored by TLC. To this mixture was added water 5 mL, and then extracted with EtOAc (15 mL × 3). The combined organic phases were washed with brine (10 mL), and dried over Na_2SO_4. After filtration, the filtrate was concentrated under reduced pressure. The residue was purified by column chromatography on silica gel (Hexane) to give title compound as clear oil (1.11 g, 94%). **^{1}H NMR** (300 MHz, $CDCl_3$) δ 7.24 (q, *J* = 7.3, 6.9 Hz, 1H), 6.97 (d, *J* = 7.6 Hz, 1H), 6.88 (t, *J* = 8.5 Hz, 2H), 3.38 (t, *J* = 6.3 Hz, 2H), 3.35 (s, 2H), 2.70 (t, *J* = 7.5 Hz, 2H), 1.97 – 1.81 (m, 2H). **^{13}C NMR** (75 MHz, $CDCl_3$) δ 163.0 (d, *J* = 245.2 Hz), 144.7, 129.8, 124.2, 115.4 (d, *J* = 20.6 Hz), 112.7 (d, *J* = 21.0 Hz), 71.7, 58.7, 32.2, 31.1. **^{19}F NMR** (282 MHz, $CDCl_3$) δ -114.44 (td, *J* = 9.5, 6.2 Hz, 1F). **IR (KBr):** 3040, 2925, 2873, 2825, 1616, 1590, 1487, 1450, 1386, 1254, 1119, 944, 882, 783, 690 cm^{-1}. **HRMS (EI)** $[C_{10}H_{13}OF]$ $[M]^+$ calculated:

168.0951, found: 168.0951.

6-((4-Fluorobenzyl)oxy)-2,5,7,8-tetramethyl-2-(4,8,12-trimethyltridecyl)chromane (1w)

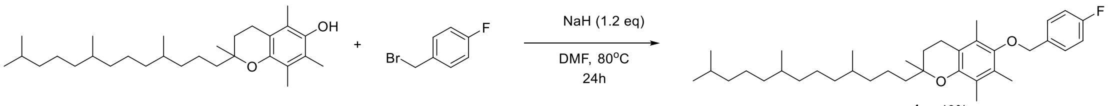

1w, 49%

To a solution of (±)-α-Tocopherol (1.36 g, 3 mmol) in DMF (10 mL) was added NaH (0.144 g, 3.6 mmol, 60% in mineral oil) at room temperature. After stirring for 30 min, then 4-Fluorobenzyl bromide (0.41 mL, 3.3 mmol) was added and the reaction was then stirred at 80 °C for 24h. After check TLC, the reaction was quenched with saturated NH_4Cl (5 mL) and extracted with EtOAc (15 mL × 3). The combined organic extract was washed with brine (15 mL) and dried over Na_2SO_4. After filtration, the filtrate was concentrated under reduced pressure. The crude reaction mixture was purified by column chromatography on silica gel (Hexane/ EtOAc 100:1) to give title compound as clear oil (0.79 g, 49%). **^{1}H NMR** (300 MHz, $CDCl_3$) δ 7.49 (dd, *J* = 8.2, 5.6 Hz, 2H), 7.11 (t, *J* = 8.6 Hz, 2H), 4.69 (s, 2H), 2.62 (t, *J* = 6.4 Hz, 2H), 2.24 (s, 3H), 2.19 (s, 3H), 2.14 (s, 3H), 1.96 – 1.73 (m, 2H), 1.66 – 1.51 (m, 3H), 1.51 – 1.38 (m, 4H), 1.35 – 1.22 (m, 12H), 1.21 – 1.07 (m, 7H), 0.96 – 0.83 (m, 12H). **^{13}C NMR** (75 MHz, $CDCl_3$) δ 162.5 (d, *J* = 245.9 Hz), 148.1, 133.9, 129.6, 128.0, 126.0, 123.1, 117.7, 115.4, 74.9, 74.1. 40.2, 39.5, 37.7, 37.6, 37.6, 37.4, 32.9, 32.8, 31.4, 28.1, 25.0, 24.6, 24.0, 22.9, 22.8, 21.2, 20.8, 19.8, 13.0, 12.1, 12.0. **^{19}F NMR** (282 MHz, $CDCl_3$) δ -115.06 (t, *J* = 7.3 Hz, 1F). **IR (KBr):** 3036, 2950, 2925, 2867, 1510, 1457, 1374, 1225, 1155, 1087, 1014, 821 cm^{-1}. **HRMS (ESI)** [$C_{36}H_{55}O_2NaF$] $[M+Na]^+$ calculated: 561.4084, found: 561.4092.

1-Fluoro-4-((((*2S*,*5R*)-2-isopropyl-5-methylcyclohexyl)oxy)methyl)benzene (1x)

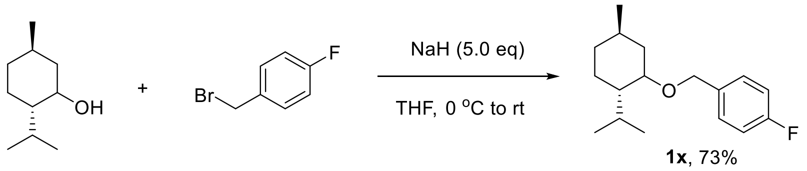

1x, 73%

To a solution of menthol (1.25 g, 8 mmol) in THF (30 mL) was added NaH (1.60 g, 40.0 mmol, 60% in mineral oil) at 0 °C. After stirring for 30 min, then 4-Fluorobenzyl bromide (1.96 mL, 16 mmol) was added at 0 °C and the reaction was then stirred at room temperature overnight. After check TLC, the reaction was quenched with saturated NH_4Cl (5 mL) and extracted with Et_2O (15 mL × 3). The combined organic extract was washed with brine (15 mL) and dried over Na_2SO_4. After filtration, the filtrate was concentrated under reduced pressure. The crude reaction mixture was purified by column chromatography on silica gel (Hexane/DCM 100:1) to give title compound as clear oil (1.54 g, 73%). **^{1}H NMR** (300 MHz, $CDCl_3$) δ 7.31 (dd, *J* = 8.3, 5.5 Hz, 2H), 7.02 (t, *J* = 8.7 Hz, 2H), 4.62 (d, *J* = 11.3 Hz, 1H), 4.35 (d, *J* = 11.3 Hz, 1H), 3.16 (td, *J* = 10.5, 4.1 Hz, 1H), 2.39 – 2.10 (m, 2H), 1.74 – 1.59 (m, 2H), 1.44 – 1.21 (m, 2H), 1.10 – 0.77 (m, 9H), 0.71 (d, *J* = 7.0 Hz, 3H). **^{13}C NMR** (75 MHz, $CDCl_3$) δ 162.3 (d, *J* = 245.2 Hz), 135.0 (d, *J* = 3.0 Hz), 129.6, 115.2 (d, *J* = 21.4 Hz), 78.8, 69.8, 48.4, 40.4, 34.6, 31.6, 25.6, 23.3, 22.5, 21.1, 16.1. **^{19}F NMR** (282 MHz, $CDCl_3$) δ -115.14 – -116.59 (m, 1F). **IR (KBr):** 3040, 2954, 2922, 2868, 1604, 1510, 1455, 1369, 1342, 1223,

1155, 1106, 1085, 1015, 853, 823, 587 cm^{-1}. **HRMS (EI)** [$C_{17}H_{25}OF$] [M]$^+$ calculated: 264.1889, found: 264.1900.

3-Fluoroestrone derivative (1y)

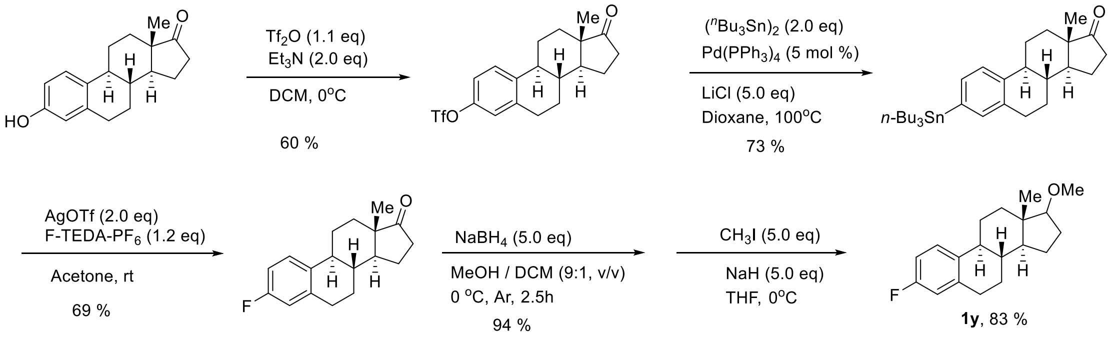

Synthetic intermediates were prepared according to procedures previously described.[10]

The alcohol intermediate (548 mg, 2 mmol) was dissolved in 10 mL dry THF, stirred at room temperature, then treated with NaH (240 mg, 10 mmol, 60% in mineral oil). After stirring 30 min, CH_3I (0.64 mL, 6 mmol) was then added slowly. The mixture was stirred at room temperature for 2h, and the reaction progress was monitored by TLC. To this mixture was added water 5 mL, and then extracted with EtOAc (10 mL × 3). The combined organic phases were washed with brine (10 mL), and dried over Na_2SO_4. After filtration, the filtrate was concentrated under reduced pressure. The residue was purified by column chromatography on silica gel (Hexane) to give title compound as white solid (478 mg, 83%). **^{1}H NMR** (300 MHz, $CDCl_3$) δ 7.23 (t, *J* = 8.1 Hz, 1H), 6.83 (d, *J* = 8.6 Hz, 1H), 6.77 (d, *J* = 9.7 Hz, 1H), 3.38 (d, *J* = 1.4 Hz, 3H), 3.37 – 3.26 (m, 1H), 2.85 (d, *J* = 6.8 Hz, 2H), 2.37 – 2.11 (m, 2H), 2.06 (d, *J* = 11.2 Hz, 2H), 1.94 – 1.81 (m, 1H), 1.78 – 1.62 (m, 1H), 1.59 – 1.15 (m, 7H), 0.79 (s, 3H). **^{19}F NMR** (282 MHz, $CDCl_3$) δ -118.45 – -118.73 (m, 1F). **MS(EI)**: *m/z* 288 [M]$^+$. The chemical shifts were consistent with those reported in the literature.

1-Fluoro-2-(3-phenylbut-3-en-1-yl)benzene (6b)[11-13]

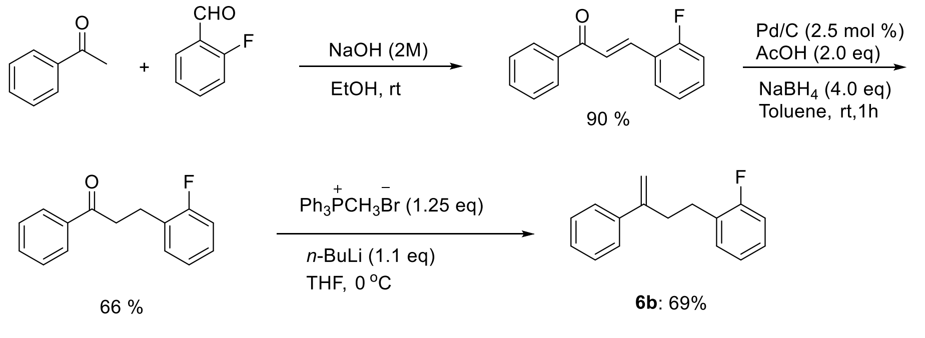

To an anhydrous THF (15 mL) solution of Methyltriphenylphosphonium bromide (1.80 g, 5.0 mmol) a was added *n*-Butyllithium (2.7 mL, 4.4 mmol, 1.64 M in Hexane) at 0°C. After the mixture was stirred at room temperature for 30 min, a THF solution of ketone (0.91 g, 4.0 mmol) was added dropwise at 0°C. After stirring at room temperature for 14 h, the mixture was quenched with water, and the aqueous layer was extracted with EtOAc (10 mL x 3). The combined organic phases were washed with brine, dried over $MgSO_4$, filtered and concentrated in vaccuo. The residue was purified by column chromatography on silica gel (Hexane) to give title compound as colorless oil (0.62 g, 69%). **^{1}H NMR** (300 MHz, $CDCl_3$) δ 7.48 – 7.39 (m, 2H), 7.38 – 7.23 (m, 3H), 7.18 – 7.05 (m, 2H),

7.06 – 6.92 (m, 2H), 5.29 (d, J = 1.4 Hz, 1H), 5.05 (s, 1H), 2.79 (s, 4H). **^{13}C NMR** (75 MHz, $CDCl_3$) δ 161.3 (d, J = 244.8 Hz), 147.7, 140.9, 130.8, 128.8 (d, J = 16.0 Hz), 128.5, 127.7 (d, J = 8.1 Hz), 127.6, 126.2, 124.0, 115.3 (d, J = 22.1 Hz), 113.0, 35.9, 28.4. **^{19}F NMR** (282 MHz, $CDCl_3$) δ -119.14 (q, J = 7.7 Hz, 1F). **IR (KBr):** 3082, 3057, 3029, 2934, 1627, 1584, 1492, 1455, 1229, 1184, 1098, 1028, 897, 755, 704 cm^{-1}. **HRMS (EI)** [$C_{16}H_{15}F$] [M]$^+$ calculated: 226.1158, found: 226.1161.

4-Fluoro-3-(3-phenylbut-3-en-1-yl)-1,1'-biphenyl (6c)

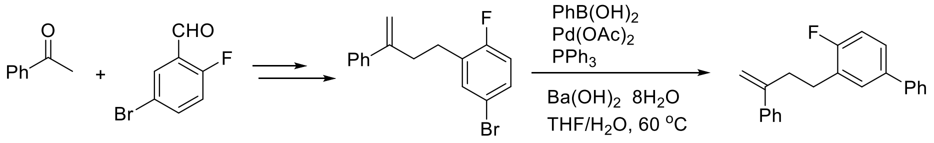

6c: 87%

Intermediate 4-Bromo-1-fluoro-2-(3-phenylbut-3-en-1-yl)benzene was obtained from the same method as **6b** and then directly underwent the cross-coupling reaction with phenylboronic acid under Pd catalyst to give compound **6c**.[14]

To a solution of 4-Bromo-1-fluoro-2-(3-phenylbut-3-en-1-yl)benzene (487 mg, 1.6 mmol) in THF (5 mL) and water 15 mL) added phenylboronic acid (234 mg, 1.9 mmol, 1.2 equiv), palladium(II) acetate (18 mg, 0.08 mmol, 0.05 equiv), triphenylphosphine (84 mg, 0.32 mmol, 0.2 equiv), and barium hydroxide octahydrate (1.00 g, 3.2 mmol, 2.0 equiv) at room temperature. After stirring for 11 h under 60 °C, the reaction mixture was cooled to room temperature, and filtered through a pad of Celite©. The filtrate was then concentrated and extracted with EtOAc. The combined organic layer was washed with brine and dried over Na_2SO_4. After filtration, the filtrate was concentrated under reduced pressure and then purificated by silica-gel column chromatography (*n*-hexane/EtOAc = 50/1) to give **6c** (420 mg, 87%) as a colorless oil. **^{1}H NMR** (500 MHz, $CDCl_3$) δ 7.62 – 7.56 (m, 2H), 7.55 – 7.51 (m, 2H), 7.51 – 7.47 (m, 2H), 7.48 – 7.36 (m, 5H), 7.39 – 7.32 (m, 1H), 7.14 (dd, J = 9.7, 8.4 Hz, 1H), 5.40 (d, J = 1.4 Hz, 1H), 5.17 (s, 1H), 2.94 (s, 4H). **^{13}C NMR** (126 MHz, $CDCl_3$) δ 161.0 (d, J = 245.6 Hz), 147.7, 140.7 (d, J = 49.6 Hz), 137.2, 129.6, 129.1, 128.9, 128.88, 128.5, 127.6, 127.3, 127.1, 126.4, 126.3, 115.6 (d, J = 22.5 Hz), 113.1, 35.9, 28.7. **^{19}F NMR** (282 MHz, $CDCl_3$) δ -121.73 (q, J = 7.2 Hz, 1F). **IR (KBr):** 3058, 3032, 2932, 2866, 1509, 1485, 1451, 1230, 1116, 905, 823, 778, 763, 697 cm^{-1}. **HRMS (EI)** [$C_{22}H_{19}F$] [M]$^+$ calculated: 302.1471, found: 302.1475.

5-(4-(2-Fluoro-5-methoxyphenyl)but-1-en-2-yl)benzo[*d*][1,3]dioxole (6d)

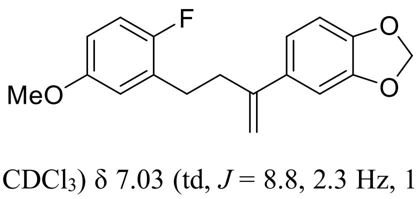

By using 1-(Benzo[*d*][1,3]dioxol-5-yl)ethan-1-one and 2-Fluoro-5-methoxybenzaldehyde as starting materials, compound **6d** was obtained from the same method as **6b** and as a colorless oil (456 mg, Yield: 69%). **^{1}H NMR** (500 MHz, $CDCl_3$) δ 7.03 (td, J = 8.8, 2.3 Hz, 1H), 6.95 (dd, J = 10.3, 2.5 Hz, 2H), 6.81 (d, J = 8.0 Hz, 1H), 6.71 – 6.57 (m, 2H), 5.97 (s, 2H), 5.22 (s, 1H), 4.99 (s, 1H), 3.79 (s, 3H), 2.74 (s, 4H). **^{13}C NMR** (126 MHz, $CDCl_3$) δ 161.6 (d, J = 244.6 Hz), 159.2 (d, J = 10.9 Hz), 147.8, 147.2, 147.1, 135.3, 130.9, 120.5 (d, J = 16.5 Hz), 119.7, 112.0, 109.6, 108.1, 106.8, 101.6 (d, J = 26.0 Hz), 101.1, 55.6, 36.3, 27.8. **^{19}F NMR** (282 MHz, $CDCl_3$) δ -116.85 – -117.18 (m, 1F). **IR (KBr):** 3080, 2936, 1627, 1505, 1490, 1442, 1285, 1232, 1189, 1152, 1110, 1038, 937, 900, 815, 736, 627 cm^{-1}. **HRMS (EI)** [$C_{18}H_{17}FO_3$] [M]$^+$ calculated: 300.1162, found: 300.1173.

1-Fluoro-2-((3-phenylbut-3-en-1-yl)oxy)benzene (6e)

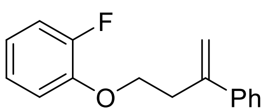

By using 3-Phenylbut-3-en-1-ol (synthesized below, see **3l**) and 2-Fluorophenol as starting materials, compound **6e** was obtained by Mitsunobu reaction: in a flame dried flask was sequentially charged 2-Fluorophenol (227 µL, 2.2 mmol), 3-Phenylbut-3-en-1-ol (430 mg, 2.9 mmol), Triphenylphosphine (760 mg, 2.9 mmol), and 10 mL anhydrous DCM under N_2 atmosphere, then stirred at 0 °C. Then DIAD (586 mg, 2.9 mmol) was added under the same temperature. The mixture was stirred for 3 h, then mixture was diluted with Et_2O, extracted with Et_2O. The combined organic phase was washed with brine and dried over Na_2SO_4, filtered and evaporated under reduced pressure. The crude was purified by column chromatography on silica gel (hexane) to give title compound as a colorless oil (496 mg, 93%). **^{1}H NMR** (300 MHz, $CDCl_3$) δ 7.48 (d, *J* = 7.0 Hz, 2H), 7.43 – 7.26 (m, 3H), 7.16 – 6.98 (m, 2H), 6.98 – 6.82 (m, 2H), 5.35 (d, *J* = 62.3 Hz, 2H), 4.15 (t, *J* = 7.2 Hz, 2H), 3.08 (t, *J* = 7.3 Hz, 2H). **^{13}C NMR** (75 MHz, $CDCl_3$) δ 152.85 (d, *J* = 245.2 Hz), 146.9, 144.3, 140.6, 128.6, 127.8, 126.2, 124.3, 121.2, 116.3 (d, *J* = 18.6 Hz), 115.1, 114.7(dd, *J* = 9.5, 6.1 Hz), 68.2, 35.2. **^{19}F NMR** (282 MHz, $CDCl_3$) δ -134.94 – -135.14 (m, 1F). **IR (KBr):** 3083, 3056, 2953, 2877, 1613, 1507, 1473, 1390, 1312, 1259, 1205, 1110, 902, 778, 705 cm^{-1}. **HRMS (EI)** [$C_{16}H_{15}FO$] $[M]^+$ calculated: 242.1107, found: 242.1096.

3.2 Synthesis of substituted alkyl fluorides 2

Alkyl fluorides **2a**, **2b**, **2c**, **2d**, **2i**, and **2j** were prepared according to known methods.[10, 15] A typical experimental procedure for the preparation of other alkyl fluorides **2e**, **2f**, **2g**, **2h**, **2k**, **2l**, **2m**, and **2n** were described below.

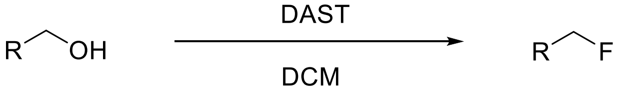

1-Fluorodecane (2e)[16]

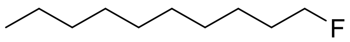

To the solution of DAST (1.45 mL, 11 mmol) in the DCM (10 mL) was added a solution of 1-Decanol (1.58 g, 10 mmol) in DCM (2 mL) dropwise at 0 °C. Then the mixture was moved to room temperature and stirred for 6 h. After that, the reaction mixture was poured into cooled water, neutralized with saturated $NaHCO_3$, and then extracted with DCM. The combined organic phase was dried over Na_2SO_4, filtered and evaporated under reduced pressure. The crude was purified by column chromatography on silica gel (hexane) to give title compound as colorless oil (1.17 g, 73%). **^{1}H NMR** (300 MHz, $CDCl_3$) δ 4.51 (t, *J* = 6.1 Hz, 1H), 4.35 (t, *J* = 6.1 Hz, 1H), 1.80 – 1.57 (m, 2H), 1.27 (s, 14H), 0.88 (t, *J* = 6.4 Hz, 3H). **^{19}F NMR** (282 MHz, $CDCl_3$) δ -215.1 – -220.8 (m, 1F). **MS(EI)**: *m/z* 160 $[M]^+$. The chemical shifts were consistent with those reported in the literature.

1-Fluoro-3-phenylpropane (2f)[17]

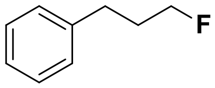

To the solution of DAST (2.0 mL, 15 mmol) in the DCM (10 mL) was added a solution of 3-Phenyl-1-propanol (1.36 g, 10 mmol) in DCM (2 mL) dropwise at -40 °C. Then the mixture was moved to room temperature and stirred for 6 h. After that, the reaction mixture was poured into cooled water, neutralized with saturated $NaHCO_3$, and then extracted with DCM. The combined organic phase was dried over Na_2SO_4, filtered and evaporated under reduced pressure. The crude was purified by column chromatography on silica gel (hexane) to give title compound as colorless oil (1.0 g, 72%). **^{1}H NMR** (300 MHz, $CDCl_3$) δ 7.37 – 7.12 (m, 5H), 4.44 (dt, *J* = 47.2, 5.9 Hz, 2H), 2.74 (t, *J* = 7.6 Hz, 2H), 2.25 – 1.80 (m, 2H). **^{19}F NMR** (282 MHz, $CDCl_3$) δ -220.47 (tt, *J* = 48.5, 25.3 Hz, 1F). **MS(EI)**: *m/z* 138 $[M]^+$. The chemical shifts were consistent with those reported in the literature.

2-Fluoroethylbenzene (2g)[18]

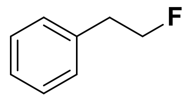

To the solution of DAST (2.0 mL, 15 mmol) in the DCM (10 mL) was added a solution of 2-Phenylethyl alcohol (1.22 g, 10 mmol) in DCM (2 mL) dropwise at -40 °C. Then the mixture was moved to room temperature and stirred for 6 h. After that, the reaction mixture was poured into cooled water, neutralized with saturated $NaHCO_3$, and then extracted with DCM. The combined organic phase was dried over Na_2SO_4, filtered and evaporated under reduced pressure. The crude was purified by column chromatography on silica gel (hexane) to give title compound as colorless oil (0.83 g, 67%). **^{1}H NMR** (300 MHz, $CDCl_3$) δ 7.51 – 7.03 (m, 5H), 4.63 (dt, *J* = 47.1, 6.6 Hz, 2H), 3.02 (dt, *J* = 22.9, 6.6 Hz, 2H). **^{19}F NMR** (282 MHz, $CDCl_3$) δ -215.75 (tt, *J* = 46.7, 23.0 Hz, 1F). **MS(EI)**: *m/z* 124 $[M]^+$. The chemical shifts were consistent with those reported in the literature.

6-Fluorohex-1-ene (2k)[19]

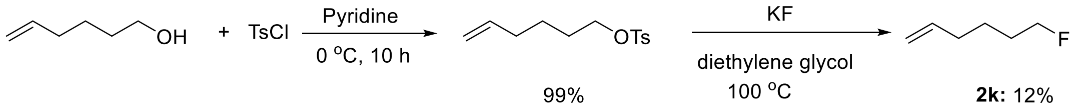

5-Hexen-1-ol (2.4 mL, 20 mmol in 10 mL of pyridine at 0 ℃) was added to a solution of *p*-toluenesulfonyl chloride (3.82 g, 20 mmol) in 10 mL of pyridine and was stirred for 10 h at 0 ℃. After checked the TLC, the mixture was added onto a crushed ice and conc. HCl mixture, extracted with diethyl ether, dried with Na_2SO_4 and the solvent evaporated in vaccuo and purified to give colorless oil in quantitative. Anhydrous potassium fluoride (1.4 g, 24 mmol) was placed into a dry two-neck 30 mL flask. One neck was capped with a rubber septum, the other was connected to a cold trap, and the apparatus was flushed with nitrogen. The former product dissolved in anhydrous diethylene glycol (5 mL) was injected to the reaction flask. The rubber septum was replaced with a glass stopper and the flask was heated to 100 ℃ with vigorous stirring. The volatile material was allowed to distil from the reaction mixture under reduced pressure (60 Torr) and collected in the cold trap affording the title product (250 mg, 12%). **^{1}H NMR** (300 MHz, $CDCl_3$) δ 6.06 – 5.54 (m, 1H), 5.14 – 4.85 (m, 2H), 4.53 (q, *J* = 6.3 Hz, 1H), 4.38 (q, *J* = 6.3 Hz, 1H), 2.10 (q, *J* = 7.0 Hz, 2H), 1.86 – 1.58 (m, 2H), 1.61 – 1.39 (m, 2H). **^{19}F NMR** (282 MHz, $CDCl_3$) δ -218.80 (tt, *J* = 48.1, 25.0 Hz, 1F). **MS(EI)**: *m/z* 102 $[M]^+$. The chemical shifts were consistent with those reported in the literature.

10-Fluorodec-1-ene (2l)[20]

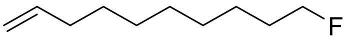

To the solution of DAST (1.5 mL, 12 mmol) in the DCM (10 mL) was added a solution of 9-Decen-1-ol (1.56 g, 10 mmol) in DCM (10 mL) dropwise at -40 °C. Then after 4 h the mixture was slowly warm up to room temperature and keep stirring for 6 h. After that, the reaction mixture was poured into cooled water, neutralized with saturated $NaHCO_3$, and then extracted with DCM. The combined organic phase was dried over Na_2SO_4, filtered and evaporated under reduced pressure. The crude was purified by column chromatography on silica gel (hexane) to give title compound as colorless oil (1.1 g, 67%). **^{1}H NMR** (300 MHz, $CDCl_3$) δ 5.96 – 5.67 (m, 1H), 4.96 (dd, *J* = 18.3, 13.6 Hz, 2H), 4.44 (dt, *J* = 47.5, 6.3 Hz, 2H), 2.04 (q, *J* = 6.9 Hz, 2H), 1.88 – 1.51 (m, 2H), 1.31 (s, 10H). **^{19}F NMR** (282 MHz, $CDCl_3$) δ -218.49 (tt, *J* = 48.0, 24.7 Hz, 1F). **MS(EI)**: *m/z* 158 $[M]^+$. The chemical shifts were consistent with those reported in the literature.

1,8-Difluorooctane (**2m**)[21]

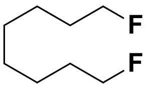

To the solution of DAST (3.3 mL, 25 mmol) in the DCM (10 mL) was added a solution of 1,8-Octanediol (1.46 g, 10 mmol) in DCM (5 mL) dropwise at 0 °C. Then the mixture was moved to room temperature and then stirred at 40 °C for 6 h. After that, the reaction mixture was poured into cooled water, neutralized with saturated $NaHCO_3$, and then extracted with DCM. The combined organic phase was dried over Na_2SO_4, filtered and evaporated under reduced pressure. The crude was purified by column chromatography on silica gel (hexane) to give title compound as colorless oil (0.68 g, 45%). **^{1}H NMR** (300 MHz, $CDCl_3$) δ 4.42 (dt, *J* = 47.4, 6.1 Hz, 4H), 1.86 – 1.52 (m, 4H), 1.55 – 1.15 (m, 8H). **^{19}F NMR** (282 MHz, $CDCl_3$) δ -218.48 (tt, *J* = 48.2, 24.9 Hz, 2F). **MS(EI)**: *m/z* 150 $[M]^+$. The chemical shifts were consistent with those reported in the literature.

(3-Fluorobutyl)benzene (2n)[17] (**No desired carbosilylation product detected**)

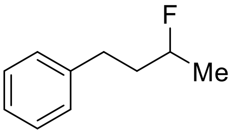

To the solution of DAST (1.3 mL, 8.8 mmol) in the DCM (10 mL) was added a solution of 4-phenylbutan-2-ol (1.2 g, 8 mmol) in DCM (5 mL) dropwise at -40 °C. Then after 4 h the mixture was slowly warm up to room temperature and keep stirring for 6 h. After that, the reaction mixture was poured into cooled water, neutralized with saturated $NaHCO_3$, and then extracted with DCM. The combined organic phase was dried over Na_2SO_4, filtered and evaporated under reduced pressure. The crude was purified by column chromatography on silica gel (hexane) to give title compound as colorless oil (0.52 g, 43%). **^{1}H NMR** (300 MHz, $CDCl_3$) δ 7.47 – 6.91 (m, 5H), 4.67 (d, *J* = 47.2 Hz, 1H), 2.95 – 2.50 (m, 2H), 2.25 – 1.64 (m, 2H), 1.34 (dd, *J* = 23.9, 6.1 Hz, 3H). **^{19}F NMR** (282 MHz, $CDCl_3$) δ -172.44 – -177.88 (m, 1F). **MS(EI)**: *m/z* 152 $[M]^+$. The chemical shifts were consistent with those reported in the literature.

1-(3-Fluoropropyl)adamantane (2h)[22,23]

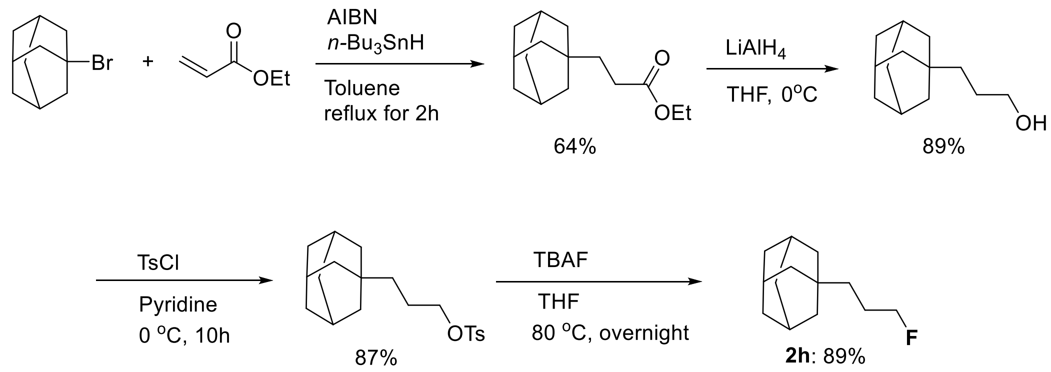

To a stirred solution of 1-bromoadamantane (5.0 g, 23.2 mmol) in toluene (80 mL) was added *n*-Bu_3SnH (7.50 mL, 27.9 mmol), ethyl acrylate (5.0 mL, 46.5 mmol) and AIBN (191 mg, 1.16 mmol) at room temperature. The resulting mixture was stirred at reflux for 2 h. After cooling to room temperature, the mixture was diluted with an aqueous solution of NH_4OH (0.20 M, 20.0 mL, 4.00 mmol) and extracted with Et_2O (4 × 100 mL). The combined organic phases were washed with brine, dried over Na_2SO_4, filtered and concentrated in vacuo. The residue was obtained was purified (hexanes → EtOAc:hexanes 1:20) to give the corresponding ethyl ester (3.5 g, 64%) as a clear oil.
To a stirred dry THF (20 mL) solution of $LiAlH_4$ (0.54 g, 14.2 mmol) was added this ethyl ester (3.0 g, 12.7 mmol) solution in THF (5 mL) at 0 °C. The mixture was stirred at that temperature for 5 h. After the reaction completed, then slowly quenched with water (5 mL) and aqueous NaOH (5 mL, 4N) extracted with Et_2O (3 × 50 mL). The combined organic phases were washed with brine, dried over Na_2SO_4, filtered and concentrated in vacuo to give 3-(Adamantan-1-yl)propan-1-ol (2.2 g, 89%) as a white solid.
3-(Adamantan-1-yl)propan-1-ol (2.13 g, 11 mmol in 10 mL of pyridine at 0 ℃) was added to a solution of *p*-toluenesulfonyl chloride (2.1 g, 11 mmol) in 10 mL of pyridine and was stirred for 10 h at 0 ℃. After checked the TLC, the mixture was added onto a crushed ice and conc. HCl mixture, extracted with diethyl ether, dried over $Na_2SO_4$and the solvent evaporated in vaccuo and purified to give 3-(Adamantan-1-yl)propyl 4-methylbenzenesulfonate as a white solid (3.34 g, 87%).
In a glovebox, a flame dried flask was charged with 3-(Adamantan-1-yl)propyl 4-methylbenzenesulfonate (2.8 g, 8.0 mmol) and Tetrabutylammonium fluoride (TBAF,16 mL, 1 M in THF). Then the flask was capped with a rubber septum before move out from glovebox. The mixture was then heated at 80 °C overnight. After that, the reaction mixture extracted with DCM. The combined organic phase was washed with brine and dried over Na_2SO_4, evaporated under reduced pressure. The crude was purified by column chromatography on silica gel (hexane) to give title compound as white solid (1.41 g, 89%). **^{1}H NMR** (300 MHz, $CDCl_3$) δ 4.41 (dt, J = 47.5, 6.3 Hz, 2H), 1.95 (s, 3H), 1.66 (q, J = 11.6, 10.9 Hz, 8H), 1.47 (d, J = 2.8 Hz, 6H), 1.27 – 0.91 (m, 2H). **^{13}C NMR** (75 MHz, $CDCl_3$) δ 85.2 (d, J = 164.8 Hz), 42.4, 39.7, 37.3, 32.0, 28.8, 23.9 (d, J = 19.2 Hz). **^{19}F NMR** (282 MHz, $CDCl_3$) δ -217.33 (tt, J = 47.1, 23.6 Hz, 1F). **IR (KBr)**: 3006, 2934, 2896, 2847, 1452, 1390, 1052, 1007, 983, 888, 629 cm^{-1}. **HRMS (EI)** [$C_{13}H_{21}F$] $[M]^+$ calculated: 196.1627, found: 196.1622.

3.3 Synthesis of alkenes 3

Alkenes **3a, 3b, 3c, 3e, 3j, 3k** were purchased from TCI or Sigma Aldrich. Alkenes **3d**, **3f**, **3g**, **3h**, **3i**, **3l**, **3m**, **3n**, **3o, 3p** were prepared according to known methods. A typical experimental procedure for the preparation of alkenes were described below.

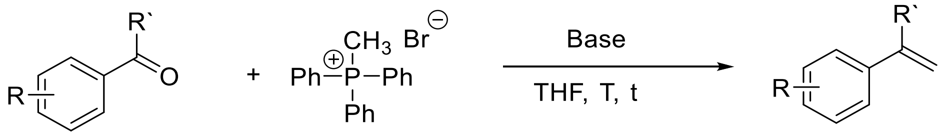

1-(*tert*-Butyl)-4-vinylbenzene (3d)[24]

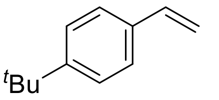

A flame dried flask was charged with 4-(*tert*-Butyl)benzaldehyde (0.84 mL, 5 mmol), K_2CO_3 (1.1 g, 8 mmol) and Methyltriphenylphosphonium bromide (2.15 g, 6 mmol) in anhydrous THF (20 mL) and heated at reflux overnight. The reaction mixture was then cooled, filtered, washed with Hexane, then concentrated under vacuum, and purified by column chromatography on silica gel (Hexane) to give title compound as colorless oil (0.69 g, 86%). **^{1}H NMR** (300 MHz, $CDCl_3$) δ 7.50 (m, 4H), 6.85 (dd, *J* = 17.7, 10.8 Hz, 1H), 5.86 (d, *J* = 16.8 Hz, 1H), 5.34 (d, *J* = 10.7 Hz, 1H), 1.47 (t, *J* = 1.8 Hz, 9H). **MS(EI)**: *m/z* 160 $[M]^+$. The chemical shifts were consistent with those reported in the literature.

1-Methoxy-4-vinylbenzene (3f)[25]

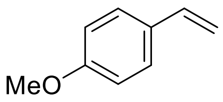

A flame dried flask was charged with 4-Methoxybenzaldehyde (1.22 mL, 10 mmol), K_2CO_3 (2.2 g, 16 mmol) and Methyltriphenylphosphonium bromide (4.28 g, 12 mmol) in anhydrous THF (20 mL) and heated at reflux overnight. The reaction mixture was then cooled, filtered, washed with Hexane, then concentrated under vacuum, and purified by column chromatography on silica gel (10% EtOAc in Hexane) to give title compound as colorless oil (0.656 g, 54%). **^{1}H NMR** (300 MHz, $CDCl_3$) δ 7.39 (d, *J* = 8.7 Hz, 2H), 6.90 (d, *J* = 8.5 Hz, 2H), 6.71 (dd, *J* = 17.5, 10.8 Hz, 1H), 5.66 (d, *J* = 17.6 Hz, 1H), 5.17 (d, *J* = 10.9 Hz, 1H), 3.84 (s, 3H). **MS(EI)**: *m/z* 134 $[M]^+$. The chemical shifts were consistent with those reported in the literature.

1-Methoxy-2-vinylbenzene (3g)[26]

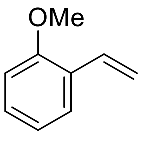

A flame dried flask was charged with 2-Methoxybenzaldehyde (1.36 g, 10 mmol), K_2CO_3 (2.2 g, 16 mmol) and Methyltriphenylphosphonium bromide (4.28 g, 12 mmol) in anhydrous THF (20 mL) and heated at reflux overnight. The reaction mixture was then cooled, filtered, washed with hexane, then concentrated under vacuum, and purified by column chromatography on silica gel (10% EtOAc in Hexane) to give title compound as colorless oil (1.29 g, 7%). **^{1}H NMR** (300 MHz, $CDCl_3$) δ 7.35 (d, *J* = 7.6 Hz, 1H), 7.11 (d, *J* = 7.5 Hz, 1H), 6.95 (dd, *J* = 17.4, 11.0 Hz, 1H), 6.80 (t, *J* = 7.2 Hz, 1H), 6.72 (d, *J* = 8.1 Hz, 1H), 5.62 (d, *J* = 18.5 Hz, 1H), 5.14 (d, *J* = 11.1 Hz, 1H), 3.68 (s, 3H). **MS(EI)**: *m/z* 134 $[M]^+$. The chemical shifts were consistent with those reported in the literature.

5-Vinylbenzo[*d*][1,3]dioxole (3h)[27]

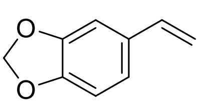

A flame dried flask was charged with Piperonal (0.75 g, 5 mmol), K_2CO_3 (1.1 g, 8 mmol) and Methyltriphenylphosphonium bromide (2.15 g, 6 mmol) in anhydrous THF (20 mL) and heated at reflux overnight. The reaction mixture was then cooled, filtered, washed with Hexane, then concentrated under vacuum, and purified by column chromatography on silica gel (10% EtOAc in Hexane) to give title compound as colorless oil (0.64 g, 86%). **^{1}H NMR** (300 MHz, $CDCl_3$) δ 6.99 (s, 1H), 6.91 – 6.81 (m, 1H), 6.82 – 6.73 (m, 1H), 6.65 (dd, *J* = 17.5, 10.9 Hz, 1H), 5.96 (s, 2H), 5.60 (d, *J* = 17.5 Hz, 1H), 5.15 (d, *J* = 10.8 Hz, 1H). **MS(EI)**: *m/z* 148 $[M]^+$. The chemical shifts were consistent with those reported in the literature.

Methyl(4-vinylphenyl)sulfane (3i)[28]

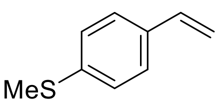

A flame dried flask was charged with 4-(Mmethylthio)benzaldehyde (1.3 mL, 10 mmol), K_2CO_3 (2.2 g, 16 mmol) and Methyltriphenylphosphonium bromide (4.28 g, 12 mmol) in anhydrous THF (20 mL) and heated at reflux overnight. The reaction mixture was then cooled, filtered, washed with Hexane, then concentrated under vacuum, and purified by column chromatography on silica gel (10% EtOAc in Hexane) to give title compound as colorless oil (1.38 g, 92%). **^{1}H NMR** (300 MHz, $CDCl_3$) δ 7.44 – 7.33 (m, 2H), 7.33 – 7.19 (m, 2H), 6.85 – 6.61 (m, 1H), 5.77 (dd, *J* = 17.6, 3.9 Hz, 1H), 5.27 (dd, *J* = 10.9, 4.0 Hz, 1H), 2.50 (s, 3H). **MS(EI)**: *m/z* 150 $[M]^+$. The chemical shifts were consistent with those reported in the literature.

(*E*)-Buta-1,3-dien-1-ylbenzene (3n)[29]

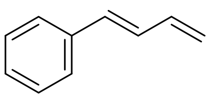

A flame dried flask was charged Methyltriphenylphosphonium bromide (3.57 g, 10 mmol) and anhydrous THF (50 mL), stirred at 0 °C. Then *n*-BuLi (6.25 mL, 10 mmol, 1.6 M in THF) was added dropwise, stirred at 0 °C for 15 min. followed by the charge the solution of Cinnamaldehyde (1.0 mL, 8 mmol) in anhydrous THF (10 mL), then stirred for 1 h. Then warm up to room temperature and keep stirred for another 1 h. The reaction mixture was quenched by saturated NH_4Cl, extracted by Et_2O, washed by saturated brine, dried and concentrated under vacuum, purified by column chromatography on silica gel (Hexane) to give title compound as colorless oil (0.87 g, 84%). **^{1}H NMR** (300 MHz, $CDCl_3$) δ 7.43 (d, *J* = 7.7 Hz, 2H), 7.33 (t, *J* = 6.6 Hz, 2H), 7.26 (d, *J* = 5.3 Hz, 1H), 6.82 (dd, *J* = 15.1, 10.1 Hz, 1H), 6.58 (d, *J* = 15.2 Hz, 1H), 6.55 – 6.43 (m, 1H), 5.36 (d, *J* = 16.8 Hz, 1H), 5.20 (d, *J* = 10.0 Hz, 1H). **MS(EI)**: *m/z* 130 $[M]^+$. The chemical shifts were consistent with those reported in the literature.

(1-Cyclopropylvinyl)benzene (3p)[30]

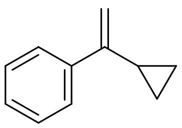

A flame dried flask was charged Methyltriphenylphosphonium bromide (1.8 g, 5 mmol) and anhydrous THF (10 mL), stirred at 0 °C. Then *n*-BuLi (3.2 mL, 5 mmol, 1.56 M in THF) was added dropwise, stirred at 0 °C for 15 min. followed by the charge the solution of Cyclopropyl phenyl ketone (1.0 mL, 8 mmol) in anhydrous THF (5 mL), then stirred for 1 h. Then warm up to room temperature and keep stirred for another 1 h. The reaction mixture was quenched by saturated NH_4Cl, extracted by Et_2O, washed by saturated brine, dried and concentrated under vacuum, purified by column chromatography on silica gel (Hexane) to give title compound as colorless oil (0.33 g, 91%). **^{1}H NMR** (300 MHz,

$CDCl_3$) δ 7.68 (d, *J* = 7.2 Hz, 2H), 7.40 (dt, *J* = 12.5, 6.5 Hz, 3H), 5.19 (d, *J* = 104.2 Hz, 2H), 1.73 (p, *J* = 7.9 Hz, 1H), 0.91 (d, *J* = 7.9 Hz, 2H), 0.68 (d, *J* = 4.9 Hz, 2H). **MS(EI)**: *m/z* 144 $[M]^+$. The chemical shifts were consistent with those reported in the literature.

Buta-1,3-dien-2-ylbenzene (3o)[31]

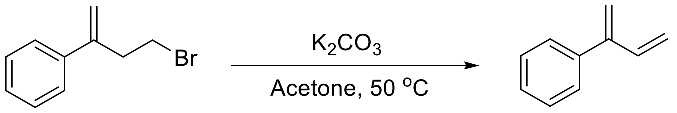

3o, 76%

A flame dried flask was charged with (4-Bromobut-1-en-2-yl)benzene (0.633 g, 3 mmol), K_2CO_3 (0.83 g, 6 mmol) and anhydrous Acetone (10 mL), and then stirred at 50 °C overnight. The reaction mixture was quenched by saturated NH_4Cl, extracted by Et_2O, washed by saturated brine, dried and concentrated under vacuum, purified by column chromatography on silica gel (Hexane) to give title compound as colorless oil (0.15 g, 76%). **^{1}H NMR** (300 MHz, $CDCl_3$) δ 7.35 (s, 5H), 6.64 (dd, *J* = 17.3, 10.8 Hz, 1H), 5.41 – 5.11 (m, 4H). **MS(EI)**: *m/z* 130 $[M]^+$. The chemical shifts were consistent with those reported in the literature.

(4-Methoxybut-1-en-2-yl)benzene (3l)[32,33]

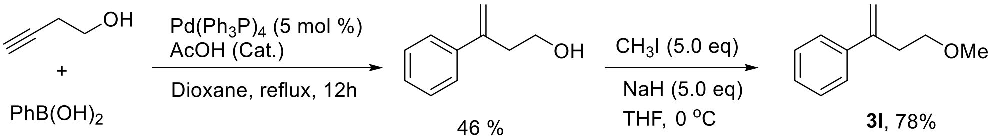

3l, 78%

A flame dried flask was charged with but-3-yn-1-ol (1.5 mL, 20 mmol), $Pd(PPh_3)_4$ (578 mg, 2.5 mol%) and phenylboronic acid (3.7 g, 30.0 mmol) under an argon atmosphere. Degassed dry 1,4-dioxane (20 mL) and AcOH (0.17 mL, 20.0 mol%) were added and the solution was stirred at r.t. for 15 min, then reflux for 12 h. The reaction was cooled to r.t. and the 1,4- dioxane was removed in vacuo. The resulting crude product was purified by flash column chromatography (1/8 EtOAc/Hexane to 1/1/5 DCM/EtOAc/Hexane) to afford 3-phenylbut-3-en-1-ol (1.50 g, 46%) as a light yellow oil.

The 3-phenylbut-3-en-1-ol (810 mg, 5 mmol) was dissolved in 15 mL dry THF, stirred at room temperature, then treated with NaH (600 mg, 25 mmol, 60% in mineral oil). After stirring 30 min, CH_3I (2.67 mL, 25 mmol) was then added slowly. The mixture was stirred at room temperature for 2h, and the reaction progress was monitored by TLC. To this mixture was slowly added water 10 mL, and then extracted with EtOAc (20 mL × 3). The combined organic phases were washed with brine (10 mL), and dried over Na_2SO_4. After filtration, the filtrate was concentrated under reduced pressure. The residue was purified by column chromatography on silica gel (Hexane) to give title compound as white solid (631 mg, 78%).**^{1}H NMR** (300 MHz, $CDCl_3$) δ 7.54 – 7.22 (m, 5H), 5.36 (d, *J* = 1.4 Hz, 1H), 5.15 (d, *J* = 1.4 Hz, 1H), 3.50 (t, *J* = 7.1 Hz, 2H), 3.34 (s, 3H), 2.81 (t, *J* = 6.9 Hz, 2H). **HRMS (EI)** [$C_{11}H_{14}O$] $[M]^+$ calculated: 162.1045, found: 162.1041. The chemical shifts were consistent with those reported in the literature.

3-Vinylestrone derivative (3m)

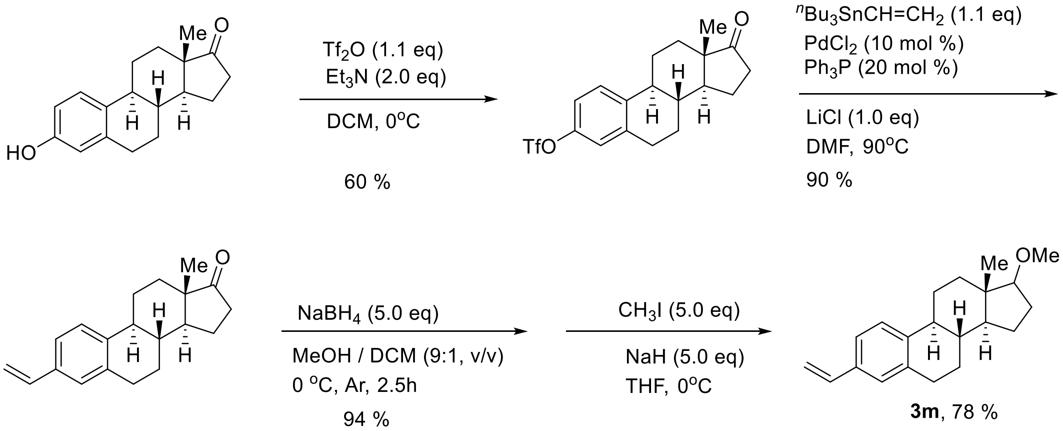

3m, 78 %

Synthetic intermediates were prepared according to procedures previously described.[34,35]

The alcohol intermediate (340 mg, 1.2 mmol) was dissolved in 10 mL dry THF, stirred at 0 °C, then treated with NaH (150 mg, 6 mmol, 60% in mineral oil). After stirring 30 min, CH_3I (0.38 mL, 6 mmol) was then added slowly. The mixture was stirred at room temperature for 2h, and the reaction progress was monitored by TLC. To this mixture was added water 5 mL, and then extracted with EtOAc (10 mL × 3). The combined organic phases were washed with brine (10 mL), and dried over Na_2SO_4. After filtration, the filtrate was concentrated under reduced pressure. The residue was purified by column chromatography on silica gel (Hexane) to give title compound as a white solid (190 mg, 78%). m.p. = 101.1 – 102.4 °C. **^{1}H NMR** (300 MHz, $CDCl_3$) δ 7.39 – 7.05 (m, 3H), 6.82 – 6.52 (m, 1H), 5.72 (d, *J* = 17.6 Hz, 1H), 5.20 (d, *J* = 10.9 Hz, 1H), 3.40 (s, 3H), 3.34 (t, *J* = 8.4 Hz, 1H), 2.99 – 2.77 (m, 2H), 2.39 – 2.17 (m, 2H), 2.08 (d, *J* = 9.2 Hz, 2H), 1.91 (d, *J* = 10.5 Hz, 1H), 1.78 – 1.12 (m, 9H), 0.81 (s, 3H). **^{13}C NMR** (75 MHz, $CDCl_3$) δ 140.3, 136.9, 136.8, 135.0, 126.9, 125.6, 123.5, 113.0, 90.8, 58.0, 50.4, 44.5, 43.3, 38.4, 38.1, 29.6, 27.8, 27.3, 26.3, 23.2, 11.6. **IR (KBr)**: 3083, 3065, 2981, 2957, 2919, 2863, 2818, 1828, 1627, 1497, 1450, 1265, 1198, 1103, 1080, 911, 831, 734, 649 cm^{-1}. **HRMS (EI)** [$C_{21}H_{28}O$] $[M]^+$ calculated: 296.2140, found: 296.2141.

3.4 Synthesis of trialkyl(4,4,5,5-tetramethyl-1,3,2-dioxaborolan-2-yl)silane

Dimethyl(phenyl)(4,4,5,5-tetramethyl-1,3,2-dioxaborolan-2-yl)silane was purchased from TCI, other silylboranate $R_3SiBPin$ were prepared according to procedures previously reported.[10,36,37]

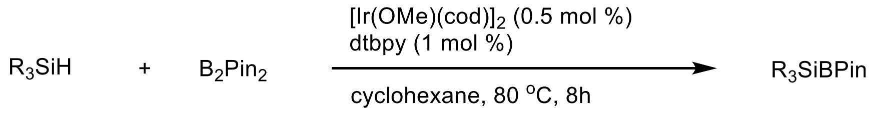

Triethyl(4,4,5,5-tetramethyl-1,3,2-dioxaborolan-2-yl)silane

An oven-dried vial was charged with $[Ir(COD)OMe]_2$ (66.3 mg, 0.1 mmol), dtbpy (54.0 mg, 0.2 mmol), B_2pin_2 (5.1 g, 20 mmol), cyclohexane (10.0 mL), and Triethylsilane (12.8 mL, 80 mmol) inside a nitrogen filled glovebox. The resulting dark brown solution was heated at 80 °C overnight outside the glovebox. After being cooled to room temperature, the crude reaction mixture was concentrated in vacuo, and the residue was purified by flash column chromatography to afford the $Et_3SiBpin$ as colorless oil (4.2 g, 87%). **^{1}H NMR** (300 MHz, $CDCl_3$) δ 1.22 (s, 12H), 0.95 (t, J = 8.0 Hz, 9H), 0.59 (dd, J = 15.8, 7.9 Hz, 6H). **MS(EI)**: *m/z* 242 $[M]^+$. The chemical shifts were consistent with those reported in the literature.

Tripropyl(4,4,5,5-tetramethyl-1,3,2-dioxaborolan-2-yl)silane

An oven-dried vial was charged with $[Ir(COD)OMe]_2$ (16.5 mg, 0.025 mmol), dtbpy (13.5 mg, 0.05 mmol), B_2pin_2 (1.3 g, 5 mmol), cyclohexane (2.0 mL), and Tripropylsilane (4.2 mL, 20 mmol) inside a nitrogen filled glovebox. The resulting dark brown solution was heated at 80 °C overnight outside the glovebox. After being cooled to room temperature, the crude reaction mixture was concentrated in vacuo, and the residue was purified by flash column chromatography to afford the $^nPr_3SiBpin$ as colorless oil (0.61 g, 43%). **^{1}H NMR** (300 MHz, $CDCl_3$) δ 1.34 (dt, J = 15.4, 7.3 Hz, 6H), 1.21 (s, 12H), 0.94 (t, J = 7.0 Hz, 9H), 0.75 – 0.44 (m, 6H). **MS(EI)**: *m/z* 269 $[M–CH_3]^+$. The chemical shifts were consistent with those reported in the literature.

***tert*-Butyldimethyl(4,4,5,5-tetramethyl-1,3,2-dioxaborolan-2-yl)silane**

An oven-dried vial was charged with $[Ir(COD)OMe]_2$ (16.5 mg, 0.025 mmol), dtbpy (13.5 mg, 0.05 mmol), B_2pin_2 (1.3 g, 5 mmol), cyclohexane (2.0 mL), and *tert*-Butyldimethylsilane (3.3 mL, 20 mmol) inside a nitrogen filled glovebox. The resulting dark brown solution was heated at 80 °C overnight outside the glovebox. After being cooled to room temperature, the crude reaction mixture was concentrated in vacuo, and the residue was purified by flash column chromatography to afford the $^nPr_3SiBpin$ as a white solid (0.56 g, 46%). **^{1}H NMR** (300 MHz, $CDCl_3$) δ 1.23 (s, 12H), 0.91 (s, 9H), 0.01 (s, 6H). **MS(EI)**: *m/z* 185 $[M–^tBu]^+$. The chemical shifts were consistent with those reported in the literature.

4. General Procedure for the Carbosilylation Reactions of Alkenes

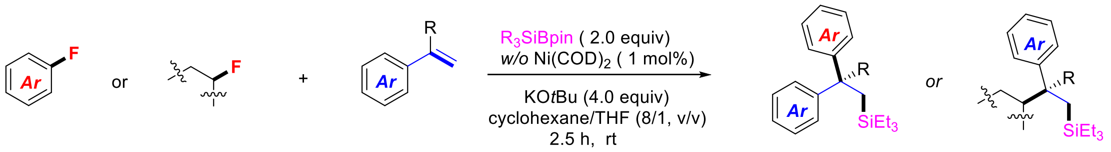

1 **2** **3** (2.0 eq) **4** **5**

In a N_2 filled glovebox, to a flame-dried screw-capped test tube were added Aryl/Alkyl fluoride **1** or **2** (0.20 mmol, 1.0 equiv), Silyl boronates (0.4 mmol, 2.0 equiv), with or without Ni(cod)$_2$ (1 mol %), Alkenes **3** (0.40 mmol, 2.0 equiv), KO*t*Bu (90mg, 0.8 mmol, 4.0 equiv) and cyclohexane/THF (1.5 mL, 8/1, *v/v*) sequentially. The tube then was sealed and removed from the glovebox. The solution was stirred at room temperature for 2.5h. The reaction tube was added *n*-Hexane (5 mL), then subject to filter through a short silica pad, and washed with Et_2O, concentrated under vacuum, followed by 3-Fluoropyridine (8.6 µL, 0.1 mmol) as an internal standard. After NMR analysis was conducted. The mixture was then concentrated again to give the residue, which was purified by column chromatography on silica gel to give the corresponding carbosilylation products **4** or **5**.

5. Unsuccessful Substrates for the Carbosilylation of Alkenes with Organic Fluorides

5.1 Organic fluorides

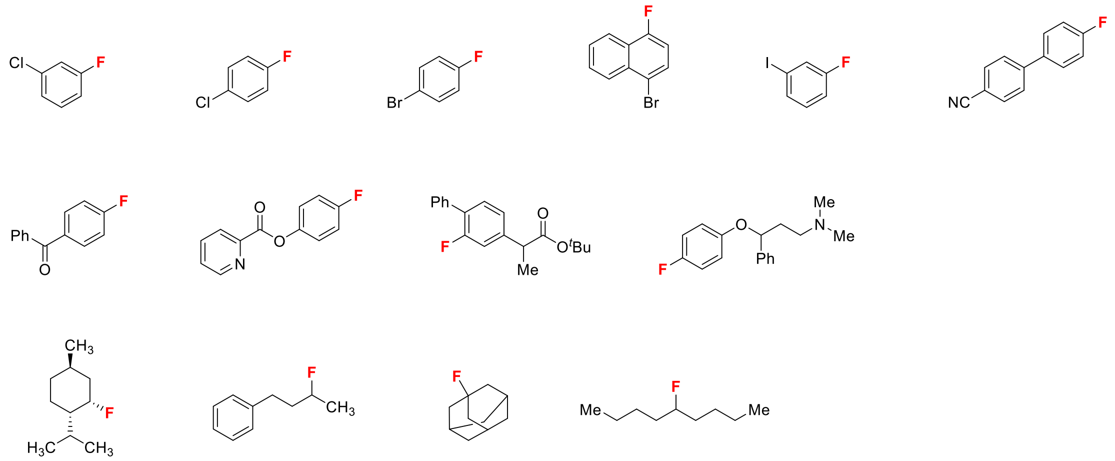

Supplementary Figure 1. Unsuccessful organic fluorides

5.2 Alkenes

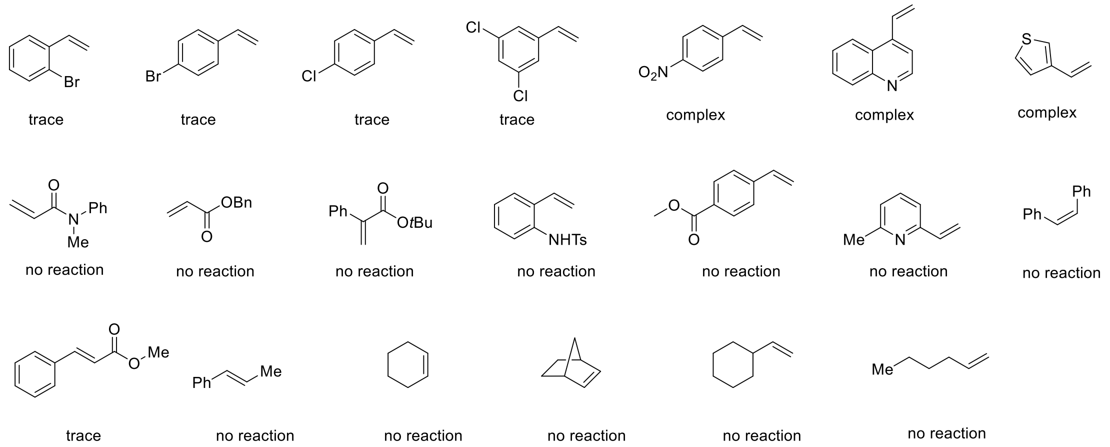

Supplementary Figure 2. Unsuccessful alkenes

6. Competition between Carbosilylation, Silylation and/or Borylation of Halogen-containing Compounds

6.1 Comparisons of aryl halides with styrene for the carbosilylation

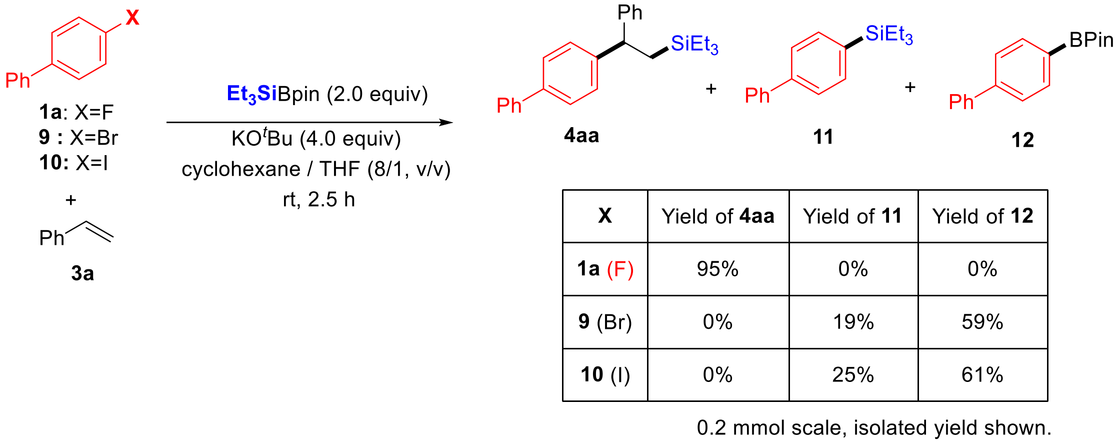

X	Yield of **4aa**	Yield of **11**	Yield of **12**
**1a** (F)	95%	0%	0%
**9** (Br)	0%	19%	59%
**10** (I)	0%	25%	61%

0.2 mmol scale, isolated yield shown.

Supplementary Figure 3. Comparisons of aryl halides with styrene for the carbosilylation

Following the general procedure, charging aryl halides (0.2 mmol), **3a** (46 μL, 0.4 mmol), silyl boronate (0.4 mmol, 2.0 equiv), KO*t*Bu (90 mg, 0.8 mmol, 4.0 equiv), and then cyclohexane (1.3 mL), anhydrous THF (0.17 mL) sequentially. And then move out from glovebox, and stirred at room temperature for 2.5 h. The reaction mixtures were analyzed by TLC before workup, then purified by column chromatography on silica gel to give corresponding compounds.

6.2 Competitive reaction between aryl halides with styrene for the carbosilylation

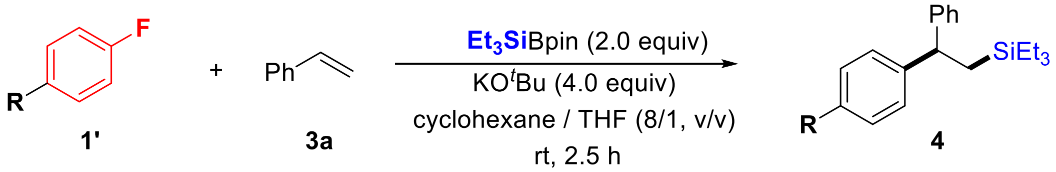

R	Yield of **4**
**1f** (H)	**4fa**: 63% (isolated)
**1'a** (Br)	4%
**1'b** (I)	5%

0.2 mmol scale, NMR yield shown.

Supplementary Figure 4. Competitive reaction between aryl halides with styrene for the carbosilylation

Following the general procedure, aryl halides (0.2 mmol), **3a** (46 μL, 0.4 mmol), silyl boronate (0.4 mmol, 2.0 equiv), KO*t*Bu (90 mg, 0.8 mmol, 4.0 equiv), and then cyclohexane (1.3 mL), anhydrous THF (0.17 mL) sequentially. And then move out from glovebox, and stirred at room temperature for 2.5 h. The reaction tube was added *n*-Hexane (5 mL), then subject to filter through a short silica pad, and washed with Et_2O, concentrated under vacuum, followed by 3-Fluoropyridine (8.6 μL, 0.1 mmol) as an internal standard. Then the ^{1}H NMR analysis and ^{19}F NMR analysis of the crude mixture were conducted to show the corresponding yields.

6.3 Comparisons of alkyl halides with styrene for the carbosilylation

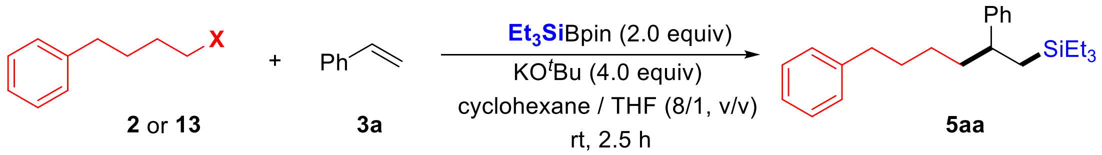

X	Yield of **5aa**
**2a** (F)	96%
**13** (Cl)	0%
**13-Br** (Br)	0%
**13-I** (I)	0%

0.2 mmol scale, NMR yield shown.

Supplementary Figure 5. Comparisons of alkyl halides with styrene for the carbosilylation

Following the general procedure, charging alkyl halides (0.2 mmol), **3a** (46 μL, 0.4 mmol), silyl boronate (0.4 mmol, 2.0 equiv), KO*t*Bu (90 mg, 0.8 mmol, 4.0 equiv), and then cyclohexane (1.3 mL), anhydrous THF (0.17 mL) sequentially. And then move out from glovebox, and stirred at room temperature for 2.5 h. The reaction tube was added *n*-Hexane (5 mL), then subject to filter through a short silica pad, and washed with Et_2O, concentrated under vacuum, followed by 3-Fluoropyridine (8.6 μL, 0.1 mmol) as an internal standard. Then the ^{1}H NMR analysis and ^{19}F NMR analysis of the crude mixture were conducted to show the corresponding yields.

6.4 Competitive reaction between alkyl fluoride and alkyl chloride with styrene for the carbosilylation

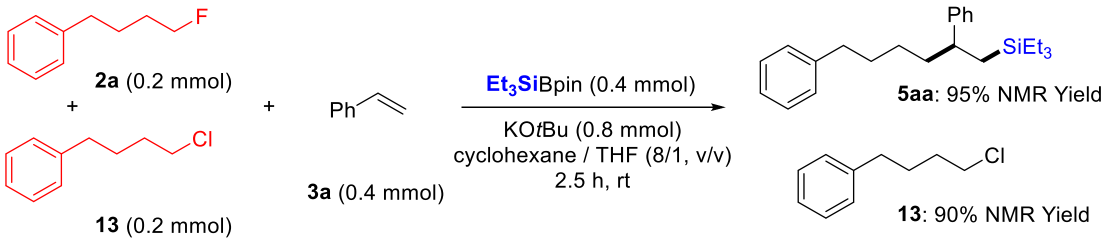

Supplementary Figure 6. Competitive reaction between alkyl fluoride and alkyl chloride with styrene for the carbosilylation

Following the general procedure, charging **2a** (30.4 mg, 0.2 mmol), **13** (33.6 mg, 0.2 mmol), **3a** (46 μL, 0.4 mmol), silyl boronate (0.4 mmol, 2.0 equiv), KO*t*Bu (90 mg, 0.8 mmol, 4.0 equiv), and then cyclohexane (1.3 mL), anhydrous THF (0.17 mL) sequentially. And then move out from glovebox, and stirred at room temperature for 2.5 h. The reaction tube was added *n*-Hexane (5 mL), then subject to filter through a short silica pad, and washed with Et_2O, concentrated under vacuum, followed by 3-Fluoropyridine (8.6 μL, 0.1 mmol) as an internal standard. Then the ^1H NMR analysis and ^{19}F NMR analysis of the crude mixture were conducted to show the details of the competitive reaction.

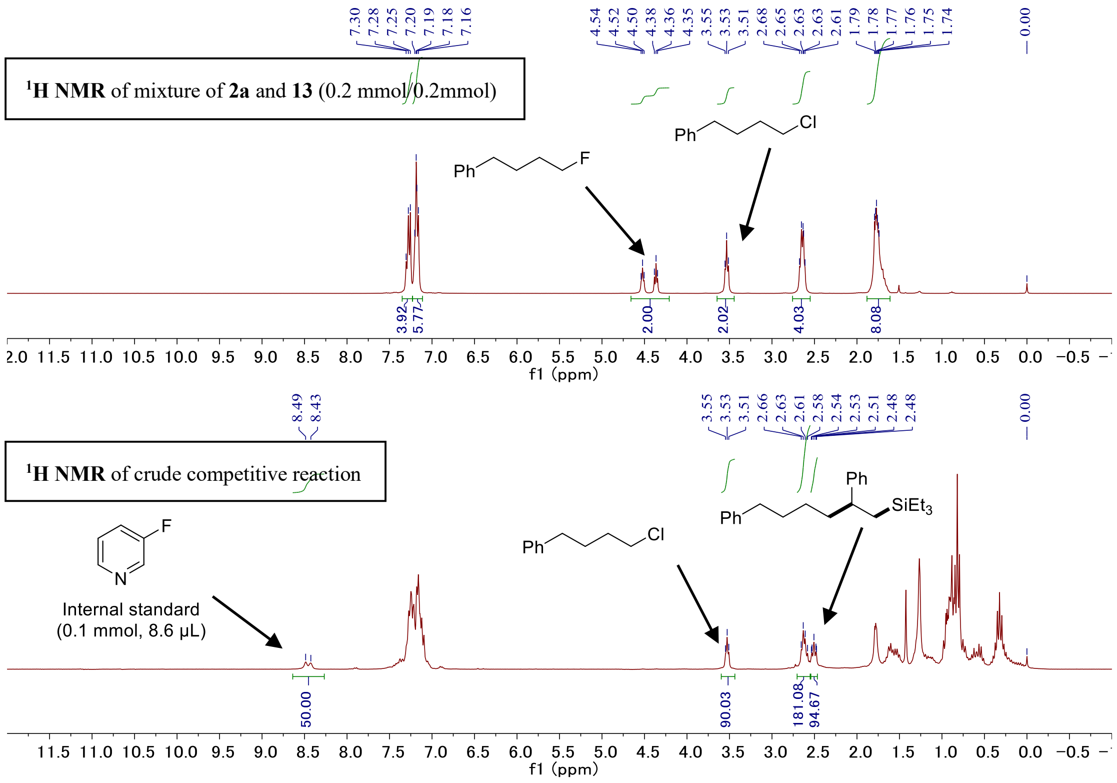

Supplementary Figure 7. Comparison of ^1H NMR analysis of the reaction mixture

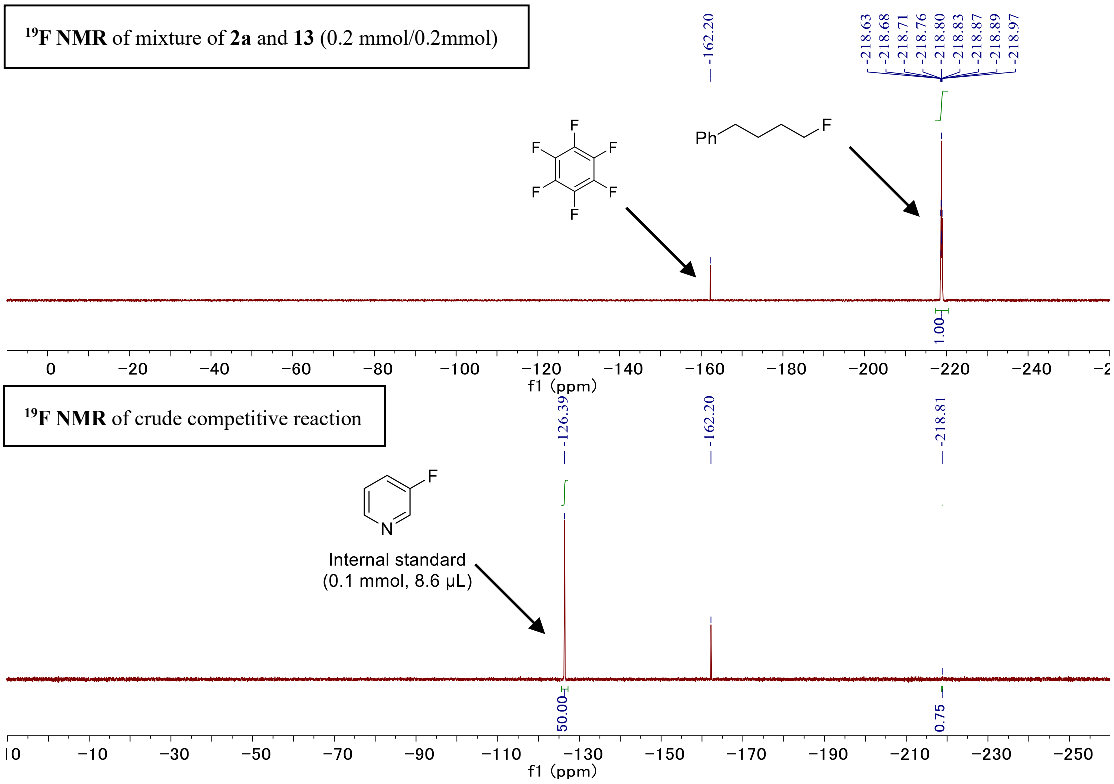

Supplementary Figure 8. Comparison of ^{19}F NMR analysis of the reaction mixture

7. Preliminary Mechanistic Investigations

7.1 Reaction with radical scavenger

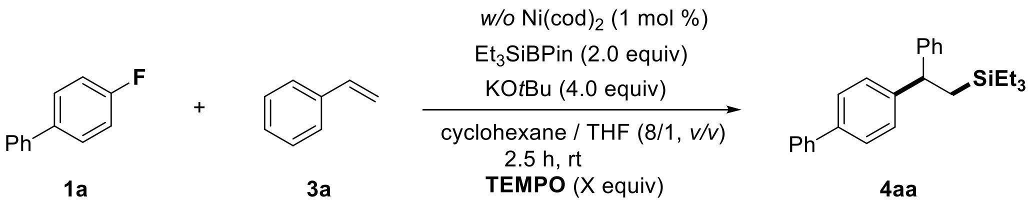

Reaction Conditions	Yield of **4aa**
TEMPO (1.0 equiv)	8%
TEMPO (2.0 equiv))	0%
$Ni(COD)_2$ (1 mol%), TEMPO (1.0 equiv)	12%
$Ni(COD)_2$ (1 mol%), TEMPO (2.0 equiv)	0%

Supplementary Figure 9. Reaction with radical scavenger

Following the general procedure, charging aryl fluoride **1a** (34.4 mg, 0.2 mmol), **3a** (46 µL, 0.4 mmol), silyl boronate (0.4 mmol, 2.0 equiv), with or without $Ni(cod)_2$, KO*t*Bu (90mg, 0.8 mmol, 4.0 equiv), TMEPO and cyclohexane/THF (1.5 mL, 8/1, *v/v*) sequentially. And then move out from glovebox, stirred at room temperature for 2.5 h. The reaction mixture after workup to conduct NMR analysis using 3-fluoropyridine (8.6 µL, 0.1 mmol) as an internal standard to give corresponding yields of **4aa**.

7.2 Radical clock experiments

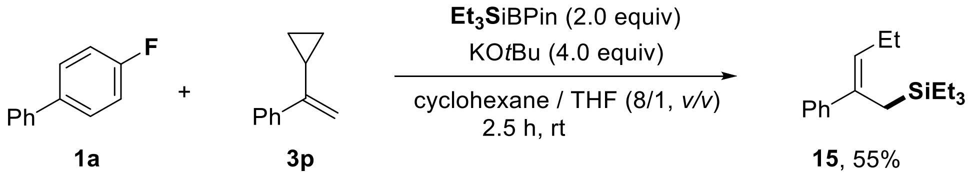

Following the general procedure, charging aryl fluoride **1a** (0.2 mmol), **3p** (57.6 mg, 0.4 mmol), silyl boronate (0.4 mmol, 2.0 equiv), KO*t*Bu (90mg, 0.8 mmol, 4.0 equiv) and cyclohexane/THF (1.5 mL, 8/1, *v/v*) sequentially. And then move out from glovebox, stirred at room temperature for 2.5 h. The reaction mixture after workup to give the residue, which was purified by column chromatography on silica gel to give the corresponding carbosilylation product **15**.

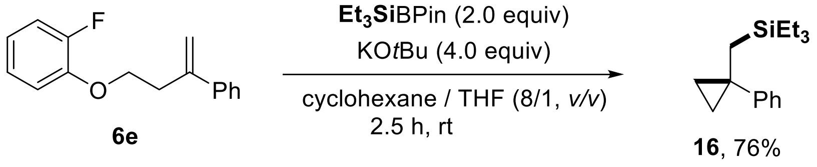

Following the general procedure, charging aryl fluoride with styrene moiety **6e** (0.2 mmol), silyl boronate (0.4 mmol, 2.0 equiv), KO*t*Bu (90mg, 0.8 mmol, 4.0 equiv) and cyclohexane/THF (1.5 mL, 8/1, *v/v*) sequentially. And then move out from glovebox, stirred at room temperature for 2.5 h. The reaction mixture after workup to give the residue, which was purified by column chromatography on silica gel to give the corresponding carbosilylation product **16**.

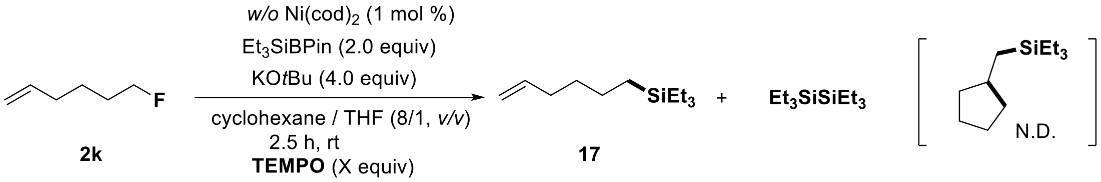

Reaction Conditions	Yield of **17**	Yield of **$Et_3SiSiEt_3$**
TEMPO (0 equiv)	73%	21%
TEMPO (2.0 equiv))	0%	0%
$Ni(COD)_2$ (1 mol%), TEMPO (2.0 equiv)	0%	0%

Supplementary Figure 10. Effect of TEMPO for the reaction of **2k** and silyl boronate

Following the general procedure, charging alkyl fluoride with alkene moiety 6-Fluorohex-1-ene **2k** (0.2 mmol), silyl boronate (0.4 mmol, 2.0 equiv), with or without $Ni(cod)_2$, KO*t*Bu (90mg, 0.8 mmol, 4.0 equiv), TMEPO and cyclohexane/THF (1.5 mL, 8/1, *v/v*) sequentially. And then move out from glovebox, stirred at room temperature for 2.5 h. The reaction mixture after workup to give the residue, which was purified by column chromatography on silica gel to give the corresponding products **17** and **Hexaethyldisilane**.

7.3 The reaction process

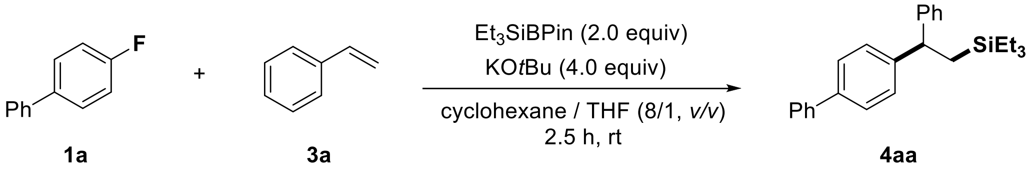

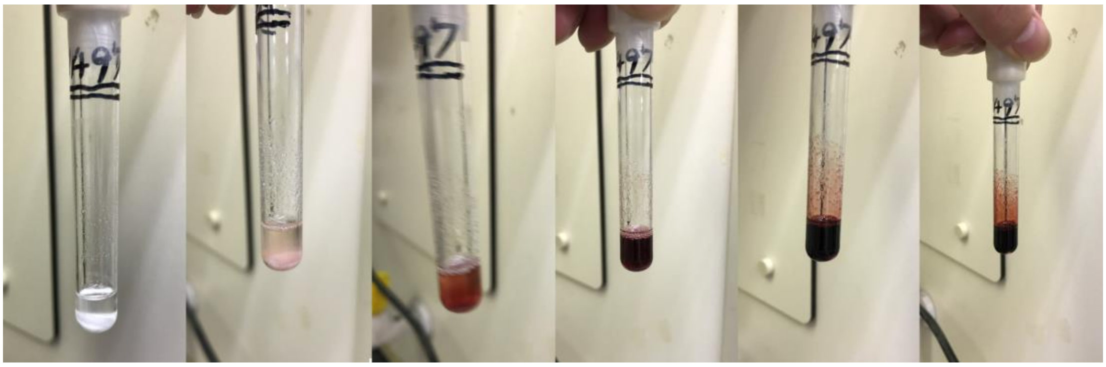

Before inject THF(0.17 mL) 0 s	After inject THF(0.17 mL) 5 s	After inject THF(0.17 mL) 10 s	After inject THF(0.17 mL) 20 s	After inject THF(0.17 mL) 30 s	After inject THF(0.17 mL) 60 s

Supplementary Figure 11. The reaction process

Following the general procedure, charging aryl fluoride **1a** (34.4 mg, 0.2 mmol), **3a** (46 μL, 0.4 mmol), silyl boronate (0.4 mmol, 2.0 equiv), KO*t*Bu (90mg, 0.8 mmol, 4.0 equiv), and cyclohexane (1.3 mL) sequentially. Move out from glovebox, and then inject anhydrous THF (0.17 mL). stirred at room temperature for 2.5 h, and monitored by pictures during this reaction time. The reaction mixture after workup to conduct NMR analysis using 3-fluoropyridine (8.6 μL, 0.1 mmol) as an internal standard to give corresponding yield of **4aa**.

7.4 The NMR spectroscopic studies

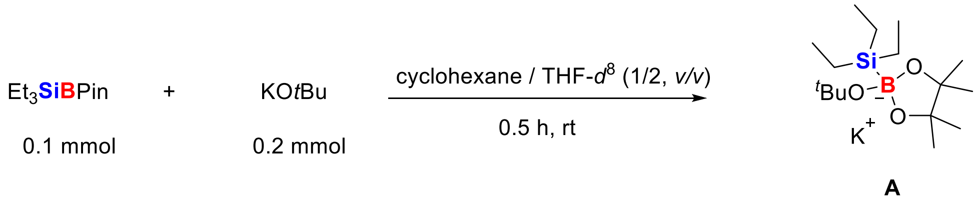

^{11}B NMR (THF-d^8, 225 MHz): δ_B = 3.88 ppm;
^{29}Si NMR (THF-d^8, 139 MHz): δ_{Si} = -9.23 ppm

Following the general procedure, to a dry NMR tube was sequentially added a silylboranate (24.2 mg, 0.1 mmol), KO*t*Bu (23 mg, 0.2 mmol) and cyclohexane/THF-d^8 (1/2, v/v, 0.6 ml) in a glovebox filled with argon gas. The NMR tube was sealed and removed from the glovebox, and stirred at room temperature. After 30 min, the ^{11}B NMR analysis and ^{29}Si NMR analysis of the crude mixture conformed that the adduct **A** was present in the reaction mixture as shown in the following charts[10, 38, 39].

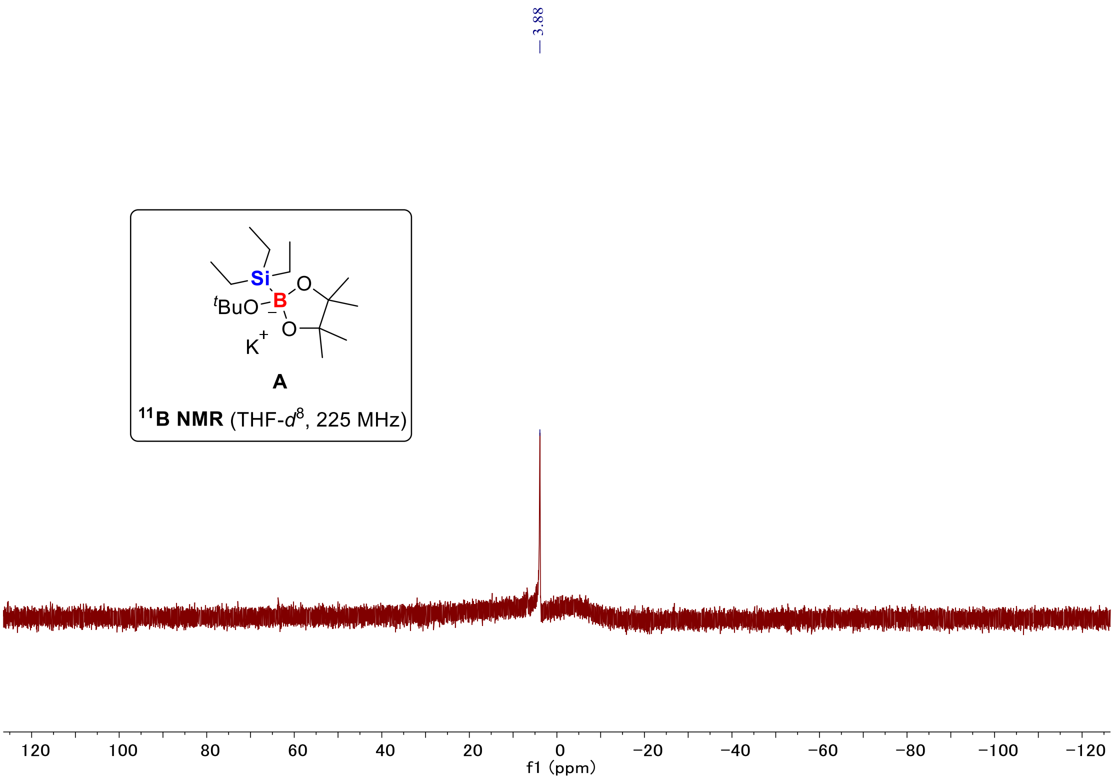

Supplementary Figure 12. ^{11}B NMR observation of KO*t*Bu and Et_3SiBpin adduct **A**

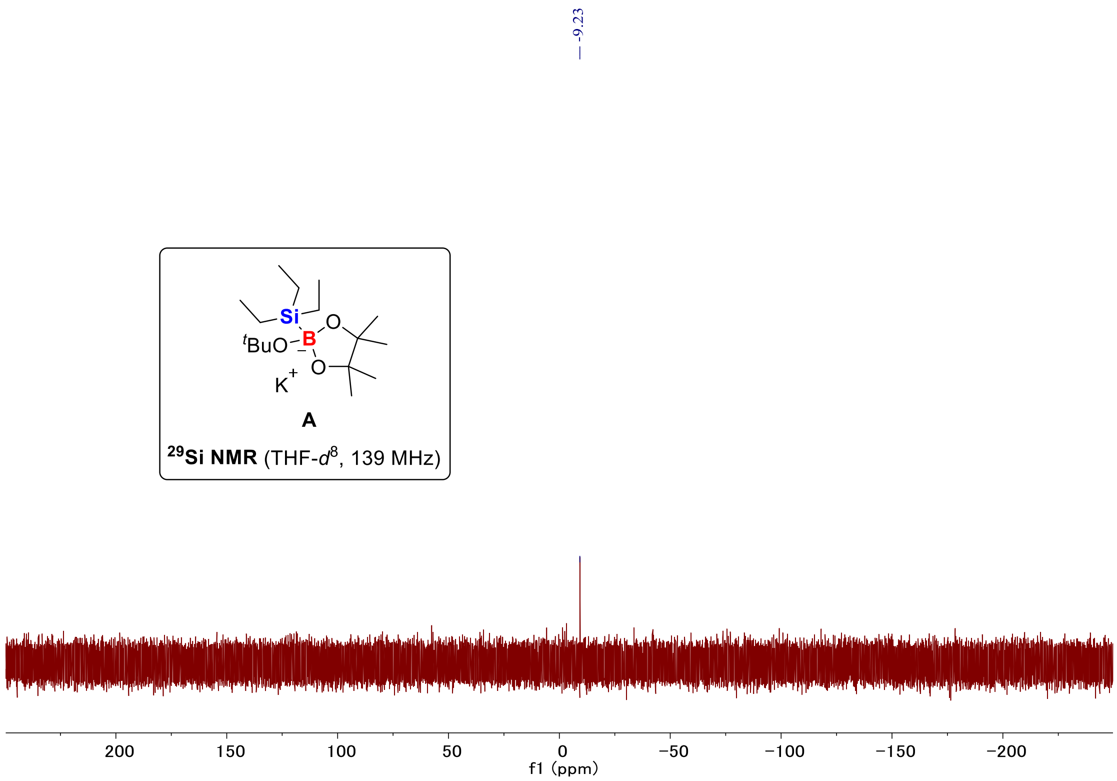

Supplementary Figure 13. ^{29}Si NMR observation of KO*t*Bu and Et_3SiBpin adduct **A**

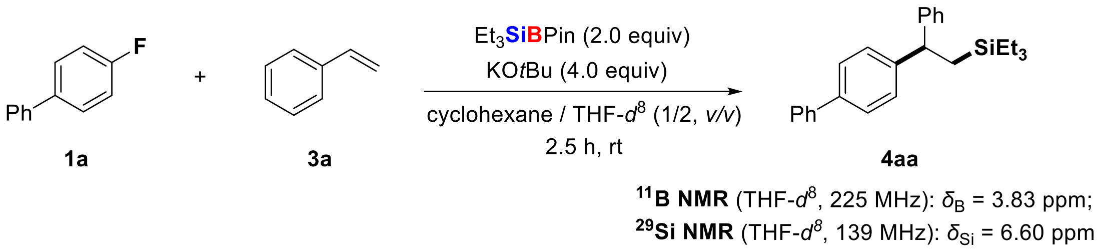

Following the general procedure, to a dry NMR tube was sequentially charging aryl fluoride **1a** (17.2 mg, 0.1 mmol), **3a** (23 μL, 0.2 mmol), silyl boronate (48.4 mg, 0.2 mmol), KO*t*Bu (45 mg, 0.4 mmol), and cyclohexane/THF-*d*^{8} (1/2, v/v, 0.6 ml) in a glovebox filled with argon gas. The NMR tube was sealed and removed from the glovebox, and stirred at room temperature. After 2.5 h, the ^{11}B NMR analysis and ^{29}Si NMR analysis of the crude mixture were conducted to monitor the reaction mixture, and shown in the following charts[40, 41].

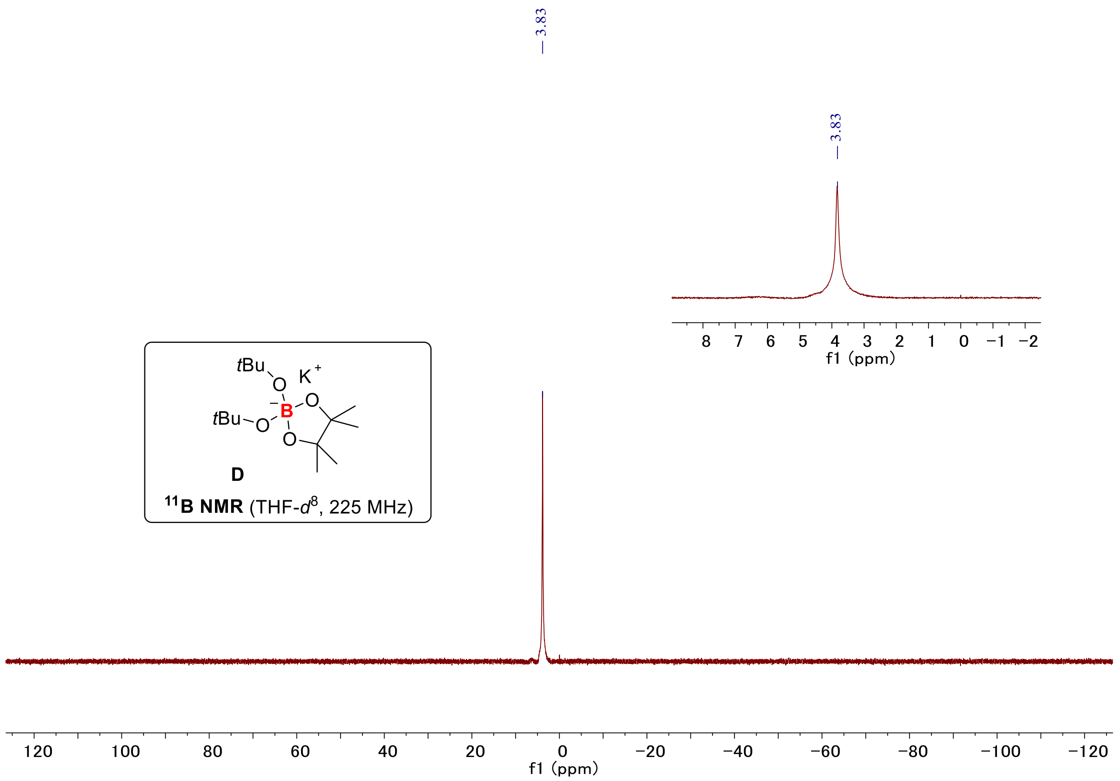

Supplementary Figure 14. ^{11}B NMR observation of adduct **D**.

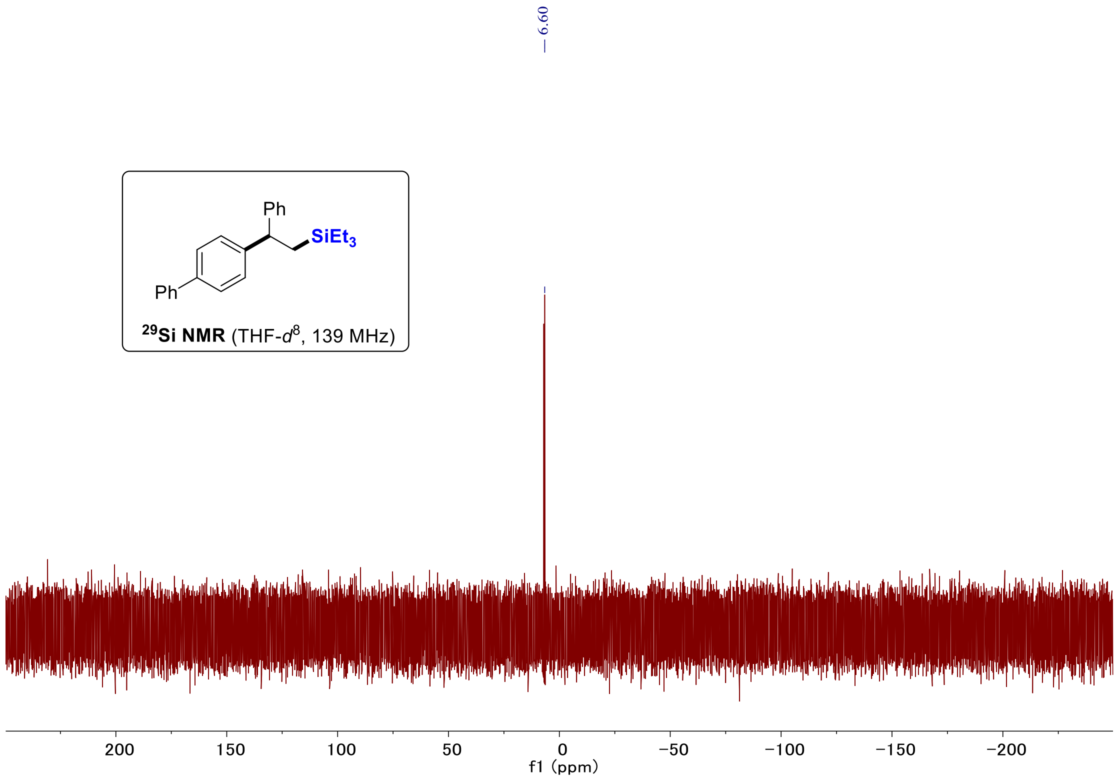

Supplementary Figure 15. ^{29}Si NMR observation of reaction crude

8. Characterization Data of Products

(2-(Biphenyl-4-yl)-2-phenylethyl)triethylsilane (4aa)

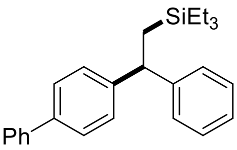

Compound **4aa** was obtained as a colorless oil (without Nickel catalysis: 67.5 mg, Yield: 91%; with Nickel catalysis: 71.5 mg, Yield: 96%). **^{1}H NMR** (500 MHz, $CDCl_3$) δ 7.58 – 7.52 (m, 2H), 7.51 – 7.45 (m, 2H), 7.40 (t, *J* = 7.7 Hz, 2H), 7.36 (d, *J* = 8.2 Hz, 2H), 7.36 – 7.30 (m, 2H), 7.33 – 7.20 (m, 3H), 7.15 (t, *J* = 7.3 Hz, 1H), 4.11 (t, *J* = 7.9 Hz, 1H), 1.49 – 1.40 (m, 2H), 0.84 (t, *J* = 7.9 Hz, 9H), 0.35 (q, *J* = 7.9 Hz, 6H). **^{13}C NMR** (126 MHz, $CDCl_3$) δ 147.3, 146.7, 141.2, 139.0, 128.8, 128.5, 1278.0, 127.7, 127.2, 127.1, 127.1, 126.2, 47.0, 19.2, 7.5, 3.6. **IR (KBr)**: 3060, 3028, 2950, 2908, 2875, 1600, 1487, 1452, 1413, 1008, 729, 696 cm^{-1}. **HRMS (EI)** [$C_{26}H_{32}Si$] $[M]^+$ calculated: 372.2273, found: 372.2280.

(2-(Biphenyl-3-yl)-2-phenylethyl)triethylsilane (4ba)

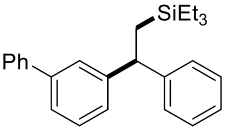

Compound **4ba** was obtained as a colorless oil (without Nickel catalysis: 64.7 mg, Yield: 87%; with Nickel catalysis: 67.7 mg, Yield: 91%). **^{1}H NMR** (500 MHz, $CDCl_3$) δ 7.64 – 7.59 (m, 2H), 7.57 (s, 1H), 7.47 (t, *J* = 7.6 Hz, 2H), 7.44 – 7.28 (m, 8H), 7.19 (t, *J* = 7.3 Hz, 1H), 4.19 (t, *J* = 7.9 Hz, 1H), 1.52 (d, *J* = 8.0 Hz, 2H), 0.89 (t, *J* = 8.0 Hz, 9H), 0.41 (q, *J* = 7.9 Hz, 6H). **^{13}C NMR** (126 MHz, $CDCl_3$) δ 148.0, 147.3, 141.6, 141.4, 128.9, 128.8, 128.5, 127.6, 127.4, 127.3, 126.6, 126.5, 126.2, 125.1, 47.4, 19.2, 7.5, 3.6. **IR (KBr):** 3060, 3027, 2952, 2908, 2873, 1598, 1492, 1477, 1417, 1173, 1006, 975, 770, 741, 698 cm^{-1}. **HRMS (EI)** [$C_{26}H_{32}Si$] $[M]^+$ calculated: 372.2273, found: 372.2289.

(2-(Biphenyl-2-yl)-2-phenylethyl)triethylsilane (4ca)

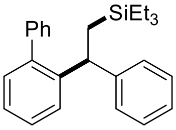

Compound **4ca** was obtained as a colorless oil (without Nickel catalysis: 30.9 mg, Yield: 42%; with Nickel catalysis: 43.2 mg, Yield: 58%). **^{1}H NMR** (300 MHz, $CDCl_3$) δ 7.57 (d, *J* = 7.4 Hz, 1H), 7.40 – 7.27 (m, 4H), 7.25 – 7.11 (m, 6H), 7.14 – 6.97 (m, 3H), 4.41 – 4.15 (m, 1H), 1.49 – 1.17 (m, 2H), 0.71 (t, *J* = 7.9 Hz, 9H), 0.18 (q, *J* = 7.7 Hz, 6H). **^{13}C NMR** (75 MHz, $CDCl_3$) δ 147.0, 144.7, 142.0, 141.5, 130.1, 129.7, 128.2, 128.0, 127.9, 127.7, 127.3, 127.0, 125.9, 125.7, 42.3, 21.0, 7.4, 3.5. **IR (KBr):** 3060, 3025, 2952, 2874, 1599, 1493, 1475, 1415, 1176, 1130, 1010, 909, 781, 737, 701 cm^{-1}. **HRMS (EI)** [$C_{26}H_{32}Si$] (M^+) calculated: 372.2273, found: 372.2260.

Triethyl(1-(naphthalen-2-yl)-2-phenylethyl)silane (4da)

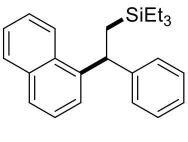

Compound **4da** was obtained as a colorless oil (without Nickel catalysis: 59.4 mg, Yield: 87%; with Nickel catalysis: 54 mg, Yield: 78%). **^{1}H NMR** (500 MHz, $CDCl_3$) δ 8.26 (d, *J* = 8.4 Hz, 1H), 7.88 (d, *J* = 8.0 Hz, 1H), 7.77 (d, *J* = 8.2 Hz, 1H), 7.68 (d, *J* = 7.2 Hz, 1H), 7.52 (t, *J* = 7.6 Hz, 2H), 7.48 (t, *J* = 7.2 Hz, 1H), 7.42 (d, *J* = 7.6 Hz, 2H), 7.29 (t, *J* = 7.6 Hz, 2H), 7.18 (t, *J* = 7.3 Hz,1H), 5.00 (t, *J* = 7.8 Hz, 1H), 1.69 – 1.56 (m, 2H), 0.90 (t, *J* = 8.0 Hz, 9H), 0.43 (q, *J* = 7.9 Hz, 6H). **^{13}C NMR** (126 MHz, $CDCl_3$) δ 147.2, 142.6, 134.2, 131.6, 129.0, 128.4, 127.9, 127.0, 126.1, 126.0, 125.5, 125.4, 124.5, 123.7, 41.9, 20.2, 7.5, 3.8. **IR (KBr):** 3052, 2952, 2874, 1598, 1453, 1416, 1177, 1015, 830, 787, 740, 702 cm^{-1}. **HRMS (EI)** [$C_{24}H_{30}Si$] $[M]^+$ calculated: 346.2117, found: 346.2107.

Triethyl(2-(4-(naphthalen-1-yl)phenyl)-2-phenylethyl)silane (4ea)

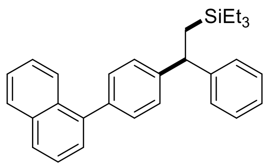

Compound **4ea** was obtained as a colorless oil (without Nickel catalysis: 75.9 mg, Yield: 90%; with Nickel catalysis: 78.5 mg, Yield: 93%). **^{1}H NMR** (500 MHz, $CDCl_3$) δ 7.92 (dd, *J* = 8.4, 3.9 Hz, 2H), 7.87 (d, *J* = 8.2 Hz, 1H), 7.57 – 7.47 (m, 2H), 7.51 – 7.39 (m, 8H), 7.35 (t, *J* = 7.6 Hz, 2H), 7.23 (t, *J* = 7.3 Hz, 1H), 4.21 (t, *J* = 7.9 Hz, 1H), 1.54 (d, *J* = 8.0 Hz, 2H), 0.92 (t, *J* = 7.9 Hz, 9H), 0.44 (q, *J* = 8.0 Hz, 6H). **^{13}C NMR** (126 MHz, $CDCl_3$) δ 147.3, 146.6, 140.3, 138.5, 133.9, 131.8, 130.2, 128.5, 128.3, 127.7, 127.6, 127.5, 127.0, 126.3, 126.0, 125.8, 125.5, 47.1, 19.4, 7.6, 3.7. **IR (KBr):** 3057, 2952, 2909, 2874, 1601, 1493, 1396, 1173, 1018, 800, 778, 7398, 703 cm^{-1}. **HRMS (EI)** [$C_{30}H_{34}Si$] $[M]^+$ calculated: 422.2430, found: 422.2414.

(2,2-Diphenylethyl)triethylsilane (4fa)[42]

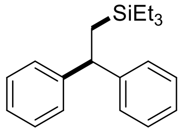

Compound **4fa** was obtained as a colorless oil (without Nickel catalysis: 74.6 mg, Yield: 63%; with Nickel catalysis: 82.9 mg, Yield: 70%). **^{1}H NMR** (500 MHz, $CDCl_3$) δ 7.29 (d, *J* = 7.3 Hz, 4H), 7.24 (t, *J* = 7.6 Hz, 4H), 7.13 (t, *J* = 7.2 Hz, 2H), 4.06 (t, *J* = 7.9 Hz, 1H), 1.41 (d, *J* = 7.9 Hz, 2H), 0.82 (t, *J* = 8.0 Hz, 9H), 0.33 (q, *J* = 8.0 Hz, 6H). **IR (KBr):** 3026, 2952, 2909, 2874, 1599, 1493, 1451, 1415, 1236, 1173, 1014, 853, 827, 778, 741, 702 cm^{-1}. **HRMS (EI)** [$C_{20}H_{28}Si$] $[M]^+$ calculated: 296.1960, found: 296.1947.

Triethyl(2-phenyl-2-(p-tolyl)ethyl)silane (4ga)

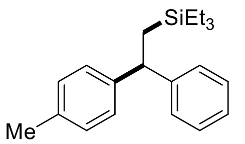

Compound **4ga** was obtained as a colorless oil (without Nickel catalysis: 105.1 mg, Yield: 85%; with Nickel catalysis: 111.8 mg, Yield: 90%). **^{1}H NMR** (500 MHz, $CDCl_3$) δ 7.46 (d, *J* = 7.1 Hz, 2H), 7.41 (t, *J* = 7.6 Hz, 2H), 7.36 (d, *J* = 7.8 Hz, 2H), 7.30 (t, *J* = 7.2 Hz, 1H), 7.23 (d, *J* = 7.9 Hz, 2H), 4.21 (t, *J* = 7.9 Hz, 1H), 2.46 (s, 3H), 1.57 (d, *J* = 7.9 Hz, 2H), 1.01 (t, *J* = 7.9 Hz, 9H), 0.51 (q, *J* = 8.0 Hz, 6H). **^{13}C NMR** (126 MHz, $CDCl_3$) δ 147.7, 144.5, 135.5, 129.1, 128.4, 127.6, 127.4, 126.0, 46.8, 21.1, 19.2, 7.5, 3.6. **IR (KBr):** 3024, 2952, 2910, 2874, 1600, 1511, 1491, 1453, 1416, 1237, 1173, 1016, 804, 740, 699 cm^{-1}. **HRMS (EI)** [$C_{21}H_{30}Si$] $[M]^+$ calculated: 310.2117, found: 310.2130.

Triethyl(2-(4'-methoxy-biphenyl-4-yl)-2-phenylethyl)silane (4ha)

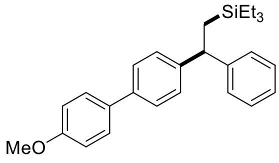

Compound **4ha** was obtained as a white solid, m.p. = 62.0 – 63.2 °C (without Nickel catalysis: 61.8 mg, Yield: 75%; with Nickel catalysis: 73.2 mg, Yield: 91%). ^{1}**H NMR** (500 MHz, $CDCl_3$) δ 7.51 (d, *J* = 8.7 Hz, 2H), 7.47 (d, *J* = 7.9 Hz, 2H), 7.40 – 7.33 (m, 4H), 7.32 – 7.27 (m, 2H), 7.22 – 7.14 (m, 1H), 6.97 (d, *J* = 8.6 Hz, 2H), 4.13 (t, *J* = 7.9 Hz, 1H), 3.85 (s, 3H), 1.47 (dd, *J* = 7.8, 2.2 Hz, 2H), 0.87 (t, *J* = 7.9 Hz, 9H), 0.39 (q, *J* = 7.9 Hz, 6H). **^{13}C NMR** (126 MHz, $CDCl_3$) δ 159.1, 147.4, 146.0, 138.6, 133.7, 128.5, 128.1, 127.9, 127.6, 126.7, 126.2, 114.2, 55.4, 46.9, 19.2, 7.5, 3.6. **IR (KBr):** 3057, 3024, 2953, 2909, 2874, 1609, 1497, 1465, 1247, 1178, 1042, 1002, 832, 740, 703 cm^{-1}. **HRMS (EI)** [$C_{27}H_{34}OSi$] $[M]^+$ calculated: 402.2379, found: 402.2366.

(2-(4'-(Benzyloxy)-biphenyl-4-yl)-2-phenylethyl)triethylsilane (4ia)

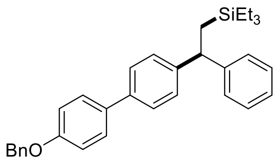

Compound **4ia** was obtained as a white solid, m.p. = 112.3 – 113.9 °C (without Nickel catalysis: 70.1 mg, Yield: 73%; with Nickel catalysis: 81.3 mg, Yield: 85%). **^{1}H NMR** (500 MHz, $CDCl_3$) δ 7.50 (d, *J* = 8.7 Hz, 2H), 7.46 (dd, *J* = 7.6, 4.4 Hz, 4H), 7.41 (t, *J* = 7.4 Hz, 2H), 7.38 – 7.33 (m, 5H), 7.28 (t, *J* = 7.7 Hz, 2H), 7.17 (t, *J* = 7.3 Hz, 1H), 7.04 (d, *J* = 8.7 Hz, 2H), 5.11 (s, 2H), 4.11 (t, *J* = 7.9 Hz, 1H), 1.46 (dd, *J* = 7.9, 2.3 Hz, 2H), 0.85 (t, *J* = 7.9 Hz, 9H), 0.37 (q, *J* = 7.9 Hz, 6H). **^{13}C NMR** (126 MHz, $CDCl_3$) δ 158.3, 147.3, 146.1, 138.5, 137.1, 133.9, 128.7, 128.5, 128.12, 128.11, 127.9, 127.64, 127.62, 126.8, 126.2, 115.2, 70.2, 46.9, 19.1, 7.5, 3.6. **IR (KBr):** 3060, 3027, 2952, 2908, 2873, 1607, 1497, 1454, 1242, 1093, 1001, 801, 738, 698 cm^{-1}. **HRMS (EI)** [$C_{33}H_{38}OSi$] [M]$^+$ calculated: 478.2692, found: 478.2672.

(2-(4-(Benzo[*d*][1,3]dioxol-5-yl)phenyl)-2-phenylethyl)triethylsilane (4ja)

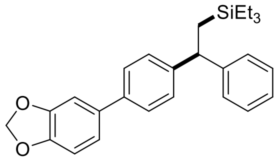

Compound **4ja** was obtained as a colorless oil (without Nickel catalysis: 57.5 mg, Yield: 69%; with Nickel catalysis: 64.9 mg, Yield: 78%). **^{1}H NMR** (500 MHz, $CDCl_3$) δ 7.46 – 7.41 (m, 2H), 7.38 – 7.34 (m, 4H), 7.29 (t, *J* = 7.7 Hz, 2H), 7.22 – 7.15 (m, 1H), 7.08 – 7.02 (m, 2H), 6.88 (d, *J* = 8.0 Hz, 1H), 5.99 (s, 2H), 4.13 (t, *J* = 7.9 Hz, 1H), 1.54 – 1.38 (m, 2H), 0.87 (t, *J* = 7.9 Hz, 9H), 0.38 (q, *J* = 7.5 Hz, 6H) **^{13}C NMR** (126 MHz, $CDCl_3$) δ 148.1, 147.2, 146.9, 146.3, 138.7, 135.5, 128.5, 127.9, 127.6, 126.9, 126.2, 120.5, 108.6, 107.6, 101.2, 46.9, 19.1, 7.5, 3.6. **IR (KBr):** 3057, 3025, 2954, 2909, 2876, 1600, 1502, 1482, 1441, 1414, 1225, 1124, 1041, 1010, 804, 737, 704 cm^{-1}. **HRMS (EI)** [$C_{27}H_{32}O_2Si$] [M]$^+$ calculated: 416.2172, found: 416.2163.

Triethyl(2-phenyl-2-(3'-(trifluoromethyl)-biphenyl-4-yl)ethyl)silane (4ka)

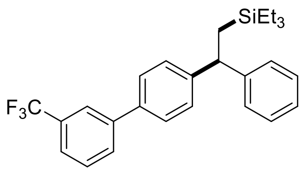

Compound **4ka** was obtained as a colorless oil (without Nickel catalysis: 44.1 mg, Yield: 50%; with Nickel catalysis: 42.2 mg, Yield: 48%). **^{1}H NMR** (500 MHz, $CDCl_3$) δ 7.71 (s, 1H), 7.64 (d, *J* = 7.7 Hz, 1H), 7.48 (d, *J* = 7.8 Hz, 1H), 7.44 (d, *J* = 7.7 Hz, 1H), 7.41 (d, *J* = 8.0 Hz, 1H), 7.32 (d, *J* = 7.9 Hz, 2H), 7.25 (d, *J* = 7.0 Hz, 2H), 7.21 (d, *J* = 7.5 Hz, 2H), 7.18 (d, *J* = 4.5 Hz, 1H), 7.09 (t, *J* = 7.1 Hz, 1H), 4.05 (t, *J* = 7.9 Hz, 1H), 1.44 – 1.31 (m, 2H), 0.77 (t, *J* = 7.9 Hz, 9H), 0.29 (q, *J* = 7.9 Hz, 6H). **^{13}C NMR** (126 MHz, $CDCl_3$) δ 147.6, 147.1, 141.9, 137.5, 131.2 (q, *J* = 32.0 Hz), 130.4, 129.3, 128.6, 128.2, 127.6, 127.3, 126.3, 124.3(q, *J* = 272.4 Hz), 123.8(dq, *J* = 7.7, 3.8 Hz), 47.0, 19.1, 7.5, 3.6, 1.2. **^{19}F NMR** (282 MHz, $CDCl_3$) δ -63.11(s, 3F). **IR (KBr):** 3057, 3029, 2955, 2910, 2875, 1600, 1486, 1443, 1414, 1335, 1168, 1128, 1097, 1075, 1016, 799, 741, 702 cm^{-1}. **HRMS (EI)** [$C_{27}H_{31}F_3Si$] [M]$^+$ calculated: 440.2147, found: 440.2148.

Triethyl(2-phenyl-2-(4'-(trifluoromethyl)-biphenyl-4-yl)ethyl)silane (4la)

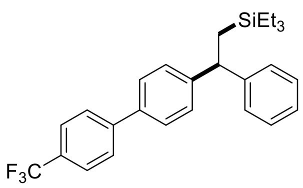

Compound **4la** was obtained as a colorless oil (without Nickel catalysis: 46.9 mg, Yield: 53%; with Nickel catalysis: 53.7 mg, Yield: 61%). **^{1}H NMR** (500 MHz, $CDCl_3$) δ 7.70 – 7.63 (m, 4H), 7.51 (d, *J* = 8.3 Hz, 2H), 7.42 (d, *J* = 8.2 Hz, 2H), 7.35 (dd, *J* = 8.2, 1.1 Hz, 2H), 7.32 – 7.25 (m, 2H), 7.19 (d, *J* = 7.3 Hz, 1H), 4.14 (t, *J* = 7.9 Hz, 1H), 1.47 (t, *J* = 8.0 Hz, 2H), 0.86 (t, *J* = 7.9 Hz, 9H), 0.38 (t, *J* = 8.0 Hz, 6H). **^{13}C NMR** (126 MHz, $CDCl_3$) δ 147.8, 147.0, 144.6, 137.5, 129.1(q, *J* = 32.4 Hz), 128.6, 128.2, 127.6, 127.3, 127.3, 126.3, 125.8(q, *J* = 3.6 Hz), 125.5(q, *J* = 272.2 Hz), 47.0, 19.1, 7.5, 3.6. **^{19}F NMR** (282 MHz, $CDCl_3$) δ -62.36 (s, 3F). **IR (KBr):** 3025, 2952, 2910, 2875, 1617, 1491, 1451, 1326, 1167, 1125, 1071, 1007, 836, 771, 742, 728, 699 cm^{-1}. **HRMS (EI)** [$C_{27}H_{31}F_3Si$] $[M]^+$ calculated: 440.2147, found: 440.2148.

(2-(4'-Chloro-biphenyl-4-yl)-2-phenylethyl)triethylsilane (4ma)

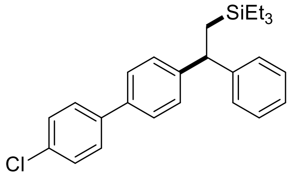

Compound **4ma** was obtained as a colorless oil (without Nickel catalysis: 56.6 mg, Yield: 70%; with Nickel catalysis: 63.1 mg, Yield: 78%). **^{1}H NMR** (500 MHz, $CDCl_3$) δ 7.52 – 7.44 (m, 4H), 7.42 – 7.36 (m, 4H), 7.38 – 7.32 (m, 2H), 7.31 – 7.27 (m, 2H), 7.21 – 7.16 (m, 1H), 4.13 (t, *J* = 7.9 Hz, 1H), 1.52 – 1.39 (m, 2H), 0.86 (t, *J* = 7.9 Hz, 9H), 0.37 (q, *J* = 7.6 Hz, 6H). **^{13}C NMR** (126 MHz, $CDCl_3$) δ 147.1, 139.5, 137.7, 133.2, 128.9, 128.6, 128.3, 128.1, 127.6, 127.0, 126.3, 46.9, 19.1, 7.5, 3.6. **IR (KBr):** 3054, 3025, 2953, 2909, 2874, 1600, 1484, 1417, 1174, 1093, 1005, 809, 742, 704 cm^{-1}. **HRMS (EI)** [$C_{26}H_{31}ClSi$] $[M]^+$calculated: 406.1884, found: 406.1887.

1-Methyl-6-(1-phenyl-2-(triethylsilyl)ethyl)-1*H*-indole (4na)

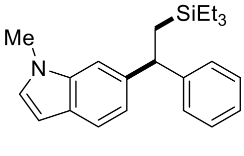

Compound **4na** was obtained as a colorless oil (39.8 mg, Yield: 57%). **^{1}H NMR** (300 MHz, $CDCl_3$) δ 7.50 (d, *J* = 8.2 Hz, 1H), 7.40 – 7.29 (m, 2H), 7.30 – 7.18 (m, 3H), 7.18 – 7.06 (m, 1H), 7.12 – 7.02 (m, 1H), 6.98 (d, *J* = 3.0 Hz, 1H), 6.40 (d, *J* = 3.1 Hz, 1H), 4.22 (t, *J* = 7.9 Hz, 1H), 3.77 (s, 3H), 1.50 (d, *J* = 7.8 Hz, 2H), 0.84 (t, *J* = 7.9 Hz, 9H), 0.35 (q, *J* = 7.9 Hz, 6H). **^{13}C NMR** (75 MHz, $CDCl_3$) δ 148.3, 141.2, 137.0, 128.7, 128.4, 127.6, 126.8, 125.9, 120.7, 119.9, 107.6, 100.7, 47.5, 32.9, 19.4, 7.5, 3.6. **IR (KBr):** 3056, 3025, 2952, 2908, 2874, 1620, 1544, 1409, 1328, 1125, 1067, 980, 909, 735, 702 cm^{-1}. **HRMS (EI)** [$C_{23}H_{31}NSi$] (M^+) calculated: 349.2226, found: 349.2217.

1-(4-(1-Phenyl-2-(triethylsilyl)ethyl)phenyl)-1*H*-pyrrole (4oa)

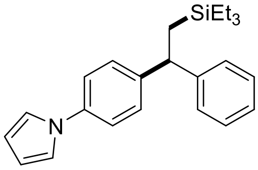

Compound **4oa** was obtained as a colorless oil (23 mg, Yield: 32%). **^{1}H NMR** (500 MHz, $CDCl_3$) δ 7.37 – 7.22 (m, 8H), 7.19 – 7.13 (m, 1H), 7.03 (s, 2H), 6.31 (s, 2H), 4.09 (t, *J* = 7.9 Hz, 1H), 1.48 – 1.34 (m, 2H), 0.83 (t, *J* = 7.9 Hz, 9H), 0.35 (q, *J* = 7.9 Hz, 6H). **^{13}C NMR** (126 MHz, $CDCl_3$) δ 147.2, 145.0, 138.9, 128.6, 128.6, 127.6, 126.3, 120.6, 119.4, 110.2, 46.7, 19.2, 7.5, 3.6. **IR (KBr):** 3056, 3025, 2952, 2909, 2874, 1611, 1520, 1482, 1415, 1330, 1071, 1018, 841, 738, 701 cm^{-1}. **HRMS (EI)** [$C_{24}H_{31}NSi$] $[M]^+$ calculated: 361.2226, found: 361.2220.

4-(4-(1-Phenyl-2-(triethylsilyl)ethyl)phenyl)pyridine (4pa)

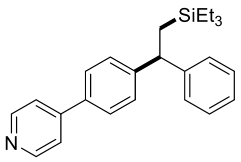

Compound **4pa** was obtained as a colorless oil (59.7 mg, Yield: 80%). **^{1}H NMR** (500 MHz, $CDCl_3$) δ 8.61 (d, *J* = 5.6 Hz, 2H), 7.54 (d, *J* = 8.0 Hz, 2H), 7.46 (d, *J* = 6.2 Hz, 2H), 7.42 (d, *J* = 7.9 Hz, 2H), 7.32 (d, *J* = 7.9 Hz, 2H), 7.27 (t, *J* = 7.6 Hz, 2H), 7.16 (t, *J* = 7.4 Hz, 1H), 4.13 (t, *J* = 7.9 Hz, 1H), 1.52 – 1.38 (m, 2H), 0.84 (t, *J* = 8.0 Hz, 9H), 0.36 (q, *J* = 8.1 Hz, 6H). **^{13}C NMR** (126 MHz, $CDCl_3$) δ 150.3, 148.8, 148.2, 146.8, 135.8, 128.6, 128.3, 127.6, 127.1, 126.4, 121.5, 47.0, 19.0, 7.5, 3.6. **IR (KBr):** 3027, 2952, 2909, 2874, 1596, 1542, 1489, 1453, 1404, 1236, 1173, 1014, 806, 771, 741, 701 cm^{-1}. **HRMS (EI)** [$C_{25}H_{31}NSi$] $[M]^+$ calculated: 373.2226, found: 373.2236.

2-Phenyl-5-(1-phenyl-2-(triethylsilyl)ethyl)pyridine (4qa)

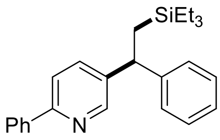

Compound **4qa** was obtained as a colorless oil (50.8 mg, Yield: 68%). **^{1}H NMR** (500 MHz, $CDCl_3$) δ 8.67 (d, *J* = 1.3 Hz, 1H), 7.96 (dt, *J* = 8.3, 1.8 Hz, 2H), 7.70 – 7.59 (m, 2H), 7.48 – 7.43 (m, 2H), 7.42 – 7.35 (m, 1H), 7.35 – 7.31 (m, 2H), 7.29 (dd, *J* = 8.6, 6.8 Hz, 2H), 7.23 – 7.15 (m, 1H), 4.14 (t, *J* = 7.9 Hz, 1H), 1.60 – 1.35 (m, 2H), 0.86 (t, *J* = 7.9 Hz, 9H), 0.38 (q, *J* = 7.9 Hz, 6H). **^{13}C NMR** (126 MHz, $CDCl_3$) δ 155.4, 149.1, 146.3, 141.2, 139.3, 135.7, 128.8, 128.7, 127.6, 126.8, 126.6, 120.3, 44.6, 19.0, 7.5, 3.6. **IR (KBr):** 3055, 3026, 2953, 2909, 2874, 1595, 1474, 1416, 1376, 1237, 1176, 1006, 910, 826, 737, 701 cm^{-1}. **HRMS (EI)** [$C_{25}H_{31}NSi$] $[M]^+$ calculated: 373.2226, found: 373.2220.

5-Phenyl-2-(1-phenyl-2-(triethylsilyl)ethyl)pyridine (4ra)

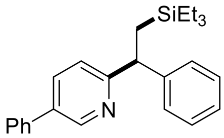

Compound **4ra** was obtained as a colorless oil (55.2 mg, Yield: 74%).**^{1}H NMR** (500 MHz, $CDCl_3$) δ 8.78 (d, *J* = 2.2 Hz, 1H), 7.80 – 7.71(m, 1H), 7.59 – 7.52 (m, 2H), 7.49 – 7.42 (m, 4H), 7.41 – 7.35 (m, 1H), 7.33 – 7.27(m, 3H), 7.23 – 7.15 (m, 1H), 4.28 (t, *J* = 7.8 Hz, 1H), 1.77 – 1.42 (m, 2H), 0.85 (t, *J* = 7.9 Hz, 9H), 0.37 (q, *J* = 7.9 Hz, 6H). **^{13}C NMR** (126 MHz, $CDCl_3$) δ 164.6, 147.6, 146.1, 138.1, 134.9, 134.2, 129.1, 128.6, 127.9, 127.88, 127.1, 126.5, 122.1, 49.3, 18.4, 7.5, 3.6. **IR (KBr):** 3060, 3025, 2952, 2905, 2873, 1594, 1473, 1413, 1005, 745, 698 cm^{-1}. **HRMS (EI)** [$C_{25}H_{31}NSi$] $[M]^+$ calculated: 373.2226, found: 373.2224.

2-Phenyl-4-(1-phenyl-2-(triethylsilyl)ethyl)pyridine (4sa)

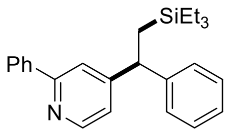

Compound **4sa** was obtained as a white solid, m.p. = 92.6 – 93.8 ^{o}C (65.7 mg, Yield: 88%). **^{1}H NMR** (500 MHz, $CDCl_3$) δ 8.57 (d, *J* = 5.1 Hz, 1H), 8.08 – 7.88 (m, 2H), 7.66 (s, 1H), 7.48 (t, *J* = 7.6 Hz, 2H), 7.45 – 7.37 (m, 1H), 7.36 – 7.27 (m, 4H), 7.24 – 7.16 (m, 2H), 4.12 (t, *J* = 7.9 Hz, 1H), 1.47 (qd, *J* = 14.7, 7.9 Hz, 2H), 0.87 (t, *J* = 7.9 Hz, 9H), 0.39 (q, *J* = 7.9 Hz, 6H). **^{13}C NMR** (126 MHz, $CDCl_3$) δ 157.7, 157.1, 149.8, 145.4, 139.8, 128.9, 128.8, 128.7, 127.7, 127.1, 126.8, 121.4, 119.9, 47.0, 18.5, 7.5, 3.6. **IR (KBr):** 3054, 2953, 2910, 2874, 1595, 1553, 1446, 1403, 1175, 1016, 820, 776, 743, 701 cm^{-1}. **HRMS (EI)** [$C_{25}H_{31}NSi$] $[M]^+$ calculated: 373.2226, found: 373.2237.

1-Methyl-2-(4-(1-phenyl-2-(triethylsilyl)ethyl)phenyl)-1*H*-indole (4ta)

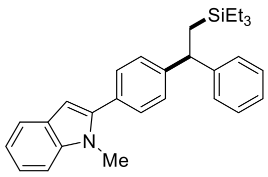

Compound **4ta** was obtained as a colorless oil (73.2 mg, Yield: 86%). **^{1}H NMR** (500 MHz, $CDCl_3$) δ 7.61 (d, *J* = 7.8 Hz, 1H), 7.39 (s, 4H), 7.35 (d, *J* = 7.3 Hz, 2H), 7.32 (d, *J* = 8.1 Hz, 1H), 7.28 (t, *J* = 7.6 Hz, 2H), 7.22 (t, *J* = 8.1 Hz, 1H), 7.17 (t, *J* = 7.3 Hz, 1H), 7.12 (t, *J* = 7.4 Hz, 1H), 6.50 (s, 1H), 4.13 (t, *J* = 7.9 Hz, 1H), 3.68 (s, 3H), 1.54 – 1.38 (m, 2H), 0.84 (t, *J* = 7.9 Hz, 9H), 0.36 (q, *J* = 7.9 Hz, 6H). **^{13}C NMR** (126 MHz, $CDCl_3$) δ 147.4, 147.0, 141.7, 138.4, 130.6, 129.5, 128.6, 128.1, 127.7, 126.3, 121.6, 120.5, 119.9, 109.7, 101.5, 47.1, 31.3, 19.2, 7.5, 3.6. **IR (KBr):** 3054, 2952, 2874, 1601, 1467, 1414, 1408, 1340, 1129, 1006, 842, 741, 703 cm^{-1}. **HRMS (EI)** [$C_{29}H_{35}NSi$] $[M]^+$ calculated: 425.2539, found: 425.2519.

(2-(4-(Benzofuran-3-yl)phenyl)-2-phenylethyl)triethylsilane (4ua)

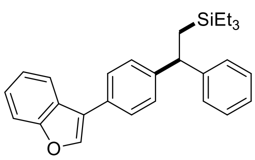

Compound **4ua** was obtained as a colorless oil (40.5 mg, Yield: 49%). **^{1}H NMR** (500 MHz, $CDCl_3$) δ 7.64 – 7.54 (m, 3H), 7.54 – 7.44 (m, 5H), 7.31 (d, *J* = 8.1 Hz, 1H), 7.28 (d, *J* = 7.1 Hz, 1H), 7.23 (t, *J* = 8.5 Hz, 2H), 6.82 (d, *J* = 10.2 Hz, 1H), 5.07 (s, 1H), 3.78 – 3.60 (m, 1H), 1.55 – 1.45 (m, 1H), 1.30 – 1.22 (m, 1H), 1.03 (t, *J* = 8.0 Hz, 9H), 0.65 – 0.41 (m, 6H). **^{13}C NMR** (126 MHz, $CDCl_3$) δ 163.4, 161.5, 153.0, 139.6, 136.2, 131.2, 130.6, 129.6, 128.8, 128.1, 127.5, 126.6, 125.3, 120.8, 115.7, 115.5 (d, *J* = 21.6 Hz), 42.2, 20.5, 7.3, 3.5. **IR (KBr):** 3056, 2953, 2909, 2874, 1601, 1508, 1487, 1458, 1426, 1408, 1232, 1159, 1014, 911, 840, 739, 703 cm^{-1}. **HRMS (EI)** [$C_{28}H_{32}OSi$] $[M]^+$ calculated: 412.2222, found: 412.2218.

(2-(Biphenyl-4-yl)-2-phenylethyl)dimethyl(phenyl)silane (4aa`)

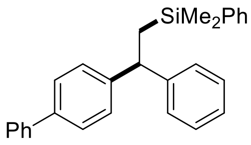

Compound **4aa`** was obtained as a colorless oil (36.9 mg, Yield: 47%). **^{1}H NMR** (500 MHz, $CDCl_3$) δ 7.68 – 7.61 (m, 2H), 7.54 – 7.45 (m, 6H), 7.42 – 7.35 (m, 10H), 7.23 – 7.18 (m, 1H), 4.14 (t, *J* = 8.0 Hz, 1H), 1.74 (d, *J* = 8.0 Hz, 2H), 0.13 (s, 3H), 0.12 (s, 3H). **^{13}C NMR** (126 MHz, $CDCl_3$) δ 146.8, 146.1, 141.1, 139.2, 139.0, 133.7, 128.9, 128.8, 128.5, 128.1, 127.8, 127.7, 127.2, 127.1, 127.1, 126.3, 47.0, 23.6, -2.4, -2.6. **IR (KBr):** 3060, 3025, 2952, 2898, 1600, 1487, 1426, 1409, 1249, 1113, 1008, 870, 836, 733, 700 cm^{-1}. **HRMS (EI)** [$C_{28}H_{28}Si$] $[M]^+$ calculated: 392.1960, found: 392.1943.

(2-(Biphenyl-4-yl)-2-phenylethyl)tripropylsilane (4aa``)

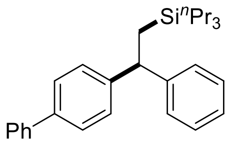

Compound **4aa``** was obtained as a colorless oil (72.9 mg, Yield: 88%). **^{1}H NMR** (500 MHz, $CDCl_3$) δ 7.62 – 7.58 (m, 2H), 7.55 – 7.51 (m, 2H), 7.48 – 7.43 (m, 2H), 7.42 – 7.40 (m, 2H), 7.40 – 7.37 (m, 2H), 7.37 – 7.34 (m, 1H), 7.34 – 7.29 (m, 2H), 7.23 – 7.18 (m, 1H), 4.16 (t, *J* = 7.9 Hz, 1H), 1.48 (d, *J* = 7.8 Hz, 2H), 1.33 – 1.18 (m, 6H), 0.91 (t, *J* = 7.2 Hz, 9H), 0.50 – 0.29 (m, 6H). **^{13}C NMR** (126 MHz, $CDCl_3$) δ 147.3, 146.7, 141.3, 139.1, 128.8, 128.5, 128.0, 127.7, 127.2, 127.14, 127.11, 126.2, 47.1, 20.2, 18.8, 17.5, 15.6. **IR (KBr)**: 3084, 3027, 2953, 2925, 1600, 1487, 1450, 1407, 1330, 1198, 1065, 1009, 820, 760, 729, 698 cm^{-1}. **HRMS (EI)** [$C_{29}H_{38}Si$] $[M]^+$ calculated: 414.2743, found: 414.2740.

(2-(Biphenyl-4-yl)-2-phenylethyl)(*tert*-butyl)dimethylsilane (4aa```)

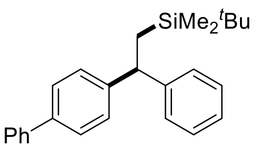

Compound **4aa```** was obtained as a colorless oil (70.2 mg, Yield: 94%). **^{1}H NMR** (500 MHz, $CDCl_3$) δ 7.66 – 7.61 (m, 2H), 7.60 – 7.55 (m, 2H), 7.50 – 7.45 (m, 2H), 7.45 – 7.42 (m, 2H), 7.42 – 7.39 (m, 2H), 7.39 – 7.33 (m, 3H), 7.28 – 7.19 (m, 1H), 4.20 (t, *J* = 7.9 Hz, 1H), 1.54 (dd, *J* = 8.0, 1.6 Hz, 2H), 0.96 (s, 9H), –0.17 (s, 3H), –0.21 (s, 3H). **^{13}C NMR** (126 MHz, $CDCl_3$) δ 147.2, 146.6, 141.1, 139.0, 128.8, 128.6, 128.0, 127.7, 127.2, 127.1, 127.1, 126.2, 47.3, 26.7, 19.7, 16.8, -5.8. **IR (KBr)**: 3060, 3028, 2954, 2922, 2855, 1602, 1486, 1469, 1407, 1362, 1248, 1172, 1009, 940, 870, 758, 730 cm^{-1}. **HRMS (EI)** [$C_{26}H3_{32}Si$] $[M]^+$ calculated: 372.2273, found: 372.2278.

(2-(Biphenyl-4-yl)-2-(naphthalen-2-yl)ethyl)triethylsilane (4ab)

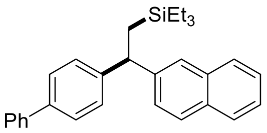

Compound **4ab** was obtained as a colorless oil (51.3 mg, Yield: 58%). **^{1}H NMR** (500 MHz, $CDCl_3$) δ 7.85 – 7.70 (m, 4H), 7.56 (d, *J* = 7.1 Hz, 2H), 7.50 (d, *J* = 8.1 Hz, 2H), 7.48 – 7.38 (m, 7H), 7.32 (t, *J* = 7.4 Hz, 1H), 4.30 (t, *J* = 7.9 Hz, 1H), 1.74 – 1.41 (m, 2H), 0.86 (t, *J* = 7.9 Hz, 9H), 0.39 (q, *J* = 7.9 Hz, 6H). **^{13}C NMR** (126 MHz, $CDCl_3$) δ 146.5, 144.7, 141.1, 139.1, 133.7, 132.3, 128.8, 128.3, 128.2, 127.9, 127.7, 127.3, 127.2, 127.1, 126.8, 126.1, 125.5, 125.4, 47.0, 18.9, 7.6, 3.7. **IR (KBr):** 3054, 3019, 2952, 2905, 2873, 1600, 1487, 1458, 1414, 1238, 1177, 1008, 844, 813, 754, 726, 696 cm^{-1}. **HRMS (EI)** [$C_{25}H_{31}NSi$] $[M]^+$ calculated: 442.2430, found: 442.2413.

(2-(Biphenyl-4-yl)-2-(*p*-tolyl)ethyl)triethylsilane (4ac)

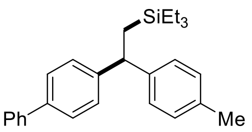

Compound **4ac** was obtained as a colorless oil (63 mg, Yield: 81%). **^{1}H NMR** (500 MHz, $CDCl_3$) δ 7.60 – 7.52 (m, 2H), 7.55 – 7.44 (m, 2H), 7.42 – 7.37 (m, 2H), 7.42 – 7.36 (m, 2H), 7.36 – 7.33 (m, 1H), 7.24 – 7.20 (m, 2H), 7.07 (d, *J* = 7.8 Hz, 2H), 4.07 (t, *J* = 7.9 Hz, 1H), 2.28 (s, 3H), 1.42 (dd, *J* = 7.9, 1.0 Hz, 2H), 0.84 (t, *J* = 7.9 Hz, 9H), 0.36 (q, *J* = 7.9 Hz, 6H). **^{13}C NMR** (126 MHz, $CDCl_3$) δ 147.0, 144.3, 141.2, 138.9, 135.6, 129.2, 128.8, 127.9, 127.5, 127.2, 127.1, 127.1, 46.6, 21.1, 19.2, 7.5, 3.7. **IR (KBr):** 3052, 3021, 2952, 2909, 2874, 1510, 1487, 1458, 1414, 1008, 802, 738, 703 cm^{-1}. **HRMS (EI)** [$C_{27}H_{34}Si$] $[M]^+$ calculated: 386.2430, found: 386.2412.

(2-(Biphenyl-4-yl)-2-(4-(*tert*-butyl)phenyl)ethyl)triethylsilane (4ad)

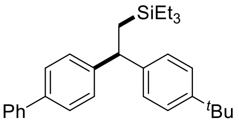

Compound **4ad** was obtained as a colorless oil (57.4 mg, Yield: 67%). **^{1}H NMR** (500 MHz, $CDCl_3$) δ 7.62 – 7.56 (m, 2H), 7.55 – 7.49 (m, 2H), 7.47 – 7.39 (m, 4H), 7.37 – 7.26 (m, 5H), 4.11 (t, *J* = 7.9 Hz, 1H), 1.53 – 1.41 (m, 2H), 1.31 (s, 9H), 0.85 (t, *J* = 7.9 Hz, 9H), 0.37 (q, *J* = 7.9 Hz, 6H). **^{13}C NMR** (126 MHz, $CDCl_3$) δ 148.9, 146.7, 144.4, 141.2, 138.9, 128.8, 128.1, 127.2, 127.1, 127.1, 125.4, 46.5, 34.5, 31.5, 19.3, 7.5, 3.6. **IR (KBr):** 3057, 2956, 2905, 2874, 1600, 1510, 1486, 1459, 1413, 175, 1008, 824, 739, 705 cm^{-1}. **HRMS (EI)** [$C_{30}H_{40}Si$] $[M]^+$ calculated: 428.2899, found:428.2882.

(2,2-Di(biphenyl-4-yl)ethyl)triethylsilane (4ae)

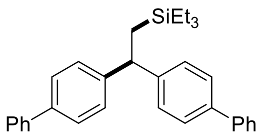

Compound **4ae** was obtained as a colorless oil (70.6 mg, Yield: 79%). **^{1}H NMR** (500 MHz, $CDCl_3$) δ 7.62 – 7.55 (m, 4H), 7.54 – 7.48 (m, 3H), 7.48 – 7.37 (m, 8H), 7.38 – 7.29 (m, 4H), 4.24 – 4.13 (m, 1H), 1.50 (t, *J* = 8.1 Hz, 2H), 0.86 (t, *J* = 7.9 Hz, 9H), 0.39 (q, *J* = 7.9 Hz, 6H). **^{13}C NMR** (126 MHz, $CDCl_3$) δ 146.5, 141.6, 141.1, 128.8, 127.3, 127.1, 126.6, 125.2, 47.1, 19.2, 7.5, 3.7. **IR (KBr):** 3060, 3028, 2952, 2874, 1600, 1486, 1413, 1179, 1008, 838, 741, 700 cm^{-1}. **HRMS (EI)** [$C_{32}H_{36}Si$] [M]$^+$ calculated: 448.2586, found: 448.2603.

(2-(Biphenyl-4-yl)-2-(4-methoxyphenyl)ethyl)triethylsilane (4af)

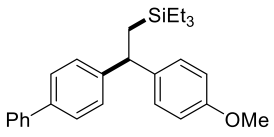

Compound **4af** was obtained as a colorless oil (65.1 mg, Yield: 81%). **^{1}H NMR** (500 MHz, $CDCl_3$) δ7.64 – 7.58 (m, 2H), 7.54 (d, *J* = 8.0 Hz, 2H), 7.45 (t, *J* = 7.6 Hz, 2H), 7.40 (d, *J* = 8.0 Hz, 2H), 7.37 – 7.32 (m, 1H), 7.30 (d, *J* = 8.4 Hz, 2H), 6.87 (d, *J* = 8.6 Hz, 2H), 4.13 (t, *J* = 7.9 Hz, 1H), 3.80 (s, 3H), 1.53 – 1.40 (m, 2H), 0.90 (t, *J* = 8.0 Hz, 9H), 0.42 (q, *J* = 7.9 Hz, 6H). **^{13}C NMR** (126 MHz, $CDCl_3$) δ 158.0, 147.2, 141.2, 139.4, 138.9, 128.8, 128.5, 127.8, 127.2, 127.1, 113.8, 55.3, 46.1, 19.3, 7.5, 3.6. **IR (KBr):** 3052, 3024, 2953, 2874, 1609, 1509, 1486, 1464, 1415, 1247, 1177, 1037, 1008, 823, 739, 699 cm^{-1}. **HRMS (EI)** [$C_{27}H_{34}OSi$] [M]$^+$ calculated: 402.2379, found: 402.2359.

(2-(Biphenyl-4-yl)-2-(2-methoxyphenyl)ethyl)triethylsilane (4ag)

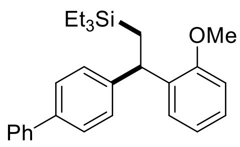

Compound **4ag** was obtained as a colorless oil (60.4 mg, Yield: 75%). **^{1}H NMR** (500 MHz, $CDCl_3$) δ 7.64 – 7.56 (m, 2H), 7.54 – 7.46 (m, 2H), 7.41 – 7.37 (m, 4H), 7.37 – 7.30 (m, 1H), 7.23 – 7.12 (m, 1H), 7.00 – 6.91 (m, 1H), 6.85 (d, *J* = 8.1 Hz, 1H), 4.69 (t, *J* = 8.0 Hz, 1H), 3.85 (s, 3H), 1.51 – 1.38 (m, 2H), 0.88 (t, *J* = 7.9 Hz, 9H), 0.40 (q, *J* = 7.9 Hz, 6H). **^{13}C NMR** (126 MHz, $CDCl_3$) δ 156.5, 146.5, 141.3, 138.6, 135.7, 128.8, 128.4, 127.8, 127.1, 127.1, 127.0, 126.9, 120.6, 110.7, 55.5, 38.3, 18.4, 7.5, 3.6. **IR (KBr):** 3052, 3029, 2953, 2909, 2874, 1599, 1488, 1464, 1240, 1110, 1032, 837, 738, 701 cm^{-1}. **HRMS (EI)** [$C_{27}H_{34}OSi$] [M]$^+$ calculated: 402.2379, found: 402.2363.

(2-(Biphenyl-4-yl)-2-(benzo[d][1,3]dioxol-5-yl)ethyl)triethylsilane (4ah)

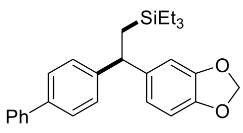

Compound **4ah** was obtained as a colorless oil (49.2 mg, Yield: 59%). **^{1}H NMR** (500 MHz, $CDCl_3$) δ 7.56 (d, *J* = 7.6 Hz, 2H), 7.50 (d, *J* = 7.9 Hz, 2H), 7.42 (t, *J* = 7.6 Hz, 2H), 7.38 – 7.29 (m, 3H), 6.85 – 6.79 (m, 2H), 6.73 (d, *J* = 8.3 Hz, 1H), 5.90 (s, 2H), 4.04 (t, *J* = 7.9 Hz, 1H), 1.40 (d, *J* = 7.9 Hz, 2H), 0.86 (t, *J* = 7.9 Hz, 9H), 0.38 (q, *J* = 7.9 Hz, 6H). **^{13}C NMR** (126 MHz, $CDCl_3$) δ 147.7, 146.8, 145.9, 141.4, 141.1, 139.0, 128.8, 127.8, 127.2, 127.1, 127.1, 120.4, 108.1, 108.1, 101.0, 46.6, 19.3, 7.5, 3.6. **IR (KBr):** 3057, 2958, 2905, 2875, 1609, 1503, 1487, 1426, 1240, 1041, 1008, 806, 741, 703 cm^{-1}. **HRMS (EI)** [$C_{27}H_{32}O_2Si$] [M]$^+$ calculated: 416.2172, found: 416.2162.

(2-(Biphenyl-4-yl)-2-(4-(methylthio)phenyl)ethyl)triethylsilane (4ai)

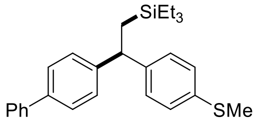

Compound **4ai** was obtained as a colorless oil (76.2 mg, Yield: 91%). **^{1}H NMR** (500 MHz, $CDCl_3$) δ 7.55 (d, *J* = 7.5 Hz, 2H), 7.48 (d, *J* = 8.1 Hz, 2H), 7.40 (t, *J* = 7.7 Hz, 2H), 7.36 – 7.28 (m, 3H), 7.27 – 7.22 (m, 2H), 7.18 (d, *J* = 8.3 Hz, 2H), 4.07 (t, *J* = 7.9 Hz, 1H), 2.44 (s, 3H), 1.47 – 1.35 (m, 2H), 0.84 (t, *J* = 7.9 Hz, 9H), 0.36 (q, *J* = 8.0 Hz, 6H). **^{13}C NMR** (126 MHz, $CDCl_3$) δ 146.5, 144.4, 141.1, 139.0, 135.7, 128.8, 128.1, 127.9, 127.3, 127.2, 127.2, 127.1, 46.4, 19.0, 16.3, 7.5, 3.6. **IR (KBr):** 3027, 2952, 2908, 2873, 1602, 1488, 1459, 1413, 1172, 1092, 1008, 815, 762, 728, 697 cm^{-1}. **HRMS (EI)** [$C_{27}H_{34}SSi$] $[M]^+$ calculated: 418.2150, found: 418.2159.

(2-([1,1'-Biphenyl]-2-yl)-2-(2-methoxyphenyl)ethyl)triethylsilane (4cg)

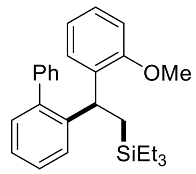

Compound **4cg** was obtained as a colorless oil (37 mg, Yield: 46%). **^{1}H NMR** (500 MHz, $CDCl_3$) δ 7.43 (d, *J* = 7.8 Hz, 1H), 7.35 – 7.25 (m, 4H), 7.24 – 7.07 (m, 6H), 6.86 (t, *J* = 7.2 Hz, 1H), 6.72 (d, *J* = 8.1 Hz, 1H), 4.70 (dd, *J* = 9.1, 6.6 Hz, 1H), 3.57 (s, 3H), 1.30 (dd, *J* = 15.0, 9.1 Hz, 1H), 1.10 (dd, *J* = 15.0, 6.6 Hz, 1H), 0.70 (t, *J* = 7.9 Hz, 9H), 0.17 (q, *J* = 7.9 Hz, 6H). **^{13}C NMR** (126 MHz, $CDCl_3$) δ 156.5, 144.7, 142.2, 141.9, 135.2, 130.1, 129.6, 128.5, 127.8, 127.7, 127.2, 126.9, 126.7, 125.5, 120.1, 110.3, 55.0, 35.1, 19.9, 7.3, 3.6. **IR (KBr):** 3064, 3026, 2952, 2909, 2874, 1599, 1489, 1475, 1460, 1414, 1241, 1182, 1034, 1010, 740, 703 cm^{-1}. **HRMS (EI)** [$C_{27}H_{34}Si$] $[M]^+$ calculated: 402.2379, found: 402.2375.

(2-(Biphenyl-2-yl)-2-(4-(methylthio)phenyl)ethyl)triethylsilane (4ci)

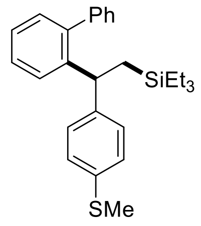

Compound **4ci** was obtained as a colorless oil (27.6 mg, Yield: 33%). **^{1}H NMR** (500 MHz, $CDCl_3$) δ 7.54 (dd, *J* = 7.9, 1.3 Hz, 1H), 7.42 – 7.31 (m, 4H), 7.24 – 7.13 (m, 4H), 7.10 (d, *J* = 8.4 Hz, 2H), 6.97 (d, *J* = 8.3 Hz, 2H), 4.23 (dd, *J* = 9.1, 6.5 Hz, 1H), 2.44 (s, 3H), 1.36 (dd, *J* = 14.9, 9.1 Hz, 1H), 1.23 (dd, *J* = 15.0, 6.6 Hz, 1H), 0.73 (t, *J* = 7.9 Hz, 9H), 0.19 (q, *J* = 7.6, 6.9 Hz, 6H). **^{13}C NMR** (126 MHz, $CDCl_3$) δ 144.6, 144.2, 141.9, 141.4, 135.2, 130.1, 129.7, 128.4, 128.0, 127.7, 127.2, 127.0, 126.8, 125.8, 41.8, 20.8, 16.3, 7.4, 3.5. **IR (KBr):** 3020, 2952, 2909, 2873, 1599, 1492, 1475, 1414, 1241, 1010, 770, 756, 739, 704 cm^{-1}. **HRMS (EI)** [$C_{27}H_{34}SSi$] $[M]^+$ calculated: 418.2150, found: 418.2164.

Triethyl(2-(*p*-tolyl)-2-(4'-(trifluoromethyl)-biphenyl-4-yl)ethyl)silane (4lc)

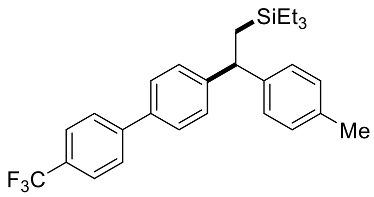

Compound **4lc** was obtained as a colorless oil (63.6mg, Yield: 70%). **^{1}H NMR** (500 MHz, $CDCl_3$) δ 7.66 (d, *J* = 1.9 Hz, 4H), 7.50 (d, *J* = 8.3 Hz, 2H), 7.41 (d, *J* = 8.2 Hz, 2H), 7.23 (d, *J* = 8.1 Hz, 2H), 7.10 (d, *J* = 7.9 Hz, 2H), 4.10 (s, 1H), 2.31 (s, 3H), 1.44 (dd, *J* = 7.9, 5.5 Hz, 2H), 0.86 (t, *J* = 7.9 Hz, 9H), 0.37 (q, *J* = 7.9 Hz, 6H). **^{13}C NMR** (126 MHz, $CDCl_3$) δ 148.1, 144.7, 144.1, 137.4, 135.8, 129.3, 129.0 (q, *J* = 32.4 Hz), 128.1, 127.4, 127.3, 127.3, 125.8 (q, *J* = 3.8 Hz), 124.5 (q, *J* = 271.8 Hz), 46.6, 21.2, 19.1, 7.5, 3.6. **^{19}F NMR** (282 MHz, $CDCl_3$) δ -62.36 (s, 3F). **IR (KBr):** 3025, 2952, 2910, 2875, 1617, 1513, 1325, 1167, 1126, 1071, 1007, 823, 782, 741 cm^{-1}. **HRMS (EI)** [$C_{28}H_{33}F_3Si$] $[M]^+$ calculated: 454.2304, found: 454.2311.

Triethyl(2-(*p*-tolyl)-2-(4'-(chloro)-biphenyl-4-yl)ethyl)silane (4mc)

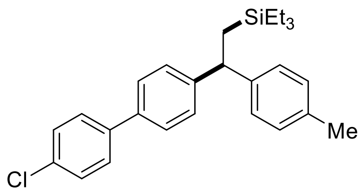

Compound **4mc** was obtained as a colorless oil (70.5 mg, Yield: 84%). **^{1}H NMR** (500 MHz, $CDCl_3$) δ 7.48 (d, *J* = 8.6 Hz, 2H), 7.45 (d, *J* = 8.3 Hz, 2H), 7.37 (dd, *J* = 8.4, 3.5 Hz, 4H), 7.23 (d, *J* = 8.1 Hz, 2H), 7.09 (d, *J* = 7.8 Hz, 2H), 4.09 (t, *J* = 7.9 Hz, 1H), 2.31 (s, 3H), 1.50 – 1.37 (m, 2H), 0.86 (t, *J* = 7.9 Hz, 9H), 0.37 (q, *J* = 7.9 Hz, 6H). **^{13}C NMR** (126 MHz, $CDCl_3$) δ 147.4, 144.2, 139.6, 137.6, 135.7, 133.1, 129.2, 128.9, 128.3, 128.0, 127.4, 127.0, 46.5, 21.1, 19.1, 7.5, 3.6. **IR (KBr):** 3016, 2951, 2909, 2873, 1510, 1485, 1414, 1235, 1093, 1015, 1005, 817, 738, 716 cm^{-1}. **HRMS (EI)** [$C_{27}H_{33}ClSi$] $[M]^+$ calculated: 420.2040, found: 420.2061.

Triethyl(2-(3-(3-methoxypropyl)phenyl)-2-(4-(methylthio)phenyl)ethyl)silane (4vi)

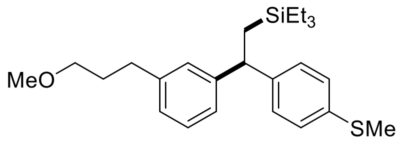

Compound **4vi** was obtained as a colorless oil (53.8 mg, Yield: 65%). **^{1}H NMR** (500 MHz, $CDCl_3$) δ 7.23 (d, *J* = 8.3 Hz, 2H), 7.20 – 7.13 (m, 3H), 7.13 – 7.07 (m, 2H), 6.98 (d, *J* = 7.5 Hz, 1H), 4.00 (t, *J* = 7.9 Hz, 1H), 3.36 (t, *J* = 6.4 Hz, 2H), 3.33 (s, 3H), 2.69 – 2.59 (m, 2H), 2.44 (s, 3H), 1.95 – 1.78 (m, 2H), 1.38 (dd, *J* = 7.9, 3.1 Hz, 2H), 0.83 (t, *J* = 7.9 Hz, 9H), 0.33 (q, *J* = 7.9 Hz, 6H). **^{13}C NMR** (126 MHz, $CDCl_3$) δ 147.2, 144.8, 142.0, 135.5, 128.4, 128.1, 127.8, 127.1, 126.3, 125.1, 72.1, 58.7, 46.7, 32.5, 31.5, 19.0, 16.4, 7.5, 3.6. **IR (KBr):** 3048, 3020, 2952, 2874, 1606, 1492, 1459, 1414, 1176, 1118, 1015, 783, 740, 705 cm^{-1}. **HRMS (EI)** [$C_{25}H_{38}OSSi$] $[M]^+$ calculated: 414.2413, found: 414.2429.

(2-(Biphenyl-4-yl)-2-(naphthalen-2-yl)ethyl)dimethyl(phenyl)silane (4ab`)

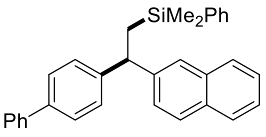

Compound **4ab`** was obtained as a colorless oil (81.3 mg, Yield: 92%). **^{1}H NMR** (500 MHz, $CDCl_3$) δ 7.82 – 7.77 (m, 2H), 7.76 – 7.71 (m, 2H), 7.60 – 7.55 (m, 2H), 7.52 – 7.41 (m, 8H), 7.40 – 7.31 (m, 7H), 4.29 (t, *J* = 8.0 Hz, 1H), 1.88 – 1.76 (m, 2H), 0.12 (s, 3H), 0.11 (s, 3H). **^{13}C NMR** (126 MHz, $CDCl_3$) δ 145.9, 144.1, 141.1, 139.2, 139.1, 133.7, 133.6, 132.3, 129.0, 128.8, 128.2, 128.2, 127.8, 127.7, 127.2, 127.2, 127.1, 126.7, 126.0, 125.6, 125.5, 47.0, 23.3, -2.4, -2.5. **IR (KBr):** 3052, 3025, 2954, 2905, 2892, 1600, 1487, 1426, 1408, 1251, 1114, 908, 836, 738, 700 cm^{-1}. **HRMS (EI)** [$C_{32}H_{30}Si$] $[M]^+$ calculated: 442.2117, found: 442.2099.

(2-(Biphenyl-4-yl)-2,2-diphenylethyl)triethylsilane (4aj)

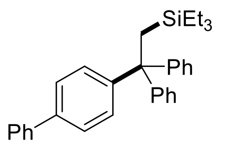

Compound **4aj** was obtained as a colorless oil (63.6 mg, Yield: 71%). **^{1}H NMR** (500 MHz, $CDCl_3$) δ 7.63 – 7.55 (m, 2H), 7.52 – 7.45 (d, *J* = 8.7 Hz, 2H), 7.45 – 7.28 (m, 9H), 7.30 – 7.22 (m, 4H), 7.22 – 7.14 (m, 2H), 2.11 (s, 2H), 0.78 (t, *J* = 8.0 Hz, 9H), 0.19 (q, *J* = 8.4, 7.8 Hz, 6H). **^{13}C NMR** (126 MHz, $CDCl_3$) δ 149.3, 148.6, 140.9, 138.5, 129.6, 129.2, 128.8, 127.8, 127.2, 127.1, 126.4, 125.9, 55.2, 26.7, 7.9, 4.6. **IR (KBr):** 3056, 3029, 2952, 2874, 1600, 1487, 1420, 1189, 1118, 1008, 833, 742, 704 cm^{-1}. **HRMS (EI)** [$C_{32}H_{36}Si$] $[M]^+$ calculated: 448.2586, found: 448.2575.

(2-(Biphenyl-4-yl)-2-phenylpropyl)triethylsilane (4ak)

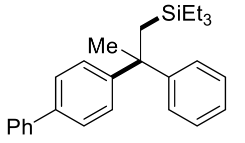

Compound **4ak** was obtained as a colorless oil (69.6 mg, Yield: 90%). ^{1}H NMR (500 MHz, $CDCl_3$) δ 7.69 – 7.58 (m, 2H), 7.57 – 7.49 (m, 2H), 7.45 (dd, *J* = 8.4, 7.0 Hz, 2H), 7.39 – 7.26 (m, 7H), 7.24 – 7.17 (m, 1H), 1.78 (s, 3H), 1.68 (s, 2H), 0.85 (t, *J* = 7.9 Hz, 9H), 0.33 (q, *J* = 8.1 Hz, 6H). **^{13}C NMR** (126 MHz, $CDCl_3$) δ 151.6, 151.3, 141.1, 138.3, 128.8, 128.0, 127.6, 127., 127.1, 127.0, 126.6, 125.7, 45.1, 30.5, 26.6, 7.6, 4.5. **IR (KBr):** 3052, 3029, 2953, 2909, 2875, 1601, 1487, 1458, 1419, 1008, 910, 841, 742, 701 cm^{-1}. **HRMS (EI)** [$C_{27}H_{34}Si$] $[M]^+$ calculated: 386.2430, found: 286.2439.

(2-(biphenyl-4-yl)-4-methoxy-2-phenylbutyl)triethylsilane (4al)

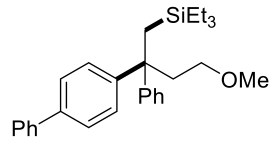

Compound **4al** was obtained as a white solid, m.p. = 64.3 – 65.0 °C (43.9 mg, Yield: 51%).**^{1}H NMR** (500 MHz, $CDCl_3$) δ 7.59 (dd, *J* = 8.3, 1.2 Hz, 2H), 7.48 (d, *J* = 8.5 Hz, 2H), 7.41 (t, *J* = 7.7 Hz, 2H), 7.35 – 7.29 (m, 1H), 7.28 (d, *J* = 8.5 Hz, 2H), 7.27 – 7.22 (m, 4H), 7.17 (dq, *J* = 5.2, 2.8 Hz, 1H), 3.21 (s, 3H), 3.14 – 3.04 (m, 2H), 2.57 – 2.43 (m, 2H), 1.59 (s, 2H), 0.79 (t, *J* = 7.9 Hz, 9H), 0.24 (q, *J* = 7.5, 6.9 Hz, 6H). **^{13}C NMR** (126 MHz, $CDCl_3$) δ 150.2, 149.5, 140.9, 138.5, 128.8, 128.0, 127.6, 127.2, 127.0, 126.5, 125.9, 70.3, 58.9, 46.8, 40.1, 23.7, 7.6, 4.2. 0.2. **IR (KBr):** 3055, 3028, 2952, 2910, 2875, 1600, 1487, 1459, 1414, 1116, 1007, 969, 837, 759, 699 cm^{-1}. **HRMS (EI)** [$C_{29}H_{38}Si$] $[M]^+$ calculated: 430.2692, found: 430.2690.

(2,2-Diphenylpropyl)triethylsilane (4fk)

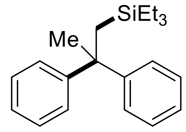

Compound **4fk** was obtained as a colorless oil (90.6 mg, Yield: 73%). **^{1}H NMR** (500 MHz, $CDCl_3$) δ 7.27 – 7.20 (m, 8H), 7.17 – 7.11 (m, 2H), 1.69 (s, 3H), 1.60 (s, 2H), 0.79 (t, *J* = 8.0 Hz, 9H), 0.25 (q, *J* = 7.9 Hz, 6H). **^{13}C NMR** (126 MHz, $CDCl_3$) δ 151.9, 127.9, 127.2, 125.6, 45.2, 30.6, 26.5, 7.6, 4.5. **IR (KBr):** 3058, 3025, 2953, 2909, 2875, 1599, 1493, 1444, 1416, 1240, 1018, 908, 835, 742, 700 cm^{-1}. **HRMS (EI)** [$C_{21}H_{30}Si$] $[M]^+$ calculated: 310.2117, found: 310.2113.

(2-(4'-Chloro-biphenyl-4-yl)-2-phenylpropyl)triethylsilane (4mk)

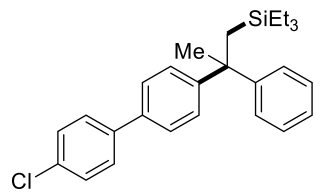

Compound **4mk** was obtained as a colorless oil (58 mg, Yield: 69%). **^{1}H NMR** (500 MHz, $CDCl_3$) δ 7.51 – 7.47 (m, 2H), 7.46 – 7.41 (m, 2H), 7.39 – 7.35 (m, 2H), 7.34 – 7.23 (m, 6H), 7.16 (tt, *J* = 6.3, 1.7 Hz, 1H), 1.72 (s, 3H), 1.62 (d, *J* = 1.7 Hz, 2H), 0.80 (t, *J* = 7.9 Hz, 9H), 0.27 (qd, *J* = 7.8, 0.8 Hz, 6H). **^{13}C NMR** (126 MHz, $CDCl_3$) δ 151.7, 151.5, 139.5, 137.1, 133.2, 129.0, 128.3, 128.0, 127.7, 127.2, 126.4, 125.8, 45.1, 30.5, 26.5, 7.6, 4.5. **IR (KBr):** 3052, 2953, 2909, 2875, 1485, 1417, 1238, 1093, 1004, 818, 749, 735, 704 cm^{-1}. **HRMS (EI)** [$C_{27}H_{33}ClSi$] $[M]^+$ calculated: 420.2040, found: 420.2037.

(2,6-Diphenylhexyl)triethylsilane (5aa)

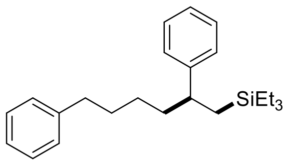

Compound **5aa** was obtained as a colorless oil (without Nickel catalysis: 66.1 mg, Yield: 94%; with Nickel catalysis: 66.9 mg, Yield: 95%). **^{1}H NMR** (500 MHz, $CDCl_3$) δ 7.30 – 7.20 (m, 4H), 7.21 – 7.12 (m, 4H), 7.13 – 7.06 (m, 2H), 2.68 – 2.56 (m, 1H), 2.55 – 2.36 (m, 2H), 1.72 – 1.47 (m, 3H), 1.31 – 1.06 (m, 2H), 0.99 – 0.90 (m, 2H), 0.82 (t, J = 8.0 Hz, 9H), 0.49 – 0.22 (m, 6H). **^{13}C NMR** (126 MHz, $CDCl_3$) δ 148.1, 143.0, 128.5, 128.3, 128.3, 127.6, 126.0, 125.7, 41.9, 41.0, 36.0, 31.7, 27.7, 20.1, 7.5, 3.8. **IR (KBr):** 3027, 2951, 2933, 2911, 2873, 1599, 1493, 1453, 1412, 1015, 778, 763, 740, 700 cm^{-1}. **HRMS (EI)** [$C_{22}H_{31}Si$] [M-CH_2CH_3]$^+$ calculated: 323.2195, found: 323.2192.

(3-(Biphenyl-4-yl)-2-phenylpropyl)triethylsilane (5ba)

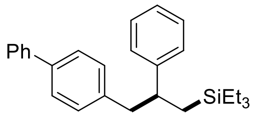

Compound **5ba** was obtained as a colorless oil (without Nickel catalysis: 63.6 mg, Yield: 89%; with Nickel catalysis: 70.5 mg, Yield: 91%). **^{1}H NMR** (500 MHz, $CDCl_3$) δ 7.55 (dd, J = 8.3, 1.3 Hz, 2H), 7.40 (dd, J = 16.6, 8.1 Hz, 4H), 7.29 (t, J = 7.4 Hz, 1H), 7.26 – 7.19 (m, 2H), 7.17 – 7.12 (m, 3H), 7.04 (d, J = 8.2 Hz, 2H), 3.07 – 2.77 (m, 3H), 1.02 (d, J = 7.1 Hz, 2H), 0.78 (t, J = 7.9 Hz, 9H), 0.43 – 0.18 (m, 6H). **^{13}C NMR** (126 MHz, $CDCl_3$) δ 146.9, 141.2, 140.1, 138.6, 129.7, 128.8, 128.2, 127.7, 127.1, 127.0, 126.8, 126.2, 47.6, 44.2, 18.7, 7.5, 3.6. **IR (KBr):** 3027, 2952, 2909, 2873, 1599, 1489, 1453, 1409, 1184, 1009, 910, 844, 762, 738, 699 cm^{-1}. **HRMS (EI)** [$C_{25}H_{29}Si$] [M-CH_2CH_3]$^+$ calculated: 357.2039, found: 357.2046.

Triethyl(3-(naphthalen-1-yl)-2-phenylpropyl)silane (5ca)

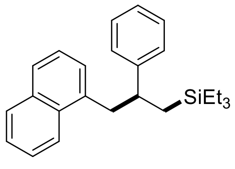

Compound **5ca** was obtained as a colorless oil (without Nickel catalysis: 61.1 mg, Yield: 85%; with Nickel catalysis: 60.0 mg, Yield: 83%). **^{1}H NMR** (500 MHz, $CDCl_3$) δ 7.87 (d, J = 8.9 Hz, 1H), 7.66 (d, J = 7.5 Hz, 1H), 7.49 (d, J = 8.2 Hz, 1H), 7.31 (ddd, J = 17.0, 8.1, 1.2 Hz, 2H), 7.12 – 7.06 (m, 1H), 7.04 (dd, J = 8.9, 5.8 Hz, 2H), 7.00 – 6.94 (m, 3H), 6.86 (d, J = 6.7 Hz, 1H), 3.14 (d, J = 7.2 Hz, 2H), 2.96 (dt, J = 10.6, 3.6 Hz, 1H), 1.08 – 0.77 (m, 2H), 0.53 (t, J = 7.9 Hz, 9H), 0.05 (qd, J = 14.8, 7.5 Hz, 6H). **^{13}C NMR** (126 MHz, $CDCl_3$) δ 147.1, 136.9, 134.0, 132.1, 128.9, 128.2, 127.6, 127.5, 126.7, 126.2, 125.7, 125.34, 125.28, 124.0, 45.6, 43.0, 18.6, 7.3, 3.6. **IR (KBr):** 3025, 2952, 2909, 2873, 1595, 1491, 1454, 1395, 1016, 791, 775, 740, 701 cm^{-1}. **HRMS (EI)** [$C_{25}H_{32}Si$] [M]$^+$ calculated: 360.2273, found: 360.2278.

Triethyl(2-phenyl-3-(4-(trifluoromethoxy)phenyl)propyl)silane (5da)

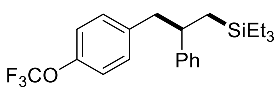

Compound **5da** was obtained as a colorless oil (without Nickel catalysis: 66.5 mg, Yield: 91%; with Nickel catalysis: 73.3 mg, Yield: 93%). **^{1}H NMR** (500 MHz, $CDCl_3$) δ 7.24 – 7.17 (m, 2H), 7.17 – 7.10 (m, 1H), 7.11 – 7.05 (m, 2H), 7.01 (d, J = 7.8 Hz, 2H), 6.98 – 6.92 (m, 2H), 2.92 – 2.80 (m, 3H), 1.07 – 0.92 (m, 2H), 0.77 (t, J = 8.0 Hz, 9H), 0.39 – 0.20 (m, 6H). **^{13}C NMR** (126 MHz, $CDCl_3$) δ 147.5 (q, J = 1.8 Hz), 146.4, 139.8, 130.4, 128.3, 127.7, 126.3, 120.63 (q, J = 256.5 Hz), 120.6, 47.3, 44.4, 18.7, 7.4, 3.6. **^{19}F NMR** (282 MHz, $CDCl_3$) δ -58.37 (s, 3F). **IR (KBr):** 3025, 2953, 2911, 2875, 1599, 1508, 1455, 1453, 1415, 1200, 1166, 1018, 846, 756, 741, 700 cm^{-1}. **HRMS (EI)** [$C_{20}H_{24}F_3OSi$] [M-CH_2CH_3]$^+$ calculated: 365.1549, found: 365.1546.

Triethyl(2-(4-(methylthio)phenyl)dodecyl)silane (5ei)

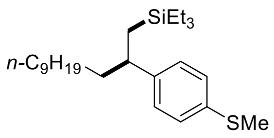

Compound **5ei** was obtained as a colorless oil (without Nickel catalysis: 57.3 mg, Yield: 76%; with Nickel catalysis: 58.4mg, Yield: 72%). **^{1}H NMR** (500 MHz, $CDCl_3$) δ 7.18 (d, *J* = 8.3 Hz, 2H), 7.09 (d, *J* = 8.3 Hz, 2H), 2.57 (tt, *J* = 9.1, 5.6 Hz, 1H), 2.47 (s, 3H), 1.66 – 1.44 (m, 2H), 1.36 – 1.08 (m, 14H), 0.92 – 0.87 (m, 7H), 0.84 (t, *J* = 7.9 Hz, 9H), 0.44 – 0.23 (m, 6H). **^{13}C NMR** (126 MHz, $CDCl_3$) δ 145.6, 135.0, 128.1, 127.0, 41.4, 41.1, 32.1, 29.79, 29.76, 29.7, 29.5, 27.9, 22.8, 20.0, 16.5, 14.3, 7.5, 3.8. **IR (KBr):** 3012, 2953, 2925, 2873, 2854, 1599, 1492, 1465, 1414, 1237, 1015, 826, 757, 741 cm^{-1}. **HRMS (EI)** [$C_{23}H_{41}SSi$] [$M-CH_2CH_3$]$^+$ calculated: 377.2698, found: 377.2694.

Triethyl(2-(4-(methylthio)phenyl)-5-phenylpentyl)silane (5fi)

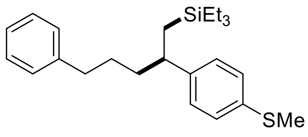

Compound **5fi** was obtained as a colorless oil (66.6 mg, Yield: 86%). **^{1}H NMR** (300 MHz, $CDCl_3$) δ 7.25 (d, *J* = 7.3 Hz, 2H), 7.19 (d, *J* = 8.0 Hz, 3H), 7.11 (t, *J* = 7.1 Hz, 4H), 2.70 – 2.57 (m, 1H), 2.61 – 2.48 (m, 2H), 2.48 (s, 3H), 1.71 – 1.53 (m, 2H), 1.54 – 1.32 (m, 2H), 0.99 – 0.87 (m, 2H), 0.84 (t, *J* = 8.0 Hz, 9H), 0.49 – 0.21 (m, 6H). **^{13}C NMR** (75 MHz, $CDCl_3$) δ 145.1, 142.7, 135.2, 128.5, 128.3, 128.0, 127.1, 125.7, 41.4, 40.6, 36.0, 29.7, 20.0, 16.5, 7.5, 3.8. **IR (KBr):** 3062, 3025, 2932, 2870, 1603, 1495, 1455, 1415, 1238, 1094, 1015, 967, 825, 759, 699 cm^{-1}. **HRMS (EI)** [$C_{24}H_{36}SSi$] [M]$^+$ calculated: 384.2307, found: 384.2296.

Triethyl(2-(4-(methylthio)phenyl)-4-phenylbutyl)silane (5gi)

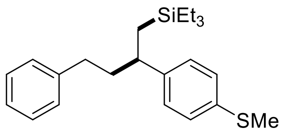

Compound **5gi** was obtained as a colorless oil (29.8 mg, Yield: 40%). **^{1}H NMR** (300 MHz, $CDCl_3$) δ 7.38 – 6.97 (m, 9H), 2.72 – 2.54 (m, 1H), 2.48 (s, 3H), 2.39 (t, *J* = 8.1 Hz, 2H), 2.02 – 1.75 (m, 2H), 0.98 – 0.88 (m, 2H), 0.81 (t, *J* = 7.9 Hz, 9H), 0.45 – 0.17 (m, *J* = 7.4 Hz, 6H). **^{13}C NMR** (75 MHz, $CDCl_3$) δ 144.9, 142.6, 135.4, 128.5, 128.4, 128.2, 127.1, 125.7, 42.7, 41.1, 34.1, 20.1, 16.5, 7.5, 3.7. **IR (KBr):** 3062, 3024, 2951, 2872, 1495, 1455, 1415, 1239, 1095, 1015, 968, 821, 760, 699 cm^{-1}. **HRMS (EI)** [$C_{23}H_{34}SSi$] [M]$^+$ calculated: 370.2150, found: 370.2159.

(5-(Adamantan-1-yl)-2-(*p*-tolyl)pentyl)triethylsilane (5hc)

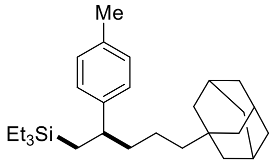

Compound **5hc** was obtained as a colorless oil (68.1 mg, Yield: 83%). **^{1}H NMR** (500 MHz, $CDCl_3$) δ 7.13 – 7.00 (m, 4H), 2.67 – 2.56 (m, 1H), 2.33 (s, 3H), 1.90 (s, 3H), 1.72 – 1.56 (m, 6H), 1.56 – 1.47 (m, 2H), 1.39 (d, *J* = 2.8 Hz, 6H), 1.14 – 1.01 (m, 3H), 0.99 – 0.88 (m, 3H), 0.85 (t, *J* = 7.9 Hz, 9H), 0.47 – 0.25 (m, 6H). **^{13}C NMR** (126 MHz, $CDCl_3$) δ 145.3, 135.1, 128.9, 127.4, 44.9, 42.7, 42.2, 41.3, 37.4, 32.4, 28.9, 21.2, 20.5, 20.1, 7.6, 3.8. **IR (KBr)**: 3047, 3020, 2949, 2834, 1513, 1455, 1415, 1377, 1238, 1015, 972, 821, 758, 719 cm^{-1}. **HRMS (EI)** [$C_{28}H_{46}Si$] [M]$^+$ calculated: 410.3369, found: 410.3364.

3-Cyclohexyl-2,3-diphenylpropyl)triethylsilane (5ia)

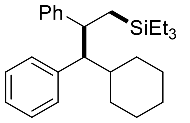

Compound **5ia** was obtained as a colorless oil (28.3 mg, Yield: 36%). **^{1}H NMR** (500 MHz, $CDCl_3$) δ 7.33 – 7.29 (m, 2H), 7.28 – 7.22 (m, 3H), 7.21 – 7.16 (m, 2H), 7.13 (t, *J* = 7.3 Hz, 1H), 7.00 (d, *J* = 8.0 Hz, 2H), 4.03 (t, *J* = 7.9 Hz, 1H), 2.40 (d, *J* = 7.3 Hz, 2H), 1.71 – 1.56 (m, 4H), 1.39 (d, *J* = 8.0 Hz, 2H), 1.21 – 1.08 (m, 2H), 0.99 – 0.85 (m, 2H), 0.87 – 0.76 (m, 11H), 0.31 (q, *J* = 8.0 Hz, 6H). **^{13}C NMR** (126 MHz, $CDCl_3$) δ 147.7, 144.6, 139.0, 129.2, 128.4, 127.6, 127.3, 126.0, 46.9, 43.8, 42.8, 41.6, 39.9, 33.2, 26.7, 26.5, 19.3, 7.5, 3.6. **IR (KBr):** 3026, 2951, 2923, 2874, 2852, 1599, 1509, 1493, 1450, 1416, 1173, 1016, 741, 700 cm^{-1}. **HRMS (EI)** [$C_{27}H_{40}Si$] $[M]^+$ calculated: 392.2899, found: 392.2910.

Triethyl(2,3,5-triphenylpentyl)silane (5ja)

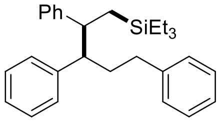

Compound **5ja** was obtained as a colorless oil (40.1 mg, Yield: 52%). **^{1}H NMR** (500 MHz, $CDCl_3$) δ 7.32 (t, *J* = 7.4 Hz, 2H), 7.27 – 7.19 (m, 3H), 7.21 – 7.04 (m, 8H), 6.86 (d, *J* = 6.8 Hz, 2H), 2.84 – 2.75 (m, 1H), 2.68 – 2.55 (m, 1H), 2.29 (dt, *J* = 14.2, 7.3 Hz, 1H), 2.12 (dt, *J* = 13.9, 8.4 Hz, 1H), 1.72 – 1.62 (m, 2H), 0.71 (dd, *J* = 14.8, 12.1 Hz, 1H), 0.68 – 0.57 (m, 10H), 0.25 – 0.02 (m, 6H). **^{13}C NMR** (126 MHz, $CDCl_3$) δ 145.8, 144.5, 142.5, 128.8, 128.51, 128.46, 128.39, 128.2, 128.2, 126.3, 126.3, 125.6, 55.1, 48.3, 35.6, 33.9, 17.1, 7.3, 3.3. **IR (KBr):** 3027, 2951, 2909, 2873, 1603, 1494, 1453, 1415, 1180, 1001, 762, 741, 700 cm^{-1}. **HRMS (EI)** [$C_{27}H_{33}Si$] $[M-CH_2CH_3]^+$ calculated: 385.2352, found: 385.2351.

Triethyl((*2S*)-2-phenyl-2-(4-(((2,5,7,8-tetramethyl-2-(4,8,12-trimethyltridecyl)chroman-6-yl)oxy)methyl)phenyl)ethyl)silane (4wa)

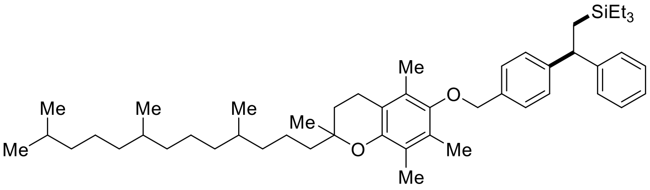

Compound **4wa** was obtained as a colorless oil (63.4 mg, Yield: 43%). **^{1}H NMR** (500 MHz, $CDCl_3$) δ 7.38 (d, *J* = 8.2 Hz, 2H), 7.35 – 7.28 (m, 4H), 7.27 – 7.21 (m, 2H), 7.14 (t, *J* = 7.3 Hz, 1H), 4.62 (s, 2H), 4.09 (t, *J* = 7.9 Hz, 1H), 2.57 (t, *J* = 6.9 Hz, 2H), 2.19 (s, 3H), 2.14 (s, 3H), 2.09 (s, 3H), 1.89 – 1.70 (m, 2H), 1.61 – 1.47 (m, 4H), 1.47 – 1.33 (m, 6H), 1.30 – 1.23 (m, 7H), 1.17 – 1.00 (m, 7H), 0.90 – 0.78 (m, 23H), 0.35 (q, *J* = 8.0 Hz, 6H). **^{13}C NMR** (126 MHz, $CDCl_3$) δ 148.2, 148.0, 147.3, 147.2, 135.8, 128.5, 128.1, 128.0, 127.6, 126.1, 123.0, 117.7, 74.9, 74.7, 47.0, 40.2, 39.5, 37.7, 37.6, 37.6 – 37.5 (m), 37.5, 37.4, 32.9, 32.8, 31.4, 28.1, 25.0, 24.6, 24.0, 22.9, 22.8, 21.2, 20.8, 19.9, 19.8, 19.1, 13.0, 12.2, 12.0, 7.5, 3.6. **IR (KBr):** 3029, 2952, 2928, 2872, 1603, 1459, 1412, 1375, 1251, 1173, 1087, 1015, 772, 761, 739, 700 cm^{-1}. **HRMS (ESI)** [$C_{50}H_{78}O_2NaSi$] $[M+Na]^+$ calculated: 761.5669, found: 761.5661.

Menthol derivative (4xa)

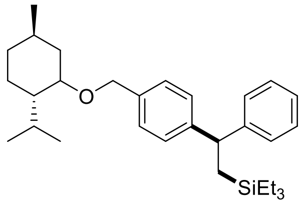

Compound **4xa** was obtained as a colorless oil (51 mg, Yield: 55%). **^{1}H NMR** (500 MHz, $CDCl_3$) δ 7.30 – 7.17 (m, 8H), 7.12 (t, *J* = 7.0 Hz, 1H), 4.59 (d, *J* = 11.4 Hz, 1H), 4.33 (d, *J* = 11.3 Hz, 1H), 4.05 (t, *J* = 7.9 Hz, 1H), 3.20 – 3.07 (m, 1H), 2.30 – 2.23 (m, 1H), 2.20 – 2.10 (m, 1H), 1.69 – 1.56 (m, 2H), 1.39 (d, *J* = 7.9 Hz, 2H), 1.36 – 1.23 (m, 2H),

0.99 – 0.89 (m, 5H), 0.89 – 0.85 (m, 4H), 0.82 (t, *J* = 8.0 Hz, 9H), 0.67 (dd, *J* = 6.9, 4.3 Hz, 3H), 0.39 – 0.28 (m, 6H). **^{13}C NMR** (126 MHz, $CDCl_3$) δ 147.5, 146.6, 136.8, 128.4, 128.1 (d, *J* = 11.6 Hz), 127.6, 127.5, 126.0, 78.6 (d, *J* = 10.4 Hz), 70.3, 48.4, 46.9, 40.4, 34.7, 31.7, 25.6, 23.3, 22.5, 21.2, 19.0, 16.1, 7.5, 3.6. **IR (KBr):** 3029, 2953, 2917, 2872, 1599, 1492, 1455, 1416, 1342, 1238, 1177, 1110, 1085, 1071, 1017, 769, 736, 699 cm^{-1}. **HRMS (EI)** [$C_{31}H_{48}OSi$] $[M]^+$ calculated: 464.3474, found: 464.3488.

Estrone derivative (4ya)

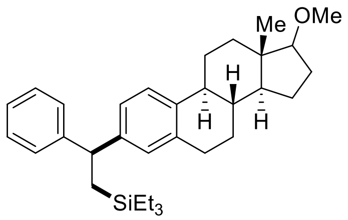

Compound **4ya** was obtained as a colorless oil (51.8 mg, Yield: 53%). **^{1}H NMR** (500 MHz, $CDCl_3$) δ 7.32 – 7.27 (m, 2H), 7.27 – 7.20 (m, 2H), 7.19 – 7.09 (m, 2H), 7.10 – 7.03 (m, 1H), 6.99 (d, *J* = 5.5 Hz, 1H), 3.99 (dd, *J* = 9.3, 6.5 Hz, 1H), 3.36 (s, 3H), 3.29 (t, *J* = 8.3 Hz, 1H), 2.85 – 2.77 (m, 2H), 2.30 – 2.21 (m, 1H), 2.21 – 2.14 (m, 1H), 2.10 – 2.00 (m, 2H), 1.88 – 1.81 (m, 1H), 1.72 – 1.63 (m, 1H), 1.56 – 1.25 (m, 8H), 1.21 – 1.12 (m, 1H), 0.82 (td, *J* = 7.9, 1.0 Hz, 9H), 0.76 (s, 3H), 0.38 – 0.26 (m, 6H). **^{13}C NMR** (126 MHz, $CDCl_3$) δ 147.3, 144.9, 138.0, 136.5, 128.4, 127.9 (d, *J* = 12.5 Hz), 127.7, 126.0, 125.4, 124.7 (d, *J* = 12.2 Hz), 90.9, 58.0, 50.5, 46.8, 44.3, 43.3, 38.5, 38.2, 29.8, 27.9, 27.4, 26.3, 23.2, 19.1, 11.7, 7.5, 3.6. **IR (KBr):** 3060, 3020, 2951, 2909, 2873, 1599, 1494, 1452, 1416, 1134, 1105, 1015, 766, 738, 700 cm^{-1}. **HRMS (ESI)** [$C_{33}H_{48}ONaSi$] $[M+Na]^+$ calculated: 511.3372, found: 511.3375.

Estrone derivative (4am)

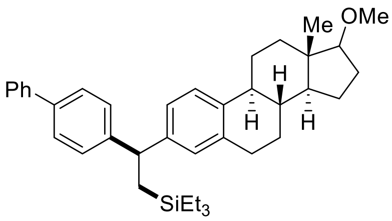

Compound **4am** was obtained as a colorless oil (89.2 mg, Yield: 79%). **^{1}H NMR** (500 MHz, $CDCl_3$) δ 7.61 – 7.55 (m, 2H), 7.53 – 7.48 (m, 2H), 7.46 – 7.37 (m, 4H), 7.36 – 7.29 (m, 1H), 7.24 – 7.18 (m, 1H), 7.17 – 7.10 (m, 1H), 7.08 – 7.03 (m, 1H), 4.06 (dd, *J* = 8.6, 7.2 Hz, 1H), 3.39 (s, 3H), 3.32 (t, *J* = 8.3 Hz, 1H), 2.89 – 2.82 (m, 2H), 2.33 – 2.25 (m, 1H), 2.26 – 2.16 (m, 1H), 2.14 – 2.02 (m, 2H), 1.93 – 1.84 (m, 1H), 1.74 – 1.65 (m, 1H), 1.56 – 1.44 (m, 3H), 1.45 – 1.30 (m, 4H), 1.26 – 1.16 (m, 1H), 0.91 (t, *J* = 7.0 Hz, 1H), 0.86 (td, *J* = 7.9, 0.9 Hz, 9H), 0.79 (s, 3H), 0.37 (q, *J* = 7.8 Hz, 6H). **^{13}C NMR** (126 MHz, $CDCl_3$) δ 146.6, 144.8, 141.2, 138.9, 138.1, 136.6, 128.8, 128.0, 127.9 (d, *J* = 9.6 Hz), 127.2, 127.1, 125.5, 124.7 (d, *J* = 8.7 Hz), 90.9, 58.0, 50.5, 46.6, 44.4, 43.3, 38.5, 38.2, 29.8, 27.9, 27.4, 26.3, 23.2, 19.2, 11.7, 7.5, 3.6. **IR (KBr):** 3048, 3029, 2951, 2873, 1606, 1486, 1459, 1414, 1134, 1004, 838, 759, 738, 701 cm^{-1}. **HRMS (ESI)** [$C_{39}H_{52}ONaSi$] $[M+Na]^+$ calculated: 587.3685, found: 587.3678.

Liquid crystal material derivative (4zi)

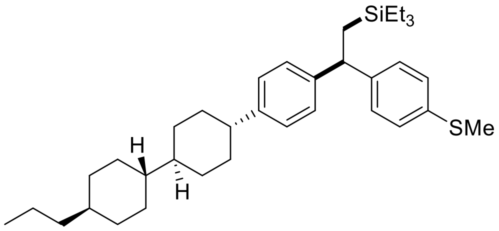

Compound **4zi** was obtained as white solid, m.p. = 148.0 – 150.3 °C (45 mg, Yield: 41%). **^{1}H NMR** (500 MHz, $CDCl_3$) δ 7.23 (d, *J* = 8.4 Hz, 2H), 7.17 (t, *J* = 7.9 Hz, 4H), 7.07 (d, *J* = 8.3 Hz, 2H), 3.99 (t, *J* = 7.9 Hz, 1H), 2.44 (s, 3H), 2.38 (tt, *J* = 12.2, 3.4 Hz, 1H), 1.90 – 1.83 (m, 2H), 1.84 – 1.78 (m, 2H), 1.80 – 1.68 (m, 4H), 1.41 – 1.35 (m, 3H), 1.35 – 1.26 (m, 3H), 1.18 – 1.09 (m, 6H), 1.05 – 0.94 (m,

3H), 0.88 (t, *J* = 7.3 Hz, 4H), 0.85 – 0.76 (t, *J* = 7.9 Hz, 10H), 0.32 (q, *J* = 7.9 Hz, 6H). ^{13}**C NMR** (126 MHz, $CDCl_3$) δ 145.7, 144.8, 144.7, 135.4, 128.2, 127.2, 127.1, 126.9, 46.4, 44.3, 43.6, 43.0, 40.0, 37.8, 34.7 (d, *J* = 2.8 Hz), 33.7, 30.5, 30.2, 20.2, 19.1, 16.4, 14.6, 7.5, 3.6. **IR (KBr):** 3052, 3016, 2952, 2920, 2873, 2851, 1610, 1492, 1448, 1411, 1172, 1135, 1015, 802, 740, 705 cm^{-1}. **HRMS (ESI)** [$C_{36}H_{56}ONaSi$] $[M+Na]^+$ calculated: 571.3770, found: 571.3742.

(4-(Biphenyl-4-yl)-4-phenylbut-2-en-1-yl)triethylsilane (4an)

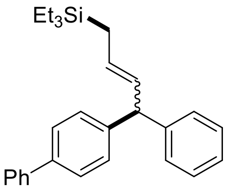

Compound **4an** was obtained as a colorless oil (32.5 mg, Yield: 41%). The *E/Z* (1.1/1) ratio was determined by ^{1}H NMR. (*E*)**-4am**: ^{1}**H NMR** (500 MHz, $CDCl_3$) δ 7.58 – 7.55 (m, 2H), 7.50 (d, *J* = 2.4 Hz, 2H), 7.41 (t, *J* = 7.7 Hz, 2H), 7.34 – 7.27 (m, 4H), 7.27 – 7.23 (m, 3H), 7.23 – 7.17 (m, 1H), 5.80 – 5.73 (m, 1H), 5.44 (dt, *J* = 15.0, 8.1 Hz, 1H), 4.71 (d, *J* = 7.6 Hz, 1H), 1.56 (dd, *J* = 8.1, 1.2 Hz, 2H), 0.91 (t, *J* = 7.9 Hz, 9H), 0.51 (q, *J* = 7.9 Hz, 6H) ^{13}**C NMR** (126 MHz, $CDCl_3$) δ 144.7, 143.9, 141.2, 139.1, 130.7, 129.0, 128.8, 128.6, 128.4, 127.2, 127.1, 126.2, 54.1, 48.0, 17.9, 13.7, 7.5, 3.4. **IR (KBr):** 3056, 3029, 2953, 2909, 2874, 1601, 1487, 1415, 1191, 1009, 969, 840, 741, 703 cm^{-1}. **HRMS (EI)** [$C_{28}H_{34}Si$] $[M]^+$ calculated: 398.2430, found: 398.2434.

(2-(Biphenyl-4-yl)-2-phenylbut-3-en-1-yl)triethylsilane (4ao)

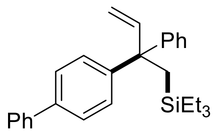

Compound **4ao** was obtained as a colorless oil (51.6 mg, Yield: 65%). ^{1}**H NMR** (500 MHz, $CDCl_3$) δ 7.62 (dd, *J* = 8.3, 1.2 Hz, 2H), 7.52 (d, *J* = 8.5 Hz, 2H), 7.44 (s, 2H), 7.37 – 7.26 (m, 7H), 7.22 (d, *J* = 4.2 Hz, 1H), 6.74 – 6.50 (m, 1H), 5.19 (d, *J* = 10.6 Hz, 1H), 4.66 (d, *J* = 18.5 Hz, 1H), 1.77 (s, 2H), 0.84 (t, *J* = 7.9 Hz, 9H), 0.31 (q, *J* = 8.1 Hz, 6H). ^{13}**C NMR** (126 MHz, $CDCl_3$) δ 148.6, 148.0, 146.5, 141.0, 138.6, 128.8, 128.4, 127.9, 127.2, 127.1, 126.5, 126.1, 114.6, 52.6, 24.8, 7.7, 4.7. **IR (KBr):** 3029, 2952, 2909, 2874, 1603, 1487, 1409, 1174, 1008, 974, 918, 836, 771, 738, 701 cm^{-1}. **HRMS (EI)** [$C_{28}H_{34}Si$] $[M]^+$ calculated: 398.2430, found: 398.2440.

(2-(2-(But-3-en-1-yl)phenyl)-2-phenylethyl)triethylsilane (7aa)

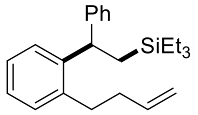

Compound **7aa** was obtained as a colorless oil (35.8 mg, Yield: 51%). ^{1}**H NMR** (500 MHz, $CDCl_3$) δ 7.34 – 7.30 (m, 2H), 7.29 – 7.24 (m, 2H), 7.22 – 7.12 (m, 4H), 7.02 – 6.98 (m, 1H), 5.87 (ddt, *J* = 16.9, 10.2, 6.6 Hz, 1H), 5.11 – 4.96 (m, 2H), 4.06 (t, *J* = 7.9 Hz, 1H), 2.72 – 2.65 (m, 2H), 2.41 – 2.33 (m, 2H), 1.43 (dd, *J* = 7.9, 2.0 Hz, 2H), 0.85 (t, *J* = 7.9 Hz, 9H), 0.35 (q, *J* = 7.9 Hz, 6H). ^{13}**C NMR** (126 MHz, $CDCl_3$) δ 147.6, 147.3, 141.9, 138.3, 128.4, 128.4, 127.8, 127.6, 126.2, 126.0, 125.2, 115.0, 47.2, 35.7, 35.6, 19.1, 7.5, 3.6. **IR (KBr):** 3060, 3026, 2952, 2910, 2874, 1641, 1602, 1493, 1453, 1415, 1240, 1175, 1015, 913, 790, 742, 707 cm^{-1}. **HRMS (EI)** [$C_{24}H_{34}Si$] $[M]^+$ calculated: 350.2430, found: 350.2430, [$C_{22}H_{29}Si$] $[M-CH_2CH_3]^+$ calculated: 321.2039, found: 321.2024.

Triethyl(2-phenyloct-7-en-1-yl)silane (5ka)

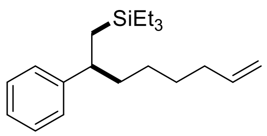

Compound **5ka** was obtained as a colorless oil (56.1 mg, Yield: 93%). ^{1}**H NMR** (500 MHz, $CDCl_3$) δ 7.32 – 7.25 (m, 2H), 7.22 – 7.14 (m, 3H), 5.78 (ddt, *J* = 16.9, 10.2, 6.7 Hz, 1H), 5.04 – 4.87 (m, 2H), 2.64 (tt, *J* = 9.0, 5.1 Hz, 1H), 2.03 – 1.95 (m, 2H), 1.70 – 1.55 (m, 2H), 1.41 – 1.26

(m, 2H), 1.26 – 1.06 (m, 2H), 1.02 – 0.92 (m, 2H), 0.85 (t, *J* = 7.9 Hz, 9H), 0.46 – 0.27 (m, 6H). **^{13}C NMR** (126 MHz, $CDCl_3$) δ 148.2, 139.2, 128.3, 127.5, 125.9, 114.2, 41.9, 41.0, 33.9, 29.1, 27.4, 20.1, 7.5, 3.8. **IR (KBr)**: 3081, 3063, 3027, 2952, 2874, 1603, 1494, 1454, 1415, 1236, 1015, 909, 741, 700 cm^{-1}. **HRMS (EI)** [$C_{20}H_{34}Si$] $[M]^+$ calculated: 302.2430, found: 302.2417.

Triethyl(2-phenyloct-7-en-1-yl)silane (5li)

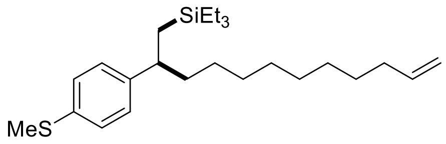

Compound **5li** was obtained as a colorless oil (56.7 mg, Yield: 70%). **^{1}H NMR** (500 MHz, $CDCl_3$) δ 7.20 – 7.16 (m, 2H), 7.11 – 7.07 (m, 2H), 5.81 (ddt, *J* = 16.9, 10.2, 6.7 Hz, 1H), 5.04 – 4.90 (m, 2H), 2.58 (ddd, *J* = 9.0, 7.5, 4.7 Hz, 1H), 2.47 (s, 3H), 2.09 – 1.97 (m, 2H), 1.64 – 1.45 (m, 2H), 1.34 (q, *J* = 7.5 Hz, 2H), 1.28 – 1.14 (m, 8H), 1.13 – 0.99 (m, 2H), 0.95 – 0.85 (m, 2H), 0.84 (t, *J* = 7.9 Hz, 9H), 0.47 – 0.25 (m, 6H). **^{13}C NMR** (126 MHz, $CDCl_3$) δ 145.6, 139.4, 135.0, 128.1, 127.1, 114.2, 41.4, 41.1, 34.0, 29.8, 29.6, 29.6, 29.2, 29.0, 27.9, 20.0, 16.6, 7.5, 3.8. **IR (KBr)**: 3076, 3018, 2927, 2871, 1598, 1491, 1463, 1416, 1240, 1184, 1097, 1011, 967, 824, 756, 724 cm^{-1}. **HRMS (EI)** [$C_{25}H_{44}SSi$] $[M]^+$ calculated: 404.2933, found: 404.2943.

Triethyl((1-phenyl-2,3-dihydro-1*H*-inden-1-yl)methyl)silane (8b)

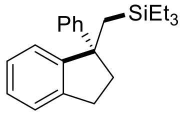

Compound **8b** was obtained as a colorless oil (52.2 mg, Yield: 81%). **^{1}H NMR** (500 MHz, $CDCl_3$) δ 7.38 – 7.34 (m, 3H), 7.28 – 7.12 (m, 6H), 2.98 – 2.86 (m, 2H), 2.77 – 2.68 (m, 1H), 2.31 – 2.18 (m, 1H), 1.84 (d, *J* = 14.8 Hz, 1H), 1.40 (d, *J* = 14.9 Hz, 1H), 0.83 (t, *J* = 7.9 Hz, 9H), 0.43 – 0.27 (m, 6H). **^{13}C NMR** (126 MHz, $CDCl_3$) δ 151.8, 148.8, 142.9, 128.1, 126.6, 126.4, 126.3, 125.7, 124.6, 124.3, 53.9, 41.5, 30.8, 26.4, 7.6, 4.7. **IR (KBr):** 3068, 3016, 2952, 2908, 2874, 1599, 1475, 1458, 1420, 1236, 1016, 777, 743, 724, 699 cm^{-1}. **HRMS (EI)** [$C_{20}H_{25}Si$] $[M-CH_2CH_3]^+$ calculated: 293.1726, found: 293.1728.

((1,5-Diphenyl-2,3-dihydro-1*H*-inden-1-yl)methyl)triethylsilane (8c)

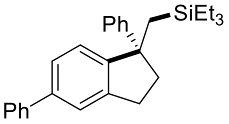

Compound **8c** was obtained as a colorless oil (69.3 mg, Yield: 87%). **^{1}H NMR** (500 MHz, $CDCl_3$) δ 7.62 (dd, *J* = 8.3, 1.3 Hz, 2H), 7.51 – 7.40 (m, 7H), 7.39 – 7.31 (m, 1H), 7.29 (t, *J* = 7.8 Hz, 2H), 7.21 – 7.15 (m, 1H), 2.98 (dd, *J* = 9.0, 4.9 Hz, 2H), 2.81 (dt, *J* = 12.8, 4.9 Hz, 1H), 2.30 (dt, *J* = 12.8, 9.0 Hz, 1H), 1.89 (d, *J* = 14.9 Hz, 1H), 1.43 (d, *J* = 14.8 Hz, 1H), 0.86 (t, *J* = 7.9 Hz, 9H), 0.50 – 0.28 (m, 6H). **^{13}C NMR** (126 MHz, $CDCl_3$) δ 151.3, 148.5, 143.5, 141.7, 139.8, 128.8, 128.2, 127.3, 127.0, 126.4, 125.8, 125.6, 124.5, 123.4, 53.7, 41.5, 30.9, 26.6, 7.6, 4.7. **IR (KBr):** 3058, 3029, 2951, 2873, 1600, 1476, 1414, 1264, 1016, 830, 760, 737, 698 cm^{-1}. **HRMS (EI)** [$C_{28}H_{34}Si$] $[M]^+$ calculated: 398.2430, found: 398.2424.

((1-(Benzo[*d*][1,3]dioxol-5-yl)-5-methoxy-2,3-dihydro-1*H*-inden-1-yl)methyl)triethylsilane (8d)

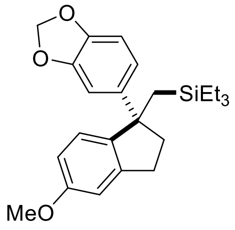

Compound **8d** was obtained as a colorless oil (75.2 mg, Yield: 95%). **^{1}H NMR** (500 MHz, $CDCl_3$) δ 7.10 (d, *J* = 8.1 Hz, 1H), 6.85 (dd, *J* = 5.8, 2.2 Hz, 2H), 6.77 (dd, *J* = 8.2, 2.0 Hz, 1H), 6.73 (dd, *J* = 8.2, 2.5 Hz, 1H), 6.68 (d, *J* = 8.2 Hz, 1H), 5.90 (dd, *J* = 5.0, 1.5 Hz, 2H), 3.82 (s, 3H), 2.88 – 2.75 (m, 2H), 2.65 – 2.55 (m, 1H), 2.22 (dt, *J* = 12.8, 9.0 Hz, 1H), 1.73 (d, *J* = 14.9 Hz, 1H), 1.34 (d, *J* = 14.9 Hz, 1H), 0.85 (t, *J* = 7.9 Hz, 9H), 0.38 (qd, *J* = 7.9, 3.4 Hz, 6H). **^{13}C NMR** (126 MHz, $CDCl_3$) δ 158.8, 153.3, 147.5, 145.4, 143.2, 134.9, 125.1, 119.1, 112.2, 110.1, 107.6, 107.5, 100.9, 55.6, 54.0, 42.4, 29.9, 26.3, 7.6, 4.7. **IR (KBr):** 3055, 2951, 2874, 1608, 1503, 1487, 1465, 1237, 1040, 937, 816, 740, 637 cm^{-1}. **HRMS (EI)** [$C_{24}H_{32}O_3Si$] [M]$^+$ calculated: 396.2121, found: 396.2135.

2,11-Di-*p*-tolyldodecane-1,12-diyl)bis(triethylsilane) (5mc)

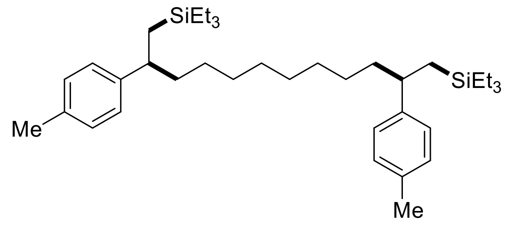

Compound **5mc** was obtained as a colorless oil (102 mg, Yield: 88%). **^{1}H NMR** (500 MHz, $CDCl_3$) δ 7.13 – 7.03 (m, 8H), 2.69 – 2.54 (m, 2H), 2.35 (s, 6H), 1.64 – 1.48 (m, 4H), 1.28 – 1.11 (m, 10H), 1.12 – 1.01 (m, 2H), 0.99 – 0.91 (m, 4H), 0.88 (t, *J* = 8.0 Hz, 18H), 0.50 – 0.29 (m, 12H). **^{13}C NMR** (126 MHz, $CDCl_3$) δ 145.3, 135.1, 128.9, 127.4, 41.5, 41.2, 29.8, 29.7, 27.9, 21.2, 20.1, 7.6, 3.8. **IR (KBr)**: 3053, 3007, 2930, 2852, 1514, 1456, 1415, 1379, 1239, 1181, 1014, 1008, 971, 820, 757, 720 cm^{-1}. **HRMS (EI)** [$C_{36}H_{60}Si_2$] [M-C_4H_6]$^+$ calculated: 548.4234, found: 548.4252.

Biphenyl-4-yltriethylsilane (11)[43]

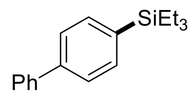

Compound **11** was obtained as a colorless oil (For Aryl bromide: 10.3 mg, Yield: 19%; for Aryl iodide: 13.4 mg, Yield: 25%). **^{1}H NMR** (300 MHz, $CDCl_3$) δ 7.72 – 7.55 (m, 5H), 7.50 – 7.38 (m, 2H), 7.41 – 7.31 (m, 1H), 7.30 – 7.16 (m, 1H), 1.08 – 0.94 (m, 9H), 0.91 – 0.76 (m, 6H). **MS(EI)**: *m/z* 268 [M]$^+$. The chemical shifts were consistent with those reported in the literature.

2-(Biphenyl-4-yl)-4,4,5,5-tetramethyl-1,3,2-dioxaborolane (12)[44]

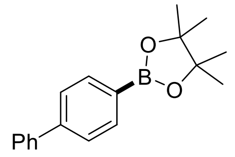

Compound **12** was obtained as a colorless oil (For Aryl bromide: 33 mg, Yield: 59%; for Aryl iodide: 34.3 mg, Yield: 61%). **^{1}H NMR** (300 MHz, $CDCl_3$) δ 7.90 (d, *J* = 7.0 Hz, 2H), 7.72 – 7.58 (m, 4H), 7.51 – 7.41 (m, 2H), 7.38 (d, *J* = 7.1 Hz, 1H), 1.37 (s, 12H). **MS(EI)**: *m/z* 281 [M+H]$^+$. The chemical shifts were consistent with those reported in the literature.

Triethyl(phenethyl)silane (14)[45]

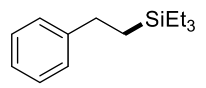

Compound **14** was obtained as a colorless oil (19.4 mg, Yield: 22%). **^{1}H NMR** (300 MHz, $CDCl_3$) δ 7.38 – 7.10 (m, 5H), 2.81 – 2.44 (m, 2H), 1.09 – 0.82 (m, 9H), 0.75 – 0.41 (m, 8H). **MS(EI)**: *m/z* 220 [M]$^+$. The chemical shifts were consistent with those reported in the literature.

(Z)-Triethyl(2-phenylpent-2-en-1-yl)silane (15)

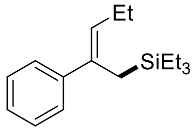

Compound **15** was obtained as a colorless oil (57.7 mg, Yield: 55%). **^{1}H NMR** (500 MHz, $CDCl_3$) δ 7.38 – 7.32 (m, 2H), 7.32 – 7.25 (m, 2H), 7.25 – 7.18 (m, 1H), 5.44 (t, J = 6.9 Hz, 1H), 2.18 (p, J = 7.4 Hz, 2H), 2.01 (s, 2H), 1.08 (t, J = 7.5 Hz, 3H), 0.85 (t, J = 7.9 Hz, 9H), 0.39 (q, J = 7.9 Hz, 6H). **^{13}C NMR** (126 MHz, $CDCl_3$) δ 145.3, 137.6, 128.2, 128.1, 126.8, 126.5, 22.7, 16.2, 14.4, 7.4, 3.9. **IR (KBr)**: 3080, 3021, 2953, 2875, 1599, 1493, 1456, 1416, 1238, 1158, 1016, 974, 823, 767, 698 cm^{-1}. **HRMS (EI)** [$C_{17}H_{28}Si$] [M]$^+$ calculated: 260.1960, found: 260.1968.

Triethyl((1-phenylcyclopropyl)methyl)silane (16)

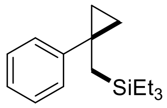

Compound **16** was obtained as a colorless oil (37.5 mg, Yield: 76%). **^{1}H NMR** (300 MHz, $CDCl_3$) δ 7.33 (d, J = 6.7 Hz, 2H), 7.23 (t, J = 7.4 Hz, 2H), 7.14 (t, J = 7.1 Hz, 1H), 1.02 (s, 2H), 0.89 – 0.79 (m, 2H), 0.77 (t, J = 7.9 Hz, 9H), 0.63 (q, J = 3.9 Hz, 2H), 0.31 (q, J = 8.0 Hz, 6H). **^{13}C NMR** (75 MHz, $CDCl_3$) δ 147.0, 128.6, 128.1, 125.9, 24.1, 22.4, 15.3, 7.5, 4.0. **IR (KBr)**: 3074, 3025, 2995, 2952, 2909, 2874, 1601, 1496, 1457, 1415, 1240, 1015, 970, 770, 746, 726, 699 cm^{-1}. **HRMS (EI)** [$C_{16}H_{26}Si$] [M]$^+$ calculated: 246.1804, found: 246.1803.

Triethyl(hex-5-en-1-yl)silane (17)

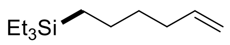

Compound **17** was obtained as a colorless oil (28.9 mg, Yield: 73%). **^{1}H NMR** (500 MHz, $CDCl_3$) δ 5.90 – 5.74 (m, 1H), 5.07 – 4.84 (m, 2H), 2.11 – 1.99 (m, 2H), 1.45 – 1.35 (m, 2H), 1.35 – 1.25 (m, 2H), 0.92 (t, J = 7.9 Hz, 9H), 0.55 – 0.45 (m, 8H). **^{13}C NMR** (126 MHz, $CDCl_3$) δ 139.3, 114.2, 33.6, 33.2, 23.5, 11.3, 7.6, 3.5. **IR (KBr)**: 3078, 3000, 2953, 2924, 2875, 1458, 1415, 1239, 1015, 909, 755, 722 cm^{-1}. **HRMS (EI)** [$C_{10}H_{21}Si$] [M-Et]$^+$ calculated: 169.1413, found: 169.1409.

(4-(Biphenyl-4-yl)-2,4-diphenylbutyl)triethylsilane (18)

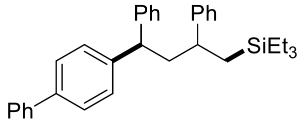

Compound **18** was obtained as a colorless oil. **^{1}H NMR** (500 MHz, $CDCl_3$) δ 7.58 (d, J = 7.6 Hz, 1H), 7.52 (d, J = 8.3 Hz, 2H), 7.47 – 7.34 (m, 3H), 7.33 – 7.06 (m, 13H), 3.74 – 3.61 (m, 1H), 2.62 – 2.49 (m, 1H), 2.49 – 2.38 (m, 1H), 2.38 – 2.28 (m, 1H), 1.03 – 0.92 (m, 2H), 0.74 (t, J = 8.0 Hz, 9H), 0.34 – 0.14 (m, 6H). **^{13}C NMR** (75 MHz, $CDCl_3$) δ 147.3, 145.6, 144.8, 144.1, 143.4, 141.1, 139.1, 128.8, 128.5, 128.4, 128.1, 127.8, 127.7, 127.2, 127.1, 126.2, 48.7, 46.9, 39.2, 20.4, 7.5, 3.7. **IR (KBr)**: 3060, 3027, 2951, 2872, 1601, 1488, 1453, 1410, 1239, 1008, 908, 761, 698 cm^{-1}. **HRMS (EI)** [$C_{34}H_{40}Si$] [M]$^+$ calculated: 476.2899, found: 476.2892.

Hexaethyldisilane[46]

Title compound was obtained as a colorless oil (19.3 mg, Yield: 21%). **^{1}H NMR** (500 MHz, $CDCl_3$) δ 0.97 (t, J = 7.9 Hz, 2H), 0.65 (q, J = 7.9 Hz, 1H). **^{13}C NMR** (126 MHz, $CDCl_3$) δ 8.5, 4.2. **MS(EI)**: *m/z* 230 [M]$^+$. The chemical shifts were consistent with those reported in the literature.

9. NMR Spectra (^{1}H NMR, ^{13}C NMR and ^{19}F NMR)

4-(Benzyloxy)-4'-fluoro-1,1'-biphenyl (1i)

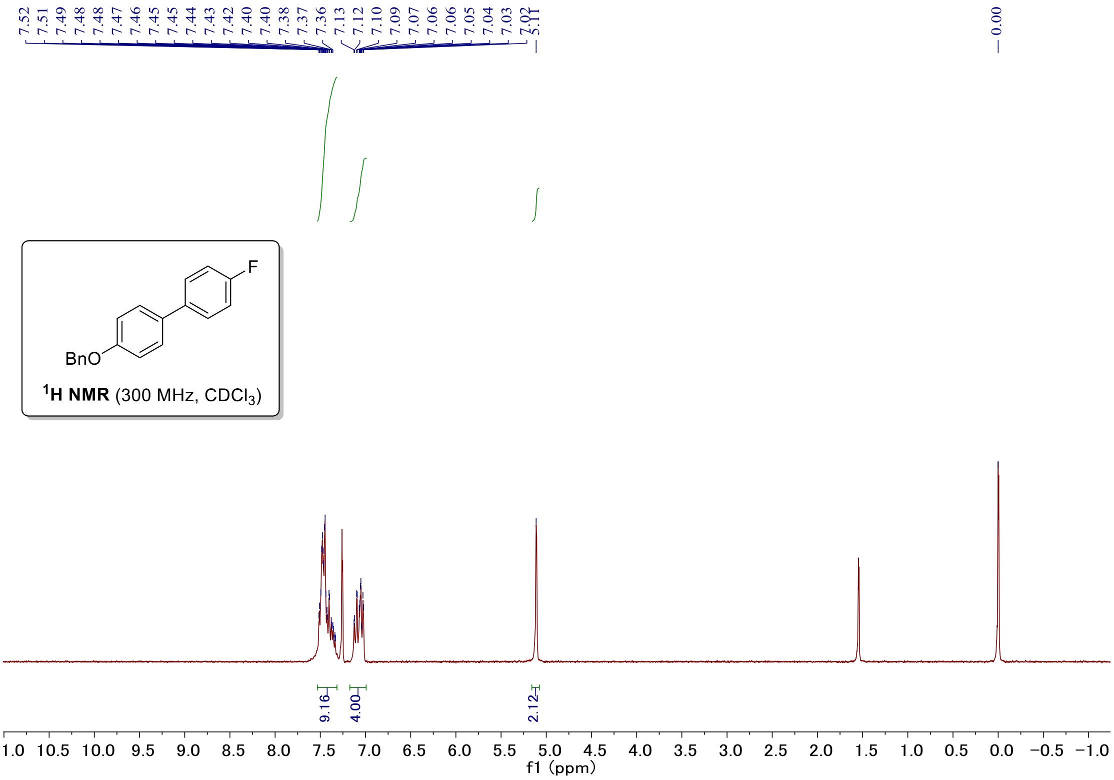

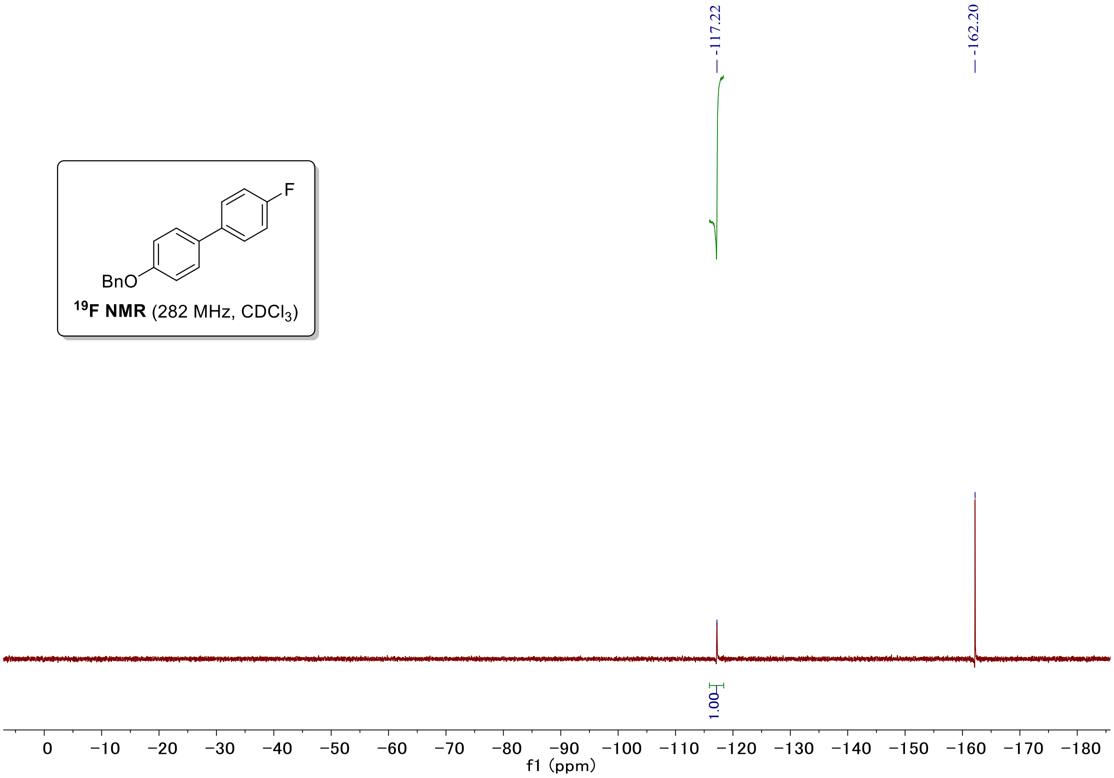

4-Fluoro-3'-(trifluoromethyl)-1,1'-biphenyl (1k)

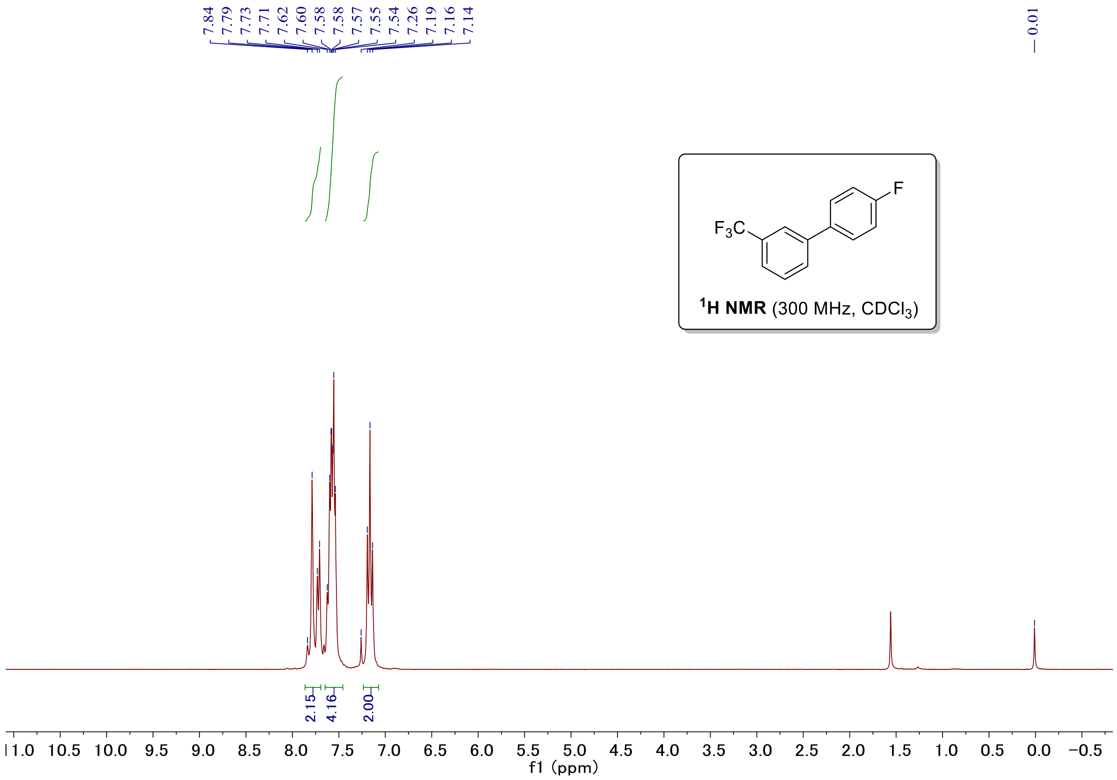

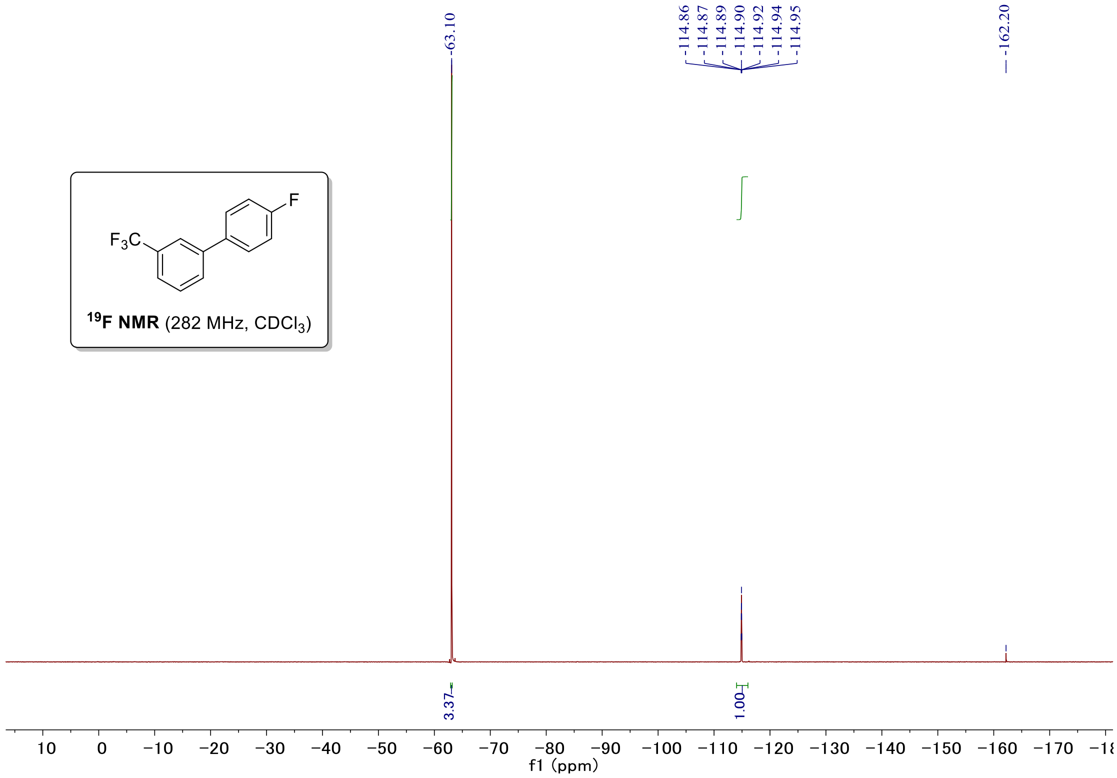

4-Fluoro-4'-(trifluoromethyl)-1,1'-biphenyl (1l)

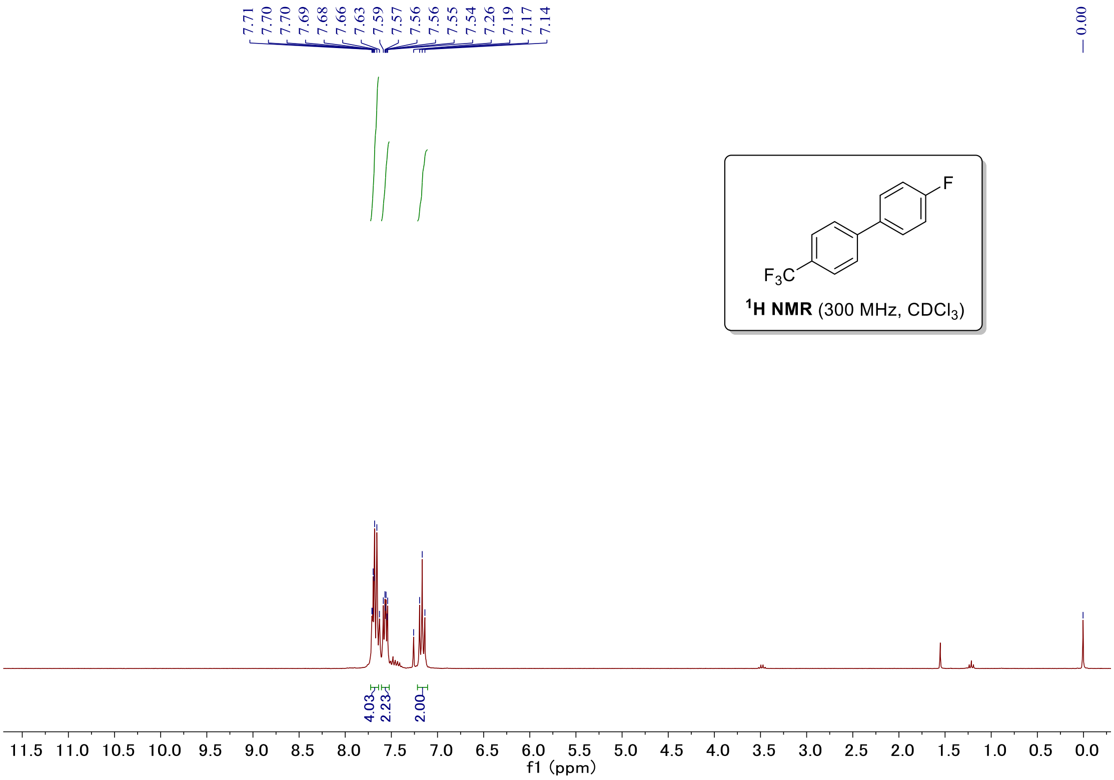

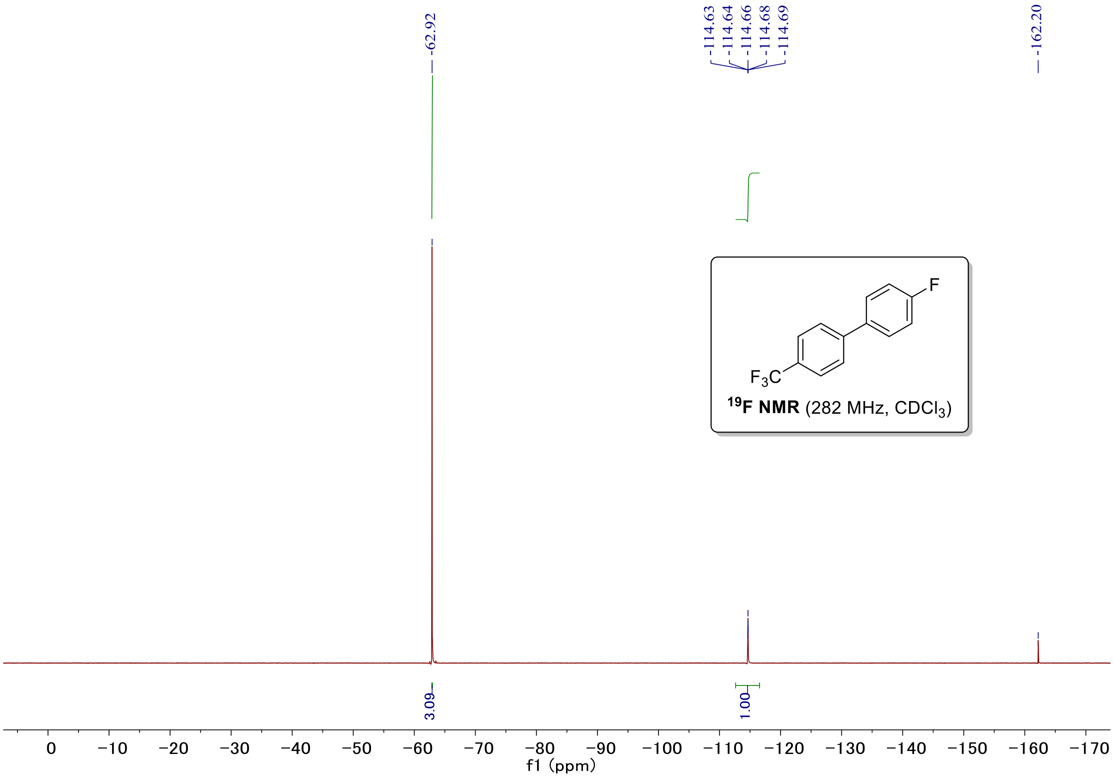

4-Chloro-4'-fluoro-1,1'-biphenyl (1m)

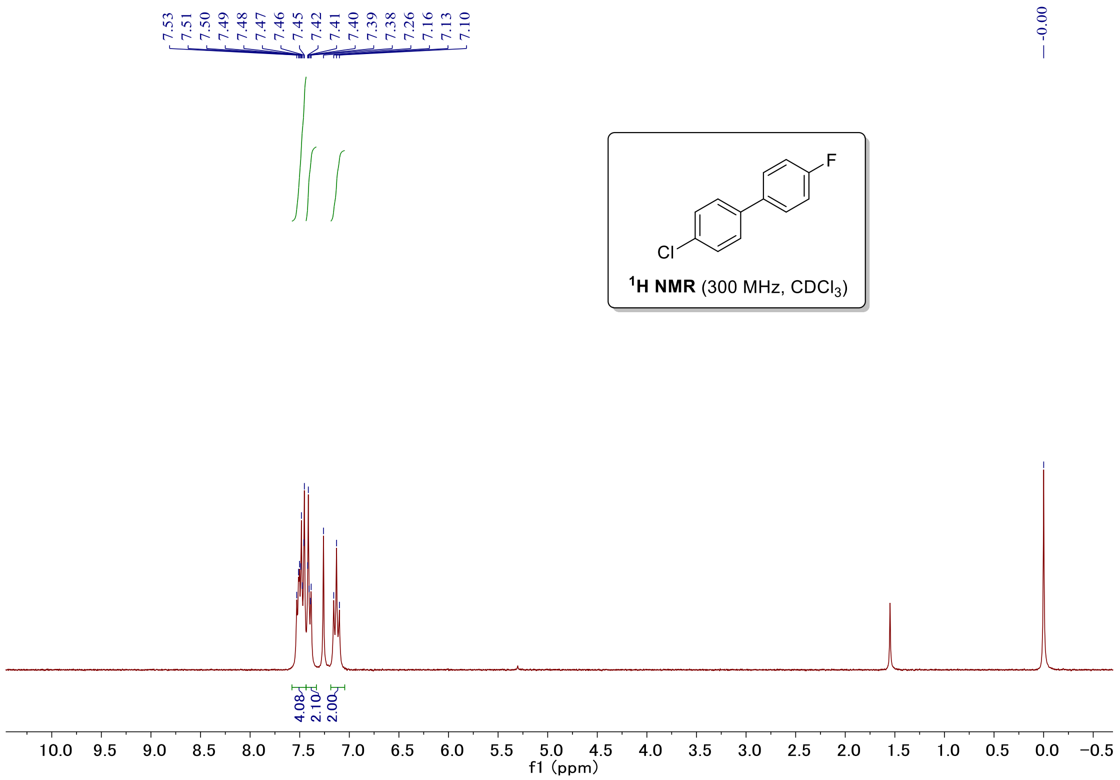

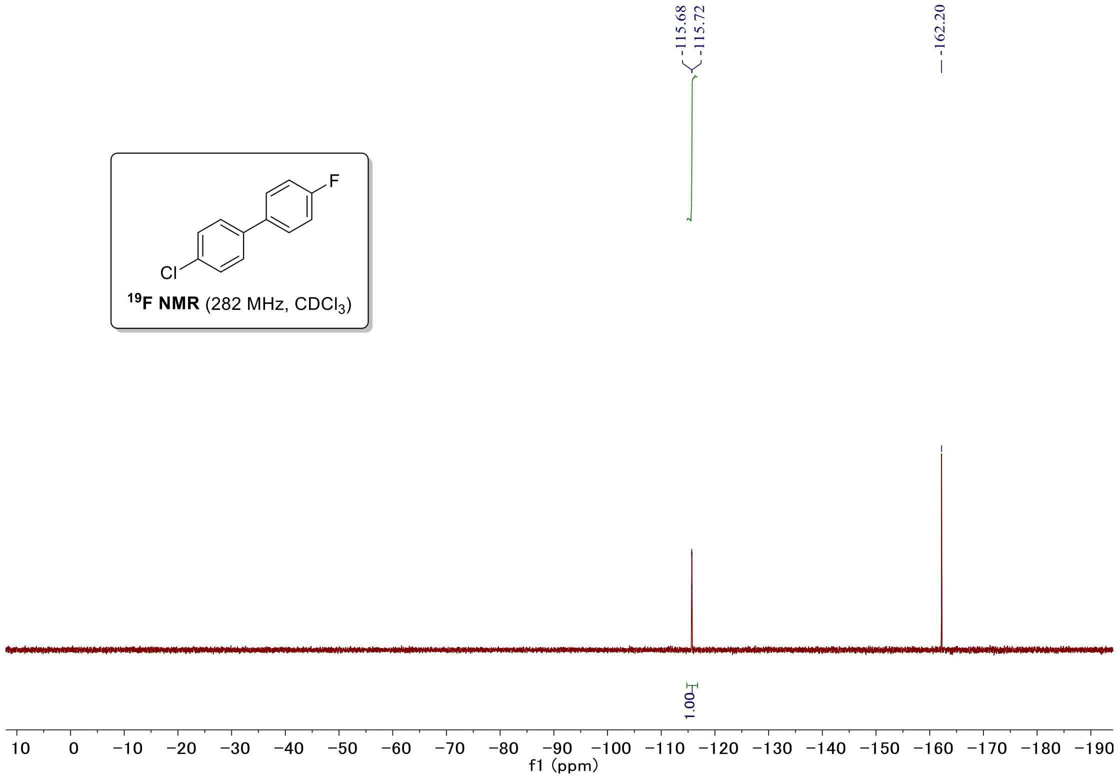

6-Fluoro-1-methyl-*1H*-indole (1n)

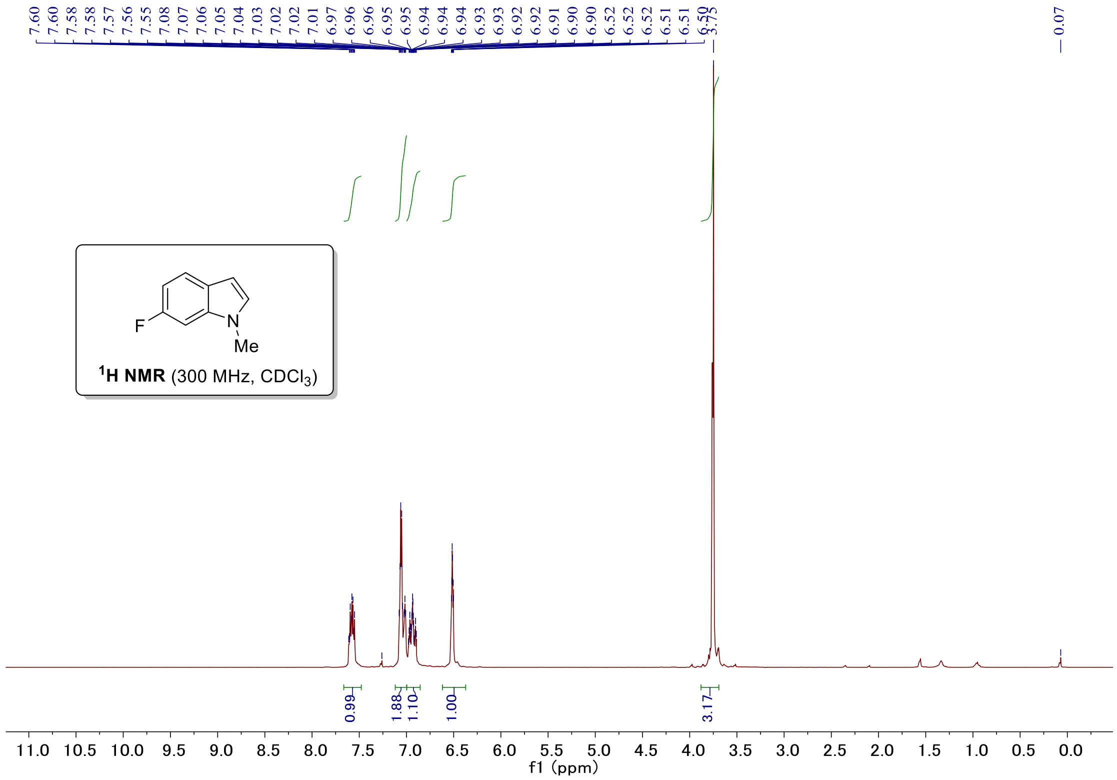

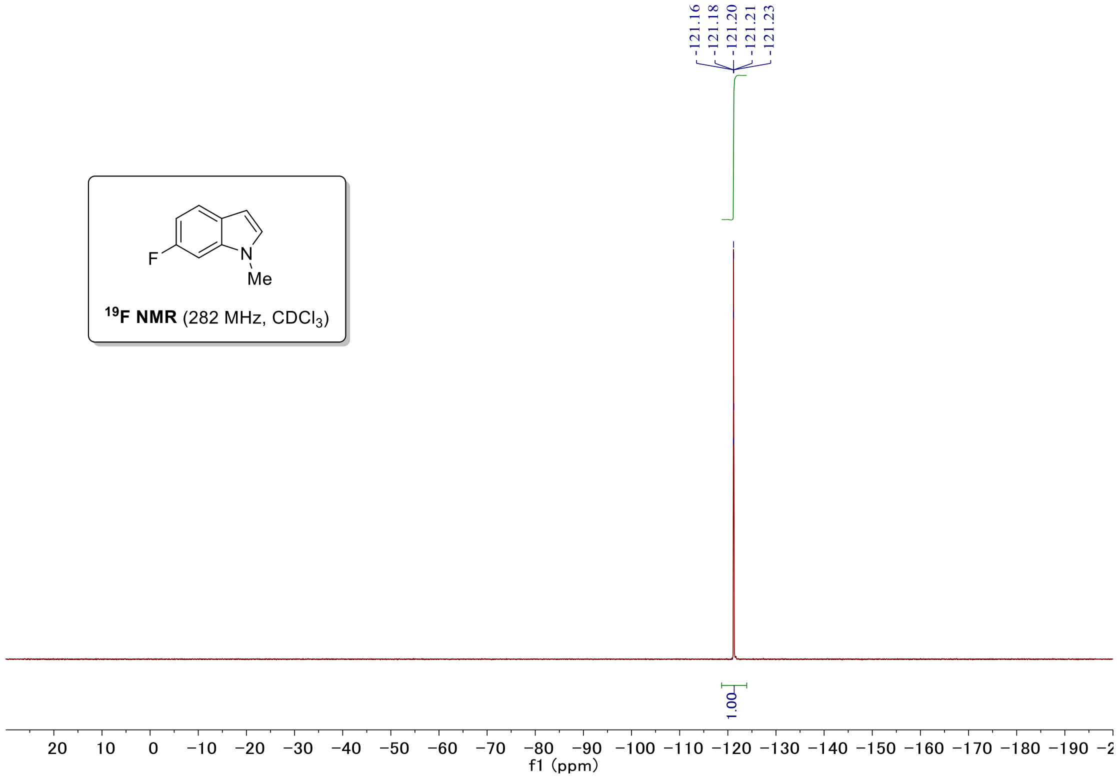

4-(4-Fluorophenyl)pyridine (1p)

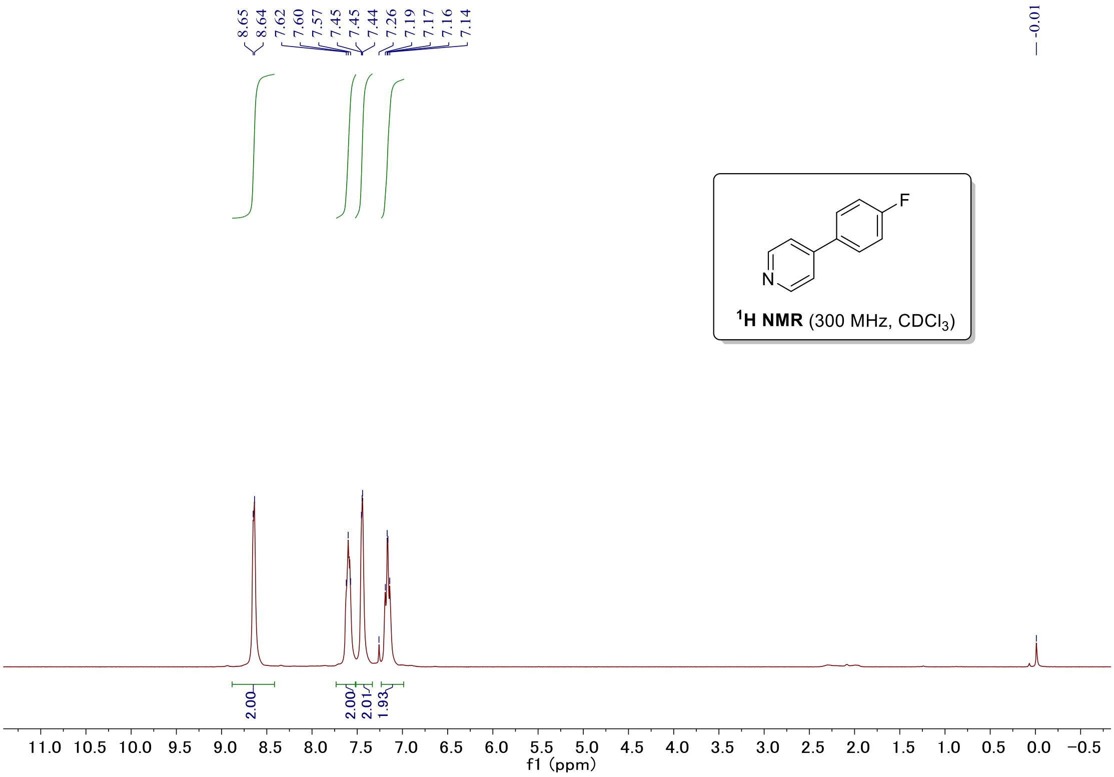

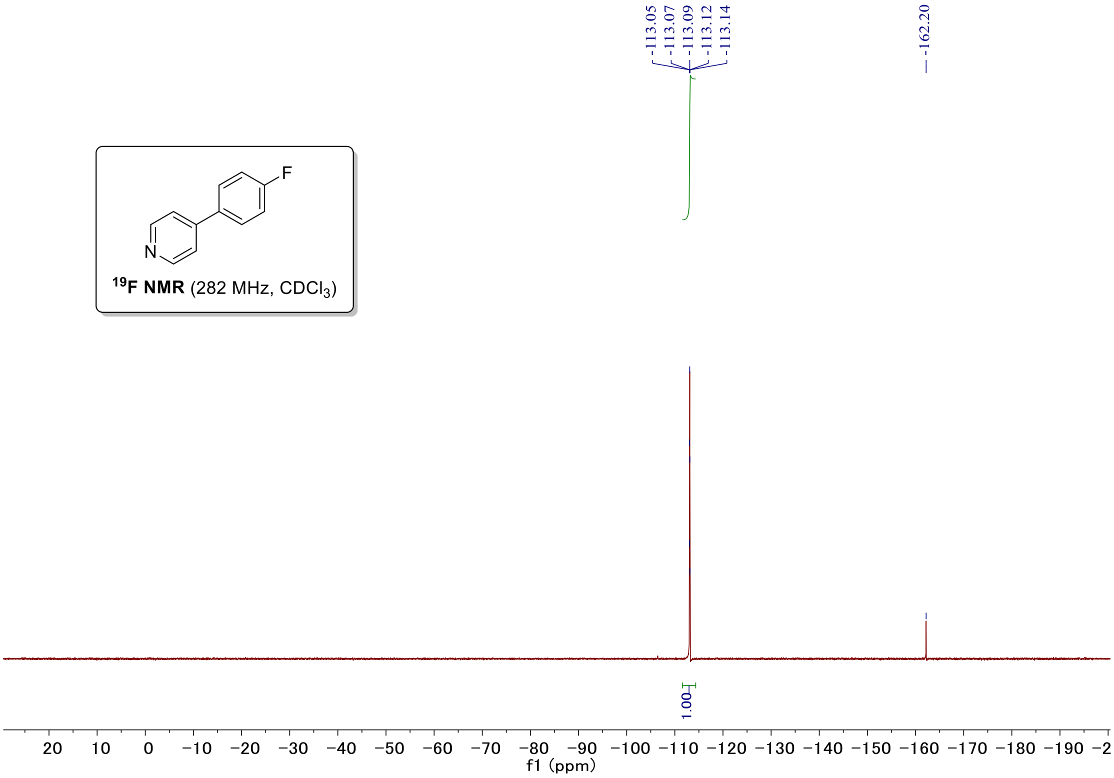

5-Fluoro-2-phenylpyridine (1q)

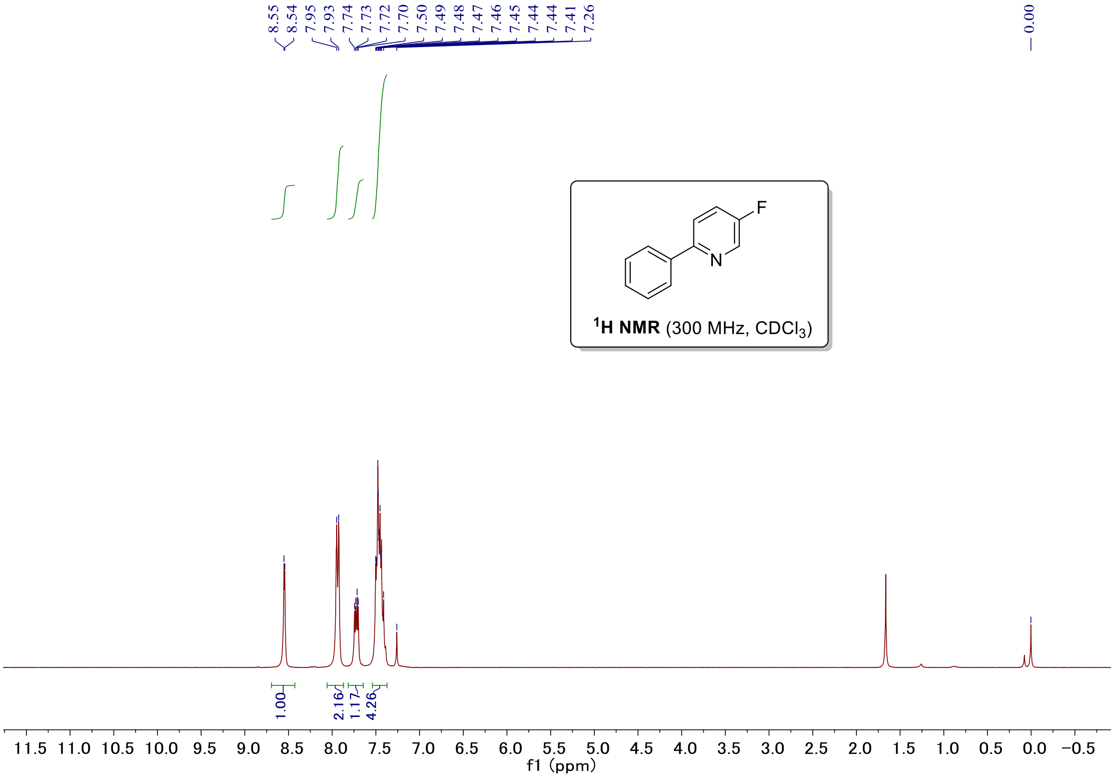

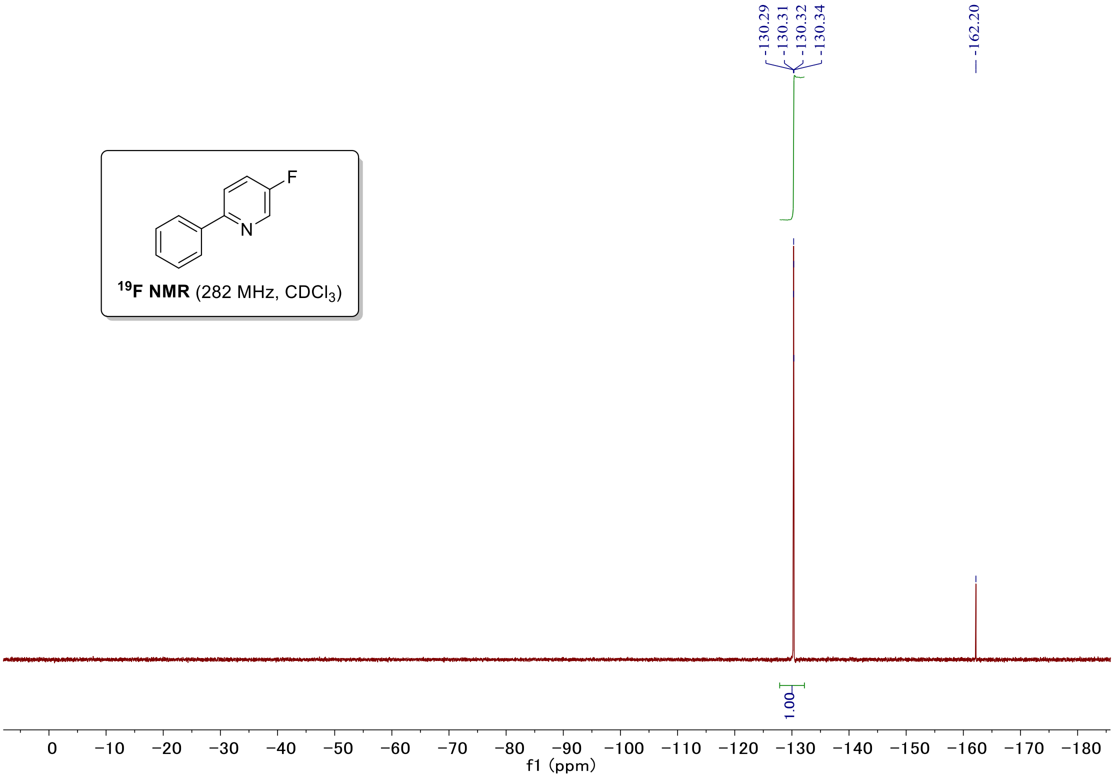

2-Fluoro-5-phenylpyridine (1r)

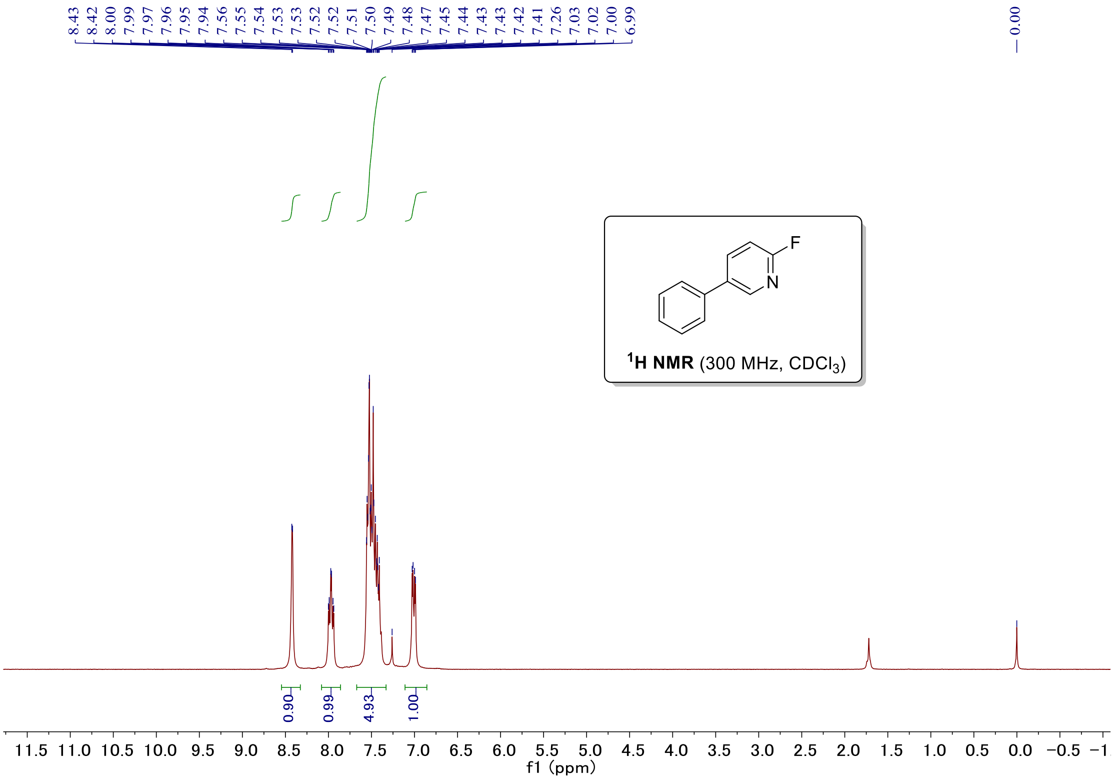

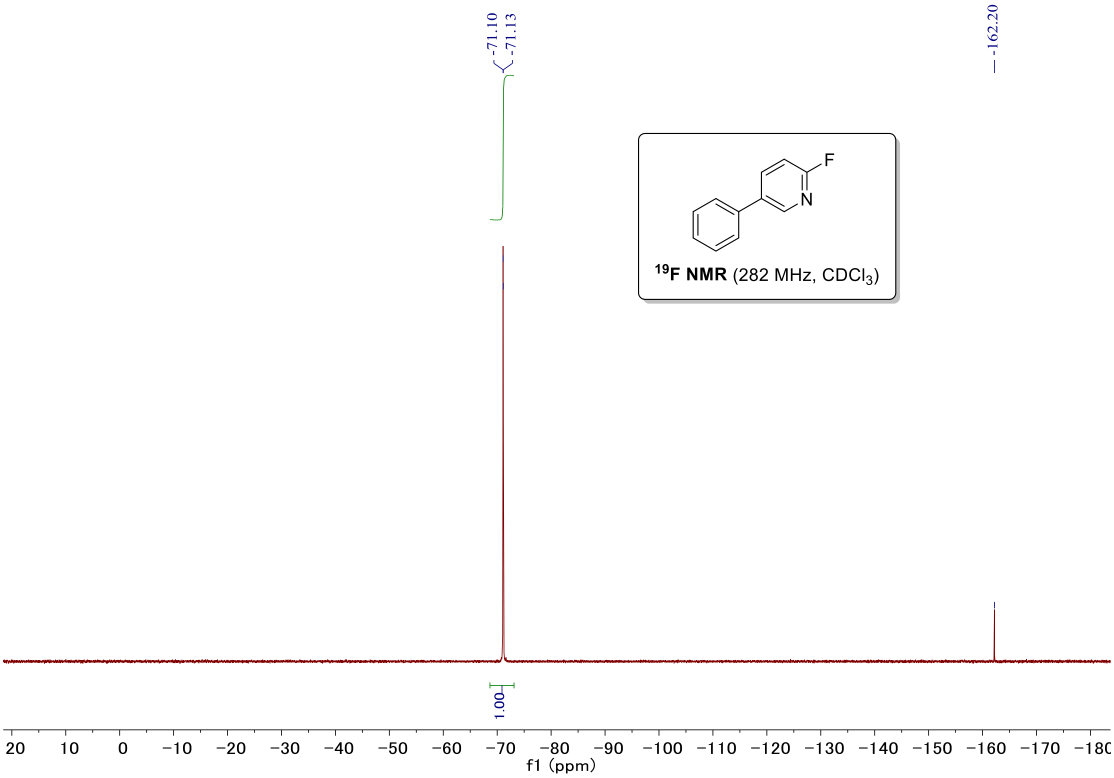

3-(4-Fluorophenyl)benzofuran (1u)

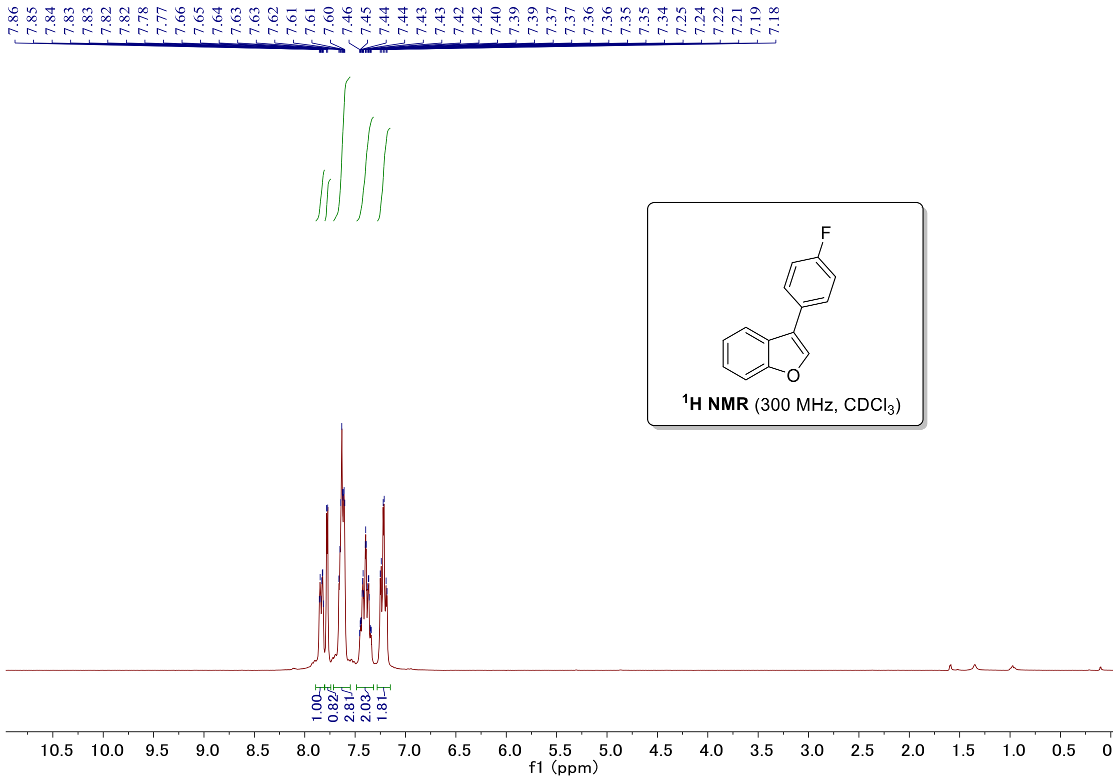

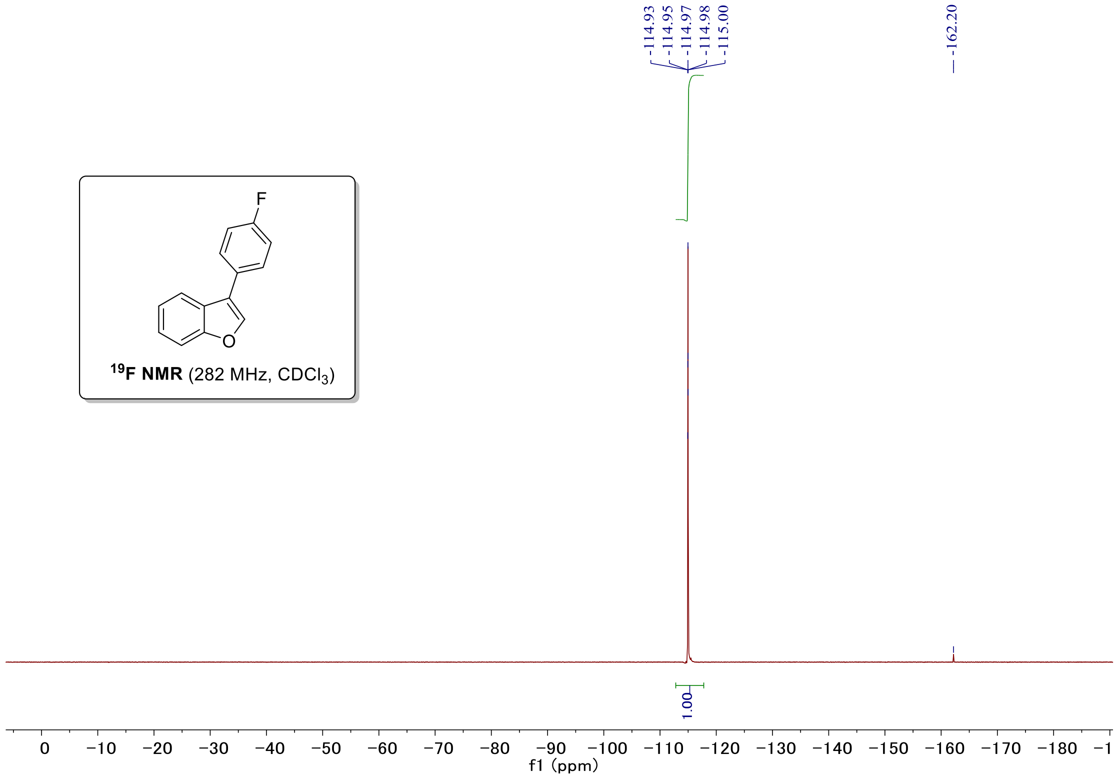

1-Fluoro-3-(3-methoxypropyl)benzene (1v)

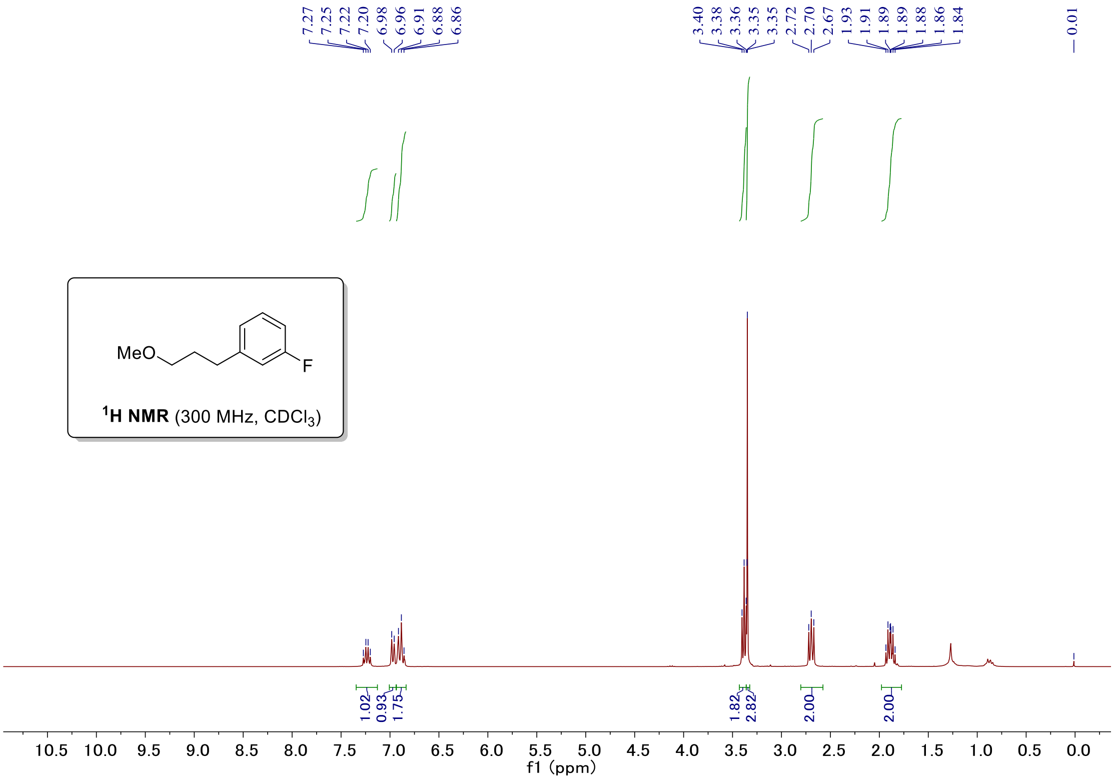

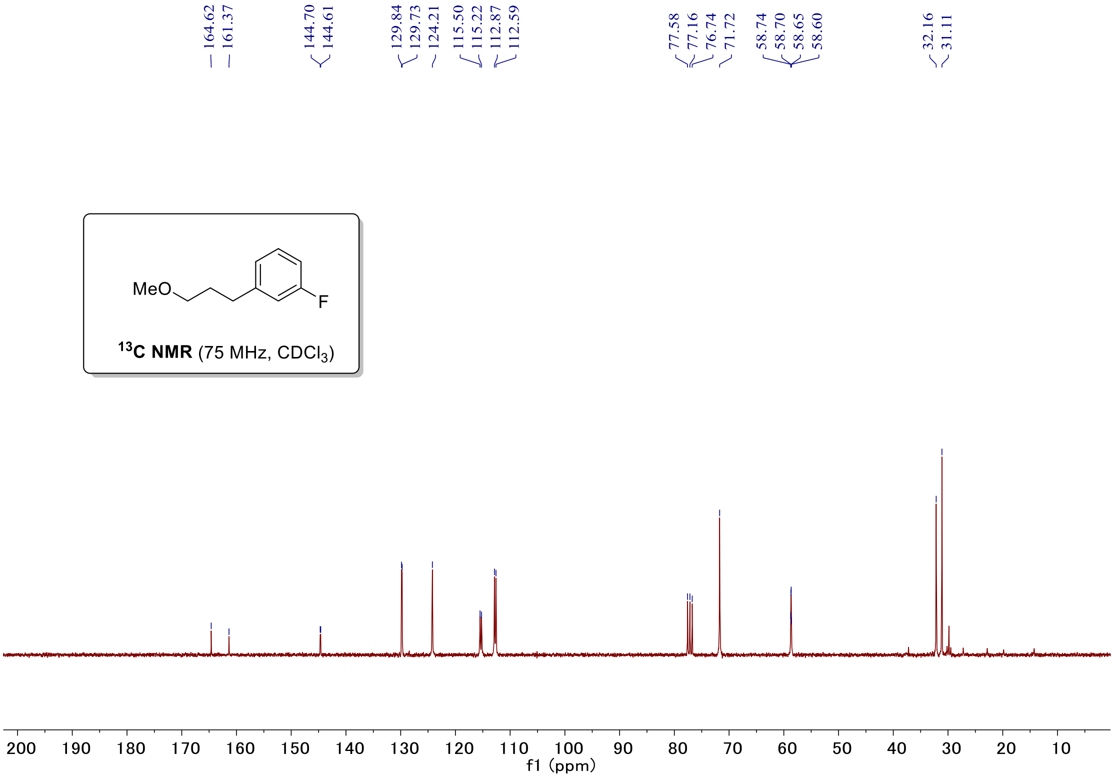

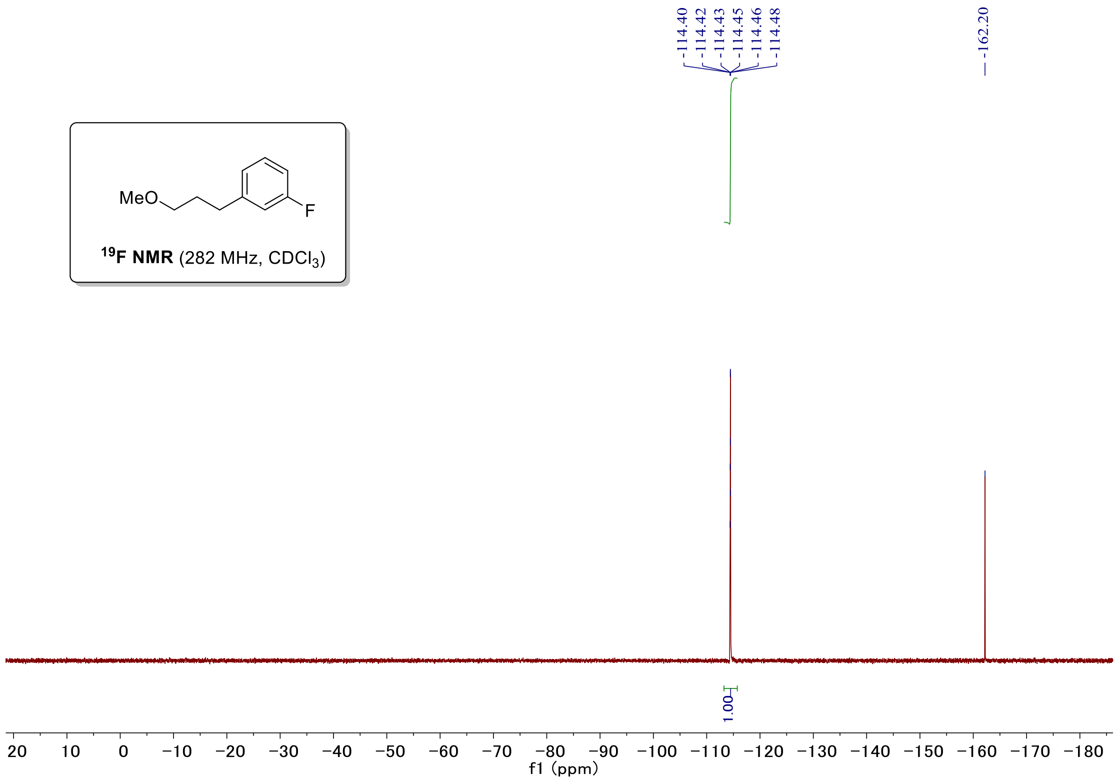

6-((4-Fluorobenzyl)oxy)-2,5,7,8-tetramethyl-2-(4,8,12-trimethyltridecyl)chromane (1w)

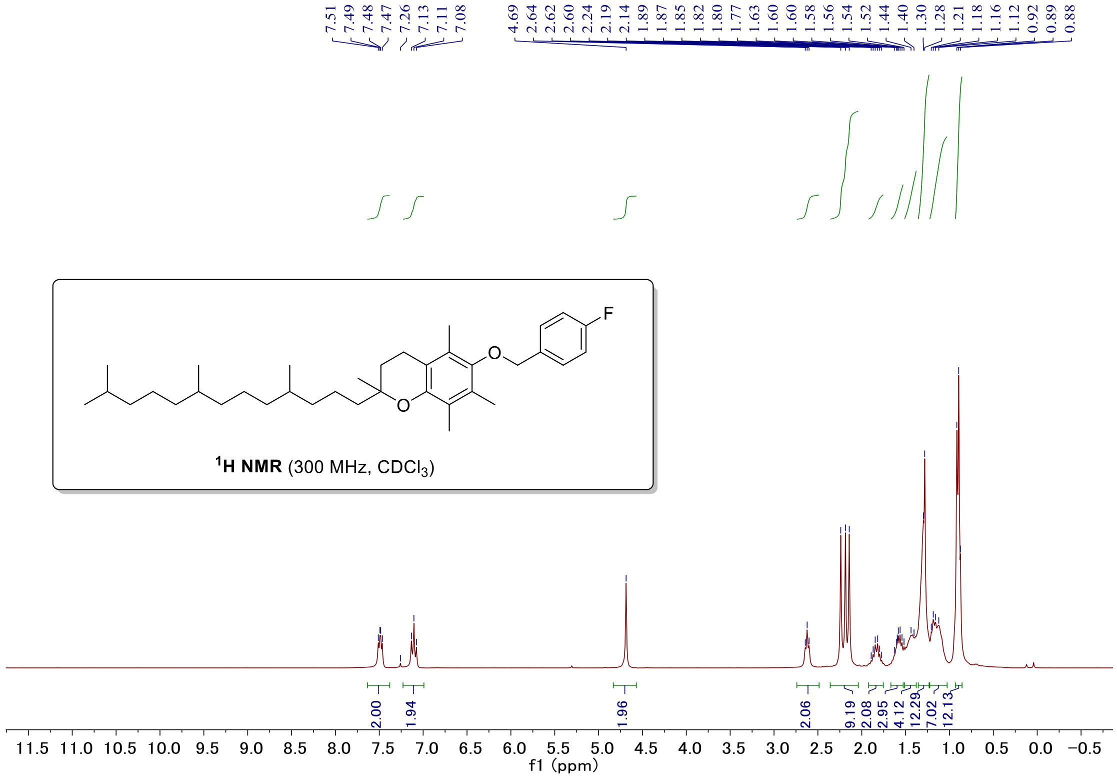

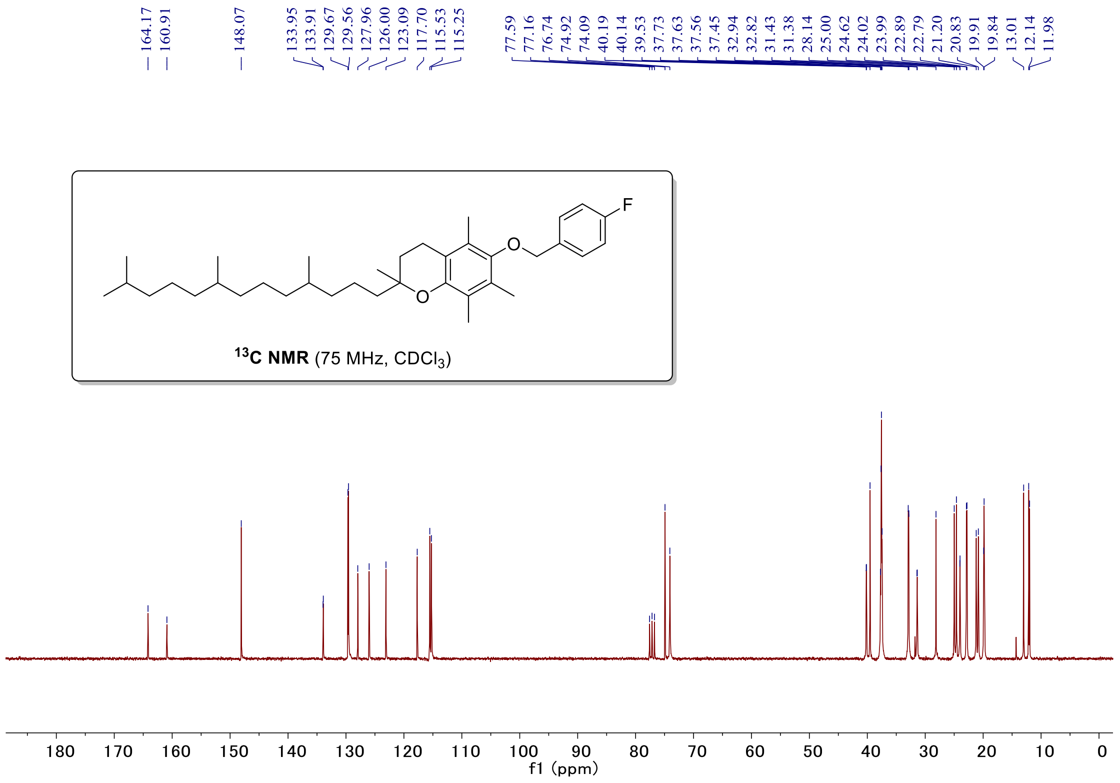
164.17
160.91
148.07
133.95
133.91
129.67
129.56
127.96
126.00
123.09
117.70
115.53
115.25
77.59
77.16
76.74
74.92
74.09
40.19
40.14
39.53
37.73
37.63
37.56
37.45
32.94
32.82
31.43
31.38
28.14
25.00
24.62
24.02
23.99
22.89
22.79
21.20
20.83
19.91
19.84
13.01
12.14
11.98
F
O
O
13C NMR (75 MHz, CDCl3)
f1 (ppm)

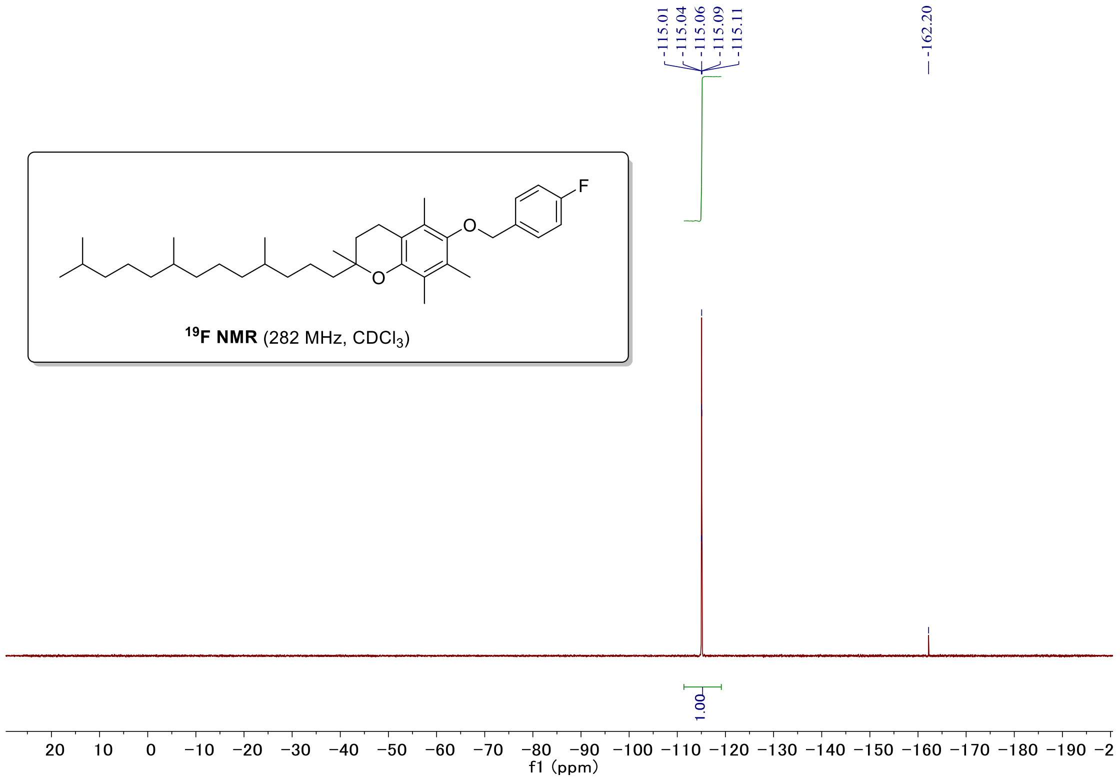
-115.01
-115.04
-115.06
-115.09
-115.11
-162.20
F
O
O
19F NMR (282 MHz, CDCl3)
1.00
f1 (ppm)

1-Fluoro-4-((((*2S*,*5R*)-2-isopropyl-5-methylcyclohexyl)oxy)methyl)benzene (1x)

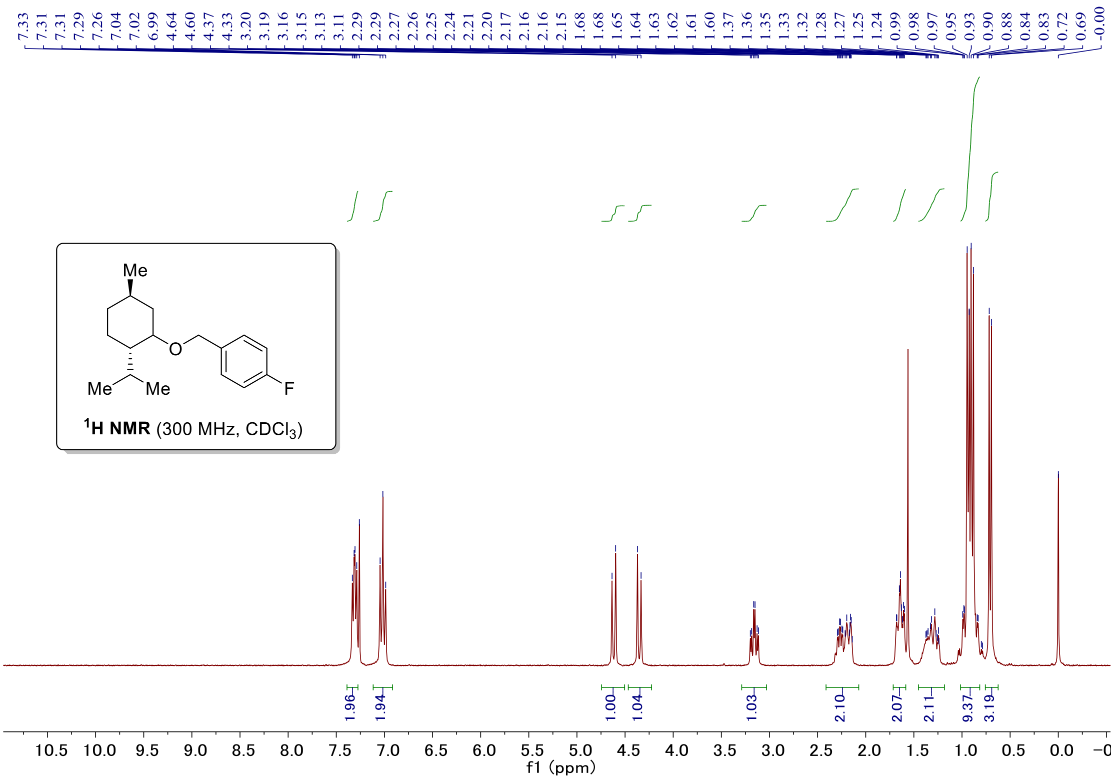

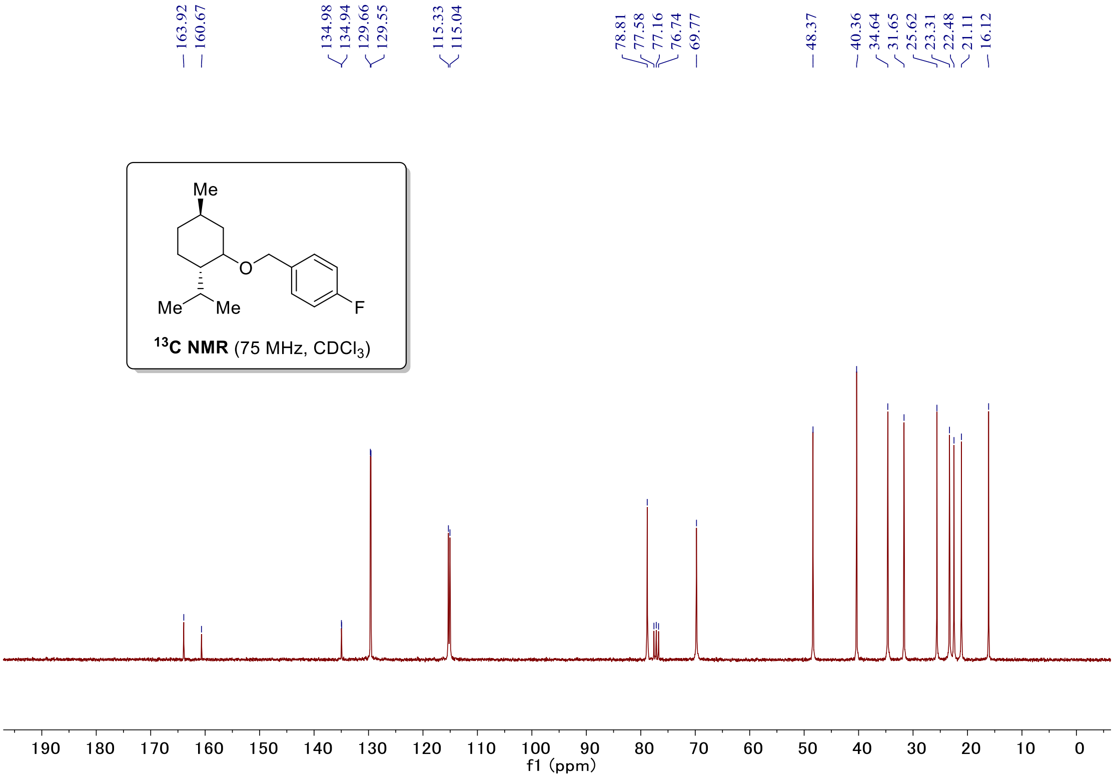

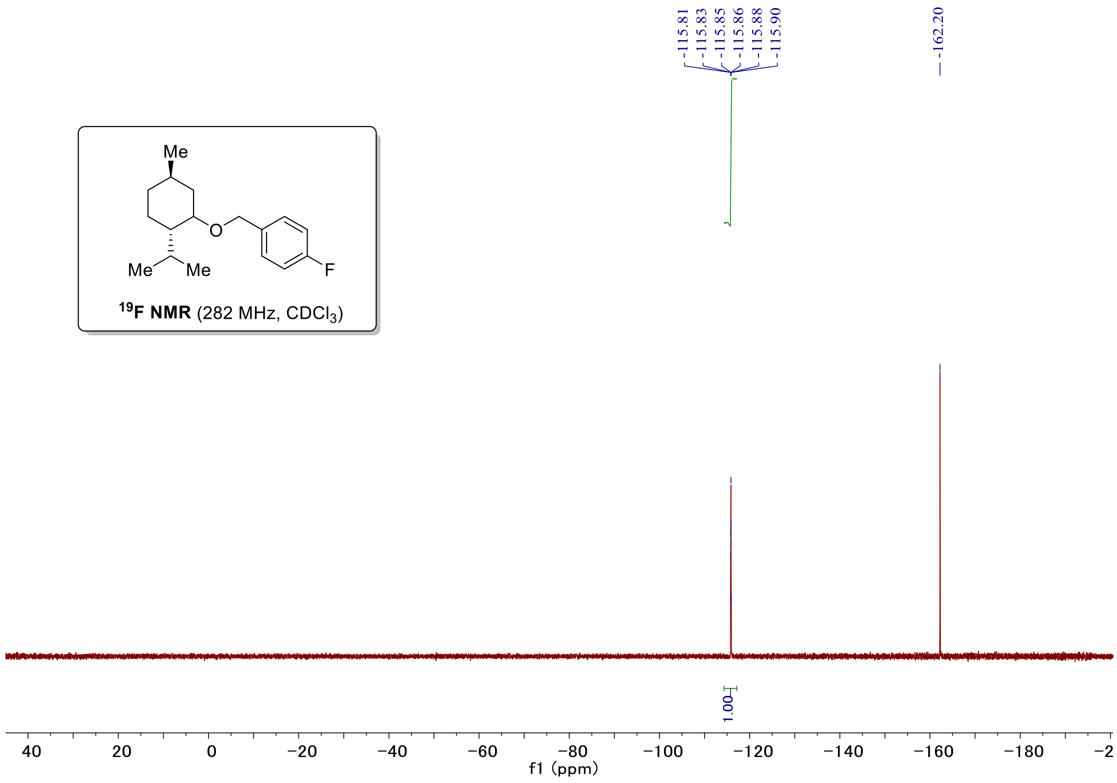

3-Fluoroestrone derivative (1y)

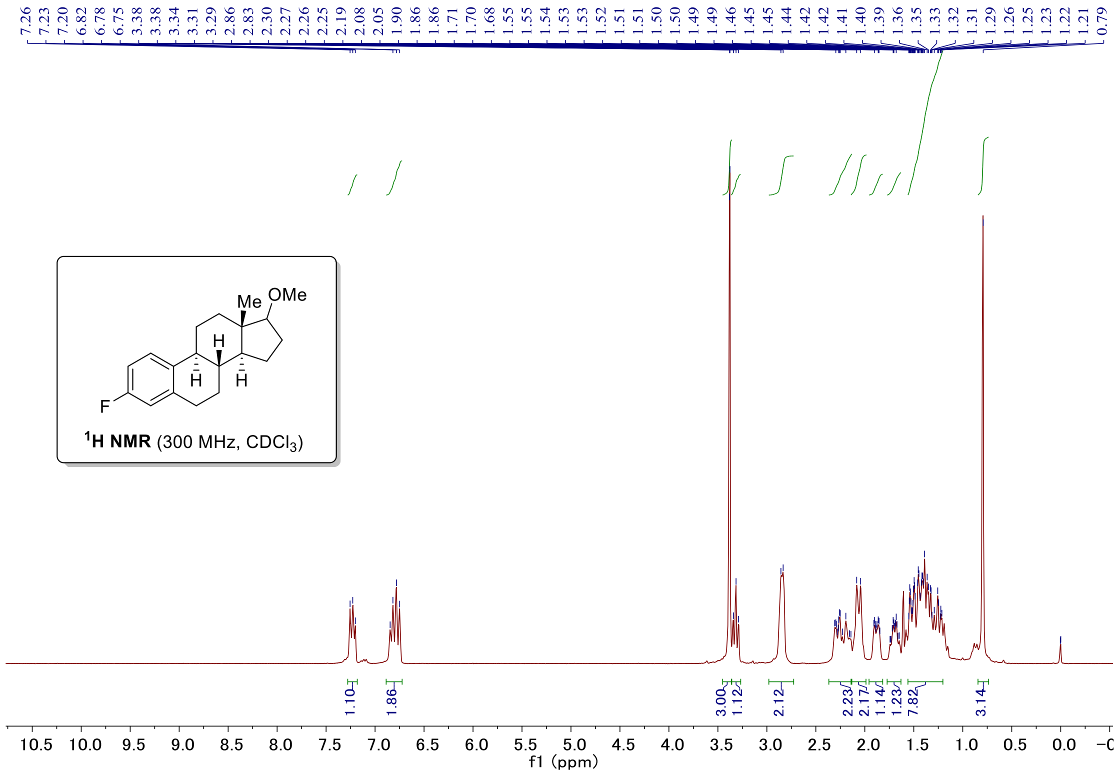

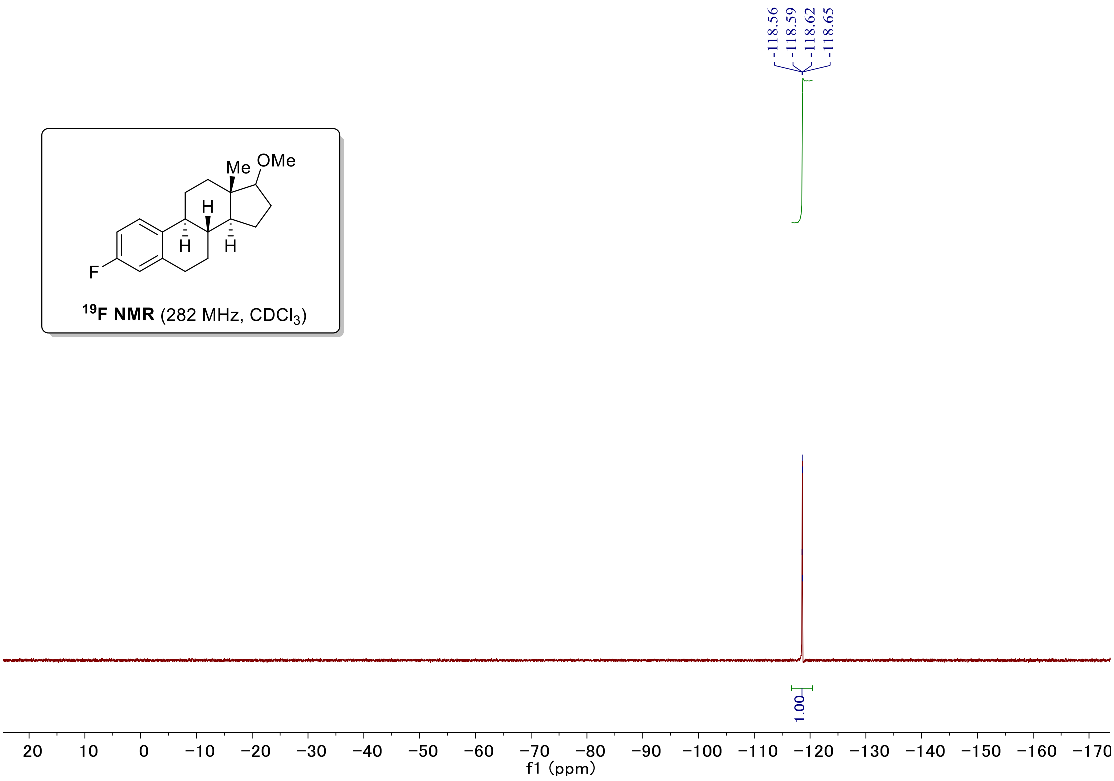

1-Fluorodecane (2e)

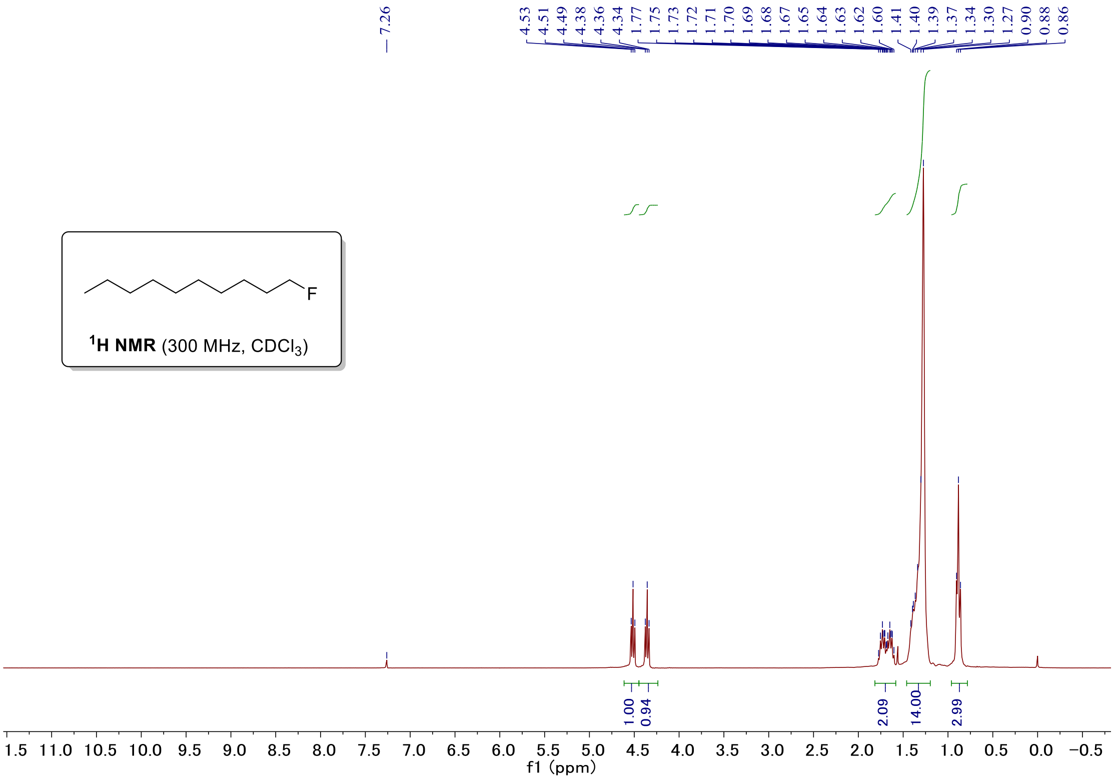

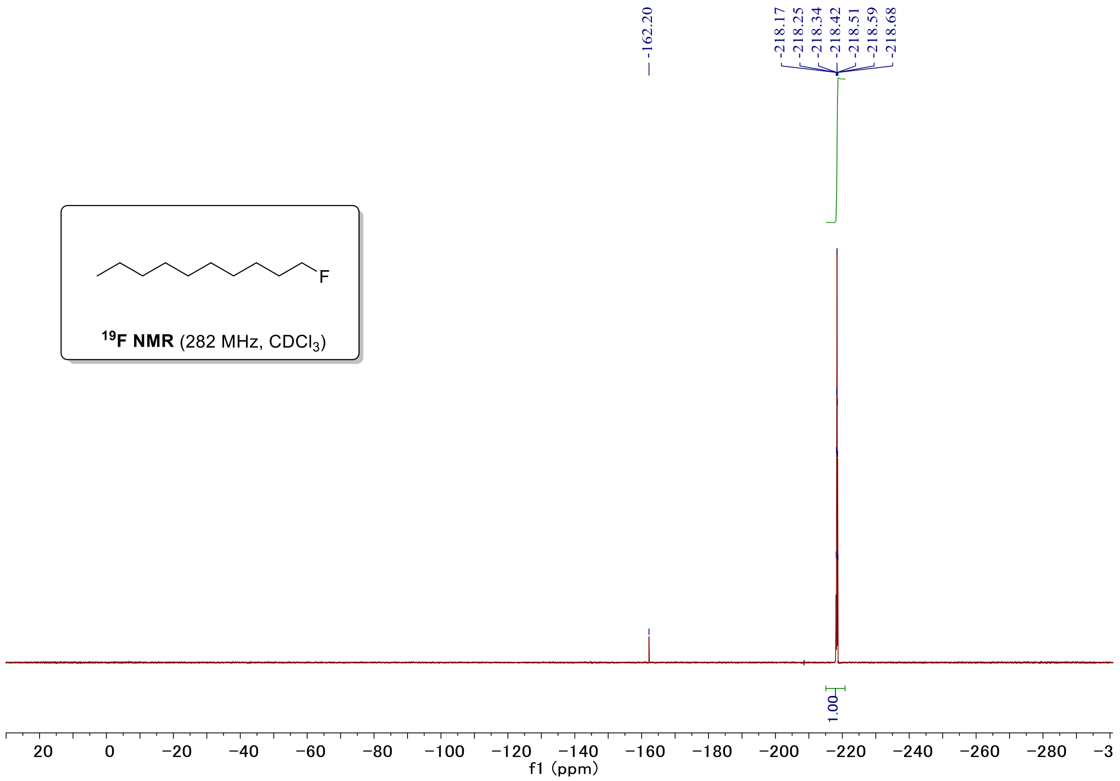

1-Fluoro-3-phenylpropane (2f)

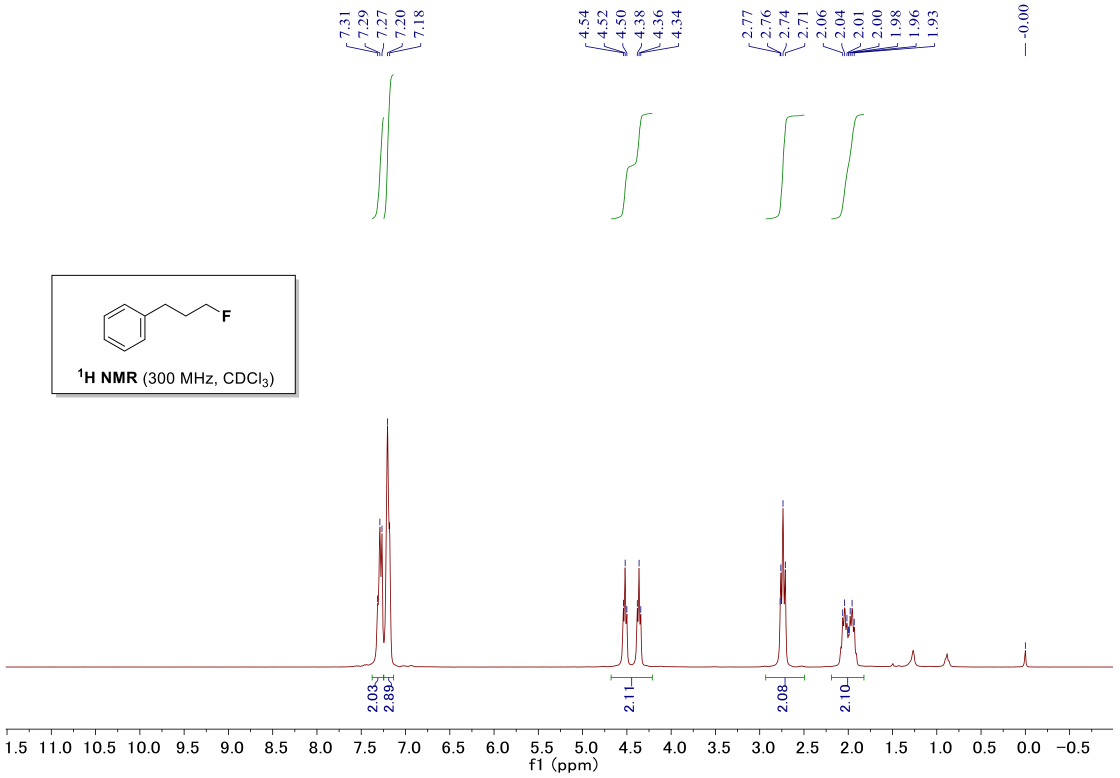

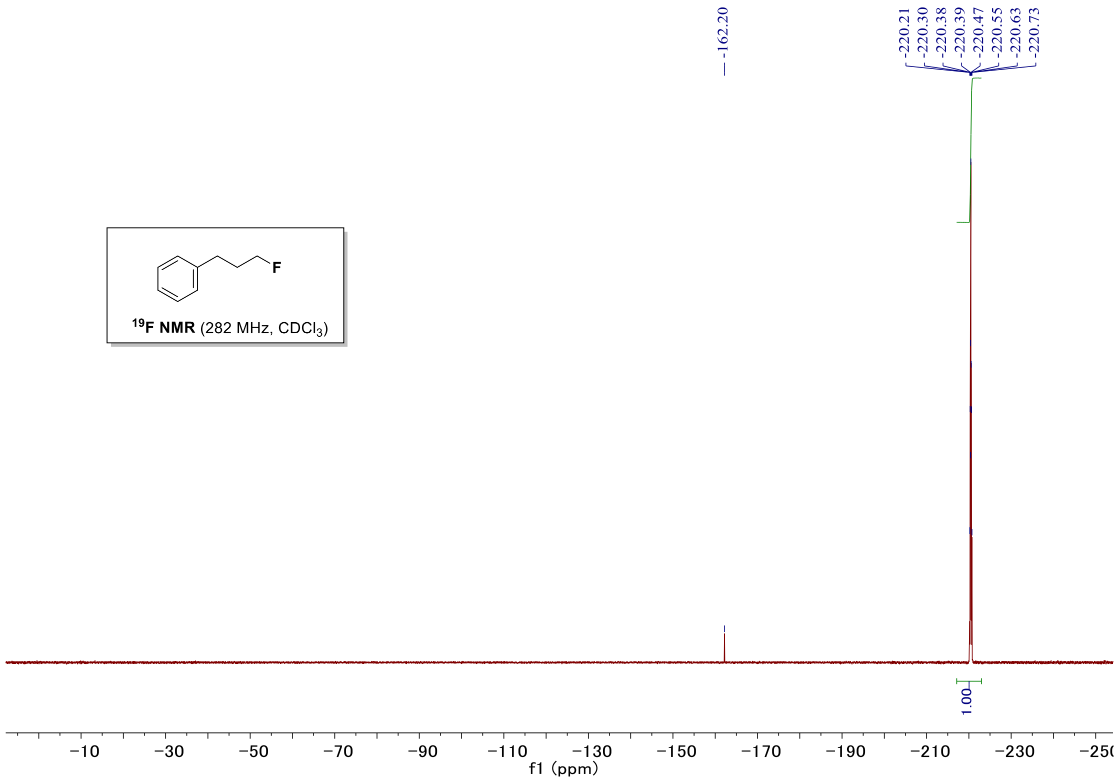

2-Fluoroethylbenzene (2g)

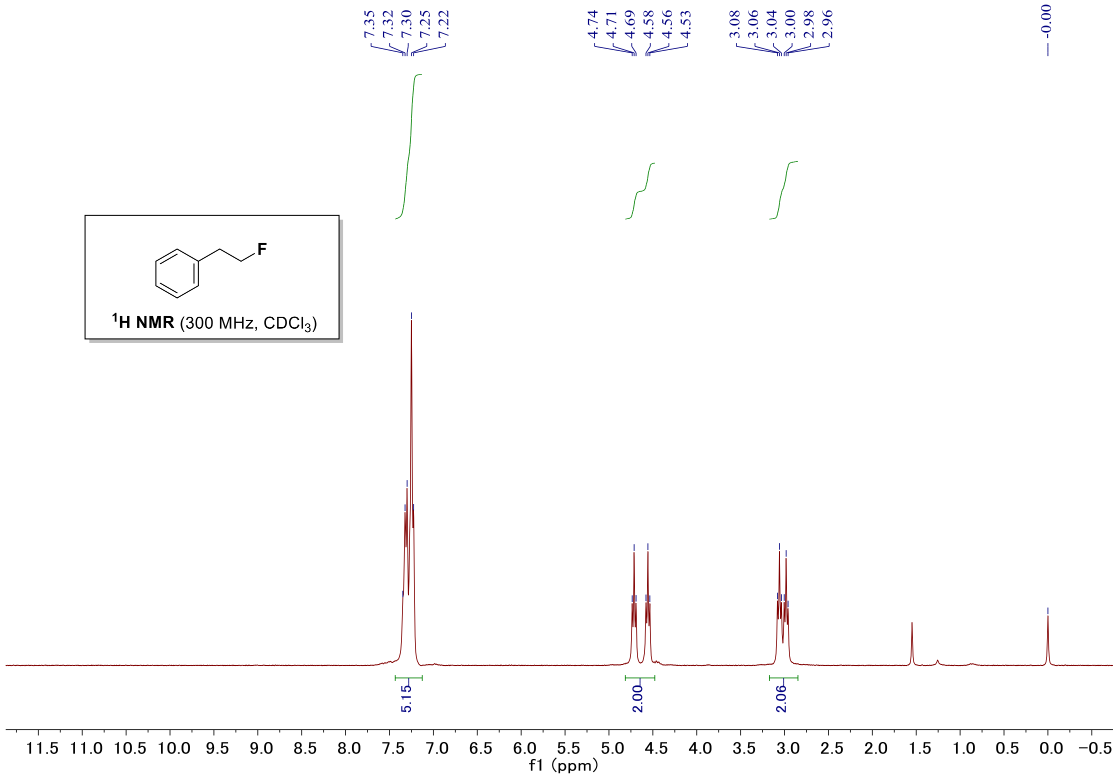

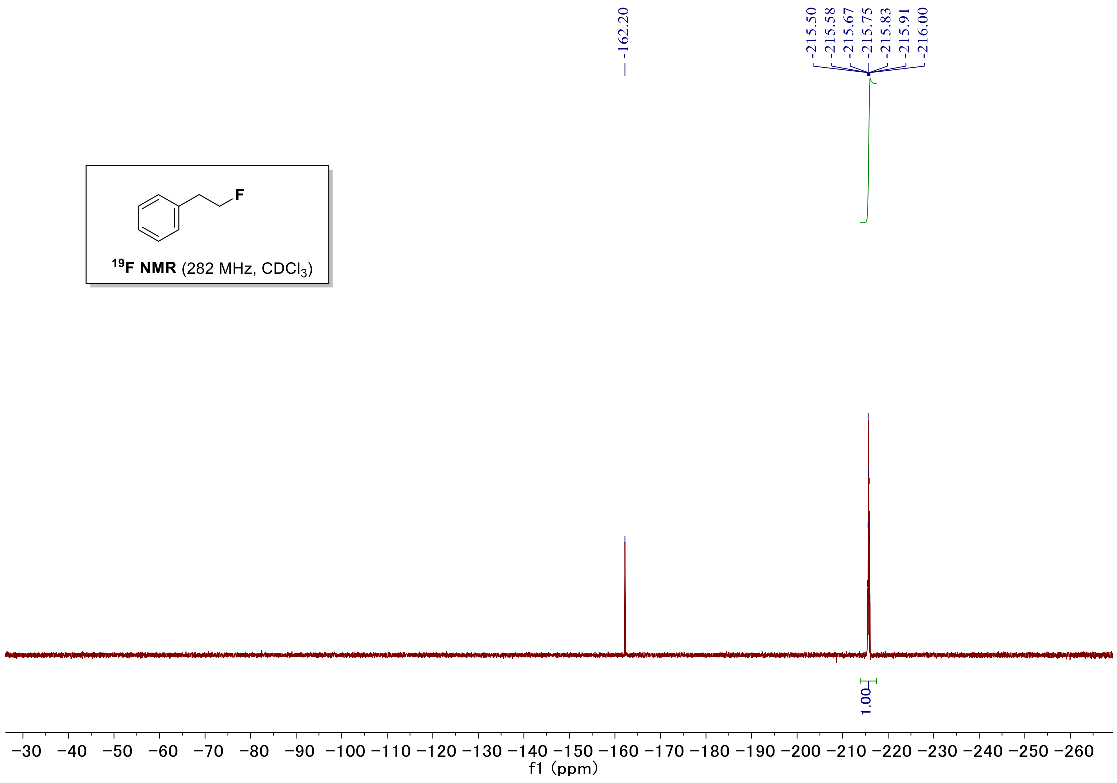

6-Fluorohex-1-ene (2k)

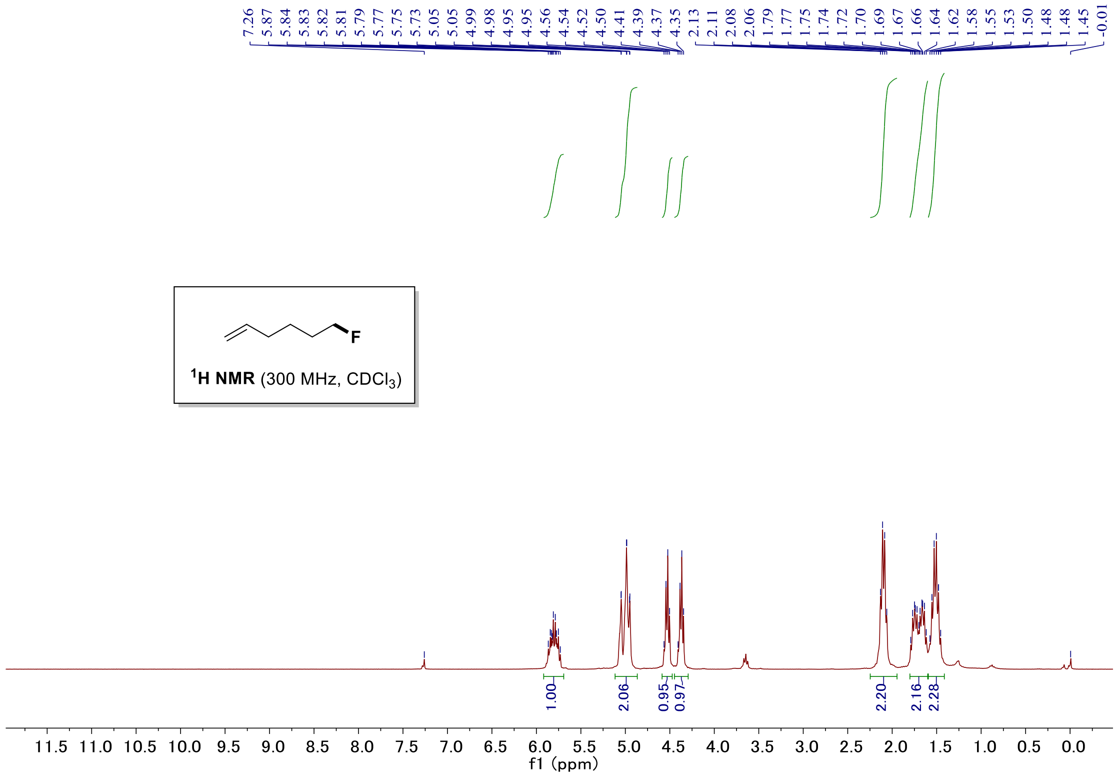

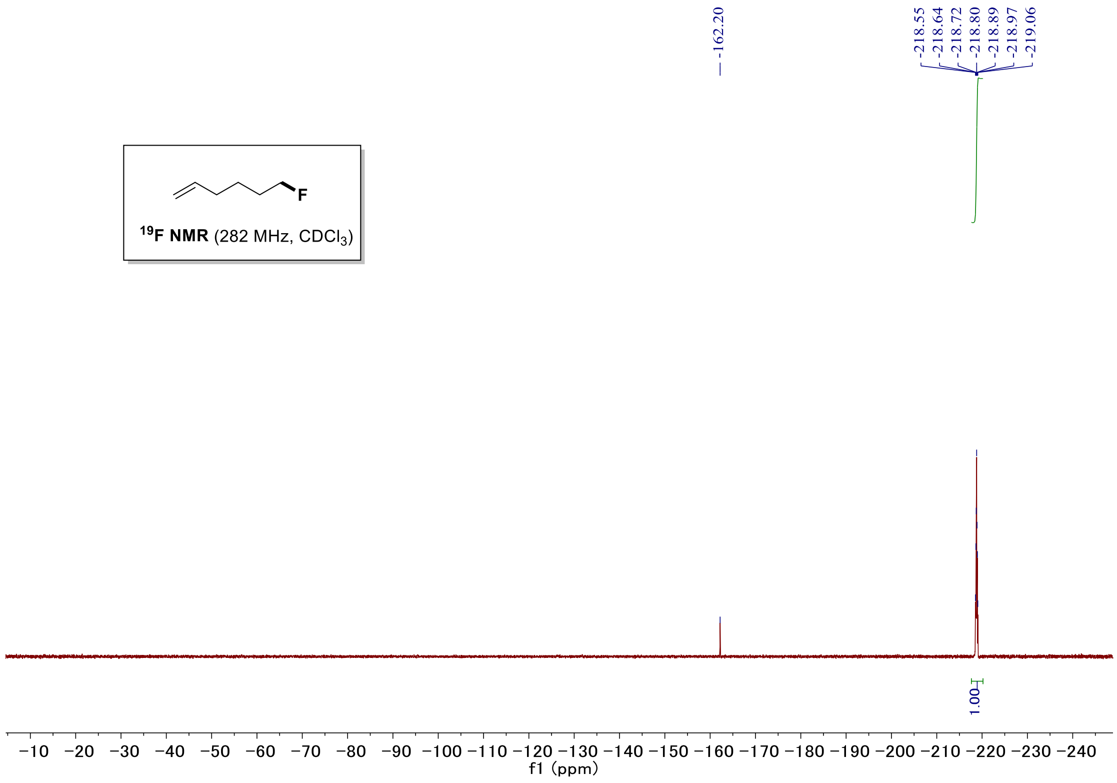

10-Fluorodec-1-ene (2l)

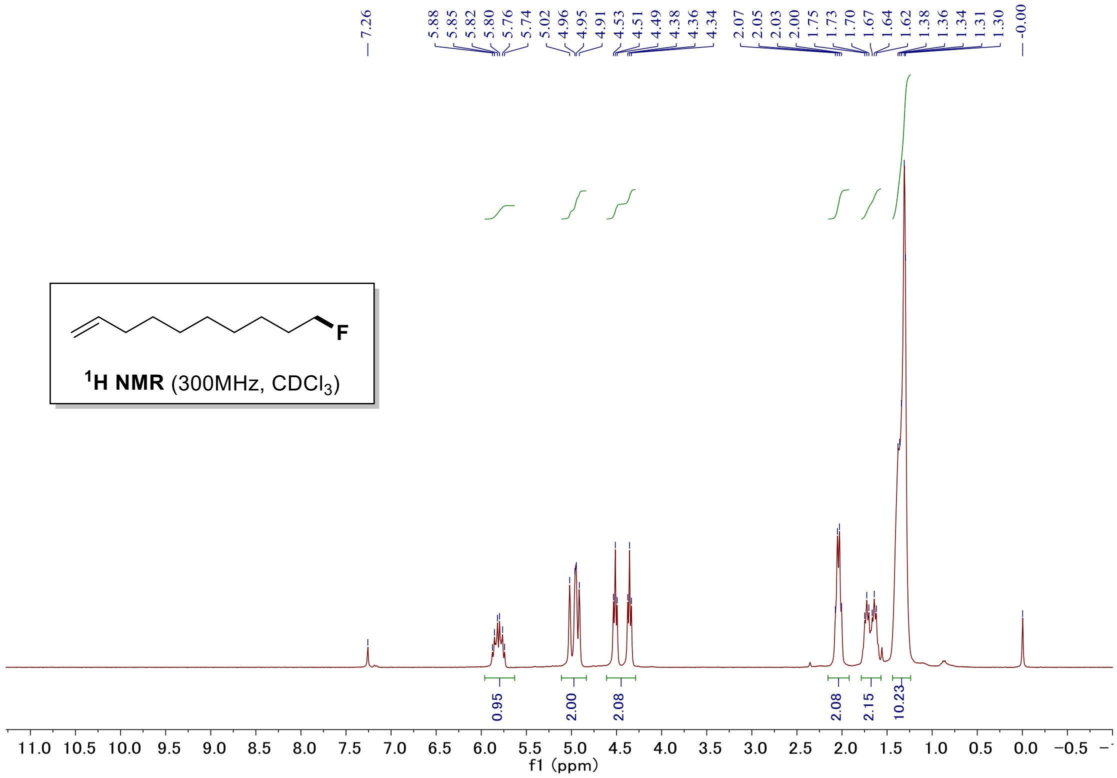

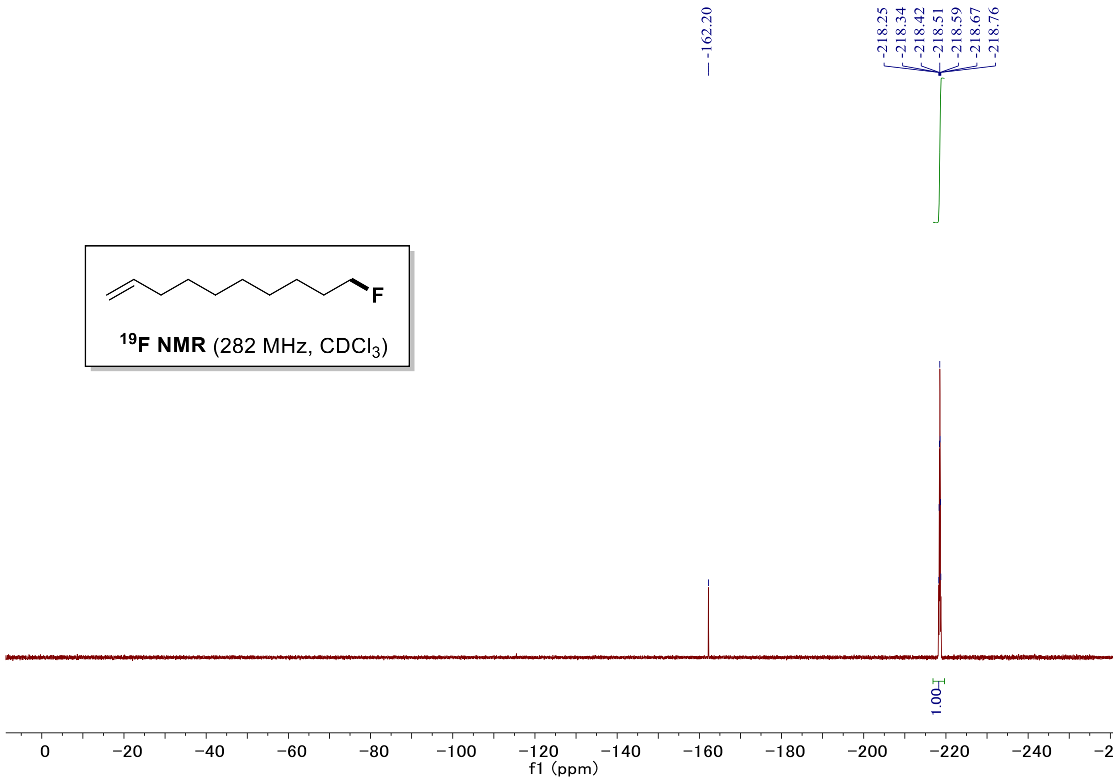

1,8-Difluorooctane (2m)

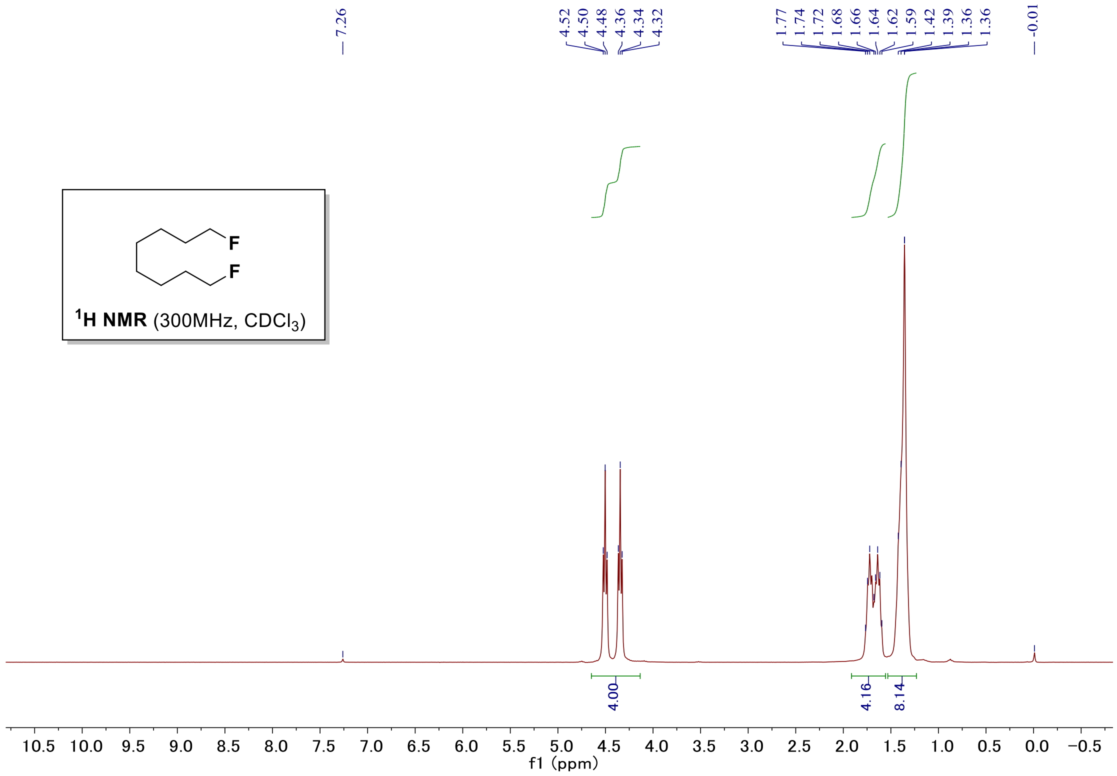

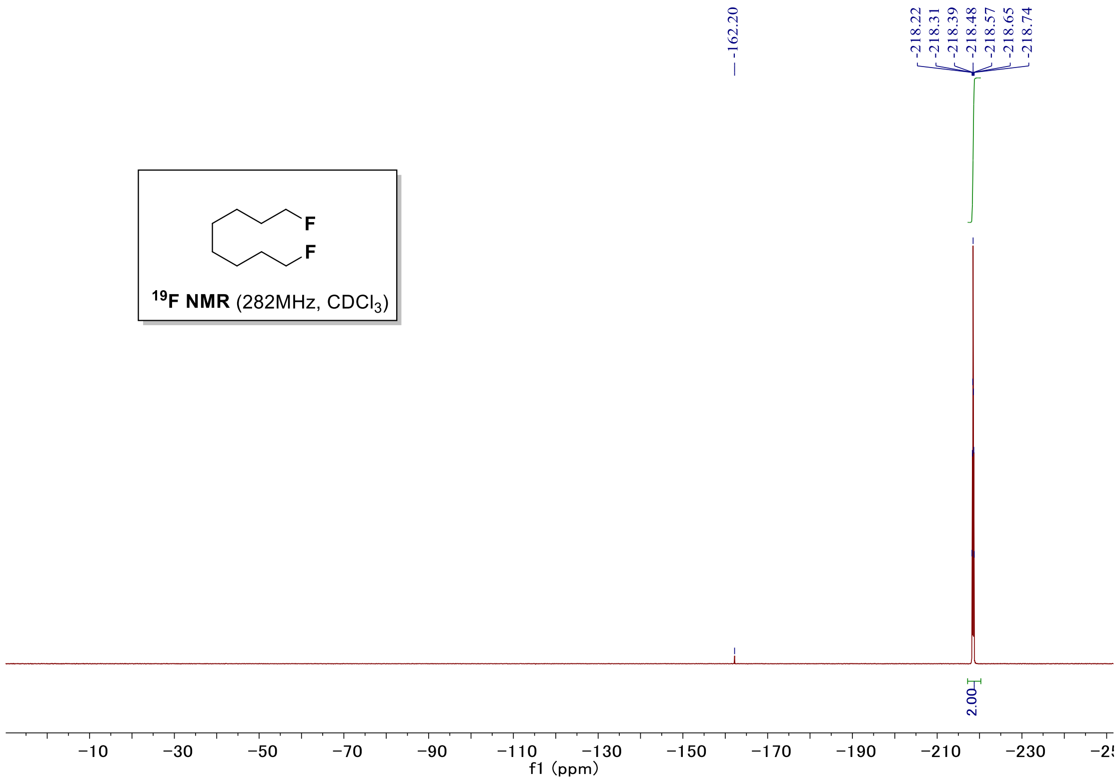

(3-Fluorobutyl)benzene (2n)

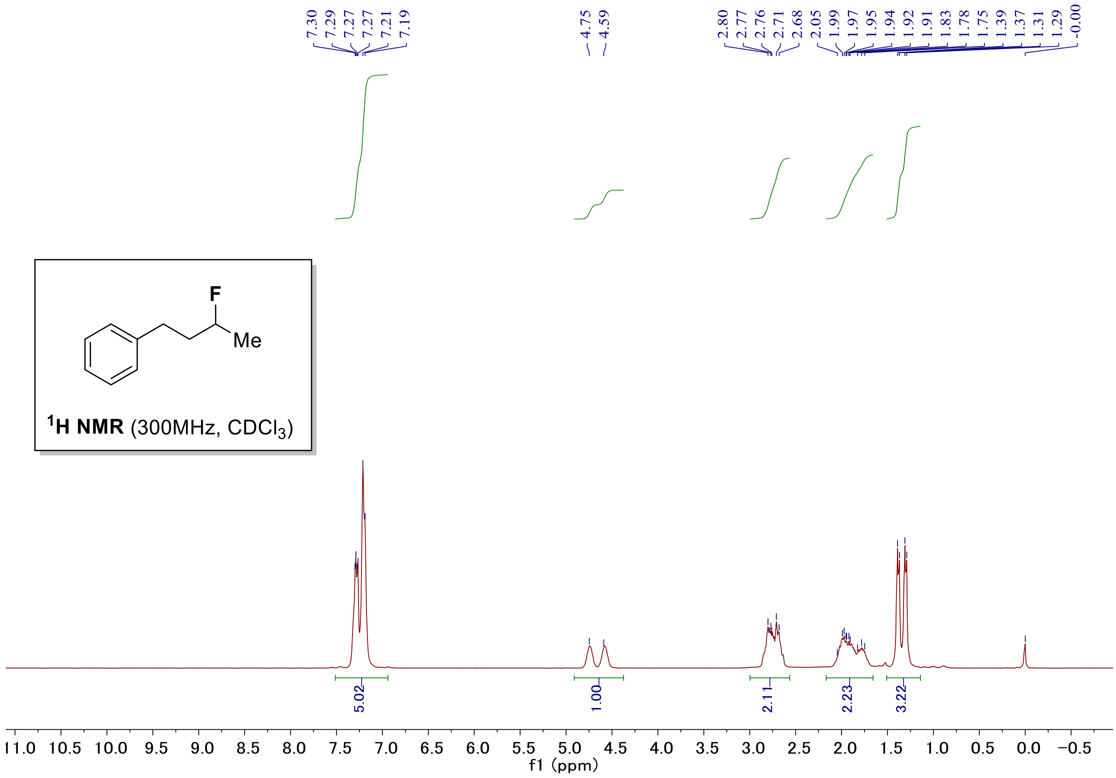

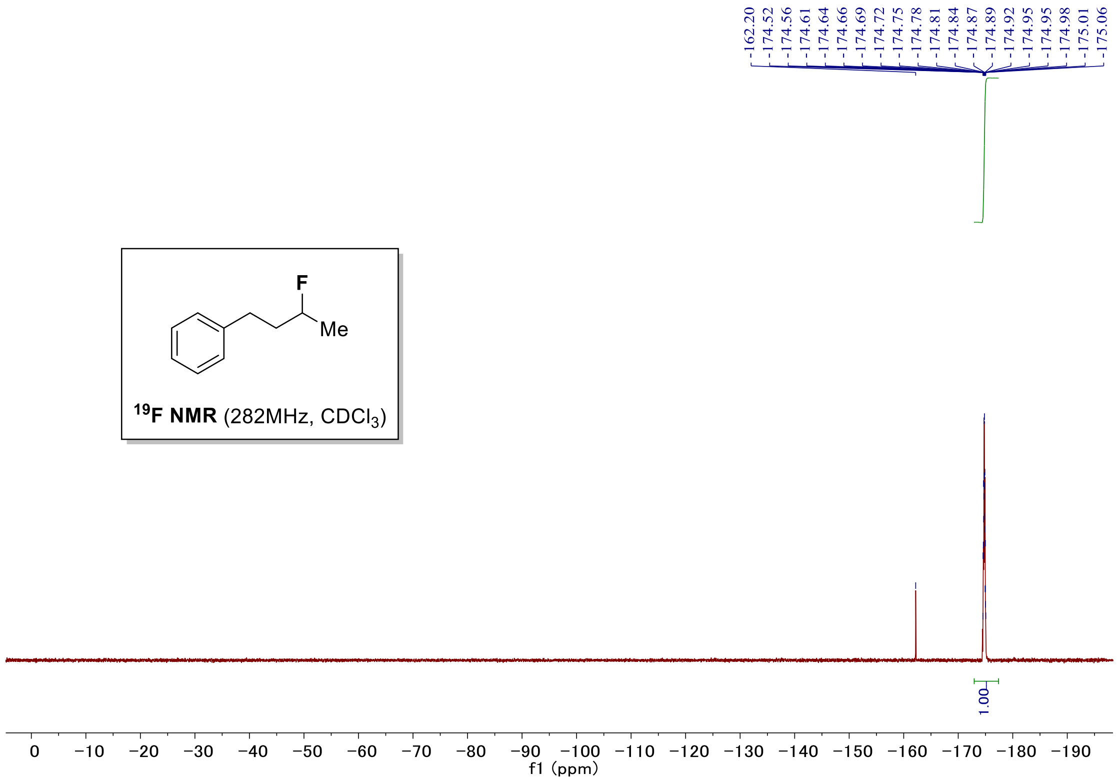

1-(3-Fluoropropyl)adamantane (2h)

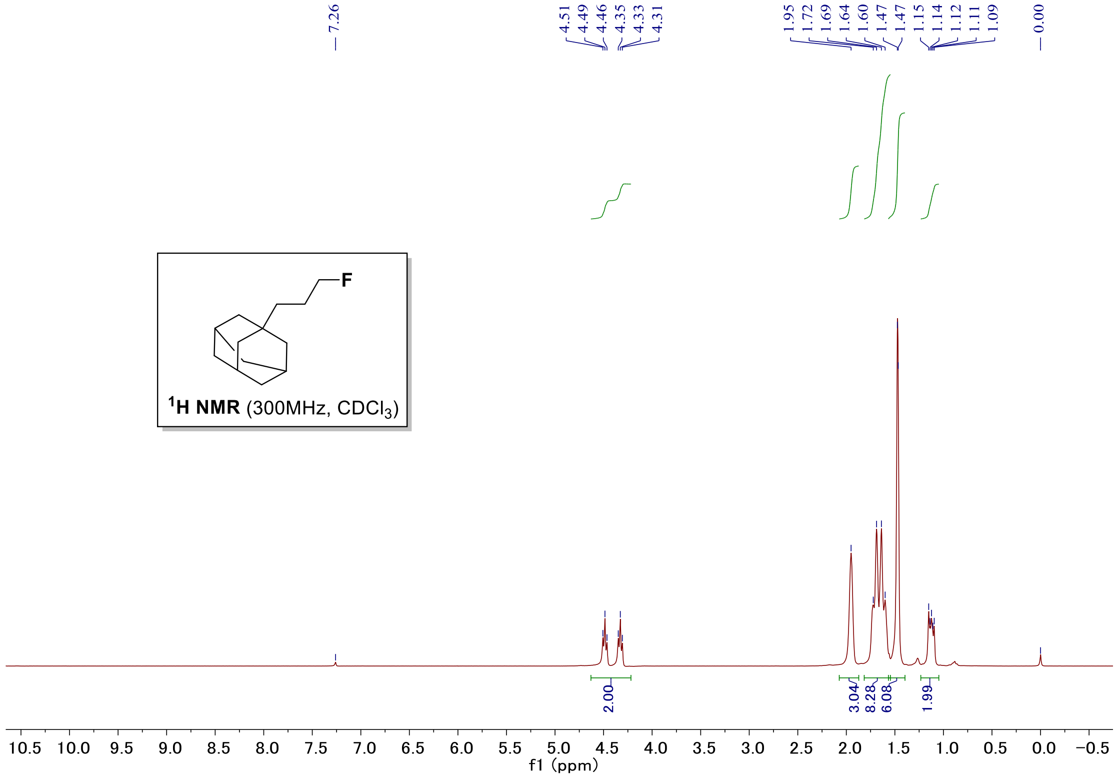

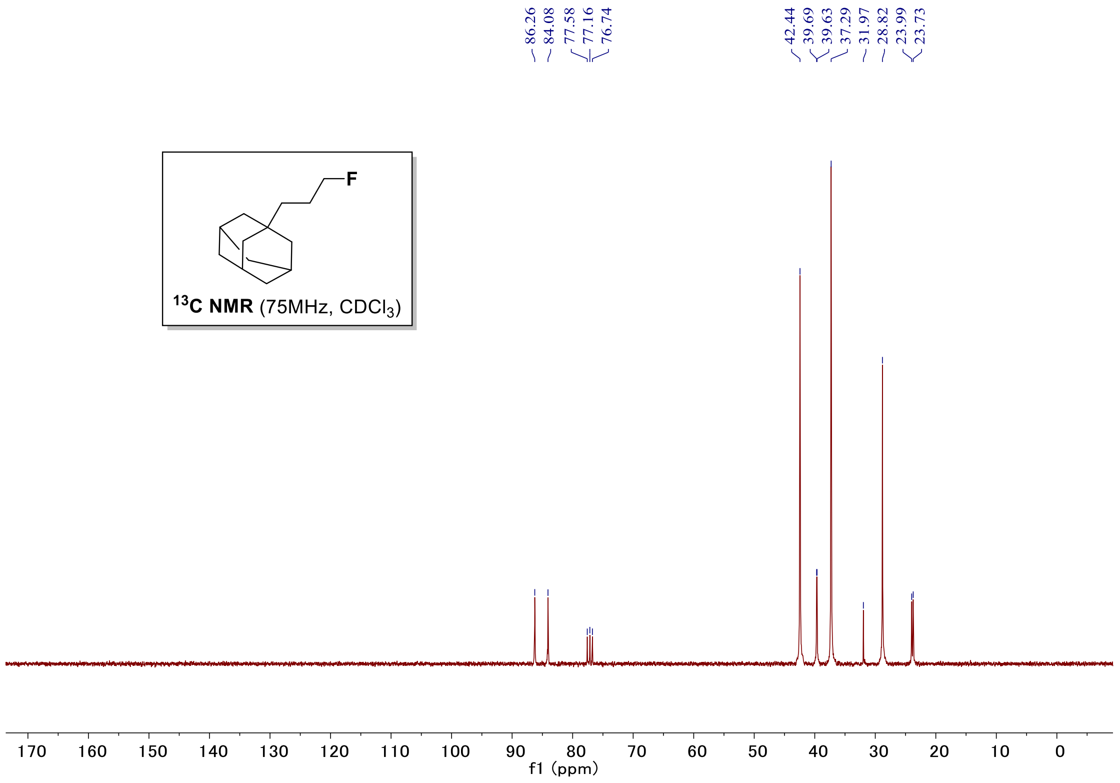

86.26
84.08
77.58
77.16
76.74
42.44
39.69
39.63
37.29
31.97
28.82
23.99
23.73
F
^{13}C NMR (75MHz, CDCl$_3$)
f1 (ppm)

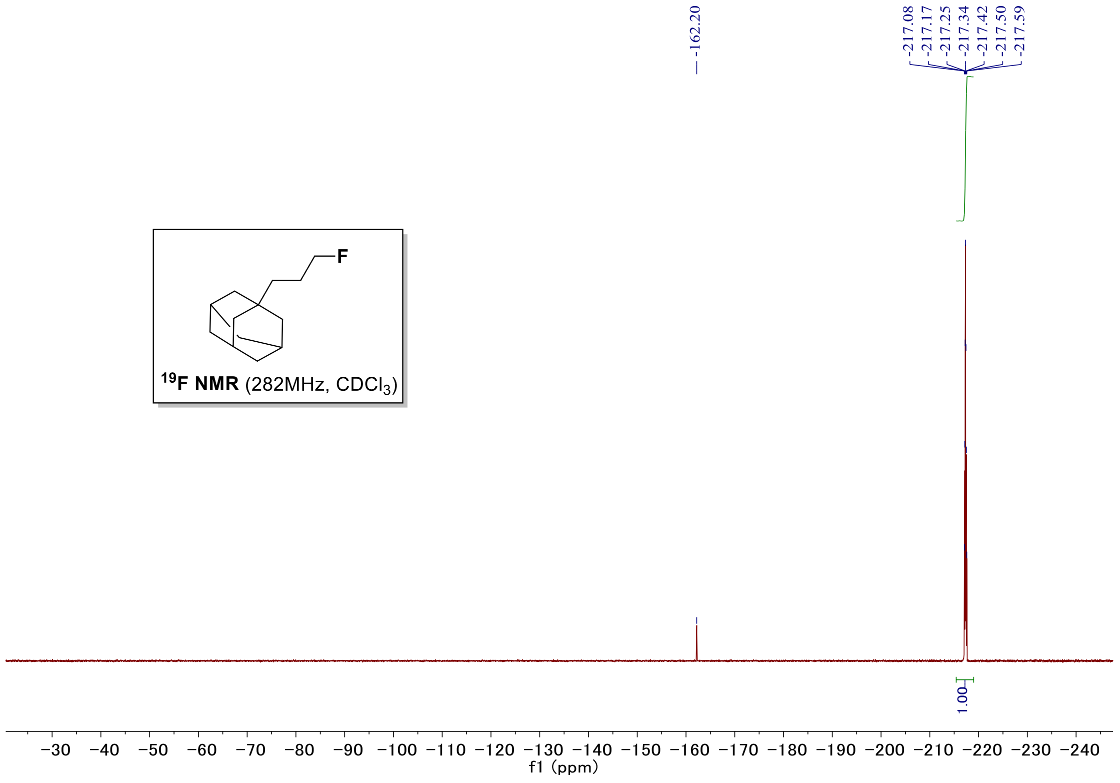

-162.20
-217.08
-217.17
-217.25
-217.34
-217.42
-217.50
-217.59
F
^{19}F NMR (282MHz, CDCl$_3$)
1.00
f1 (ppm)

1-(*tert*-Butyl)-4-vinylbenzene (3d)

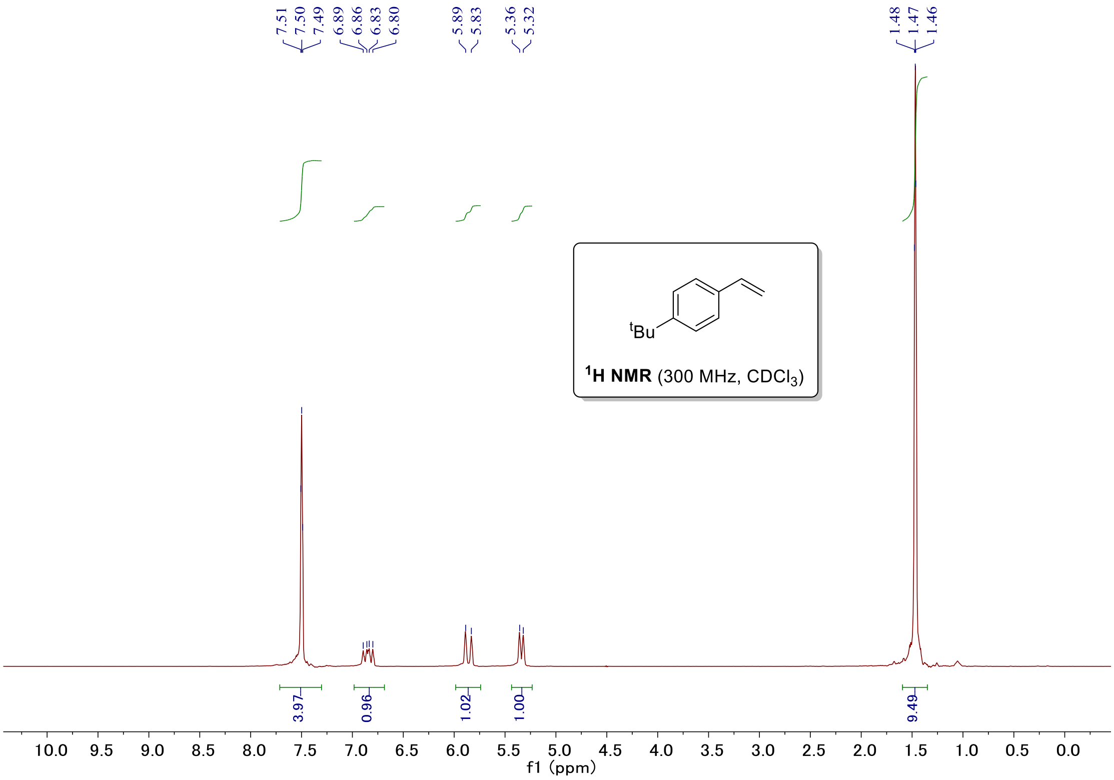

1-Methoxy-4-vinylbenzene (3f)

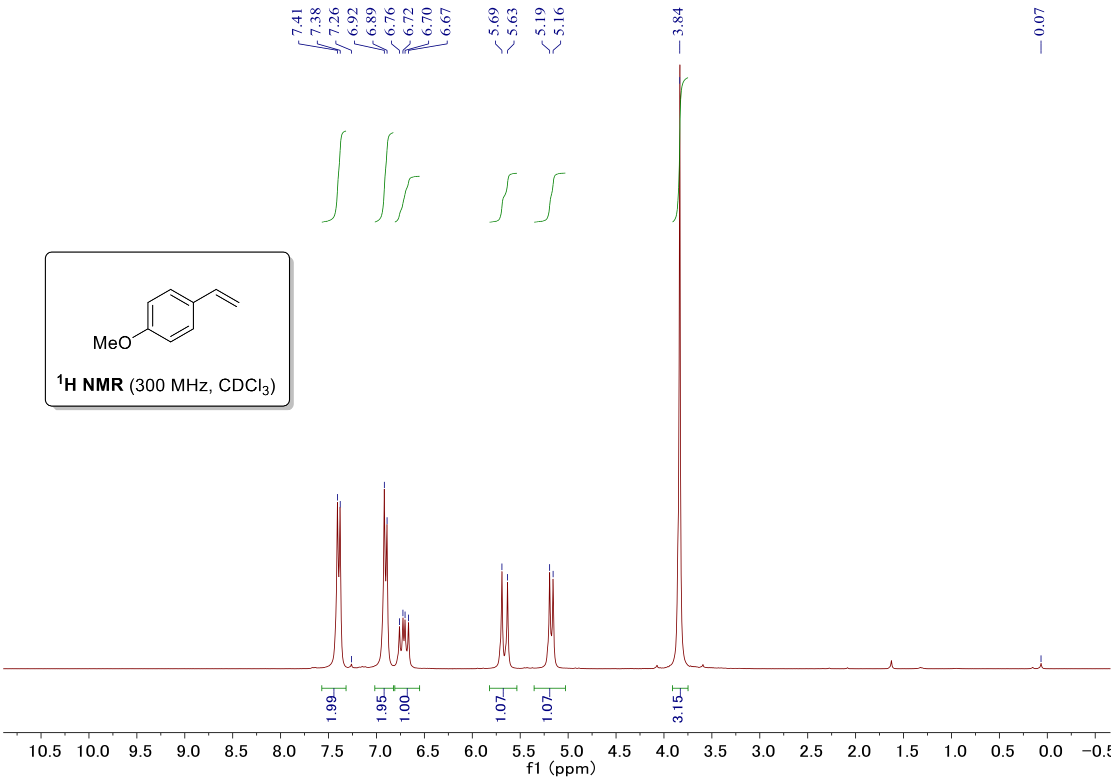

1-Methoxy-2-vinylbenzene (3g)

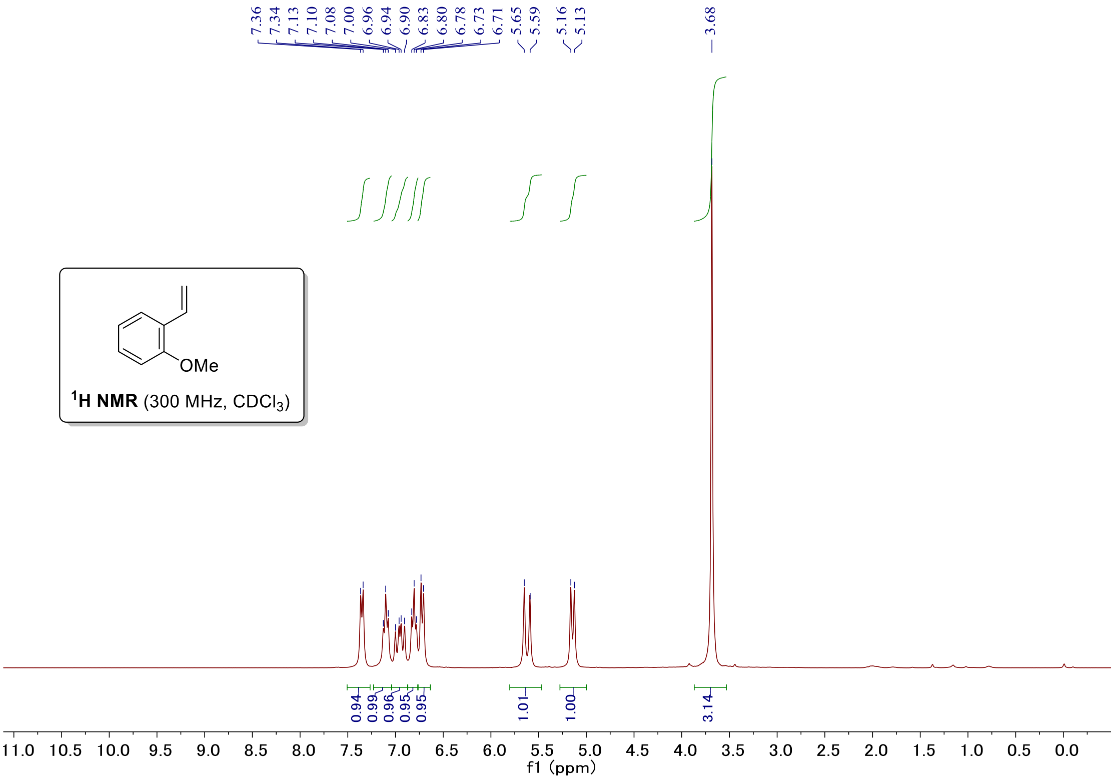

5-Vinylbenzo[*d*][1,3]dioxole (3h)

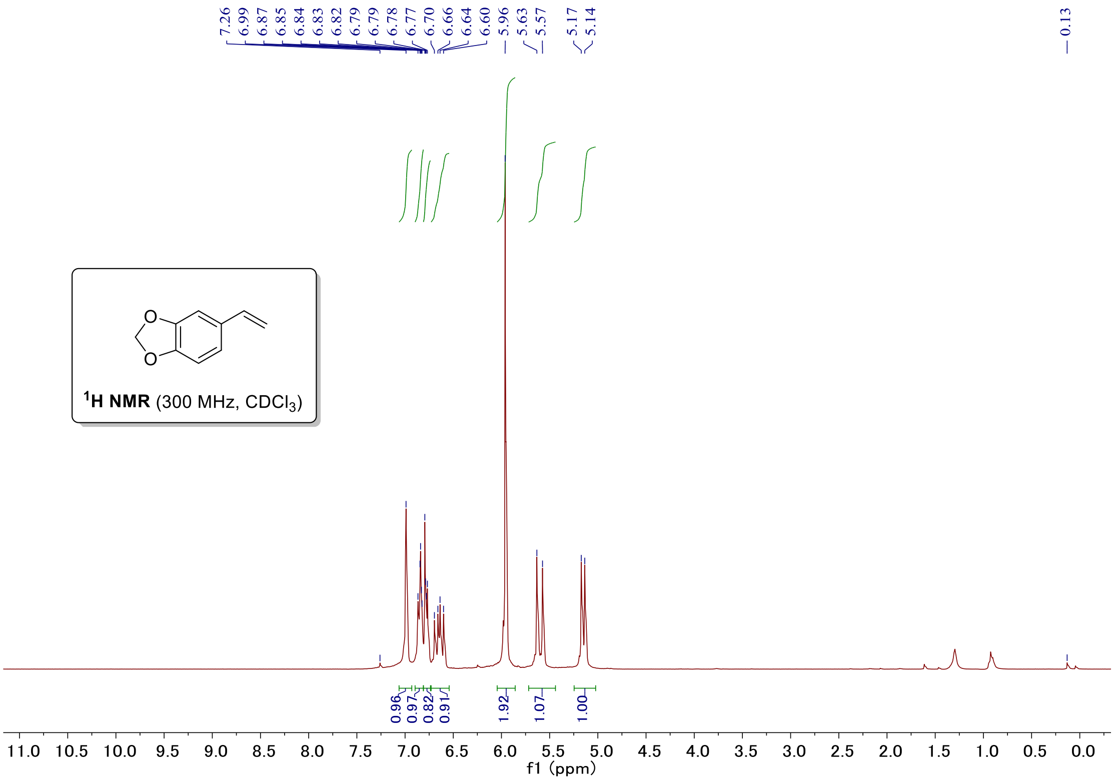

Methyl(4-vinylphenyl)sulfane (3i)

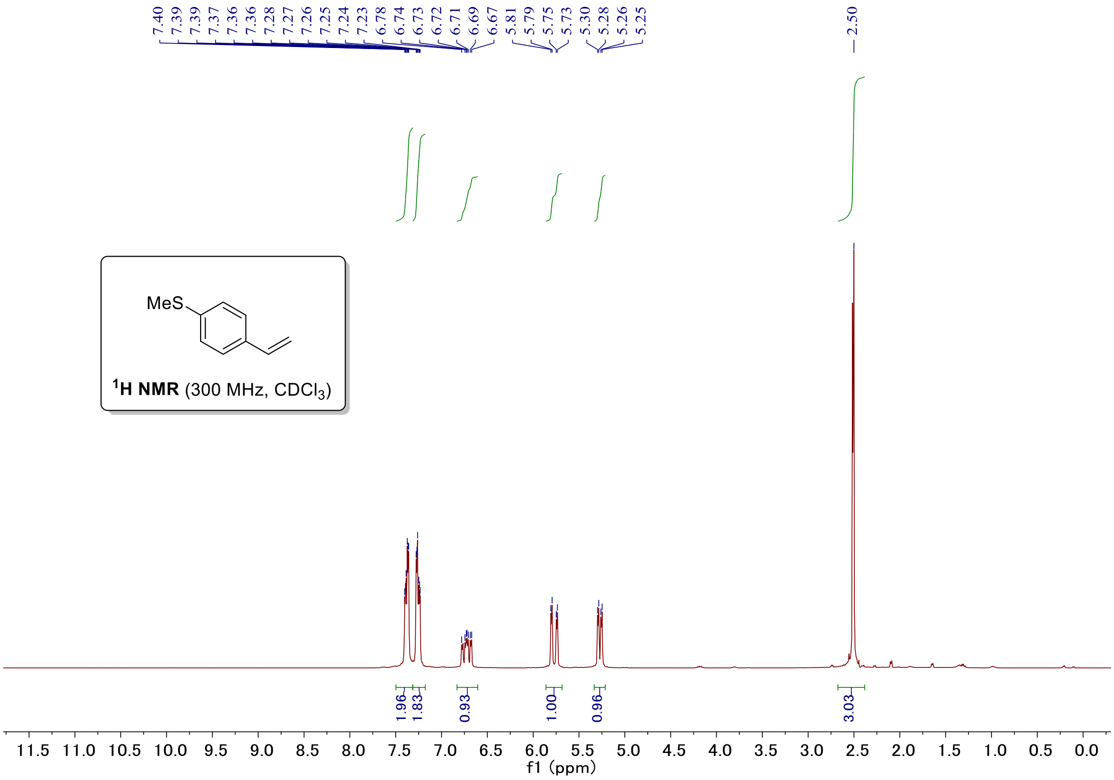

(4-Methoxybut-1-en-2-yl)benzene (3l)

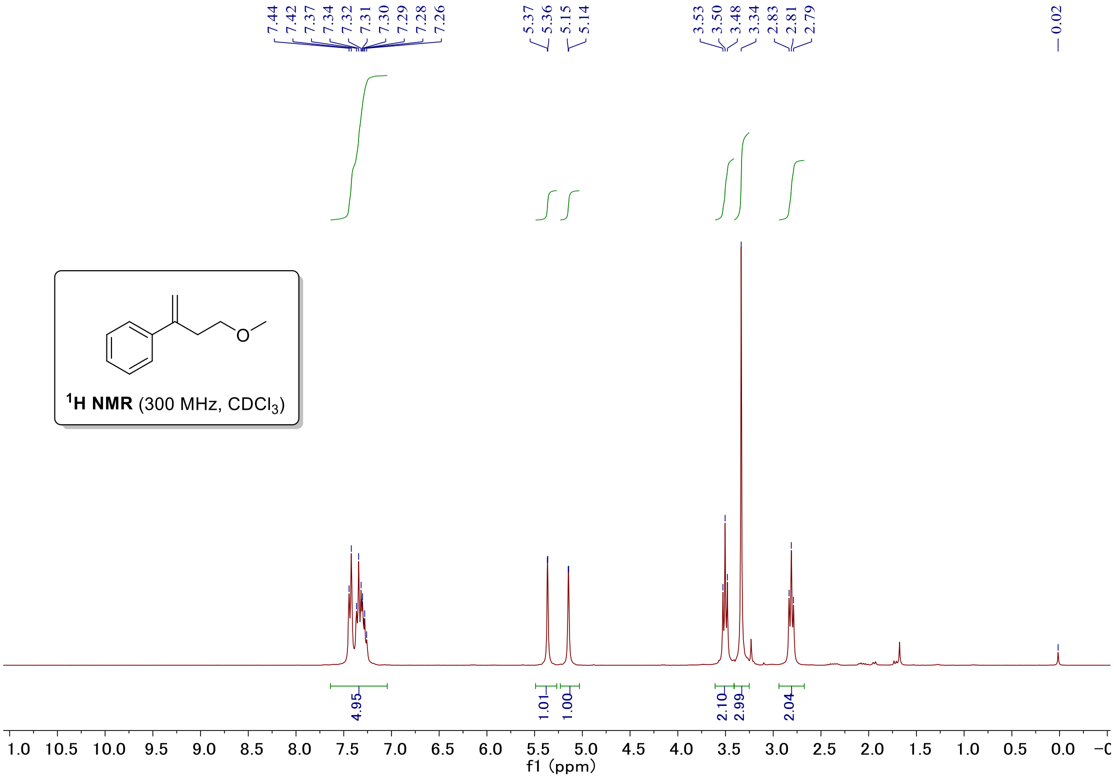

3-Vinylestrone derivative (3m)

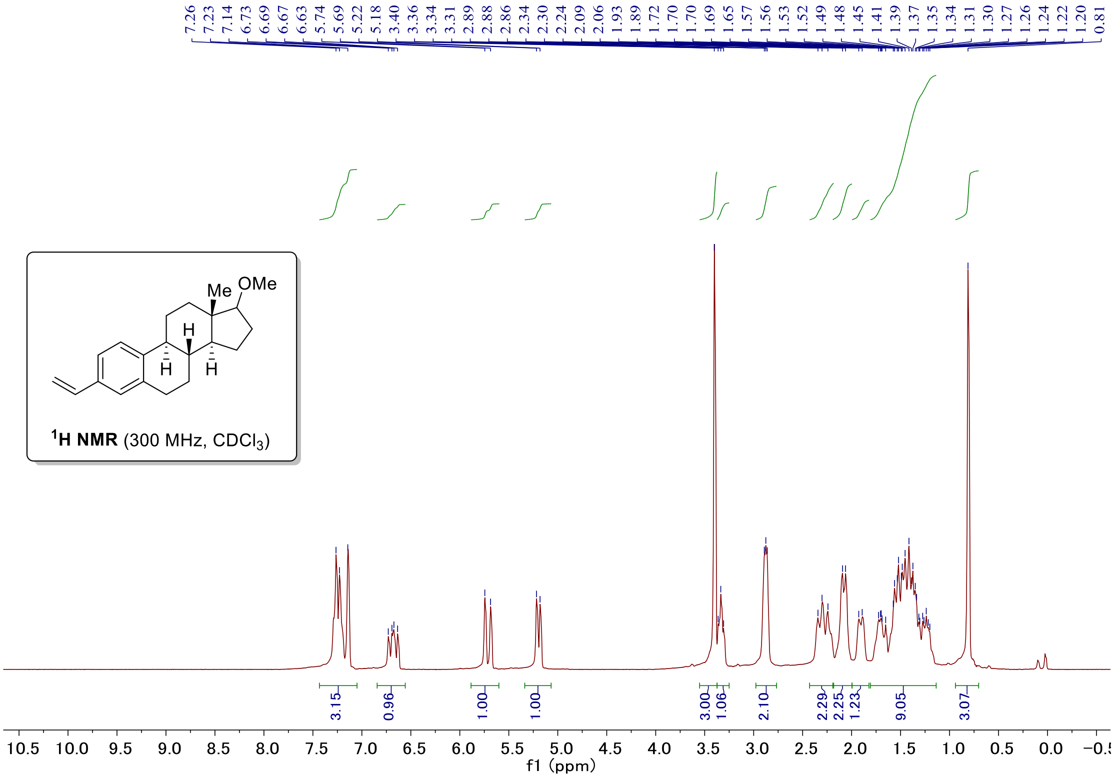

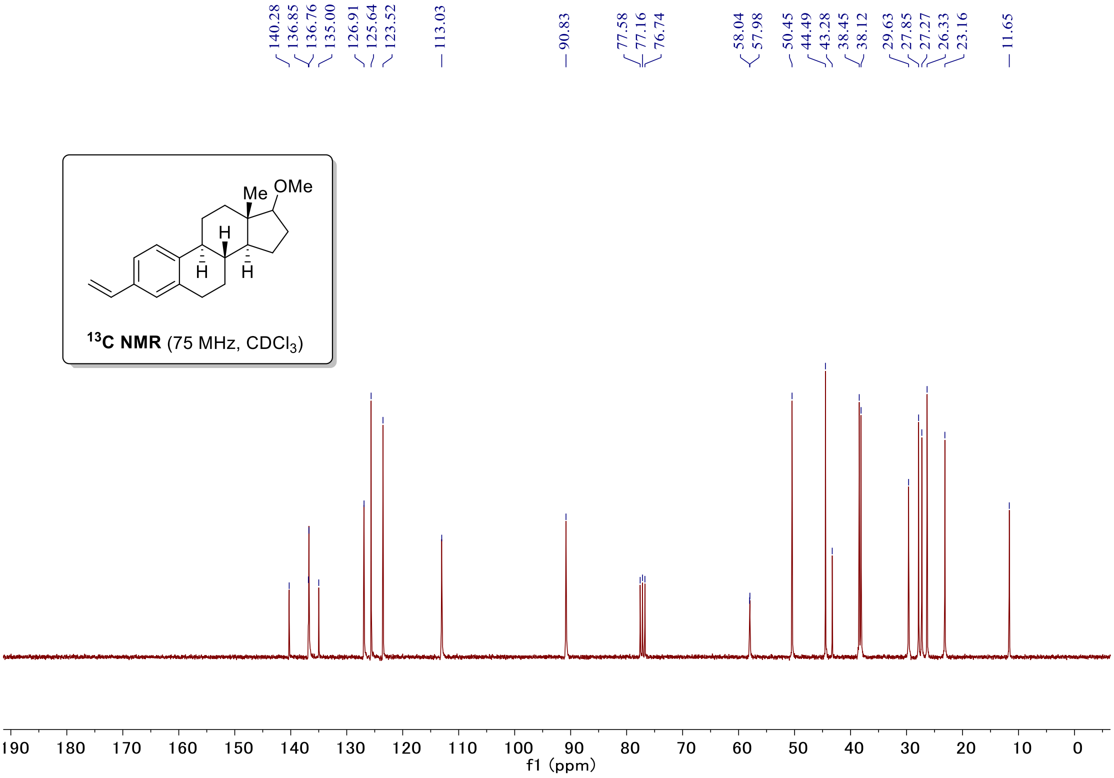

(*E*)-Buta-1,3-dien-1-ylbenzene (3n)

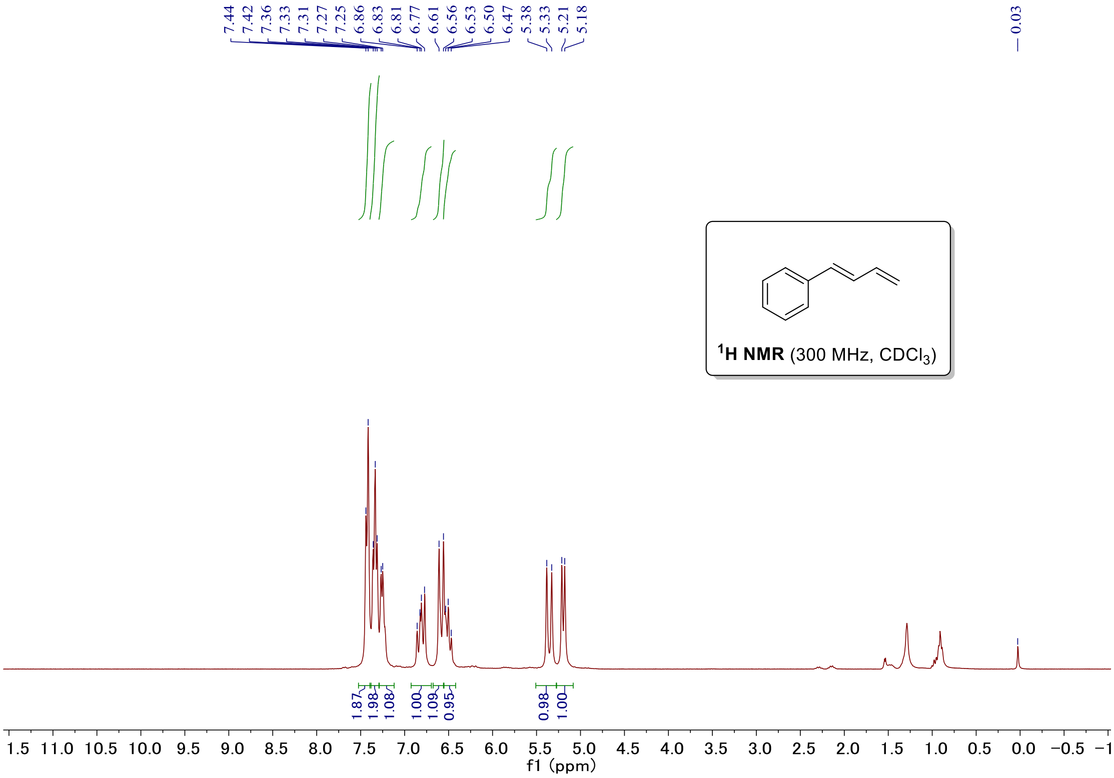

Buta-1,3-dien-2-ylbenzene (3o)

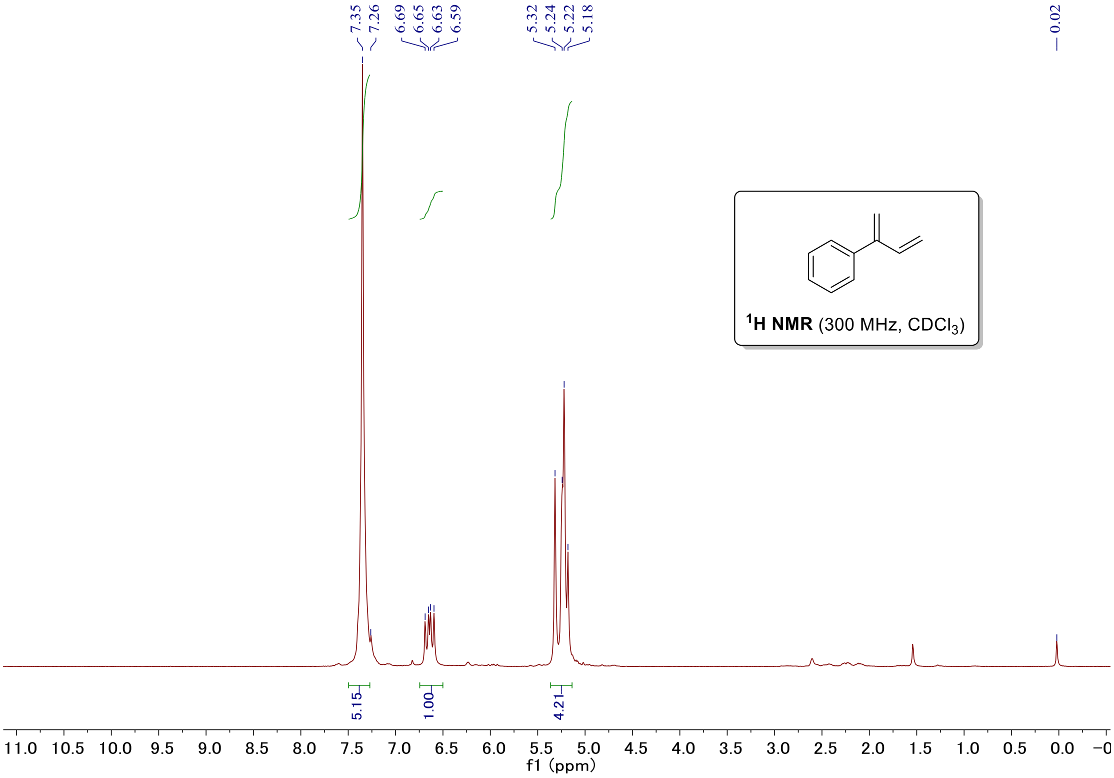

(1-Cyclopropylvinyl)benzene (3p)

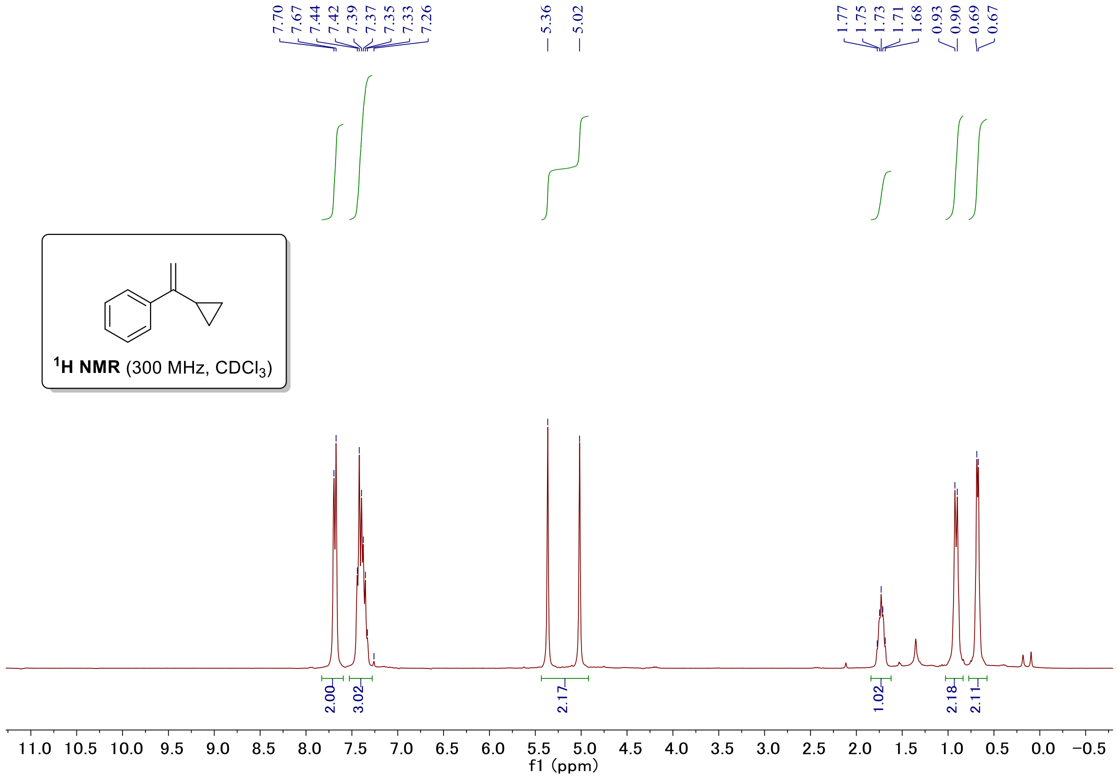

Triethyl(4,4,5,5-tetramethyl-1,3,2-dioxaborolan-2-yl)silane

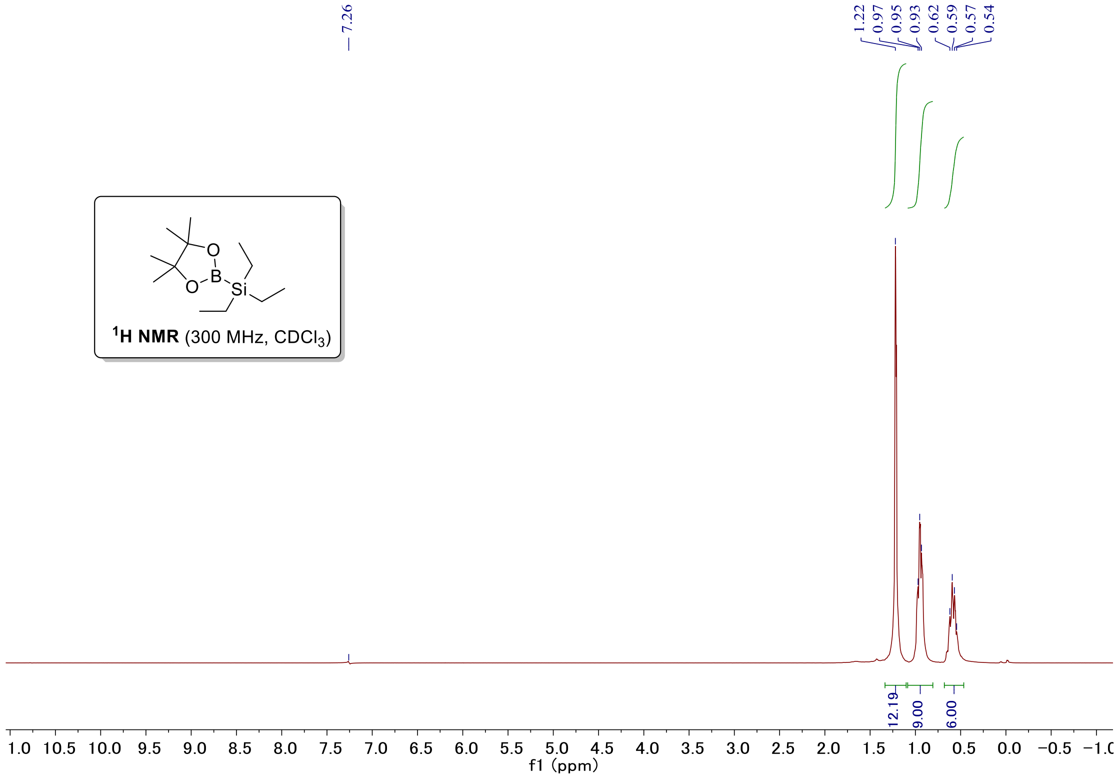

Tripropyl(4,4,5,5-tetramethyl-1,3,2-dioxaborolan-2-yl)silane

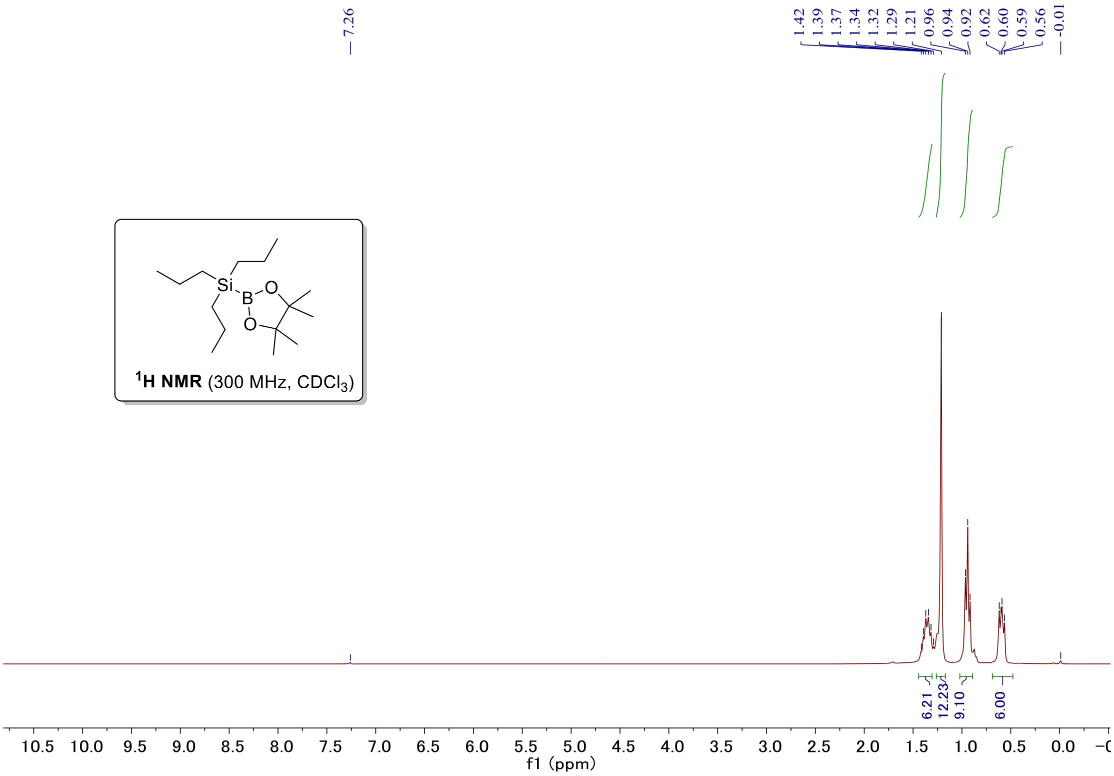

tert-Butyldimethyl(4,4,5,5-tetramethyl-1,3,2-dioxaborolan-2-yl)silane

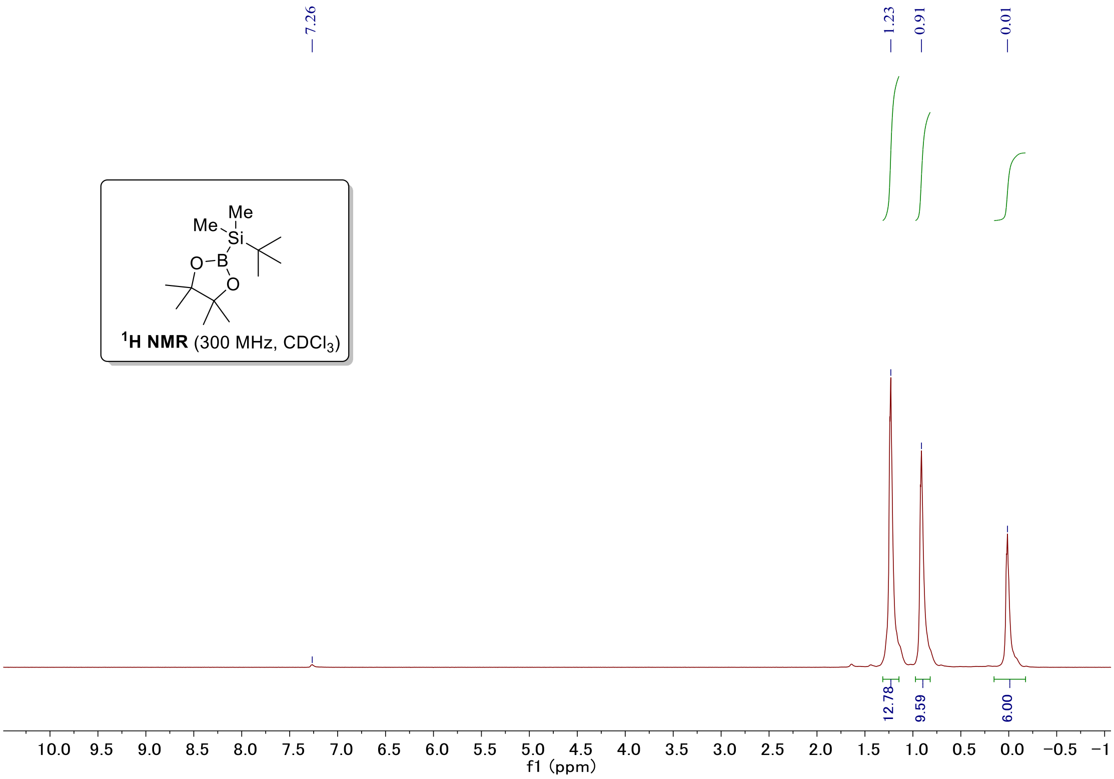

(2-(Biphenyl-4-yl)-2-phenylethyl)triethylsilane (4aa)

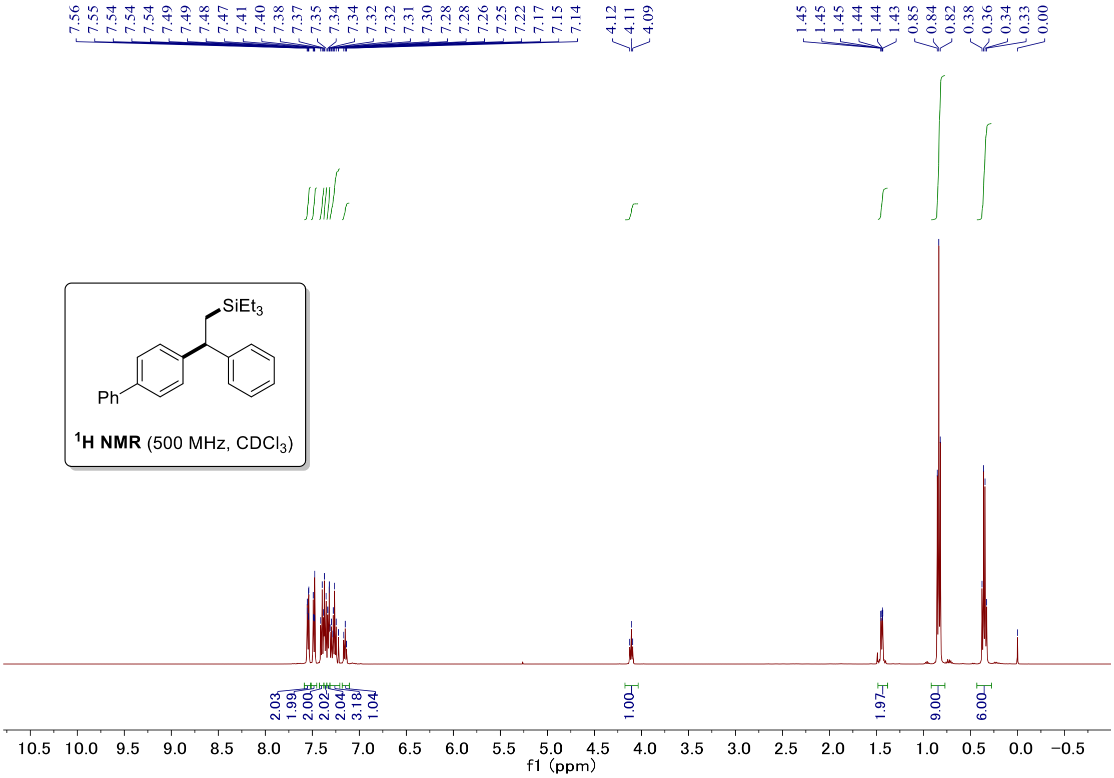

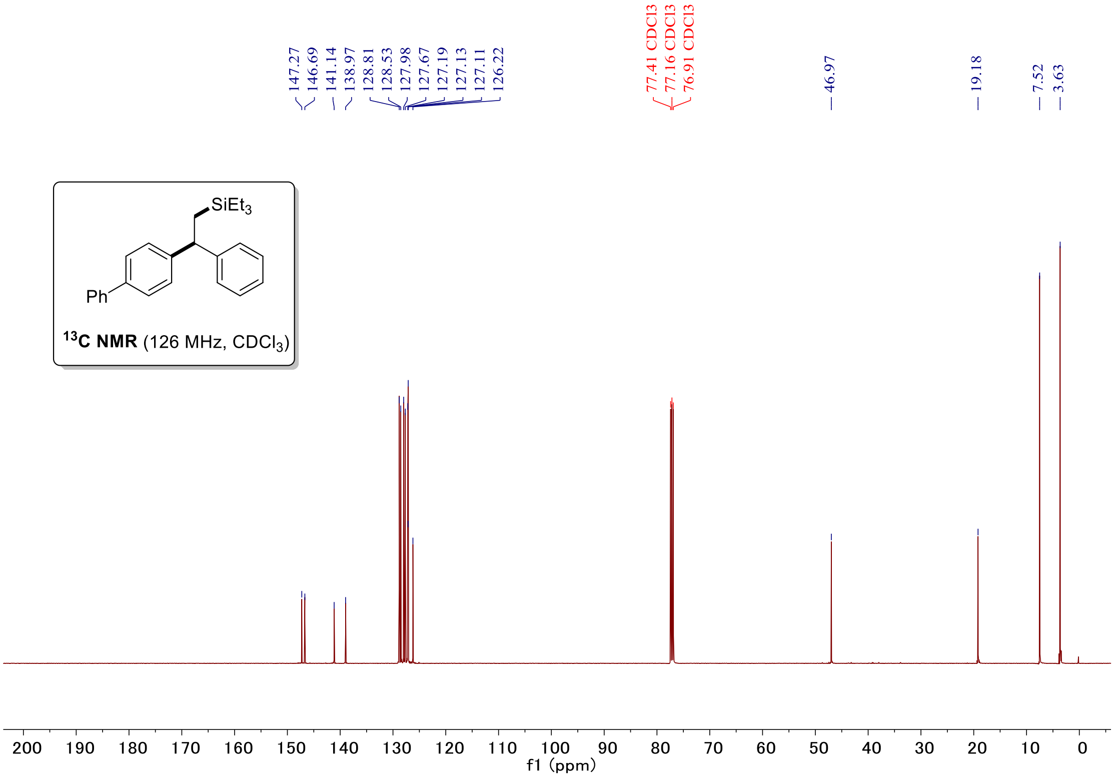

(2-(Biphenyl-3-yl)-2-phenylethyl)triethylsilane (4ba)

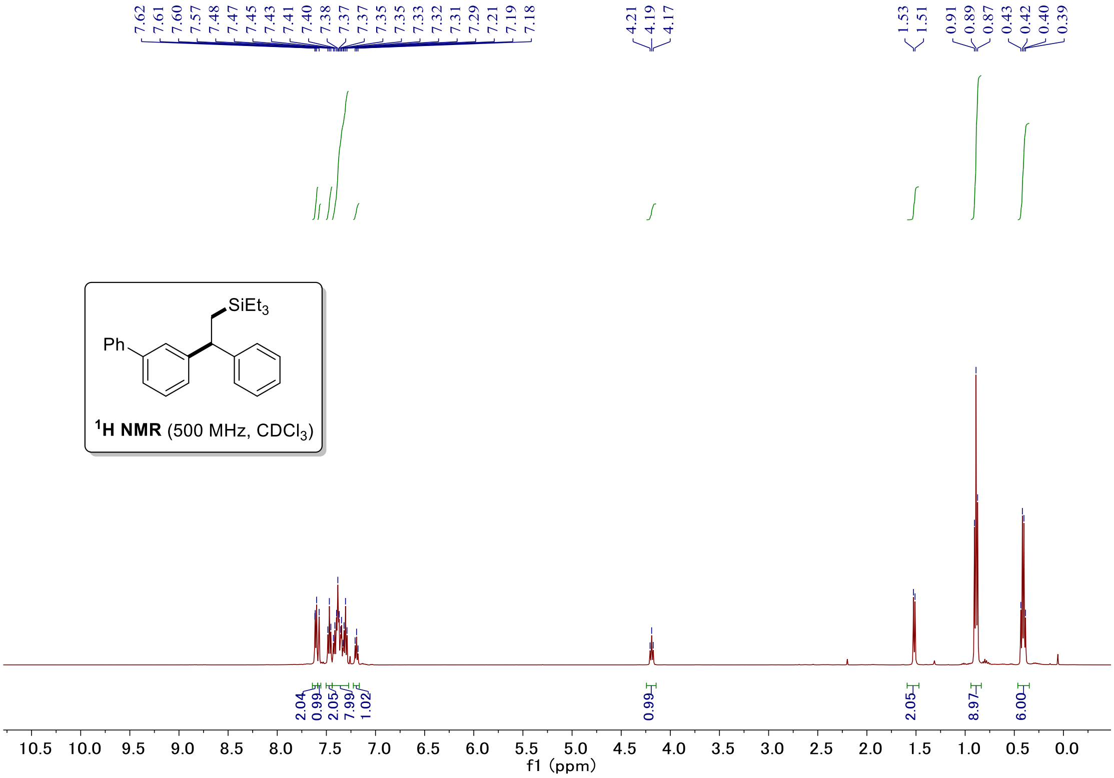

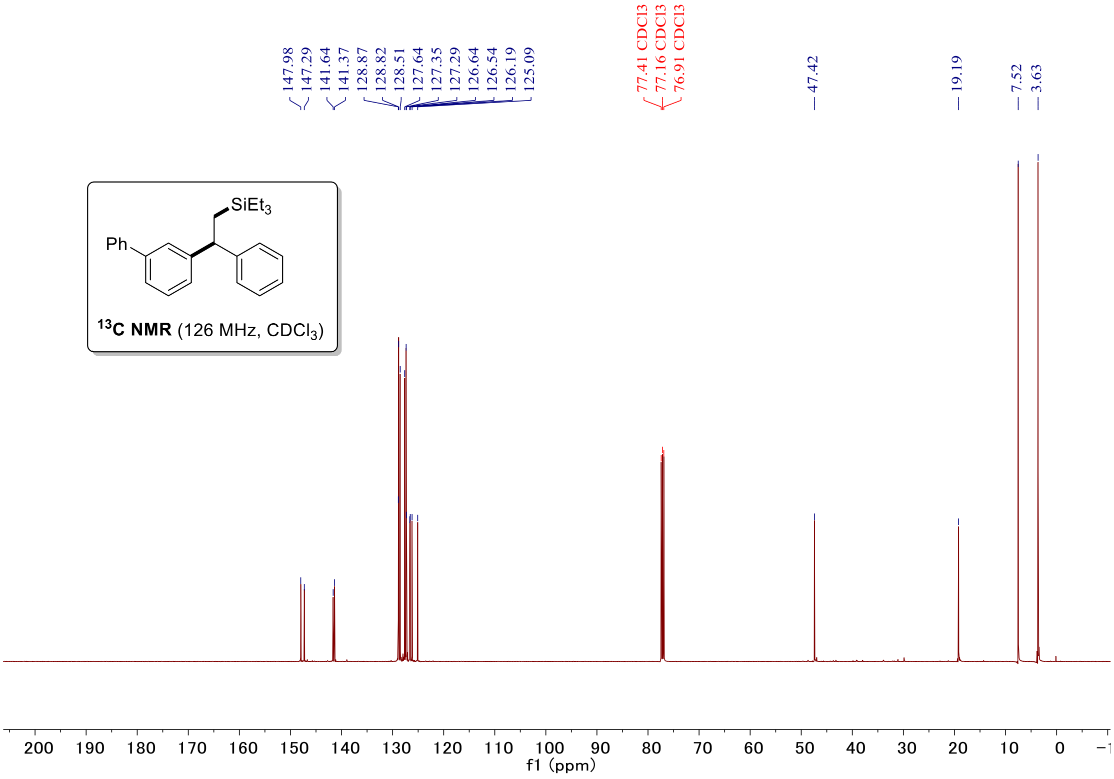

(2-(Biphenyl]-2-yl)-2-phenylethyl)triethylsilane (4ca)

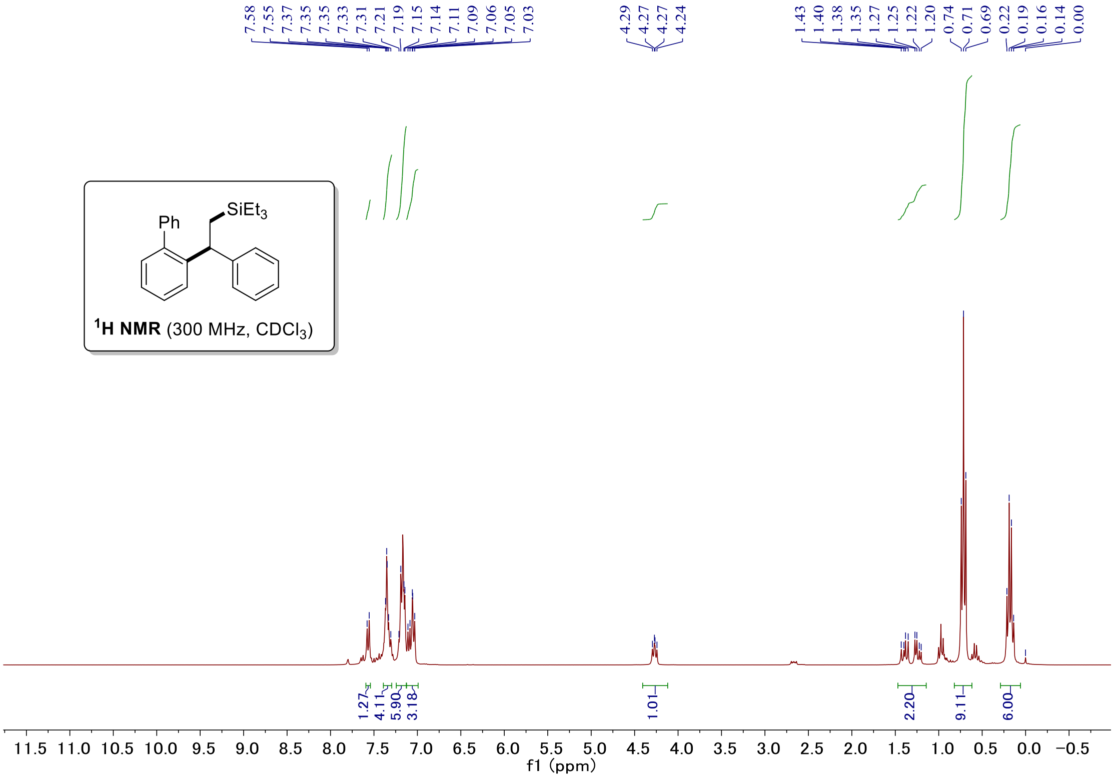

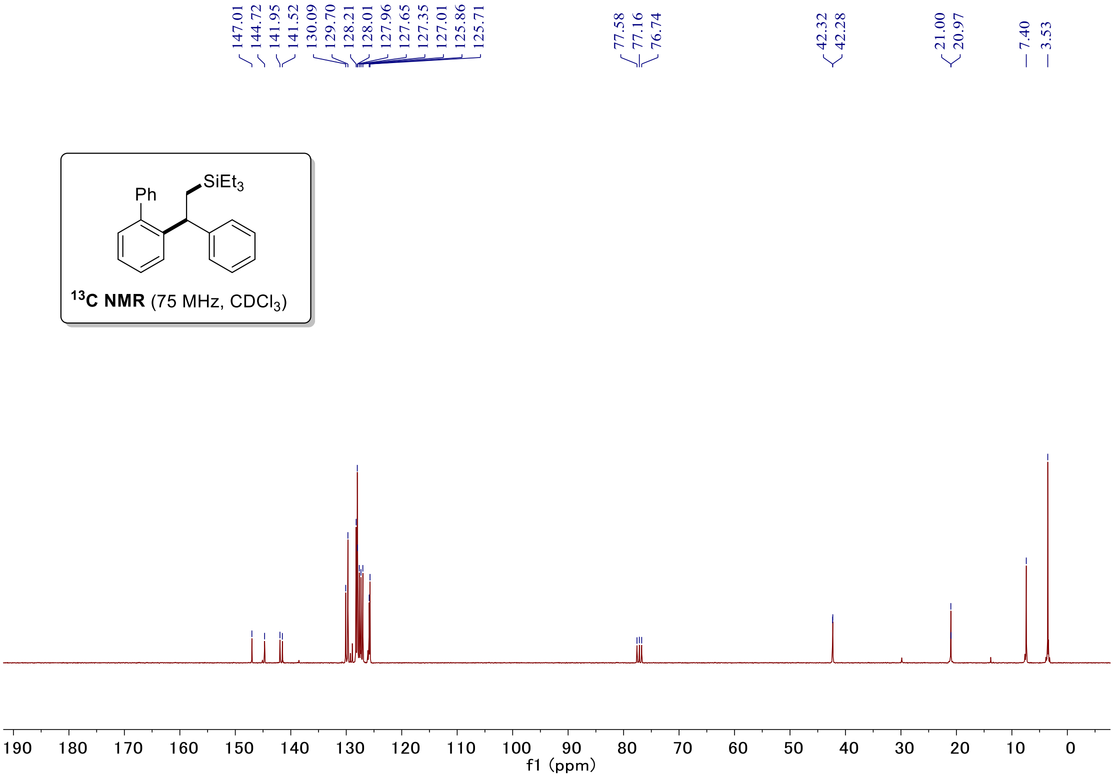

Triethyl(1-(naphthalen-2-yl)-2-phenylethyl)silane (4da)

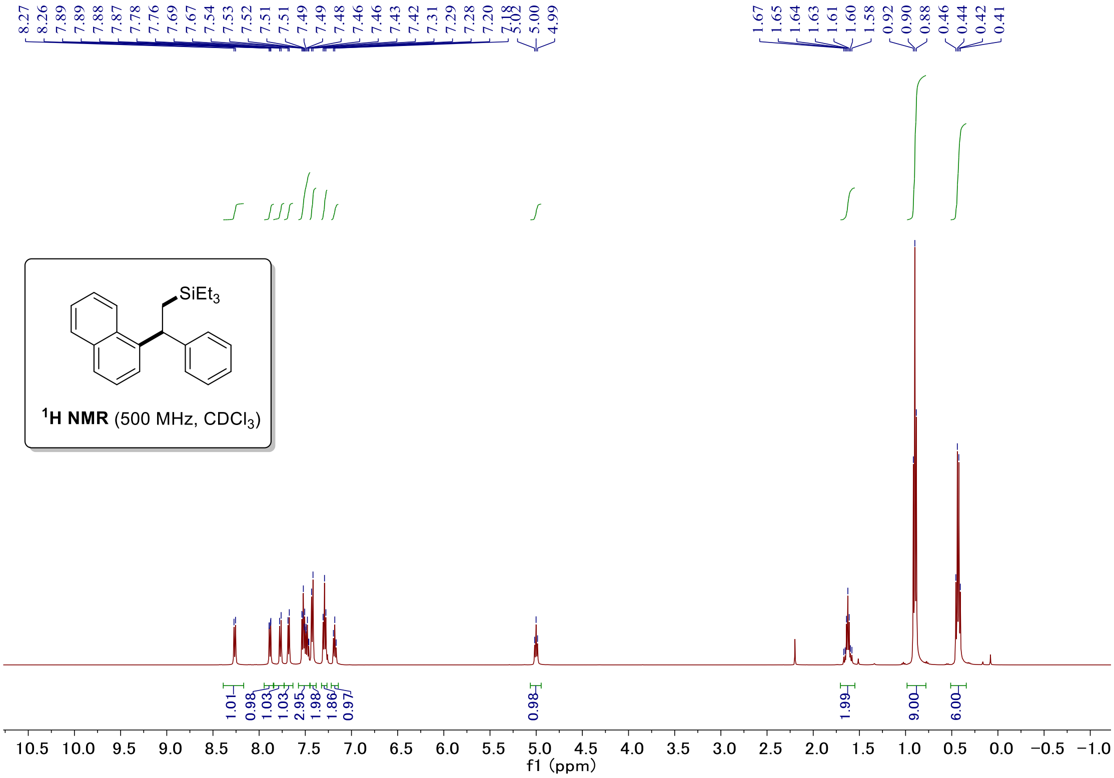

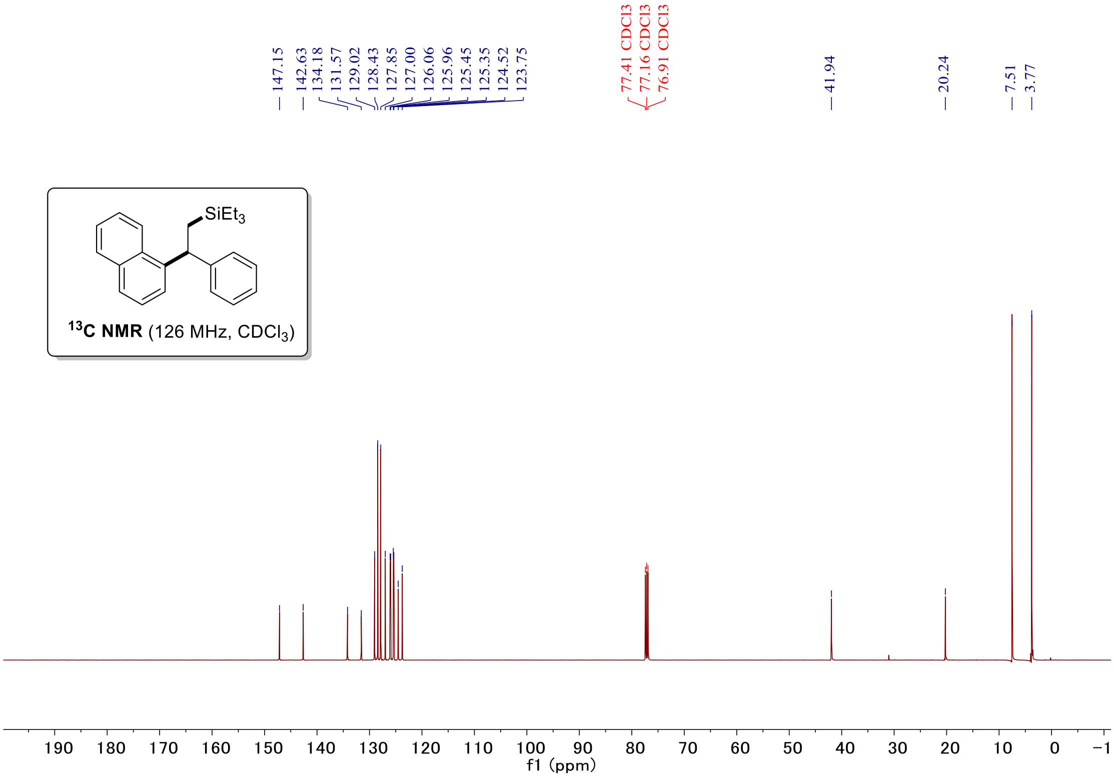

Triethyl(2-(4-(naphthalen-1-yl)phenyl)-2-phenylethyl)silane (4ea)

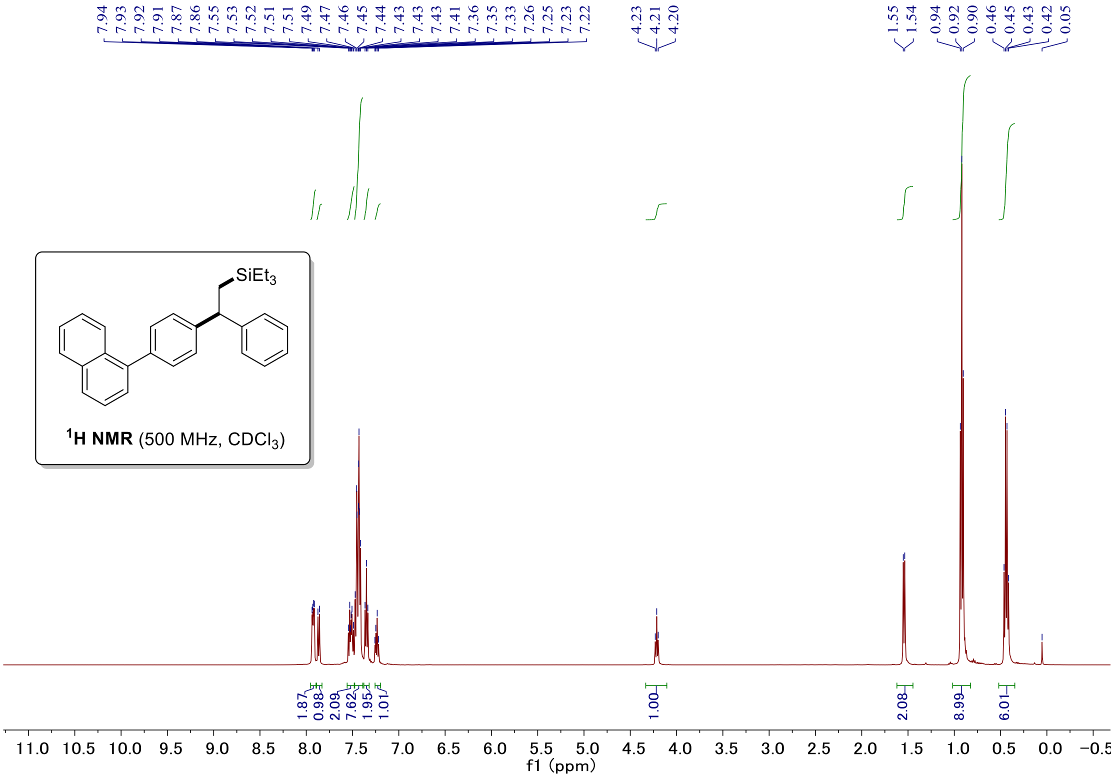

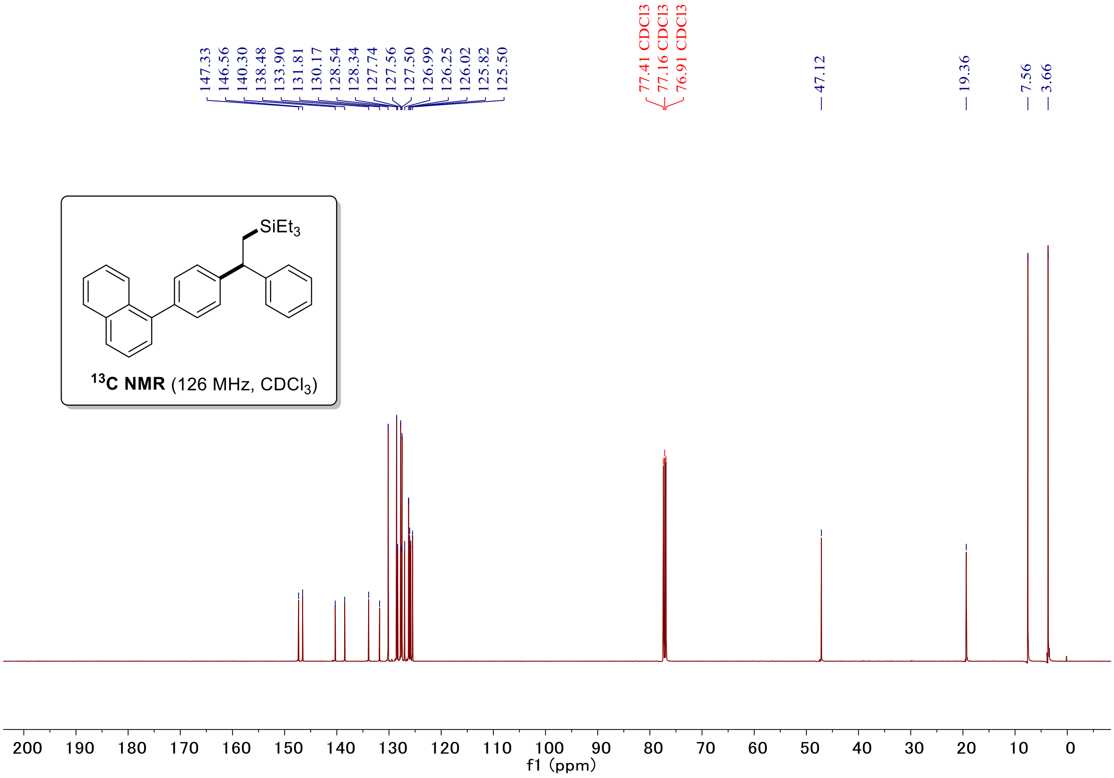

(2,2-Diphenylethyl)triethylsilane (4fa)

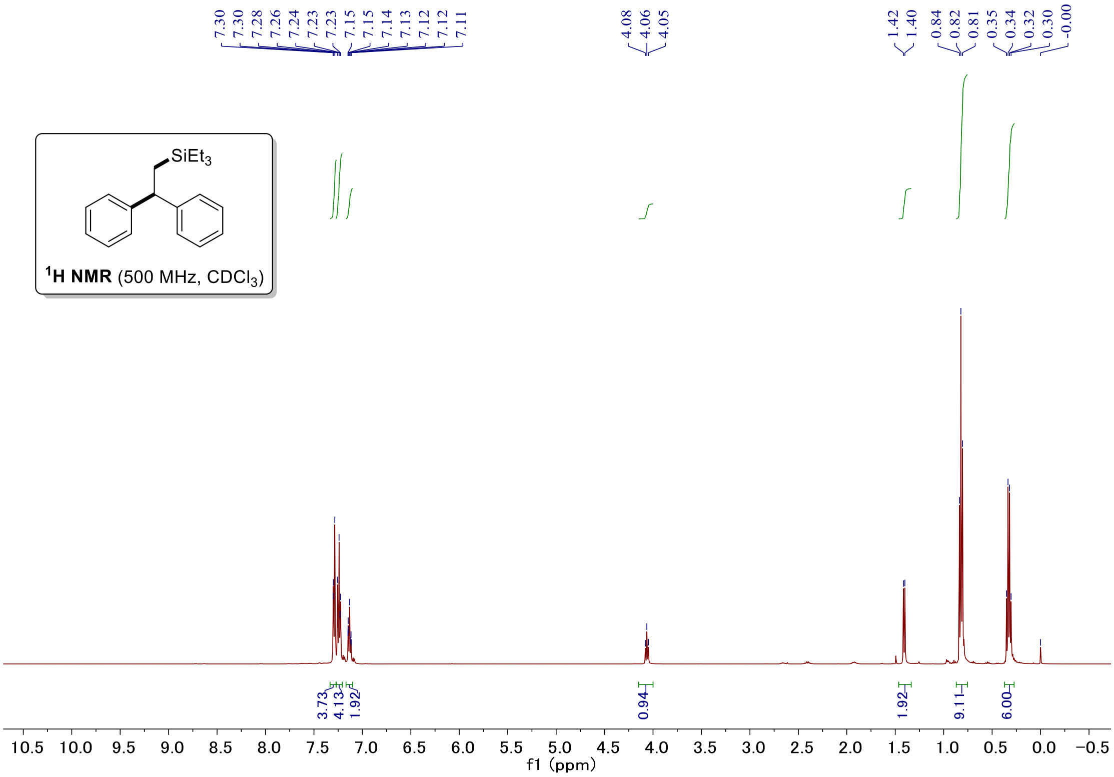

Triethyl(2-phenyl-2-(p-tolyl)ethyl)silane (4ga)

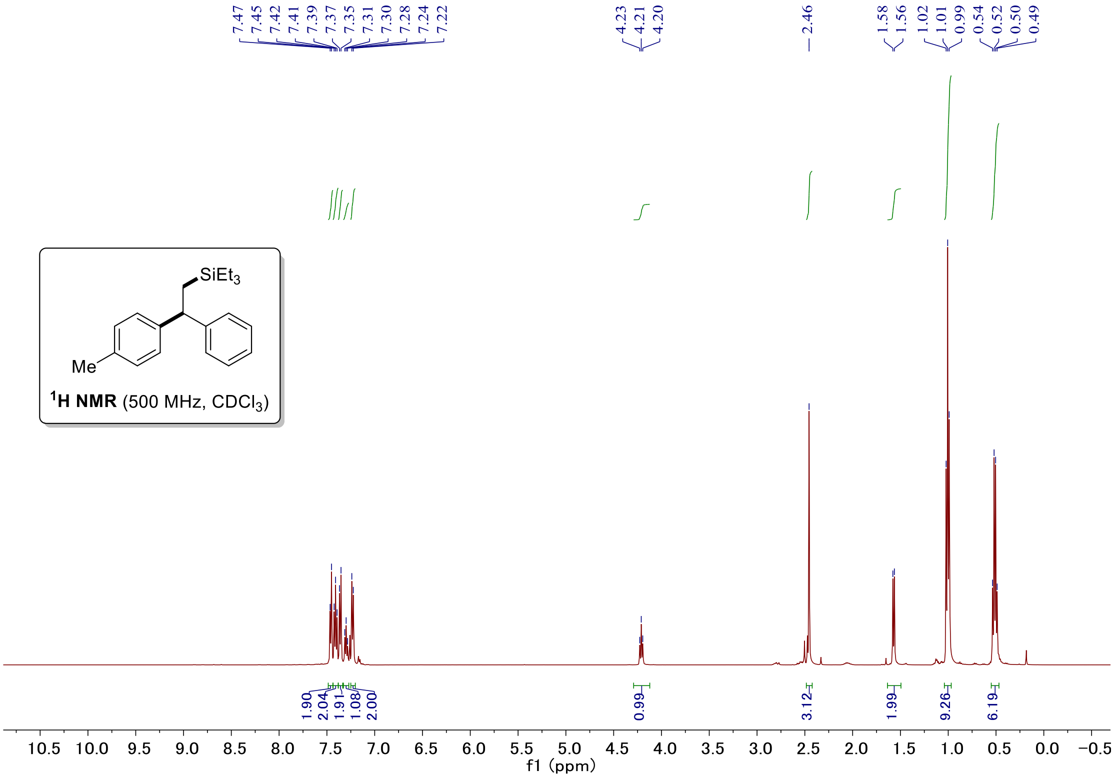

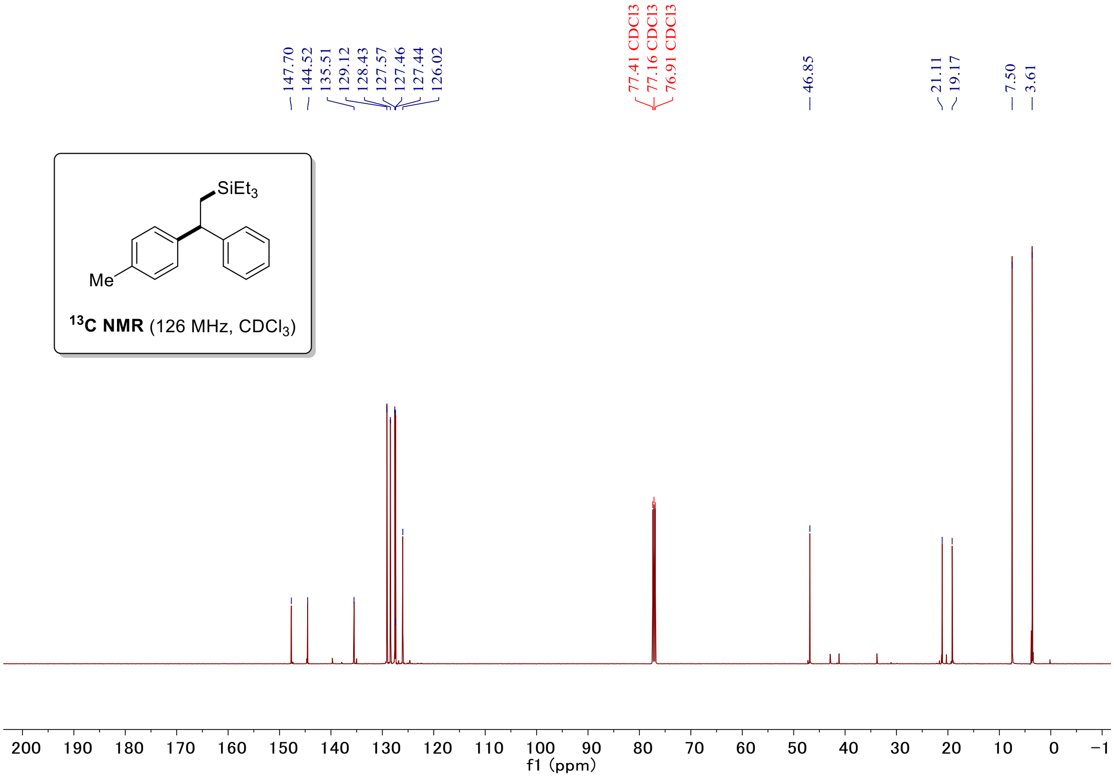

Triethyl(2-(4'-methoxy-biphenyl-4-yl)-2-phenylethyl)silane (4ha)

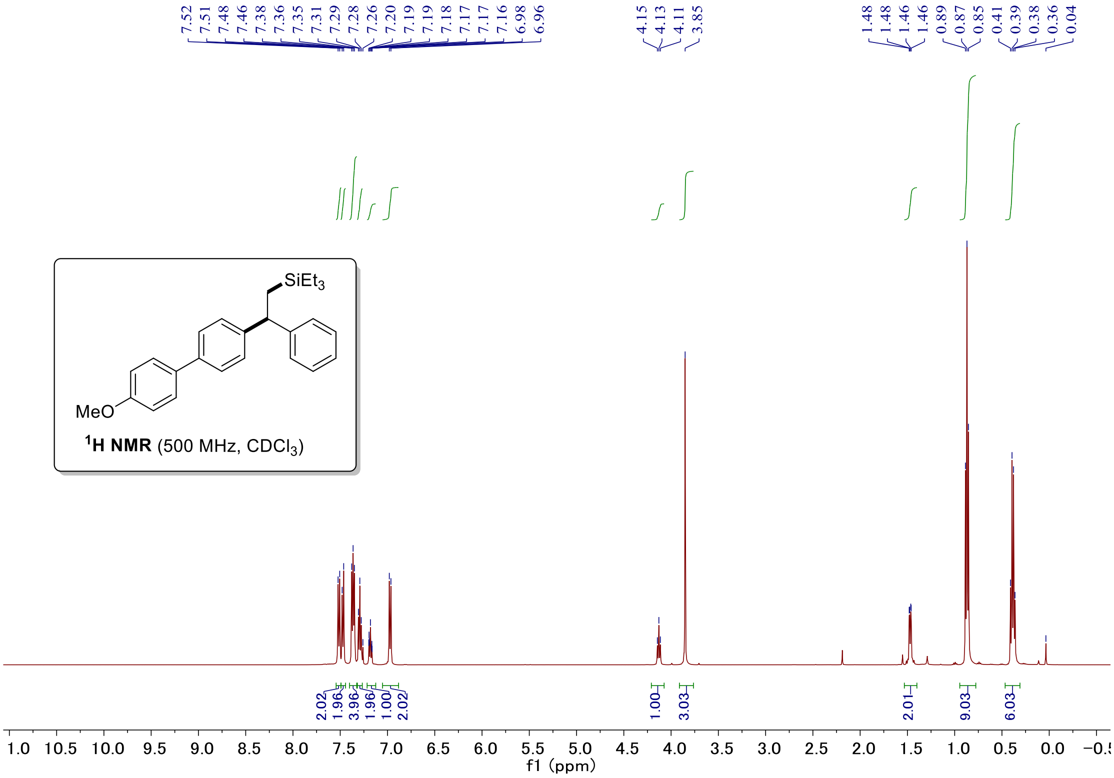

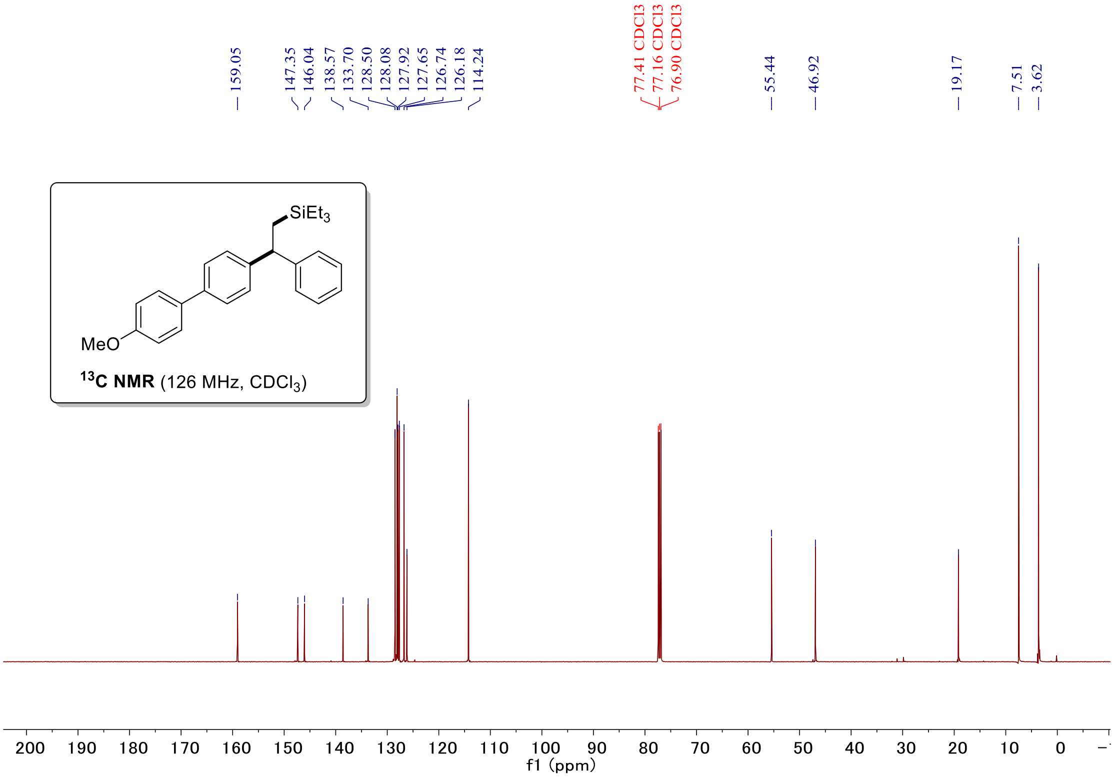

(2-(4'-(Benzyloxy)-biphenyl-4-yl)-2-phenylethyl)triethylsilane (4ia)

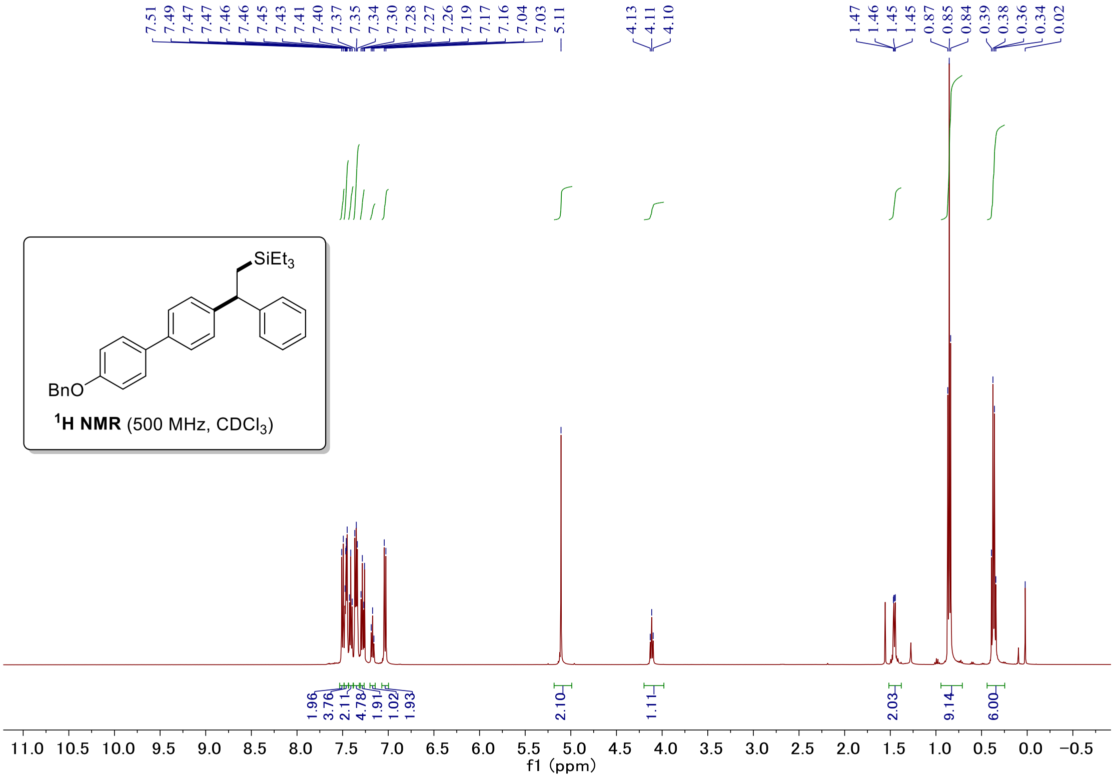

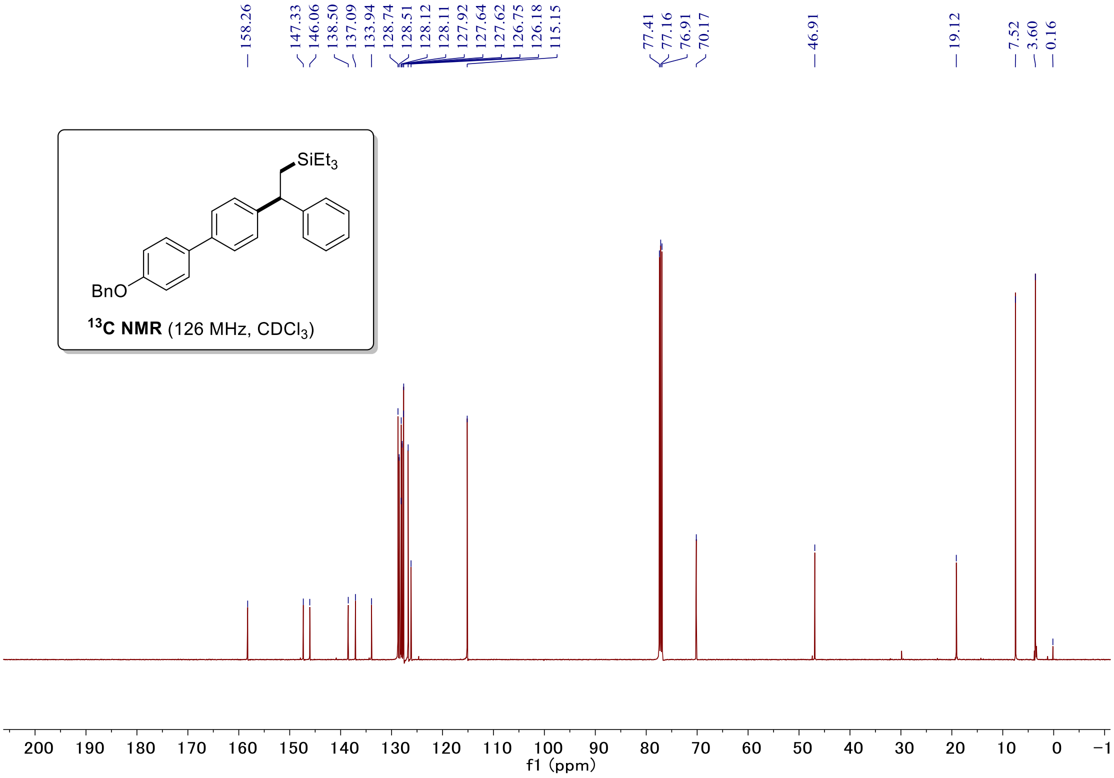

(2-(4-(Benzo[*d*][1,3]dioxol-5-yl)phenyl)-2-phenylethyl)triethylsilane (4ja)

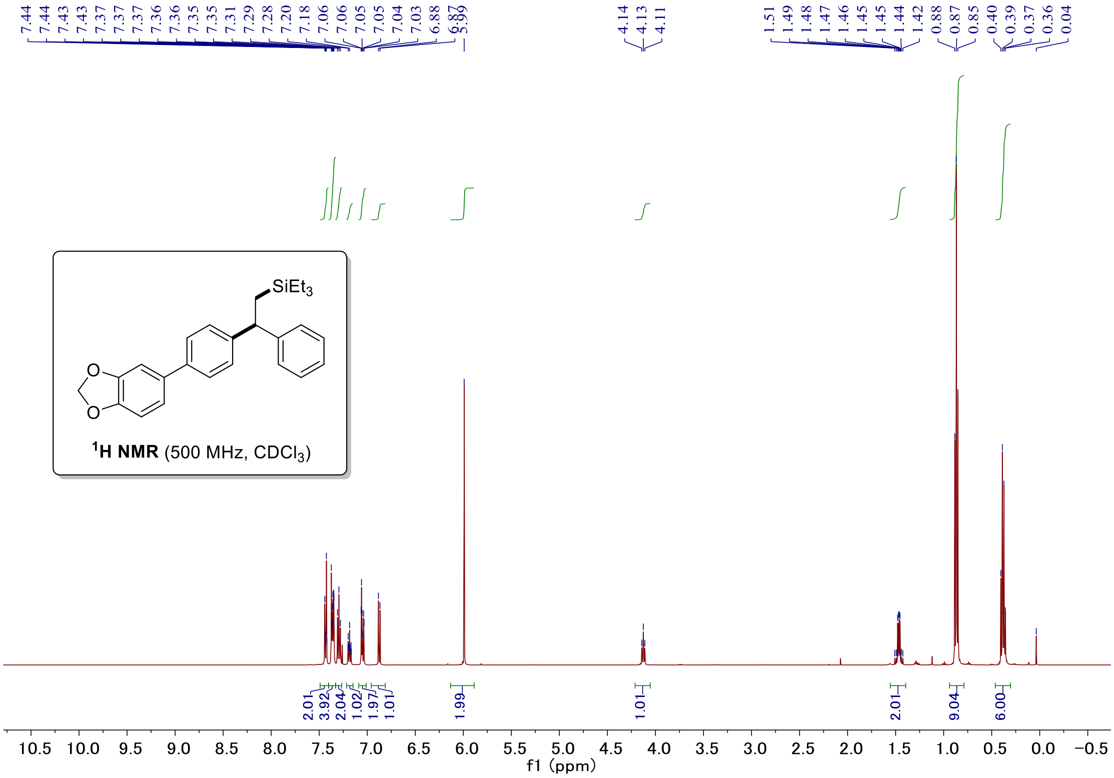

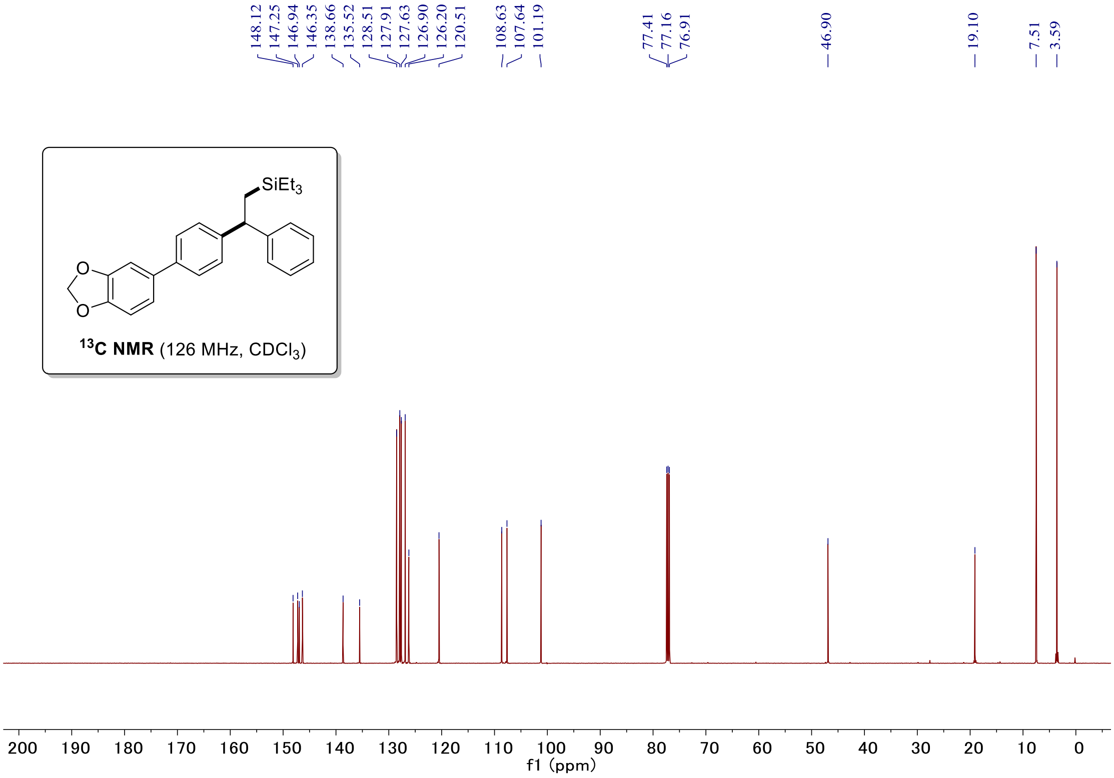

Triethyl(2-phenyl-2-(3'-(trifluoromethyl)-biphenyl-4-yl)ethyl)silane (4ka)

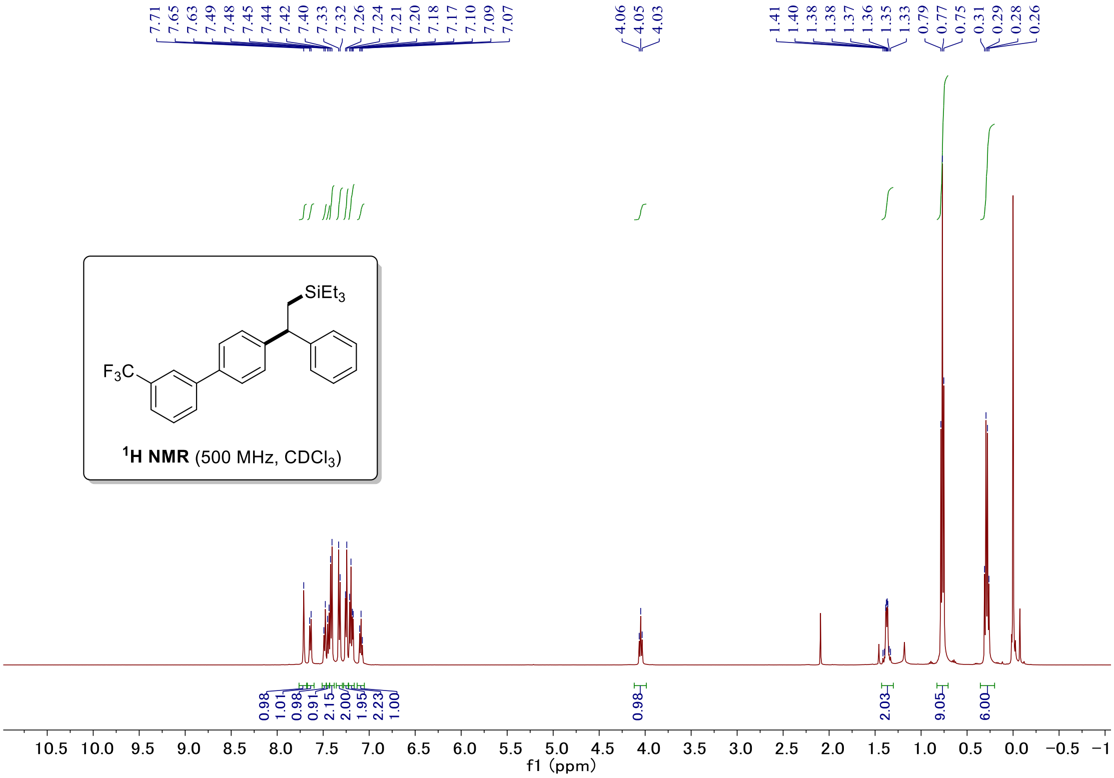

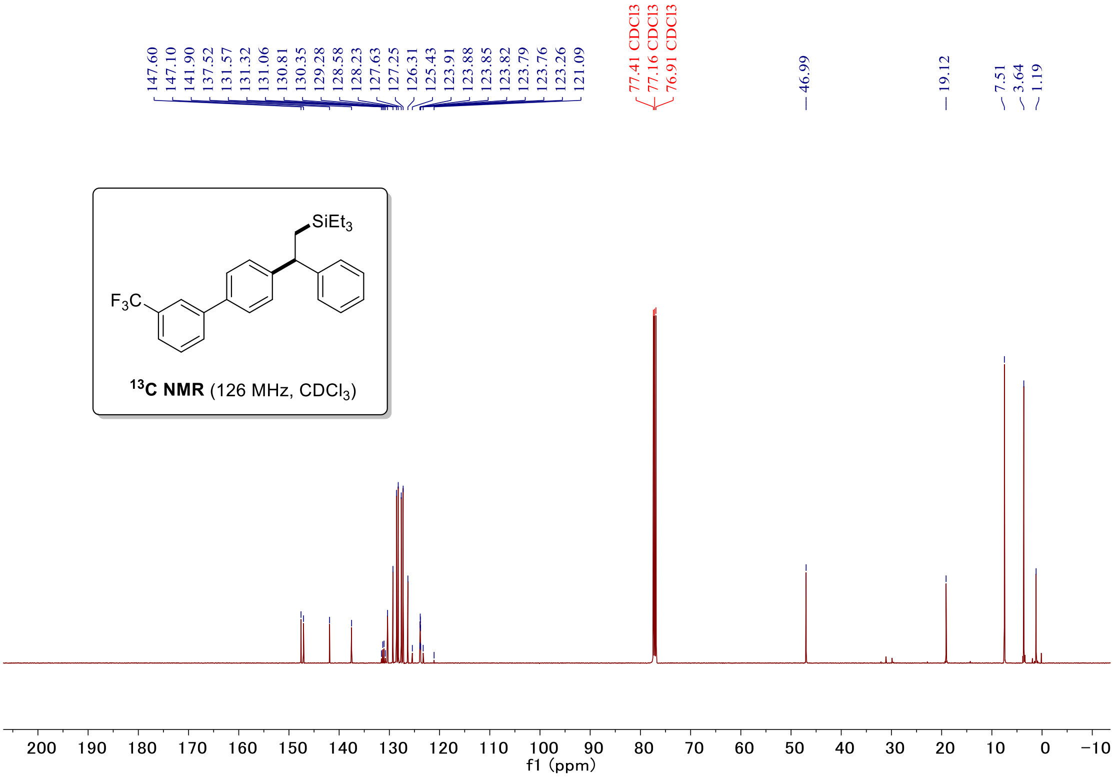

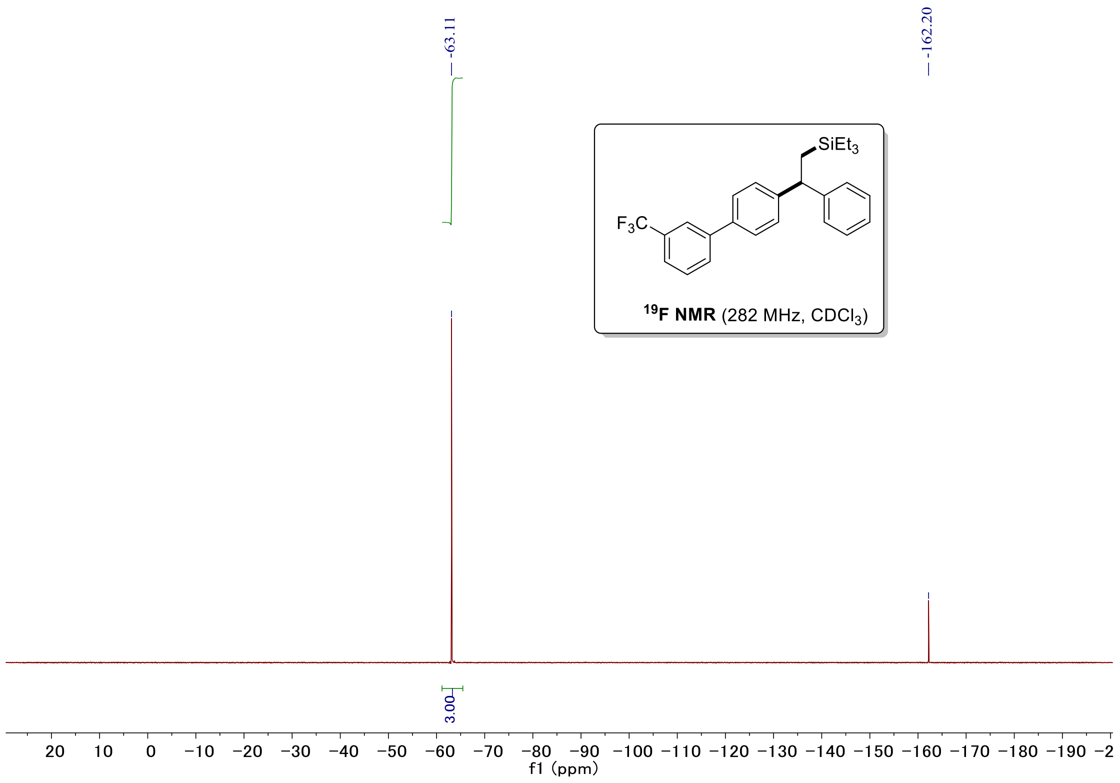

Triethyl(2-phenyl-2-(4'-(trifluoromethyl)-biphenyl-4-yl)ethyl)silane (4la)

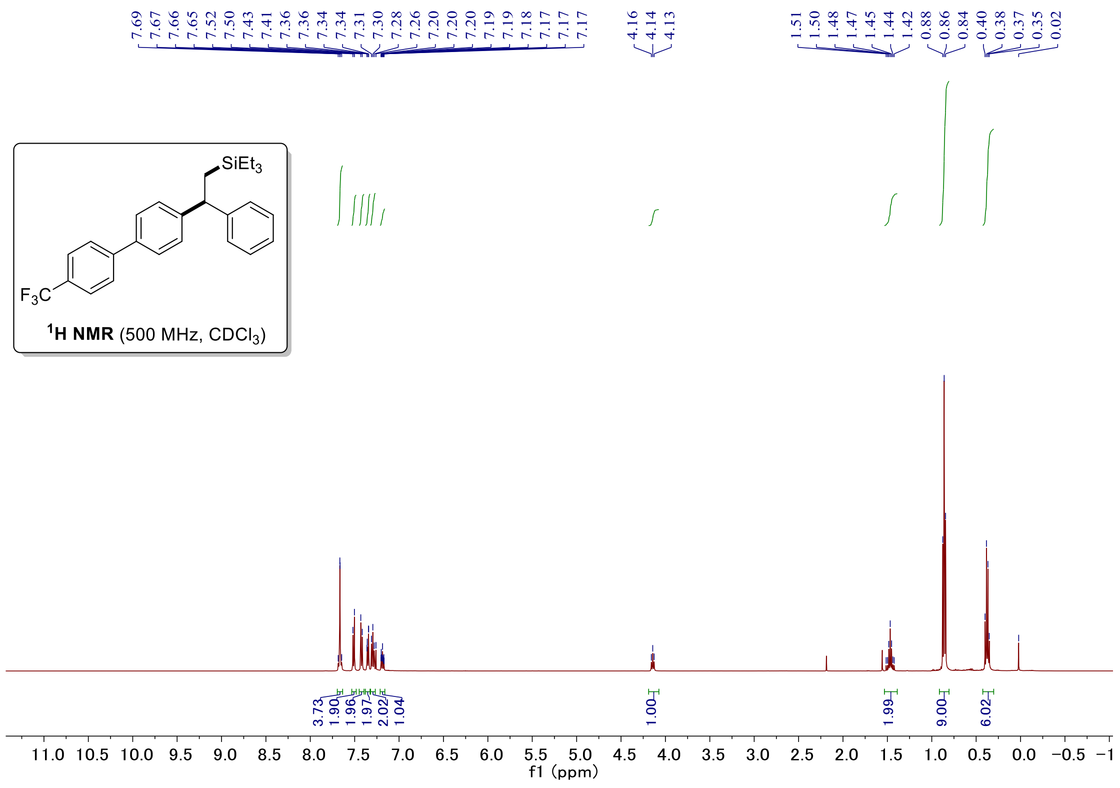

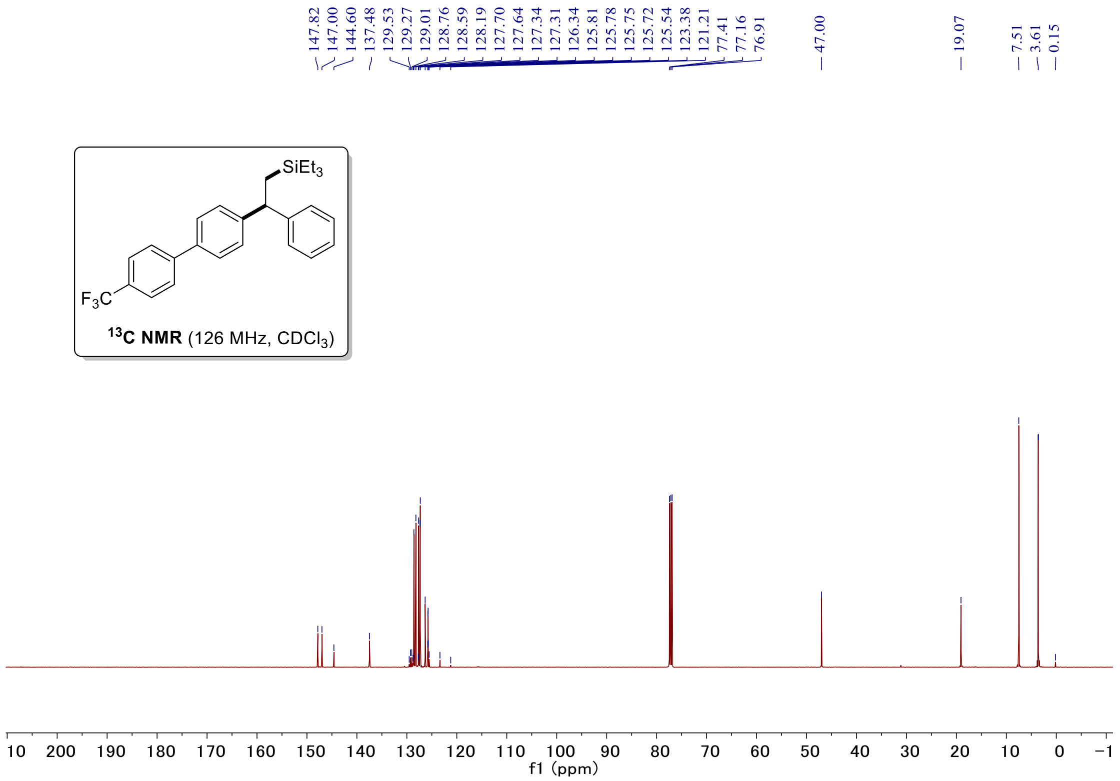
147.82
147.00
144.60
137.48
129.53
129.27
129.01
128.76
128.59
128.19
127.70
127.64
127.34
127.31
126.34
125.81
125.78
125.75
125.72
125.54
123.38
121.21
77.41
77.16
76.91
47.00
19.07
7.51
3.61
0.15
SiEt3
F3C
13C NMR (126 MHz, CDCl3)
f1 (ppm)

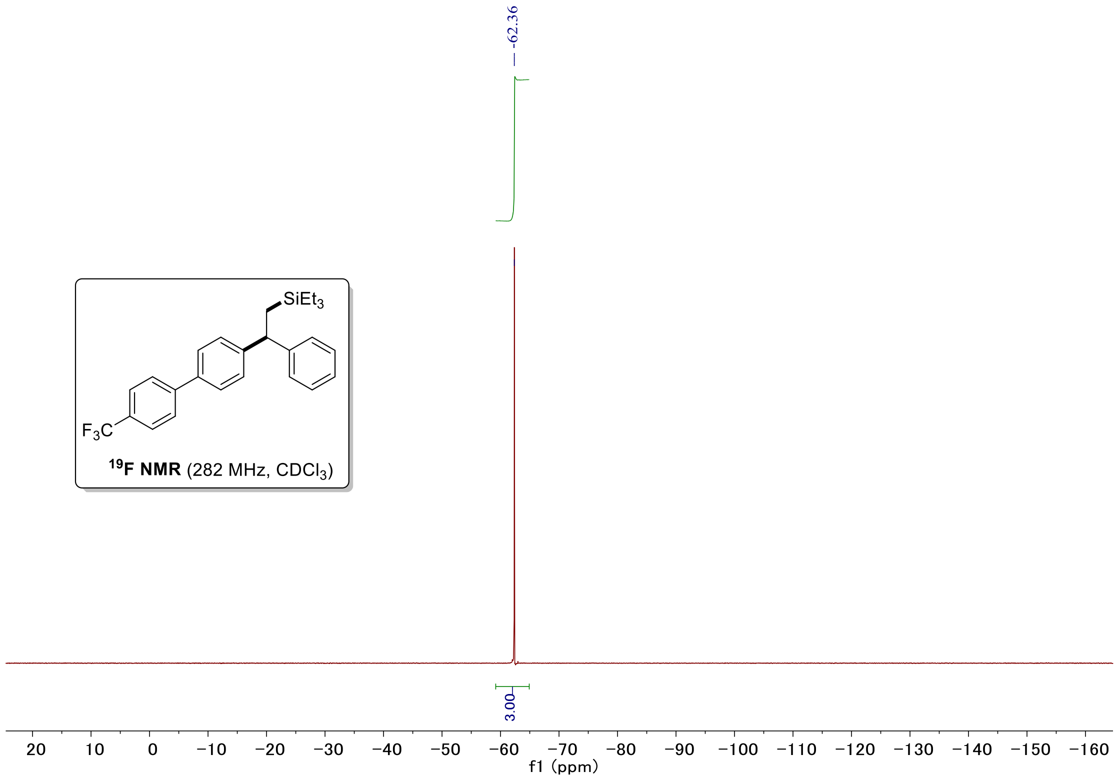
-62.36
SiEt3
F3C
19F NMR (282 MHz, CDCl3)
3.00
f1 (ppm)

(2-(4'-Chloro-biphenyl-4-yl)-2-phenylethyl)triethylsilane (4ma)

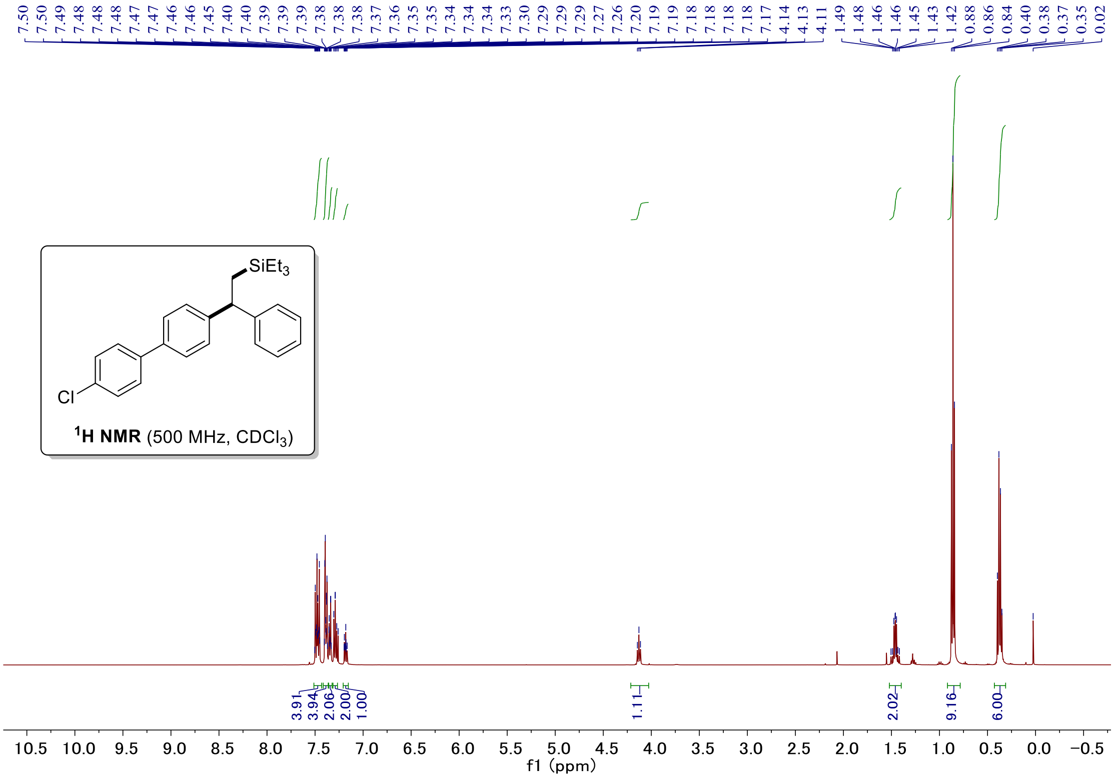

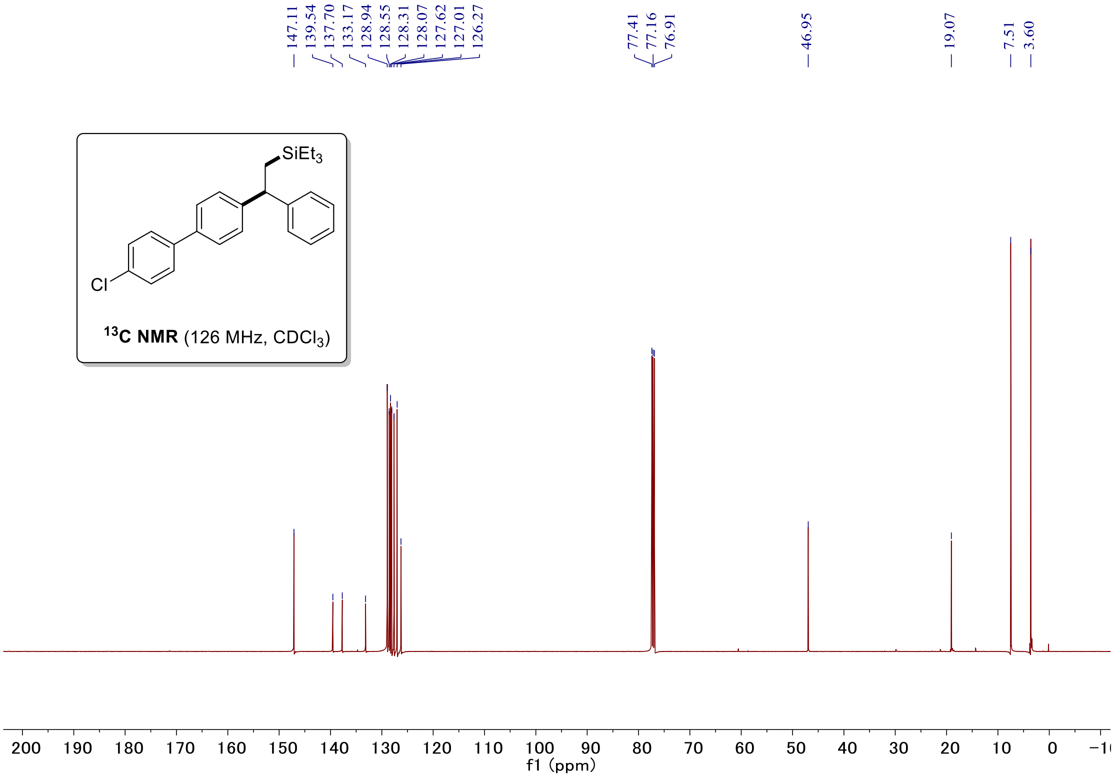

1-Methyl-6-(1-phenyl-2-(triethylsilyl)ethyl)-1*H*-indole (4na)

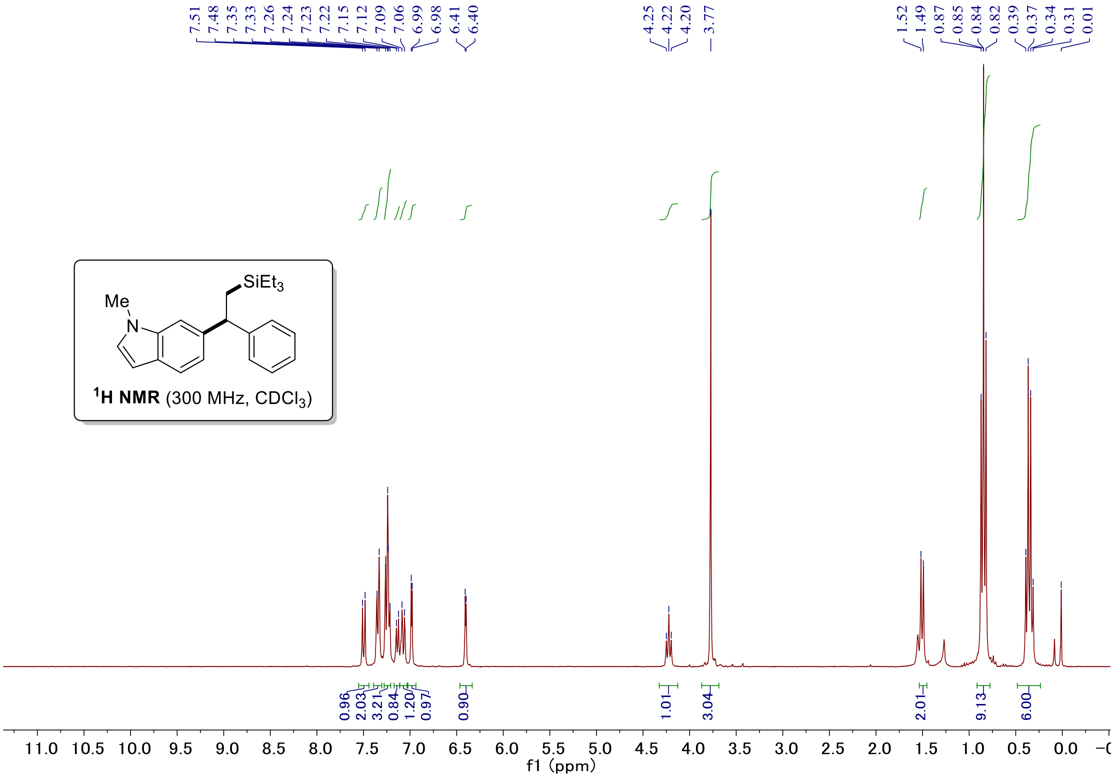

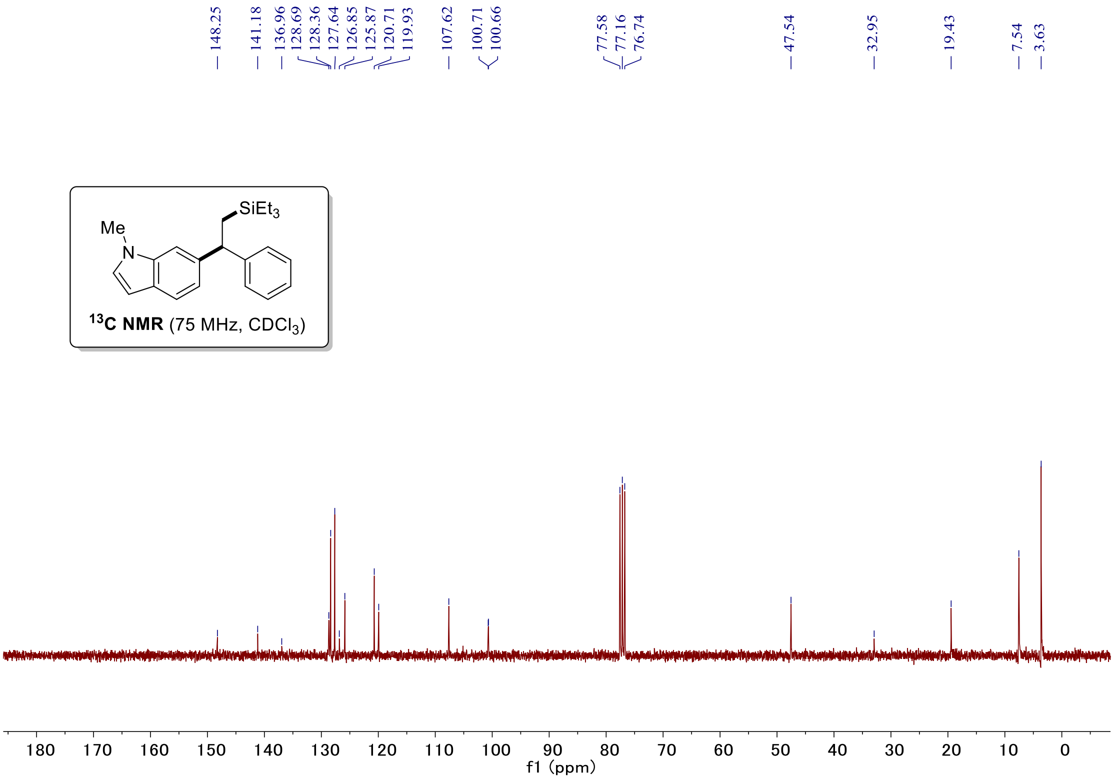

1-(4-(1-Phenyl-2-(triethylsilyl)ethyl)phenyl)-1*H*-pyrrole (4oa)

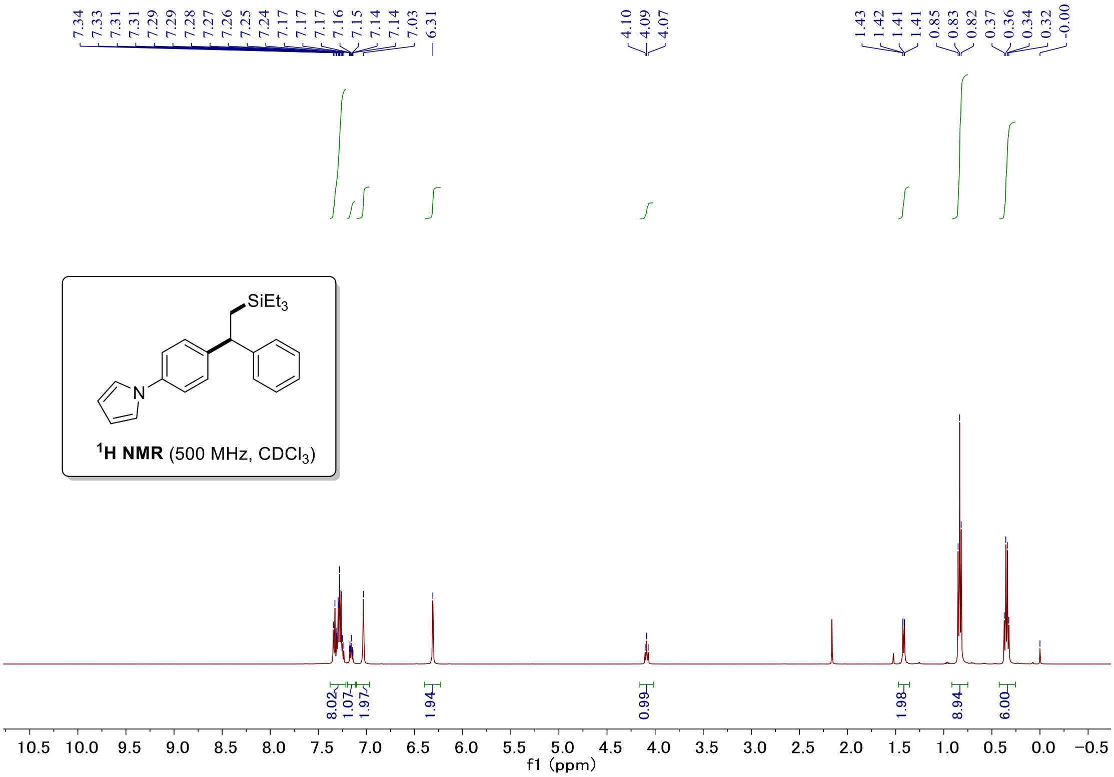

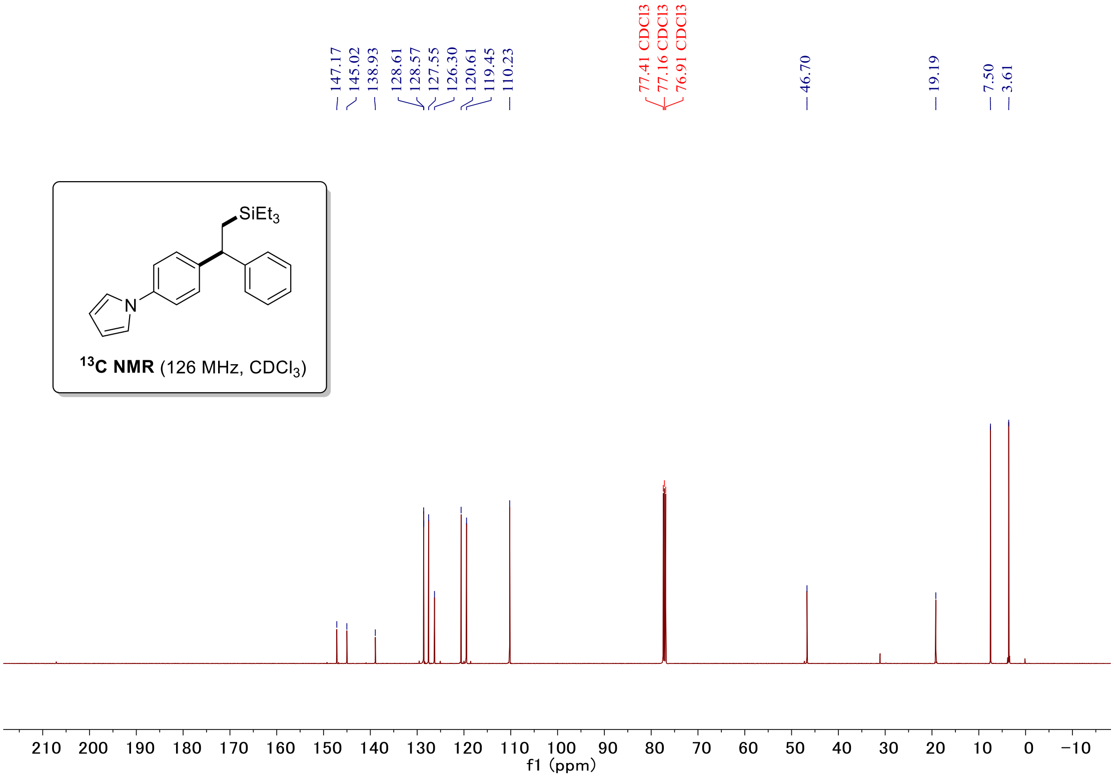

4-(4-(1-Phenyl-2-(triethylsilyl)ethyl)phenyl)pyridine (4pa)

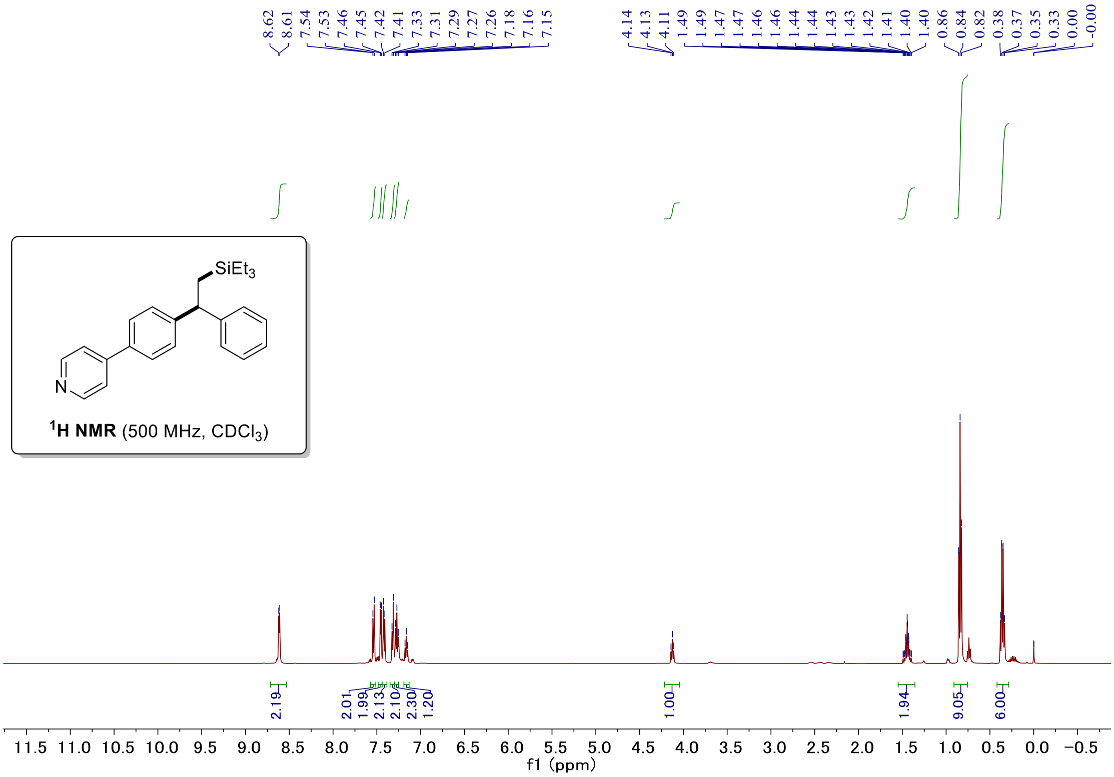

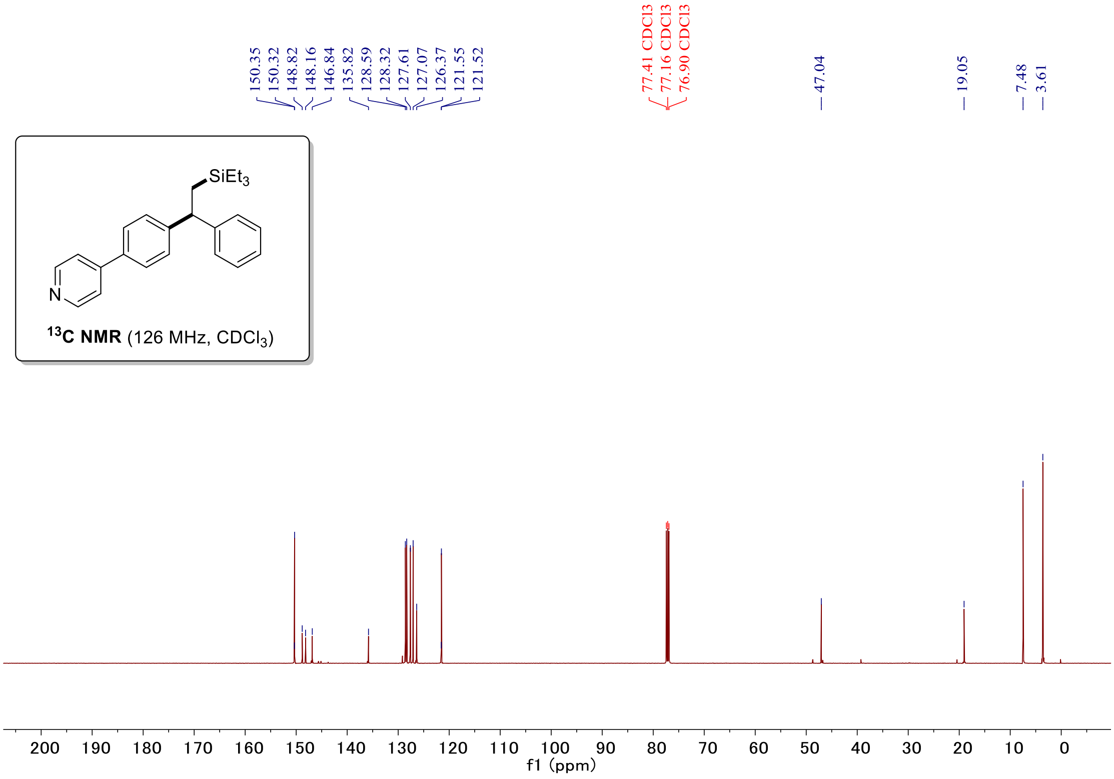

2-Phenyl-5-(1-phenyl-2-(triethylsilyl)ethyl)pyridine (4qa)

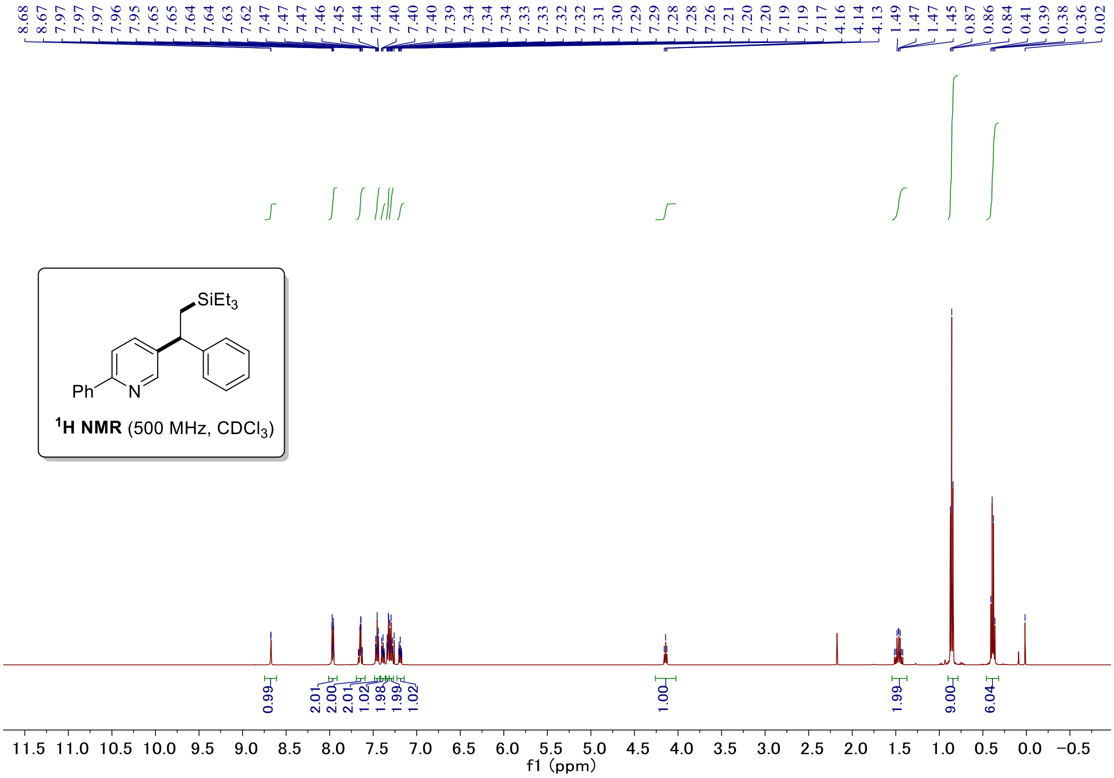

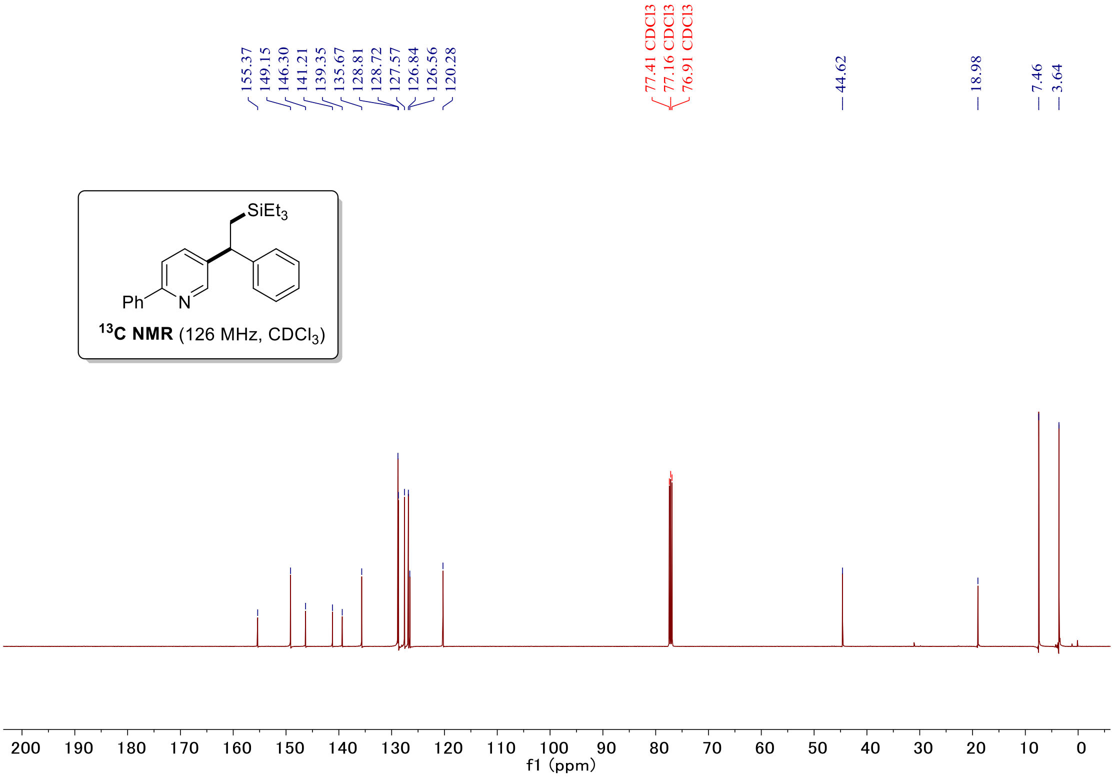

5-Phenyl-2-(1-phenyl-2-(triethylsilyl)ethyl)pyridine (4ra)

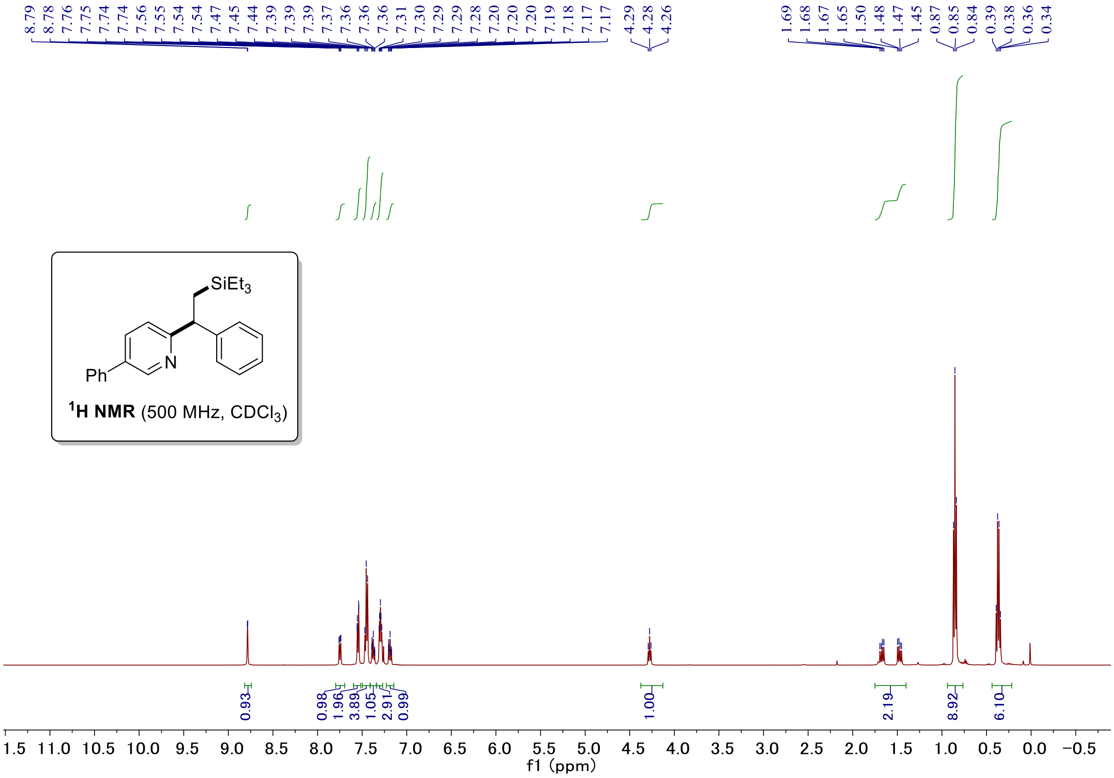

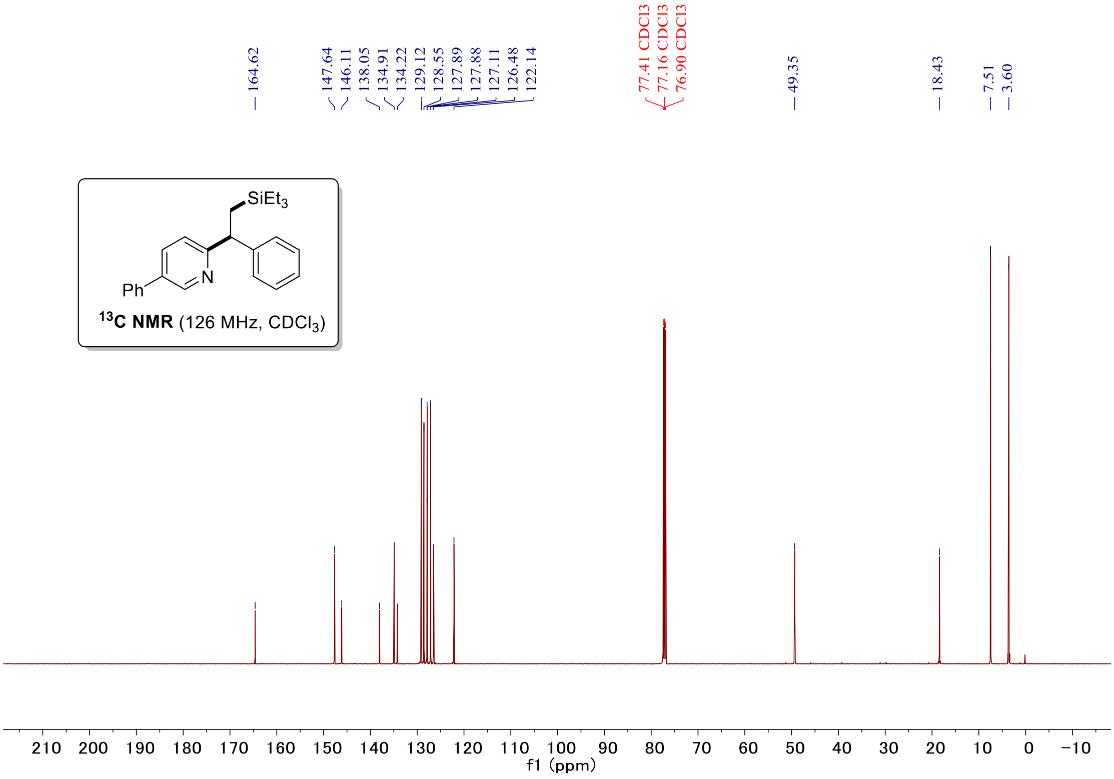

2-Phenyl-4-(1-phenyl-2-(triethylsilyl)ethyl)pyridine (4sa)

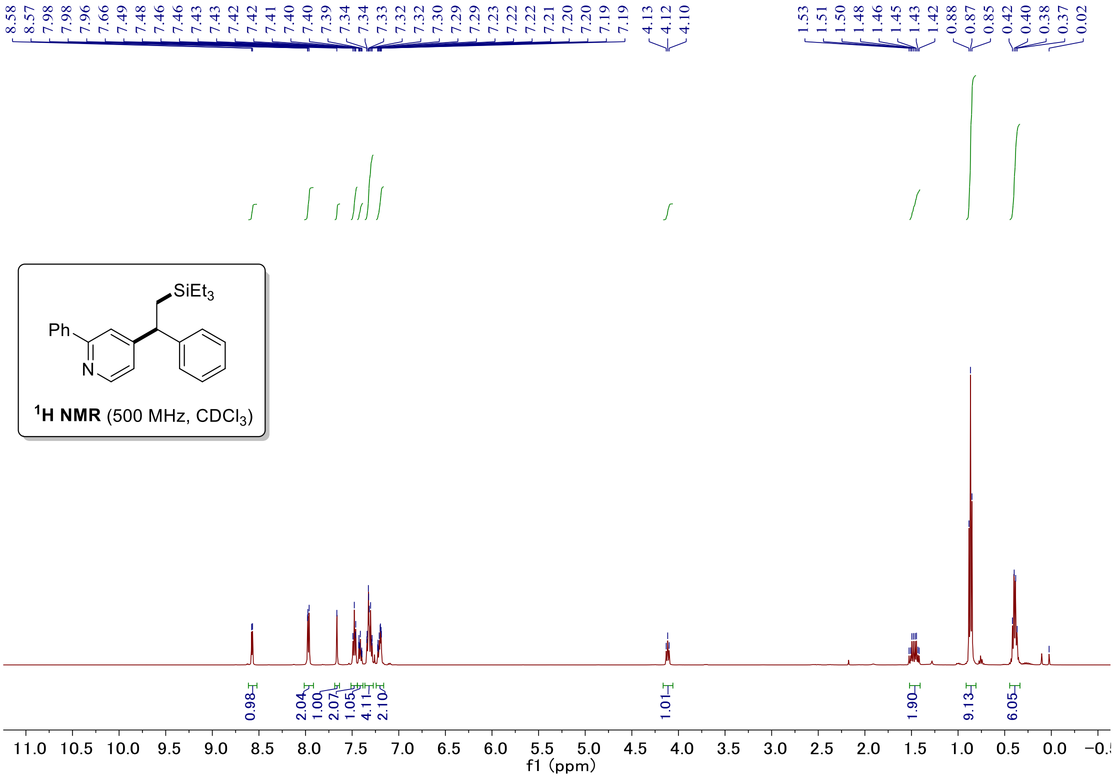

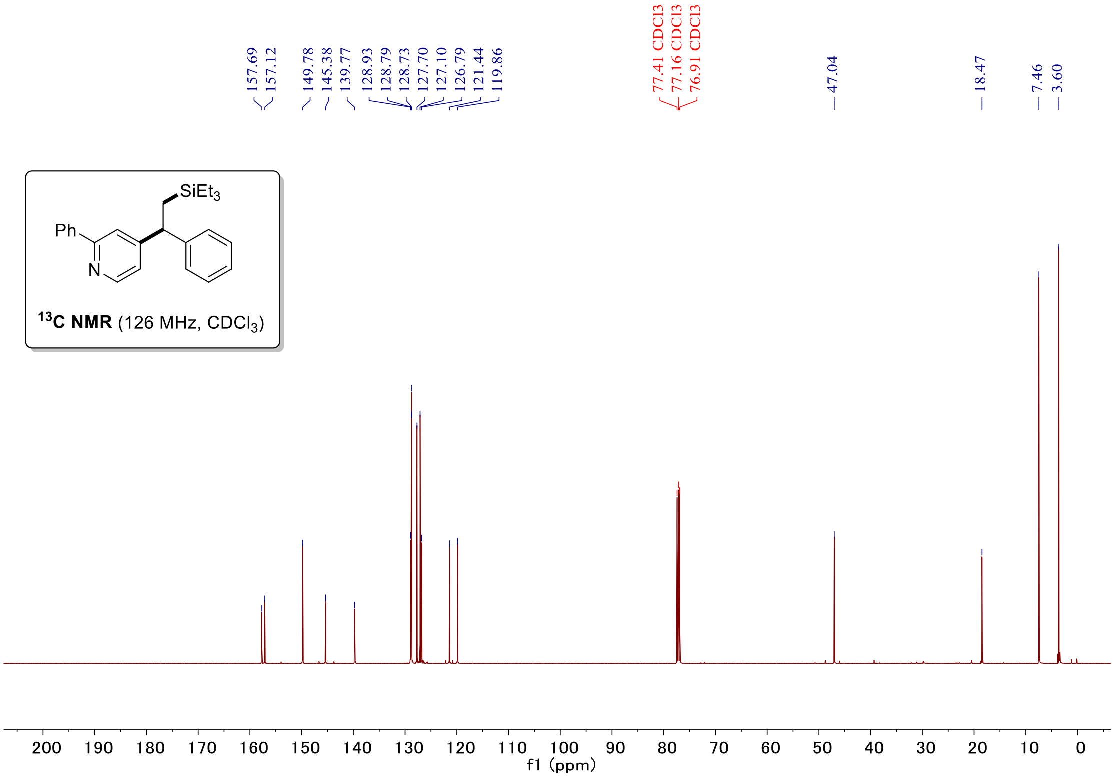

1-Methyl-2-(4-(1-phenyl-2-(triethylsilyl)ethyl)phenyl)-1*H*-indole (4ta)

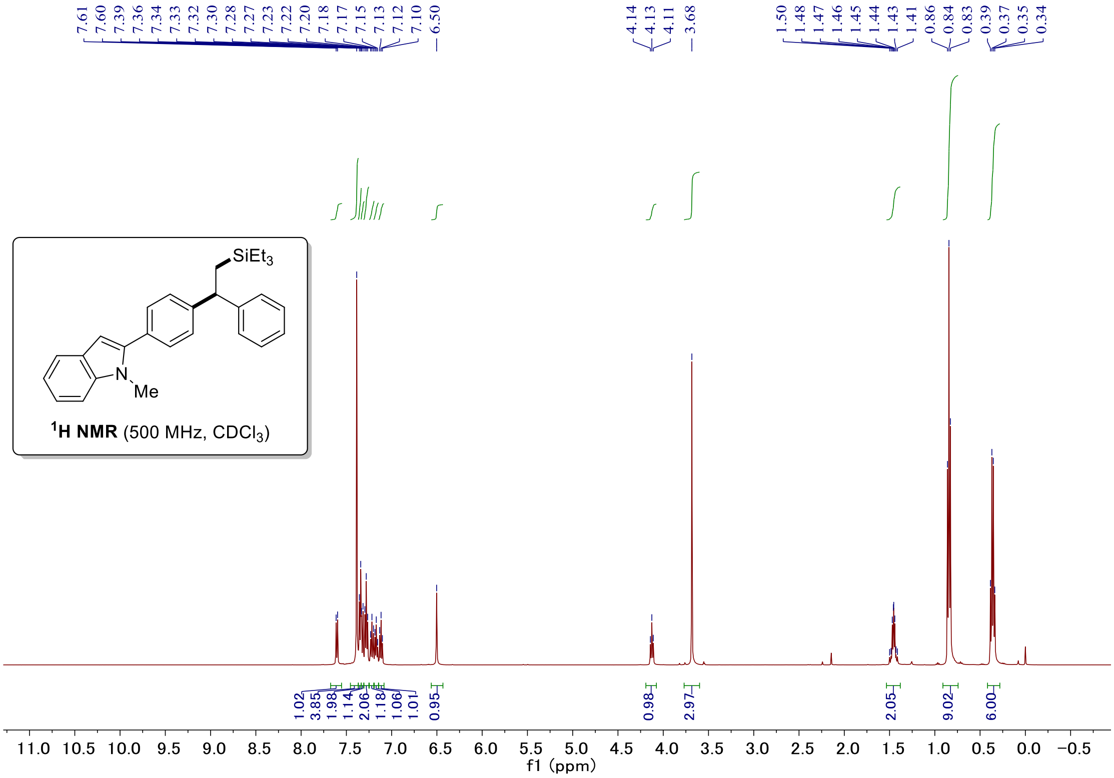

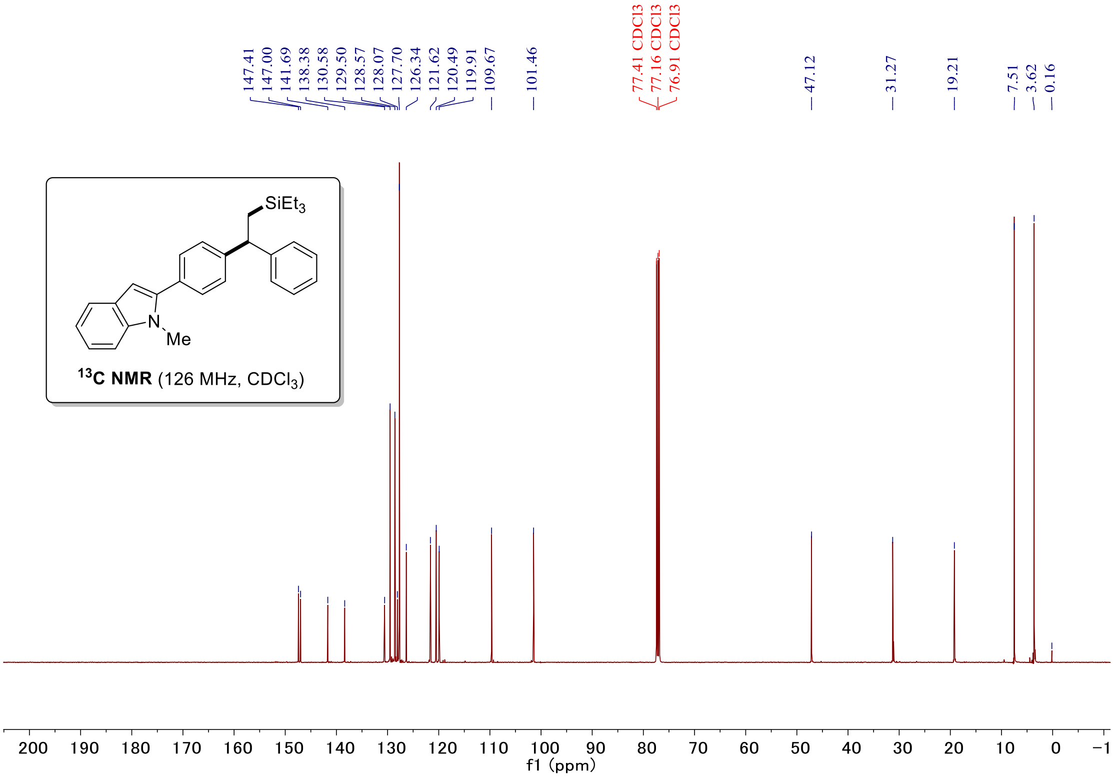

(2-(4-(Benzofuran-3-yl)phenyl)-2-phenylethyl)triethylsilane (4ua)

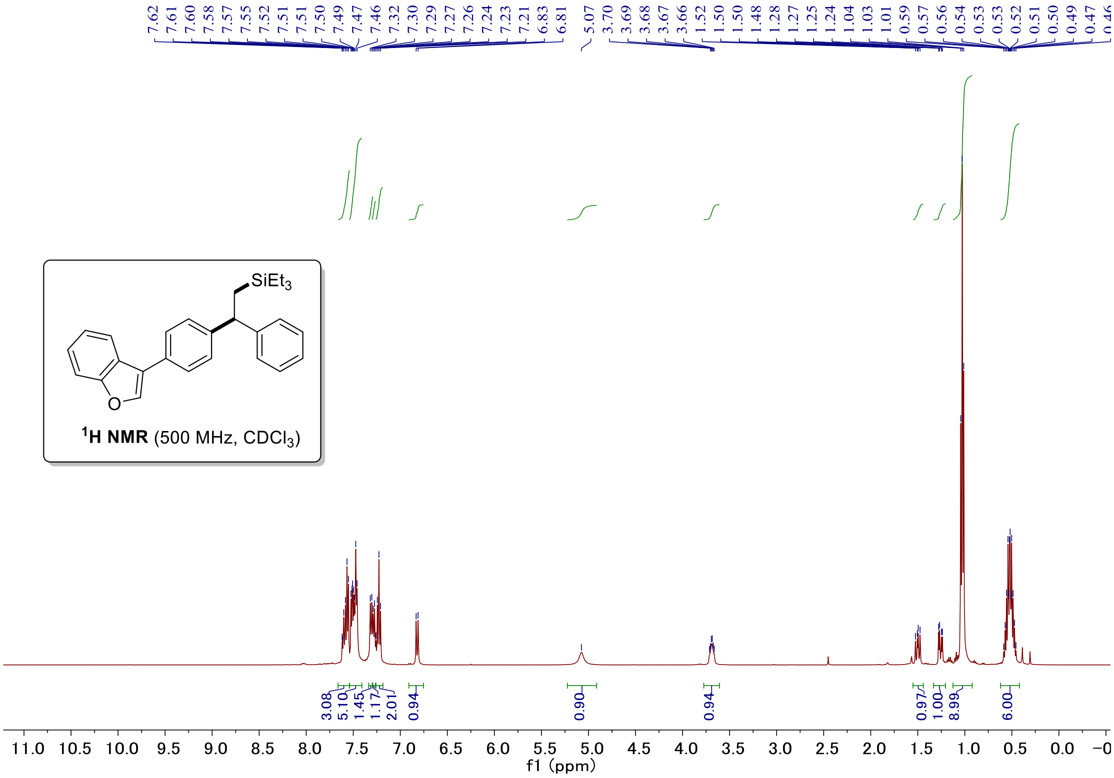

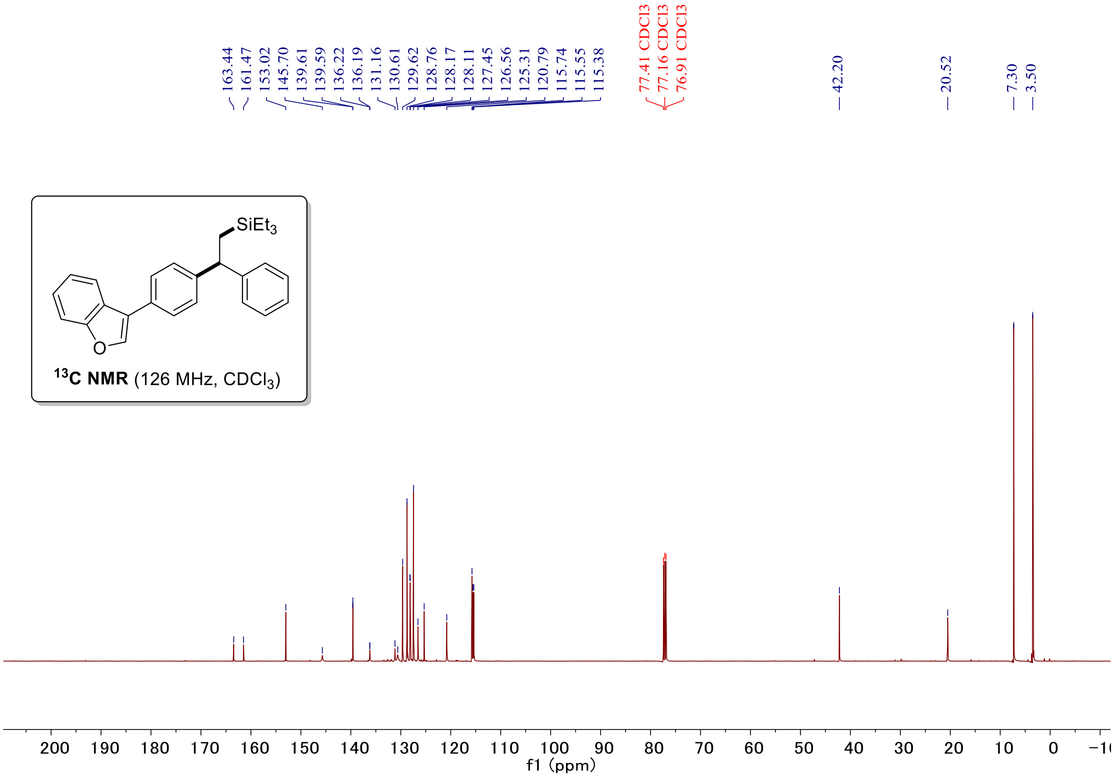

(2-(Biphenyl-4-yl)-2-phenylethyl)dimethyl(phenyl)silane (4aa`)

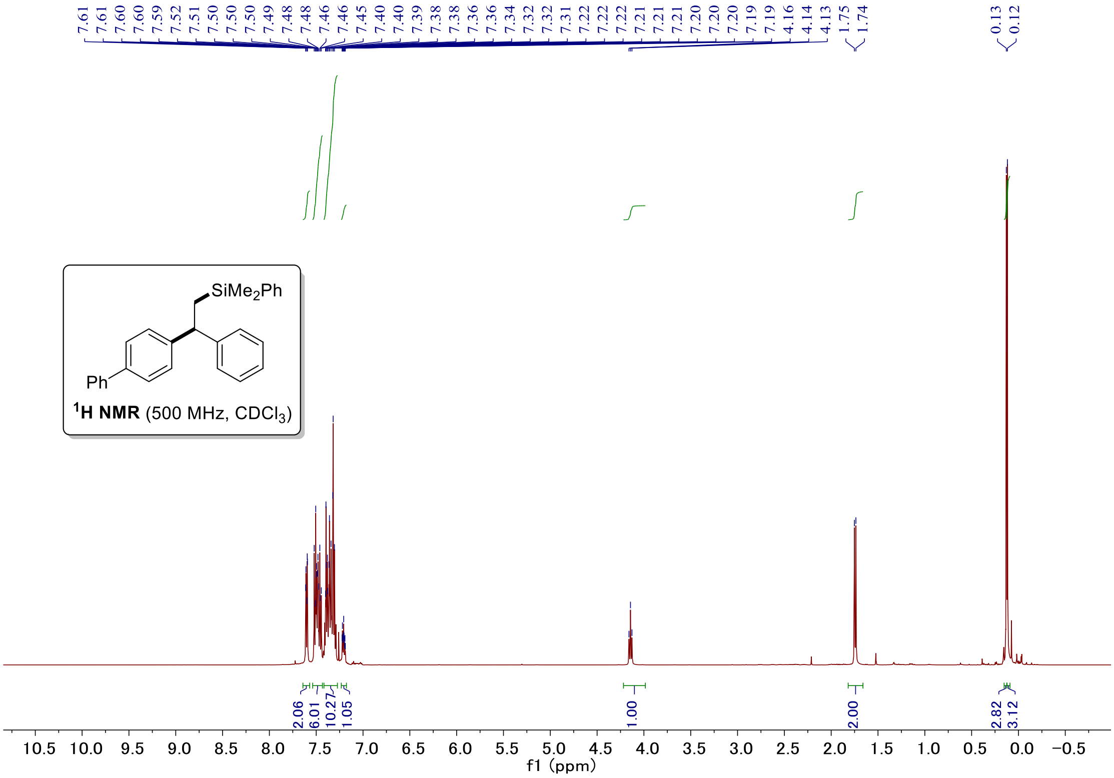

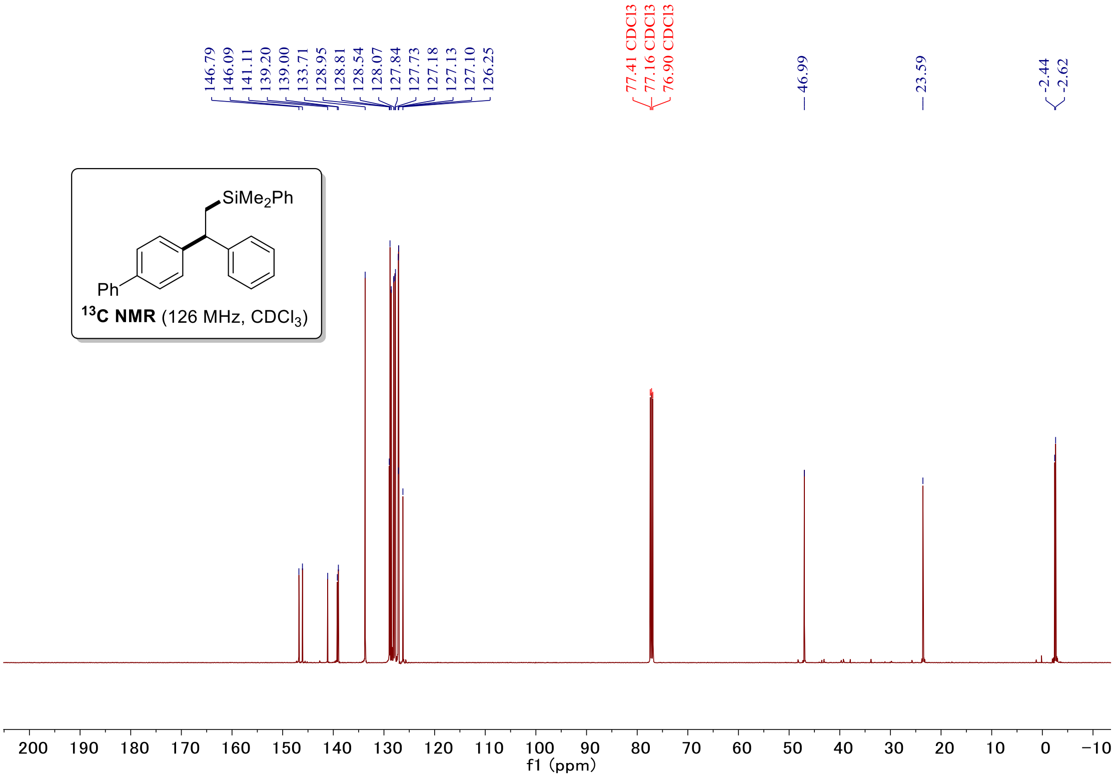

(2-(Biphenyl-4-yl)-2-phenylethyl)tripropylsilane (4aa``)

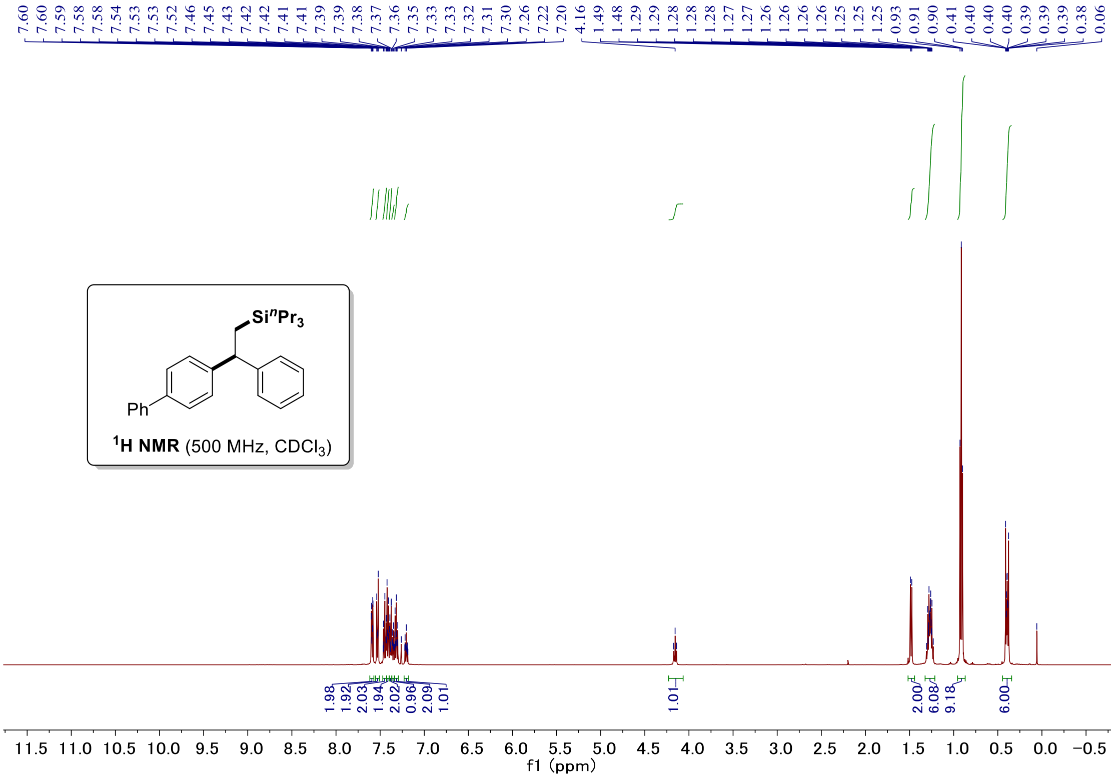

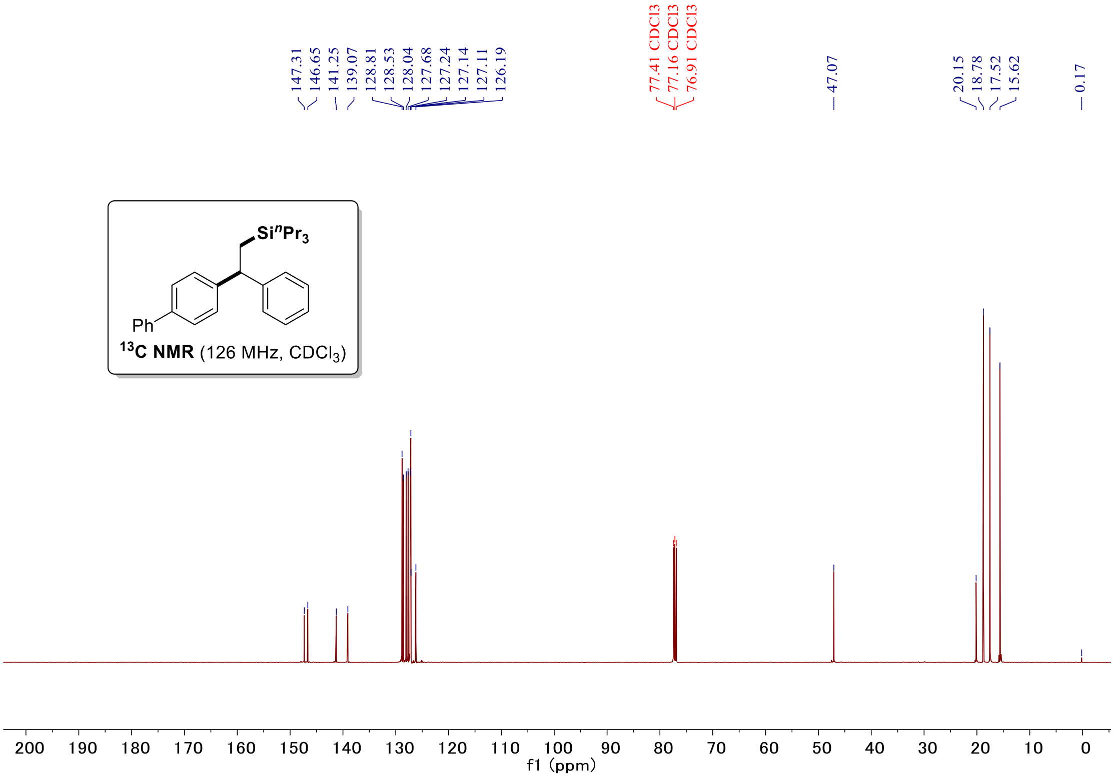

(2-(Biphenyl-4-yl)-2-phenylethyl)(*tert*-butyl)dimethylsilane (4aa```)

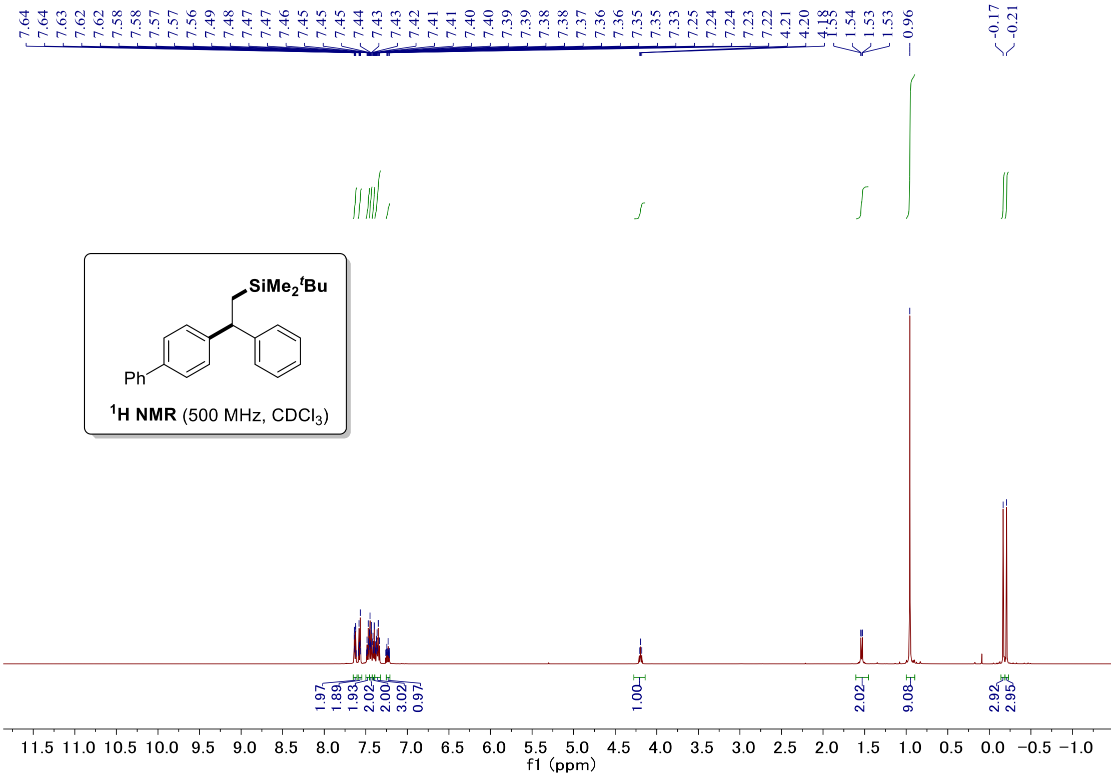

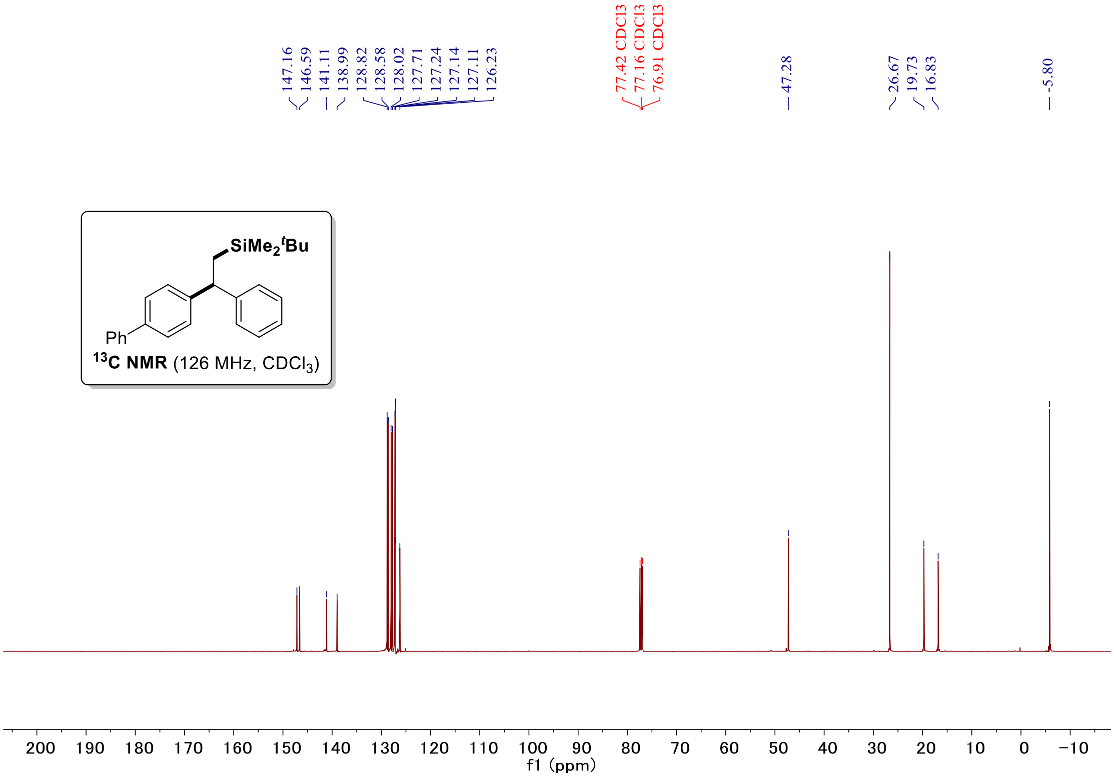

(2-(Biphenyl-4-yl)-2-(naphthalen-2-yl)ethyl)triethylsilane (4ab)

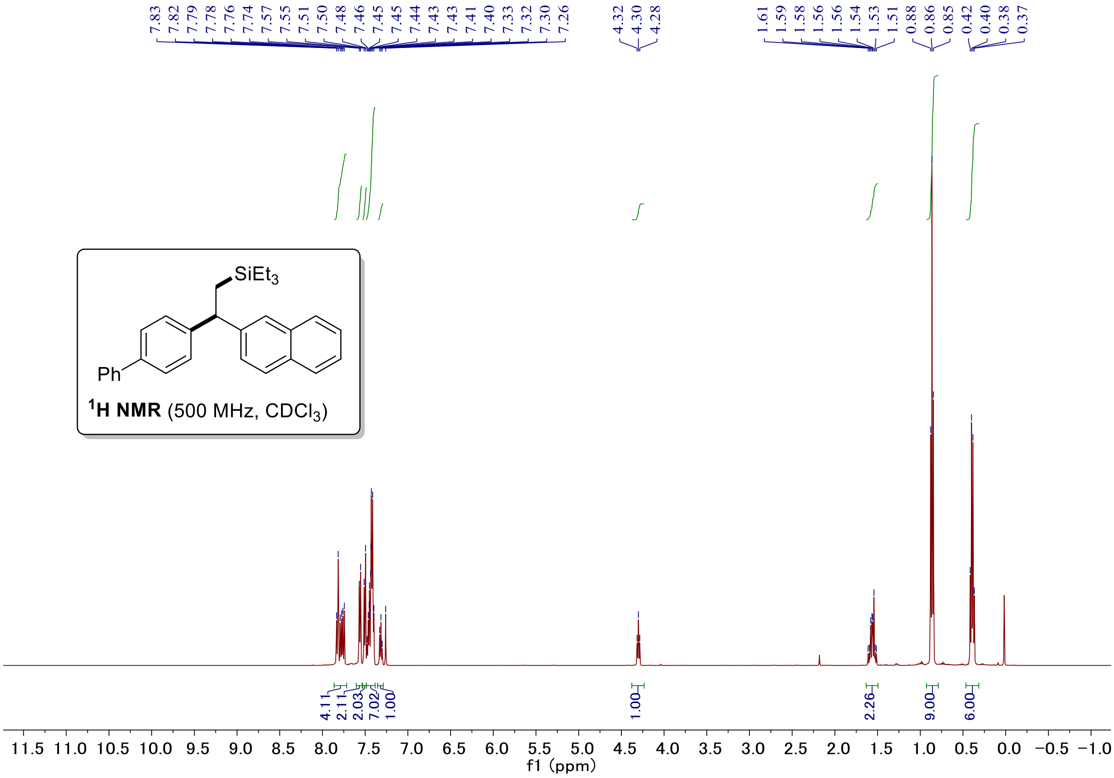

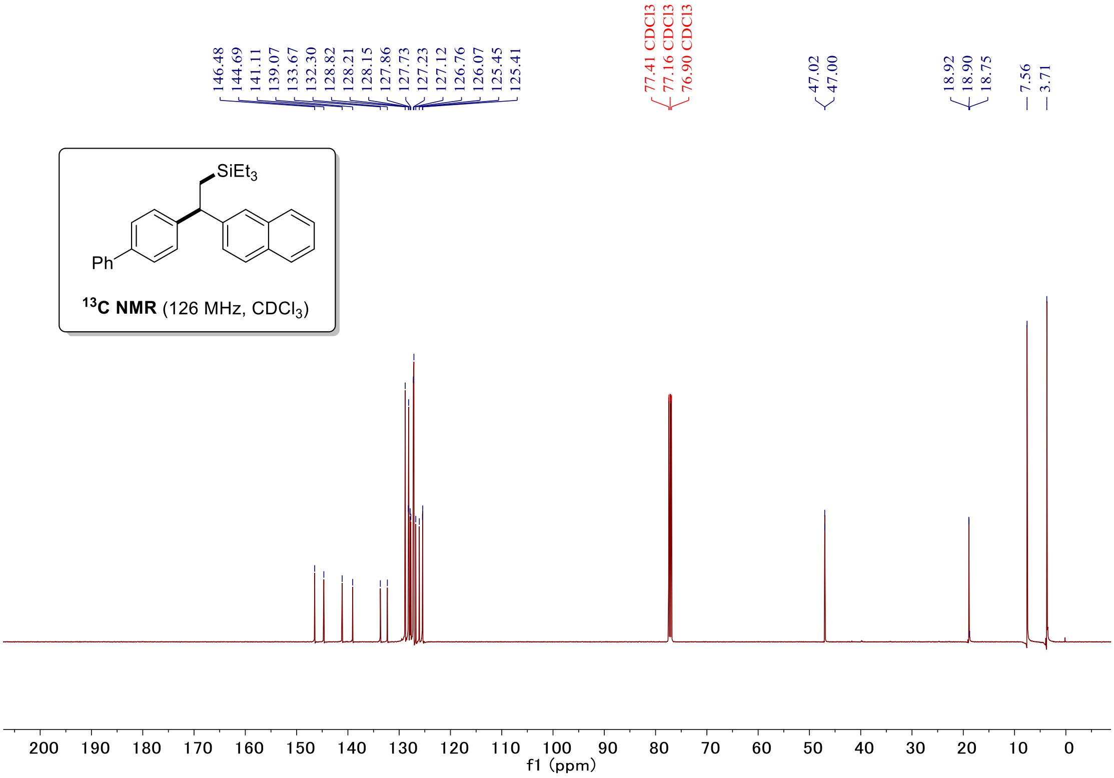

(2-(Biphenyl-4-yl)-2-(*p*-tolyl)ethyl)triethylsilane (4ac)

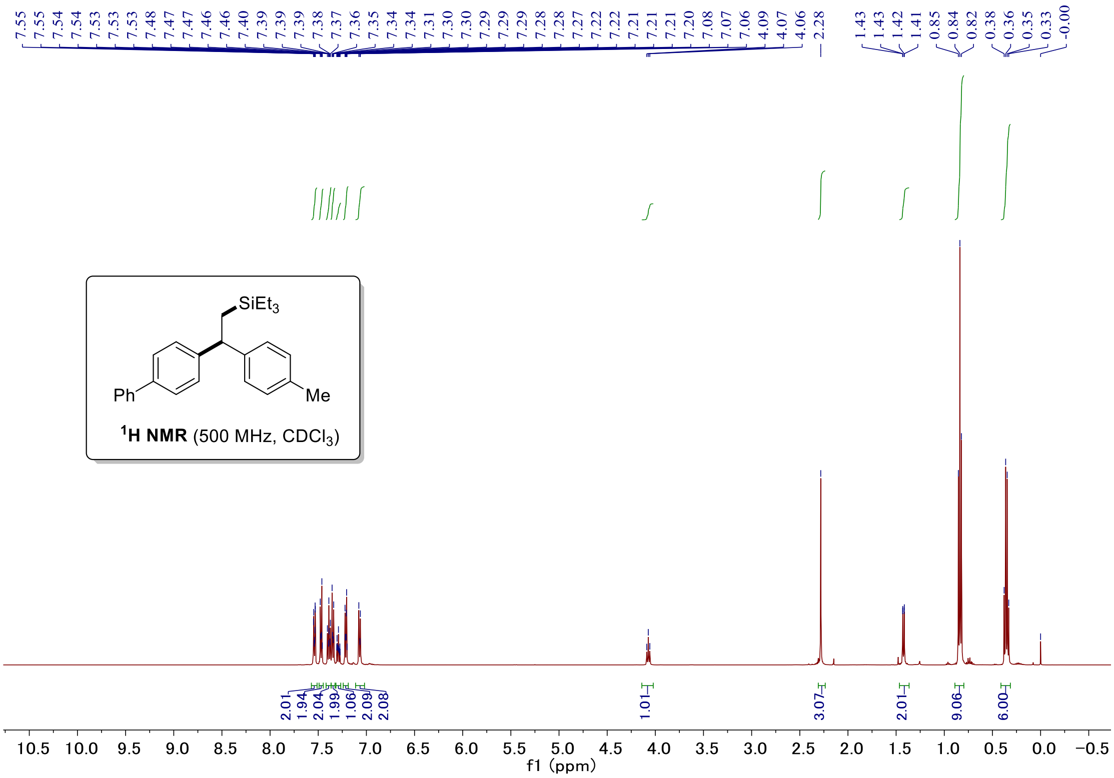

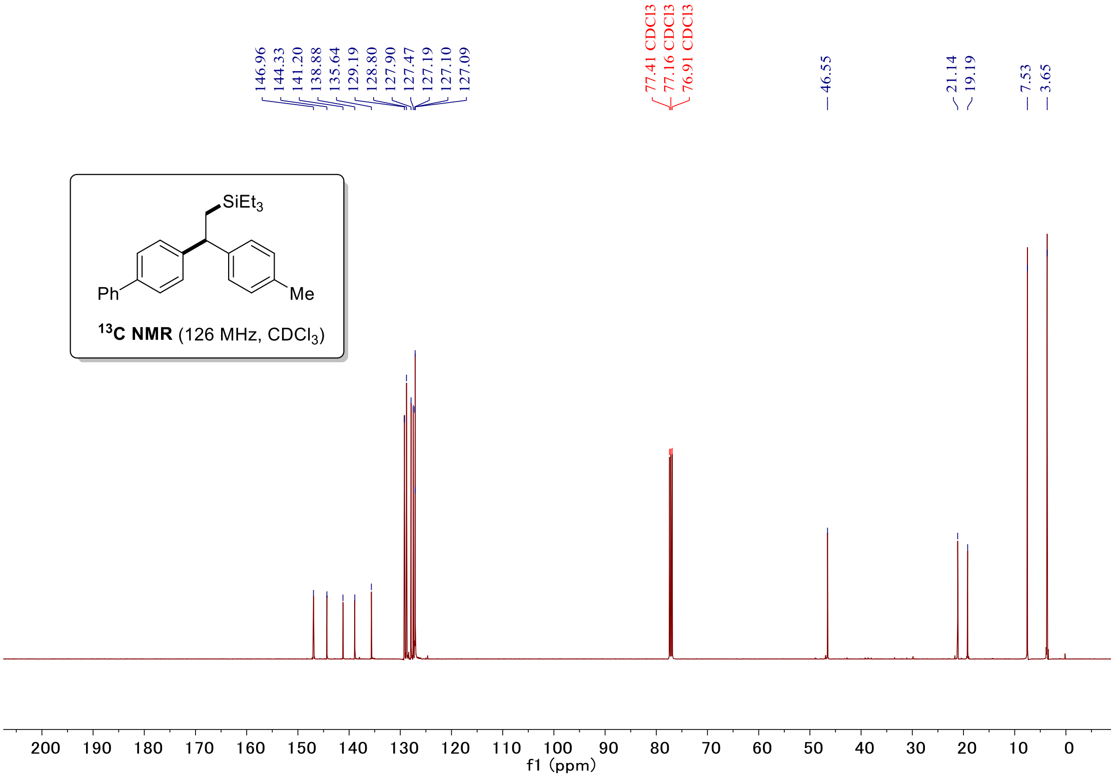

(2-(Biphenyl-4-yl)-2-(4-(*tert*-butyl)phenyl)ethyl)triethylsilane (4ad)

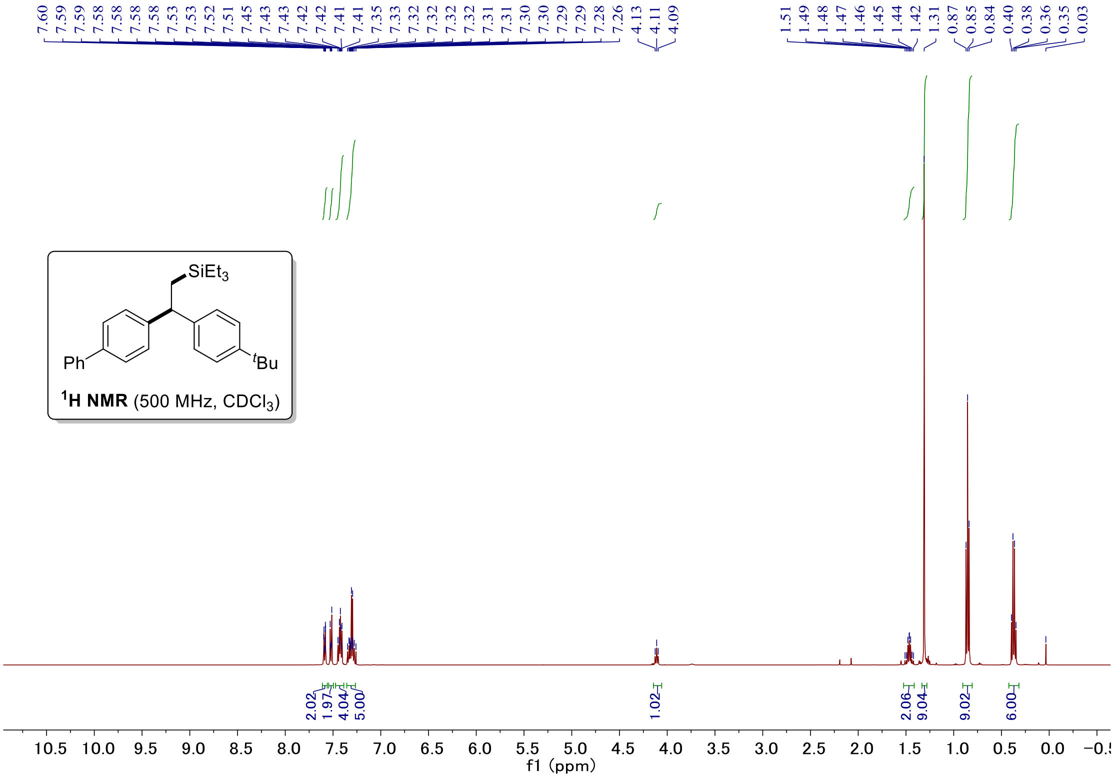

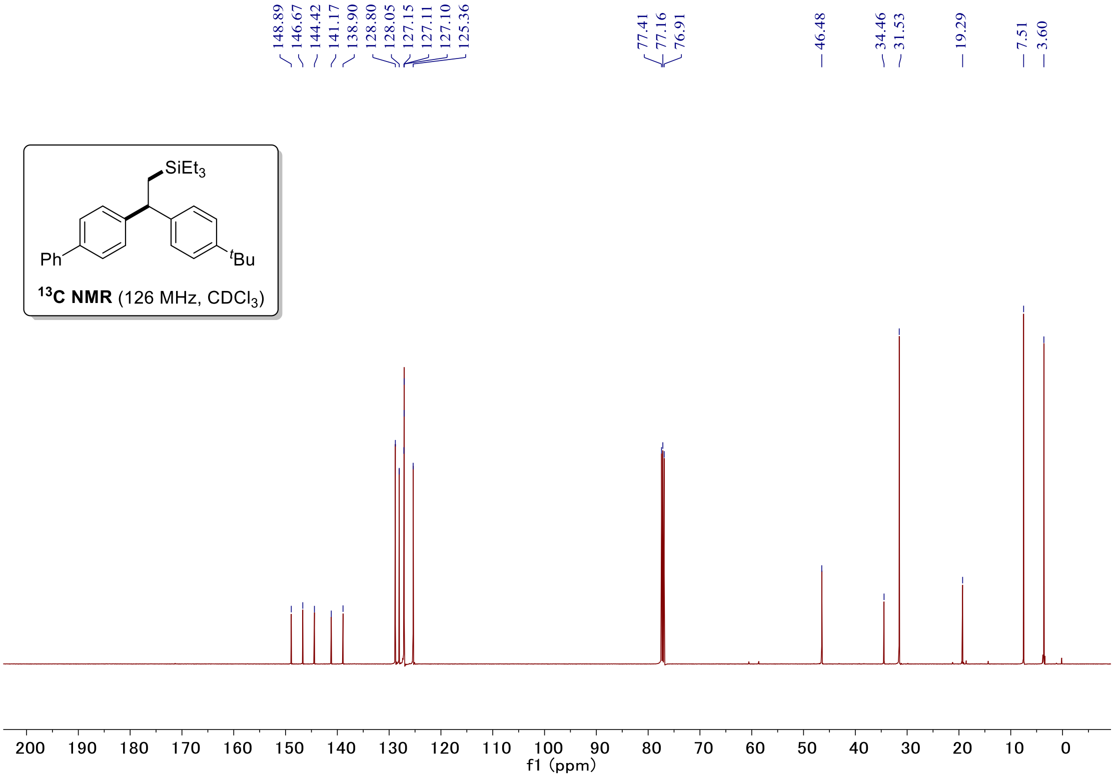

(2,2-Di(biphenyl-4-yl)ethyl)triethylsilane (4ae)

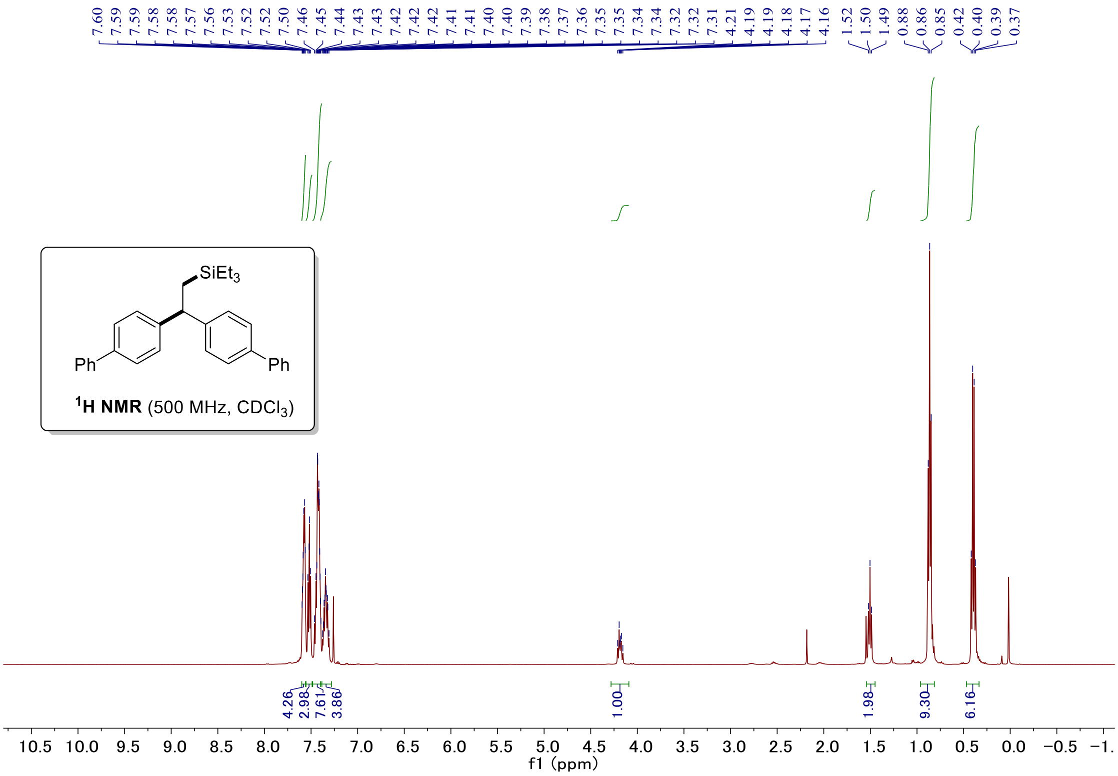

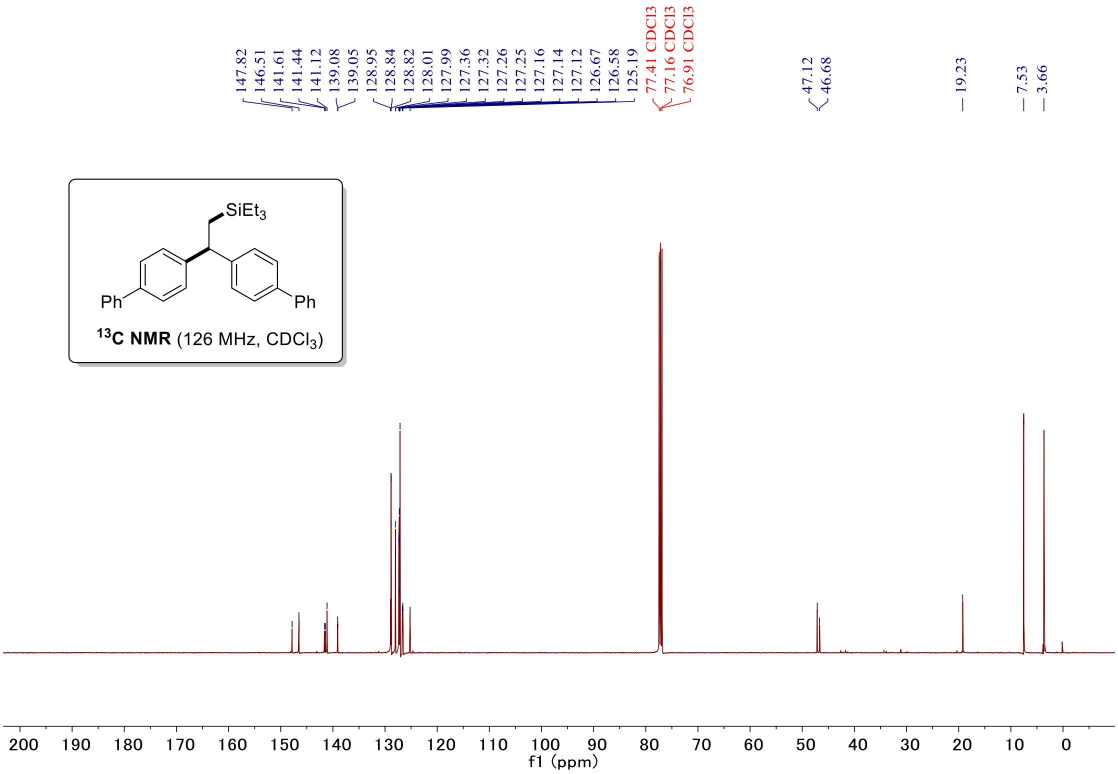

(2-(Biphenyl-4-yl)-2-(4-methoxyphenyl)ethyl)triethylsilane (4af)

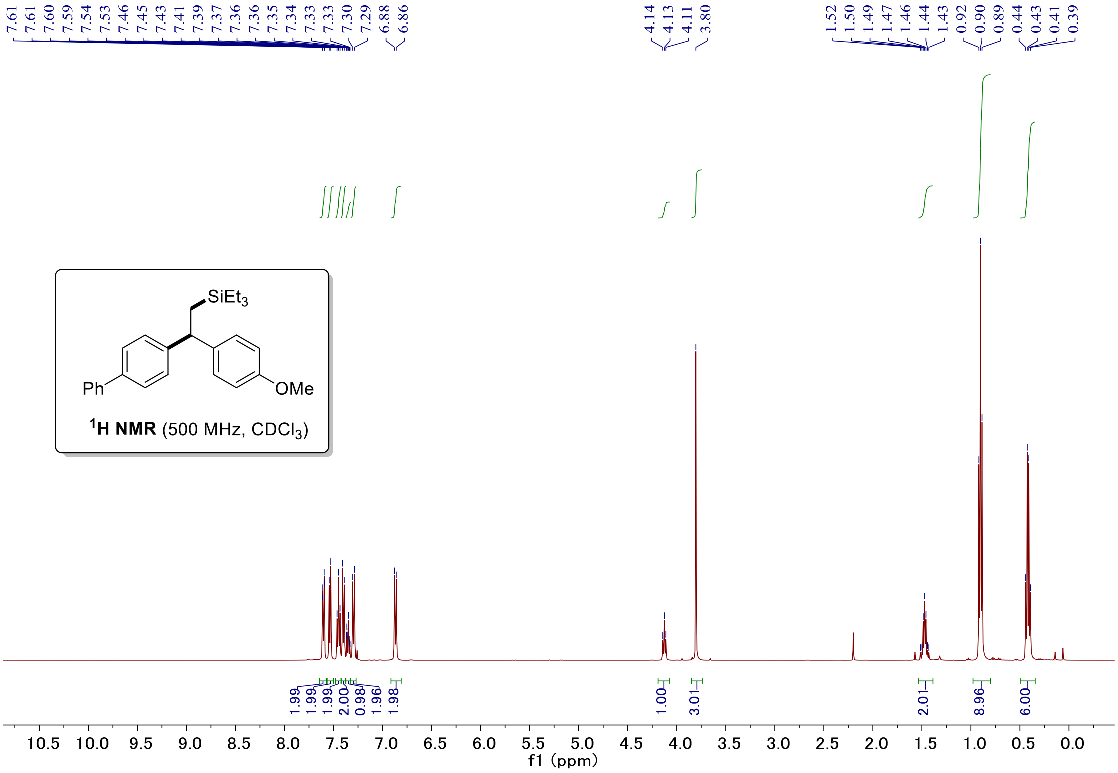

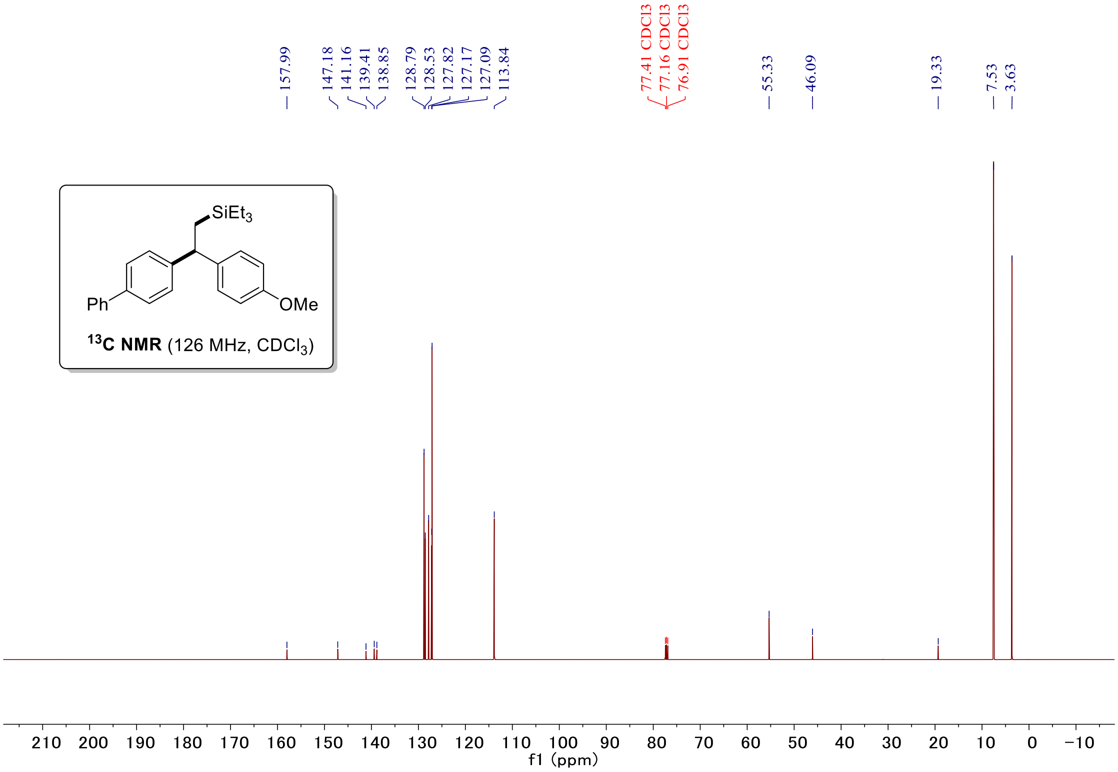

(2-(Biphenyl-4-yl)-2-(2-methoxyphenyl)ethyl)triethylsilane (4ag)

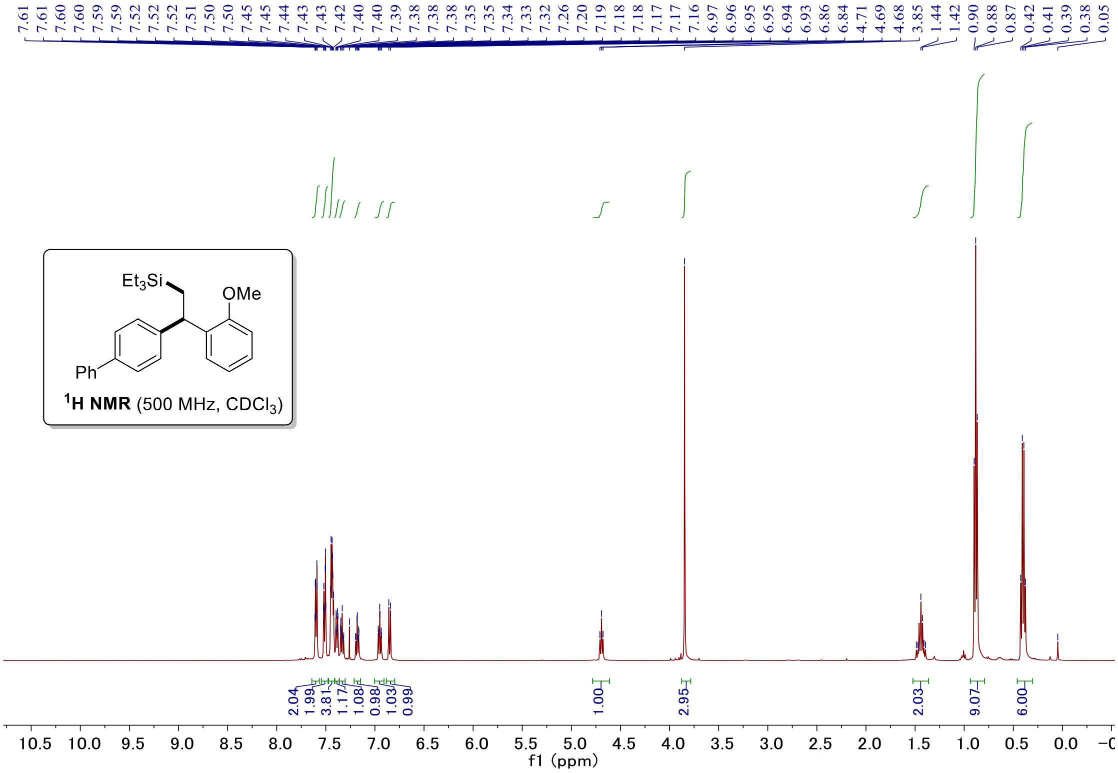

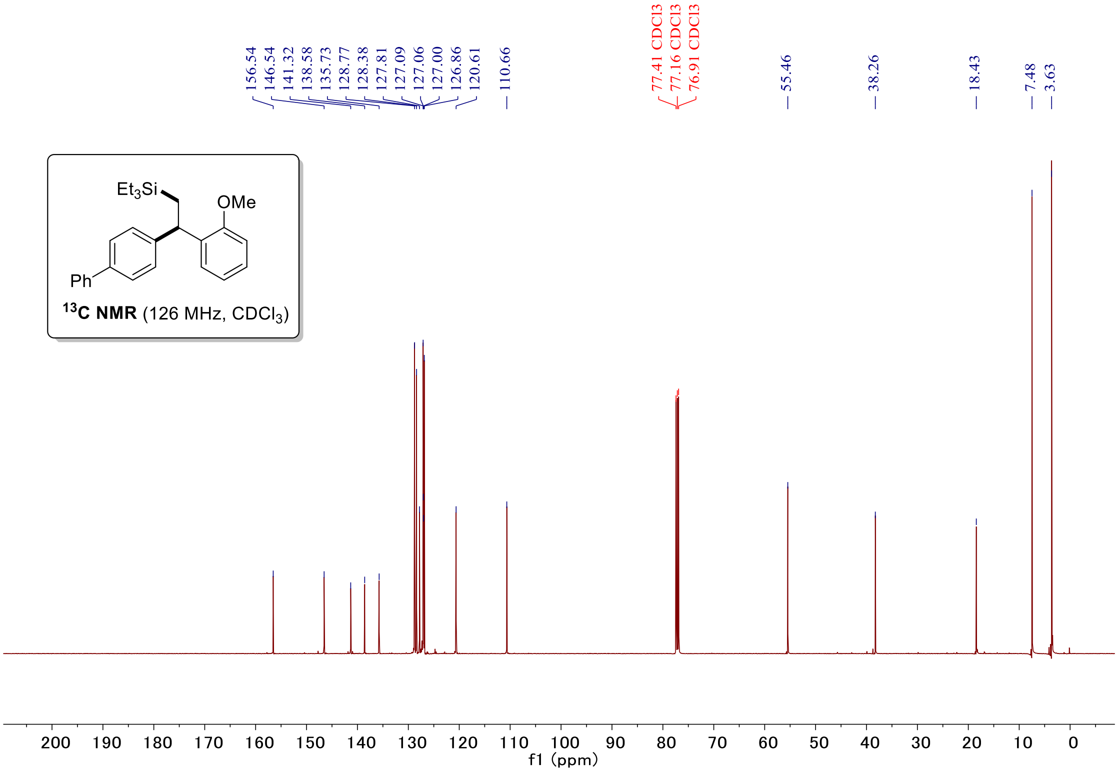

(2-(Biphenyl-4-yl)-2-(benzo[d][1,3]dioxol-5-yl)ethyl)triethylsilane (4ah)

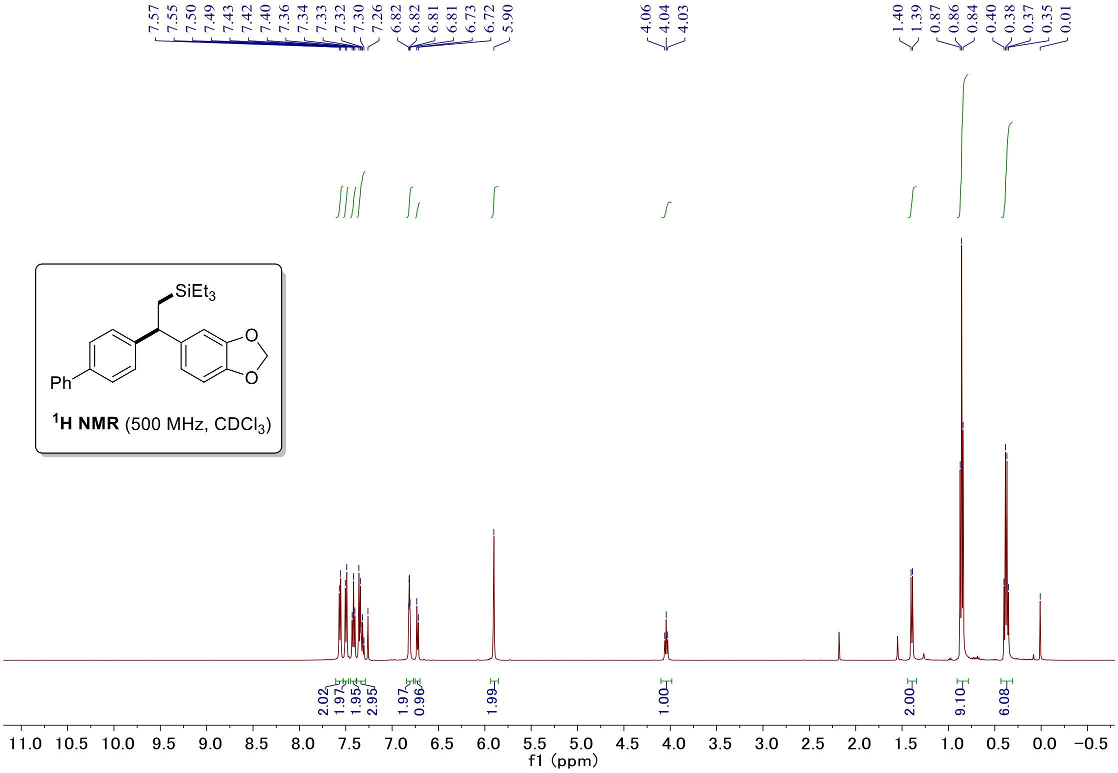

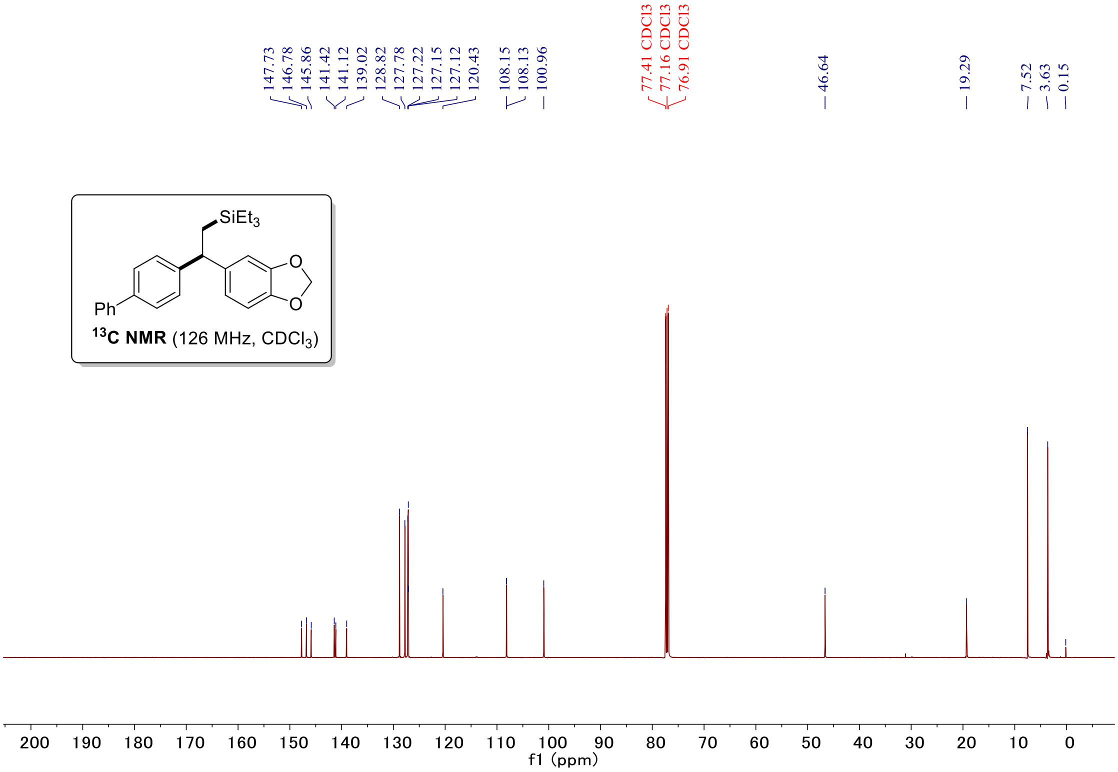

(2-(Biphenyl-4-yl)-2-(4-(methylthio)phenyl)ethyl)triethylsilane (4ai)

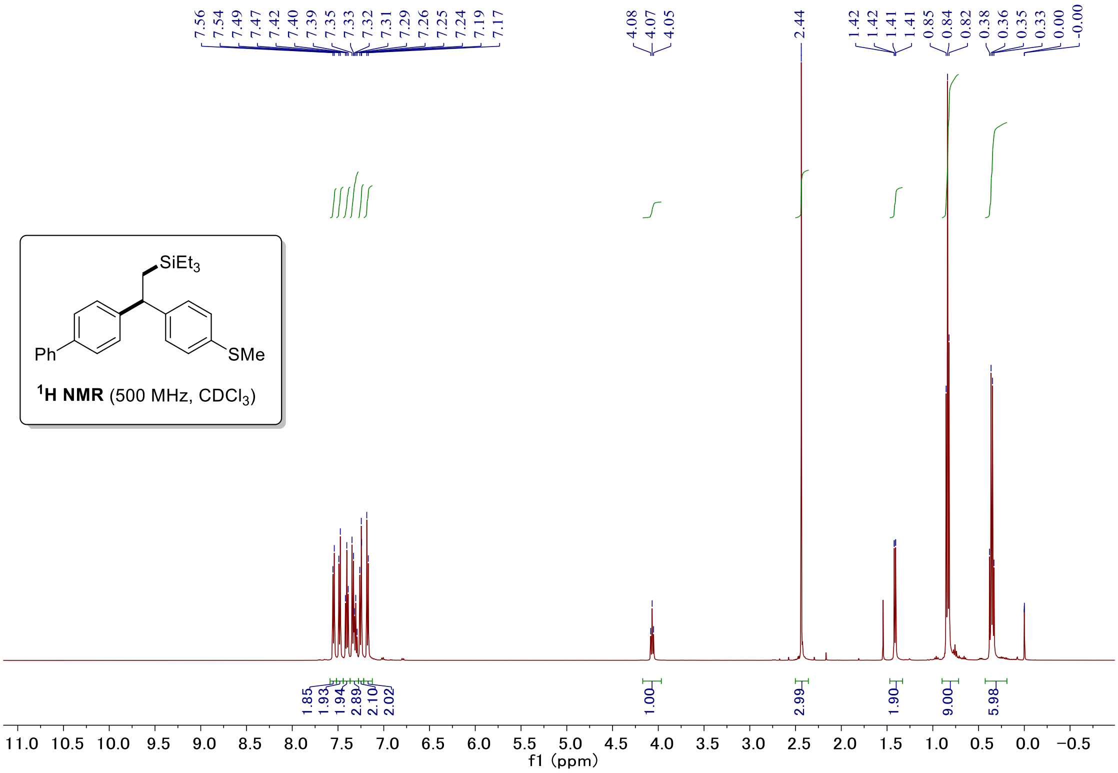

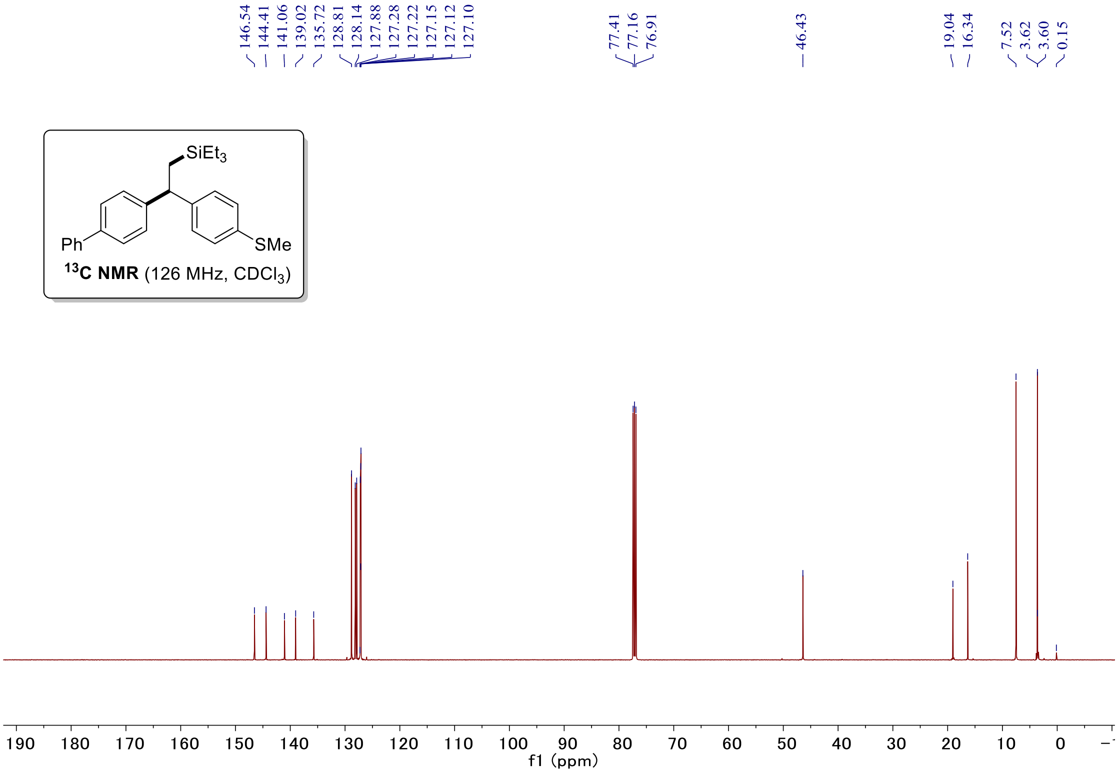

(2-(Biphenyl-2-yl)-2-(2-methoxyphenyl)ethyl)triethylsilane (4cg)

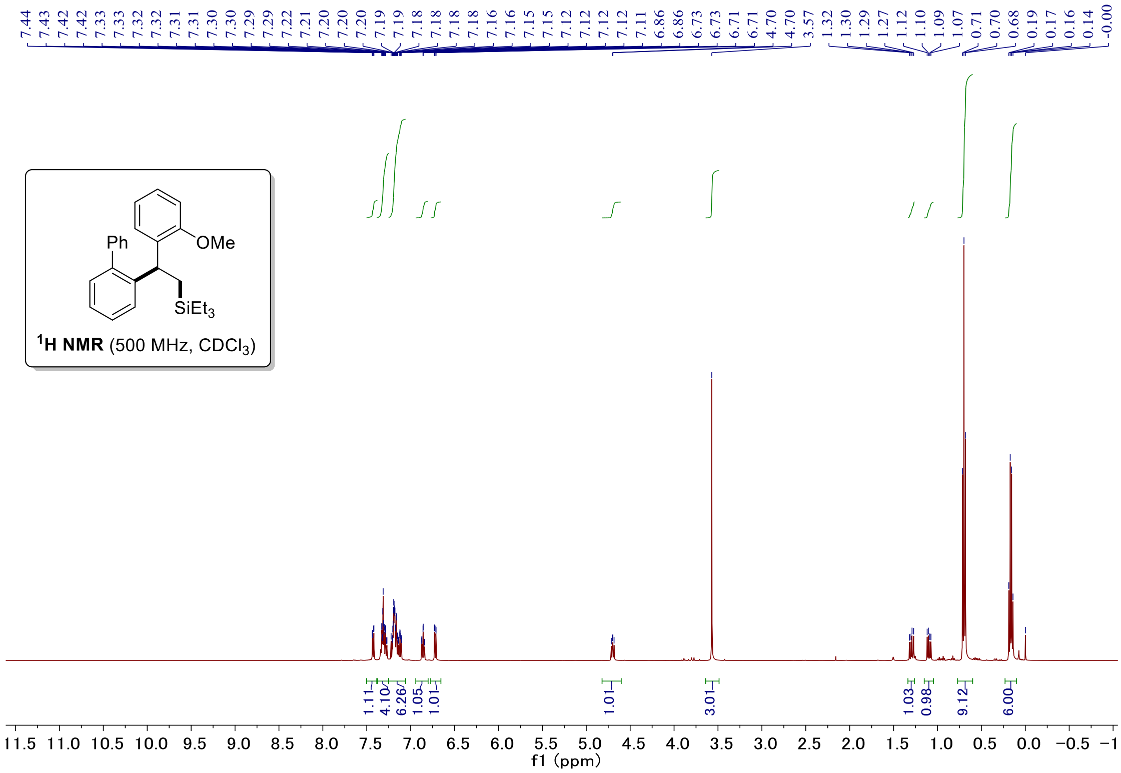

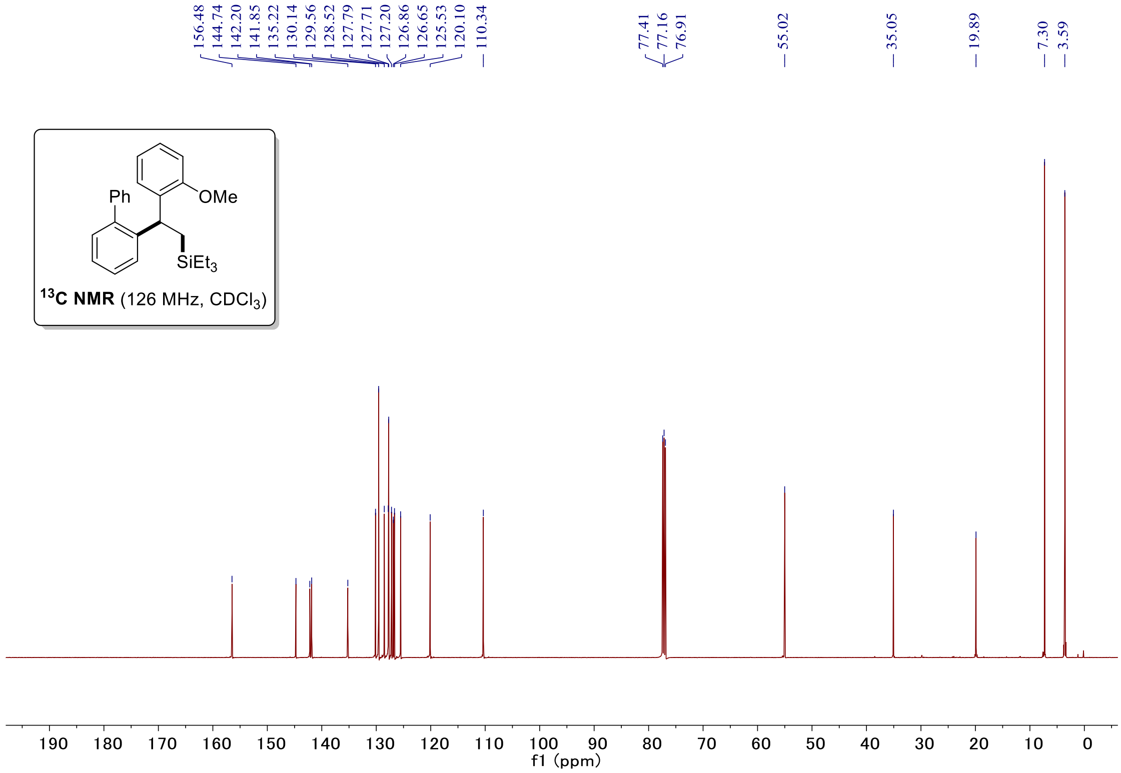

(2-(Biphenyl-2-yl)-2-(4-(methylthio)phenyl)ethyl)triethylsilane (4ci)

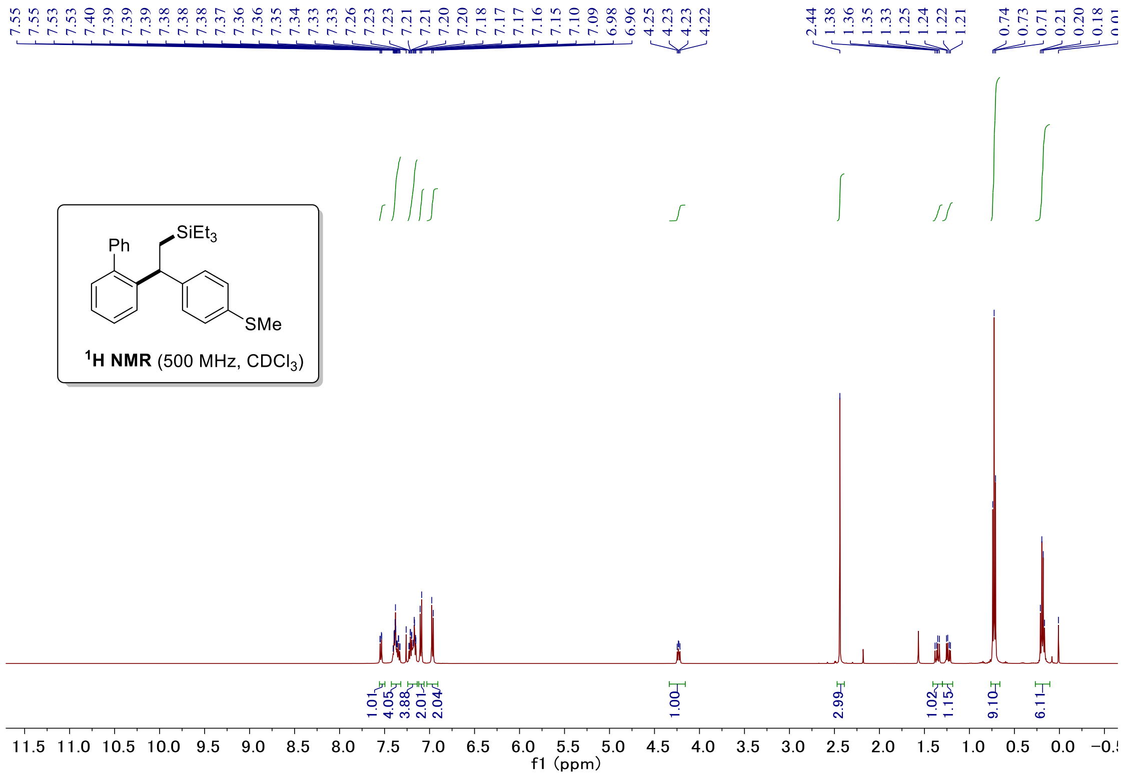

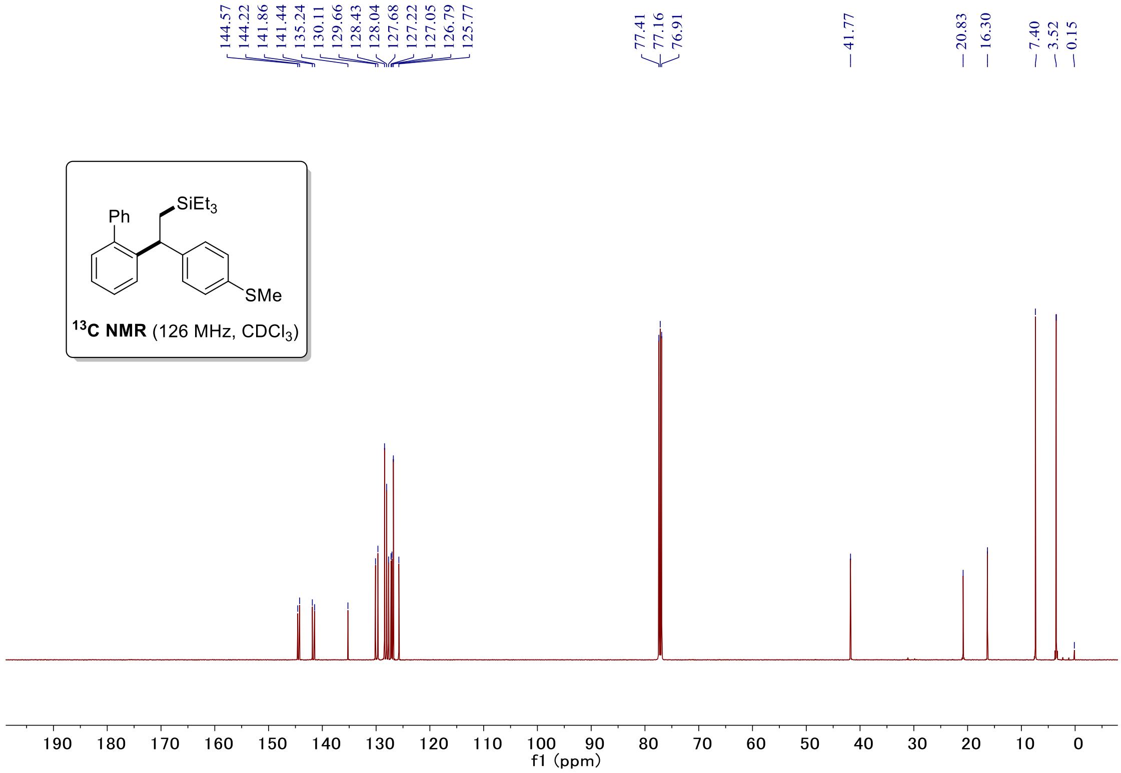

Triethyl(2-(*p*-tolyl)-2-(4'-(trifluoromethyl)-biphenyl-4-yl)ethyl)silane (4lc)

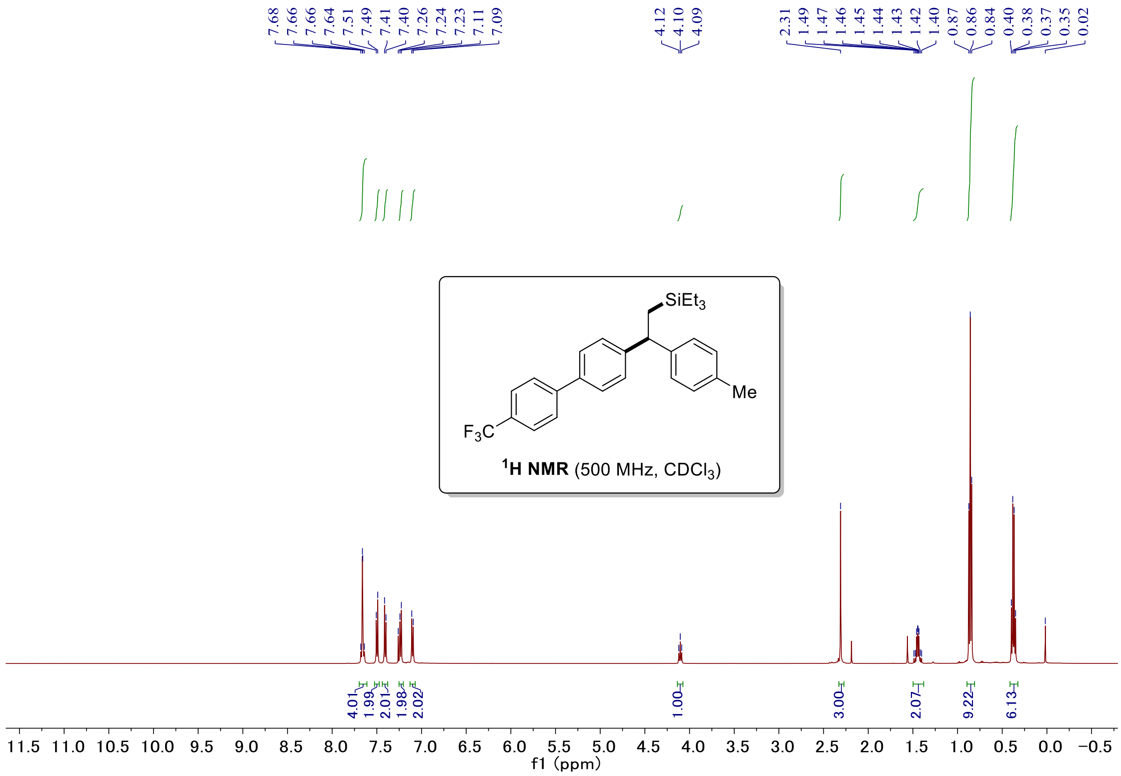

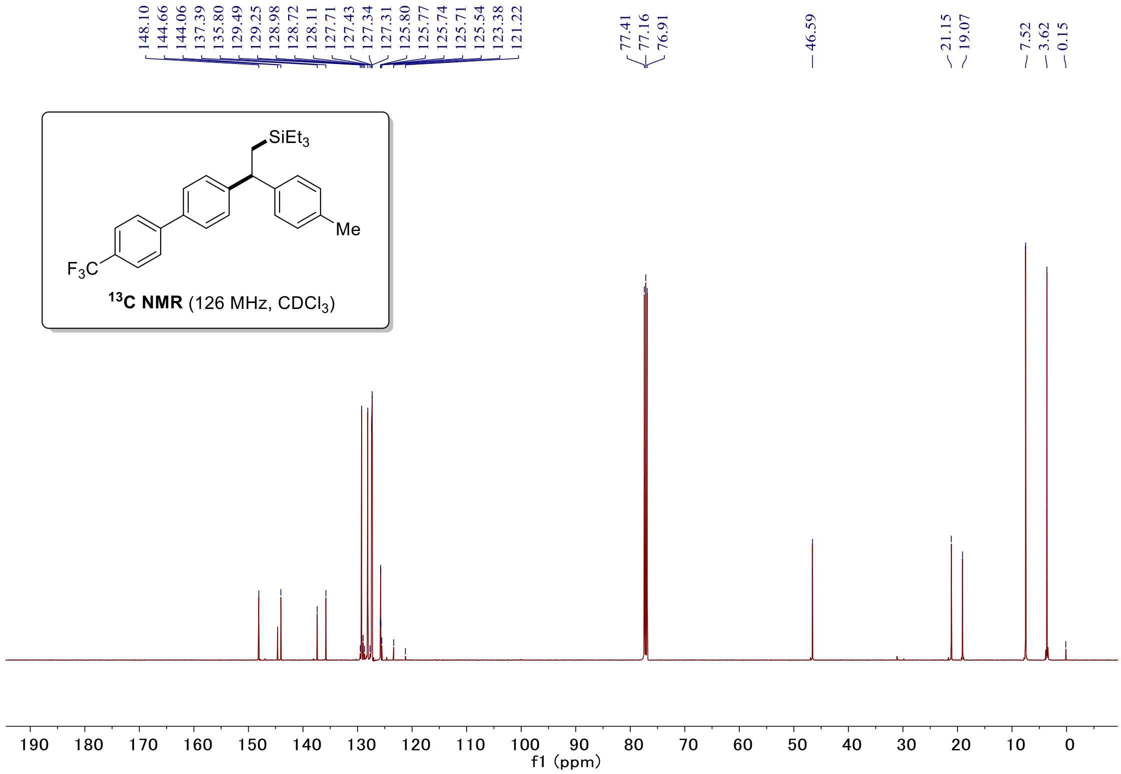

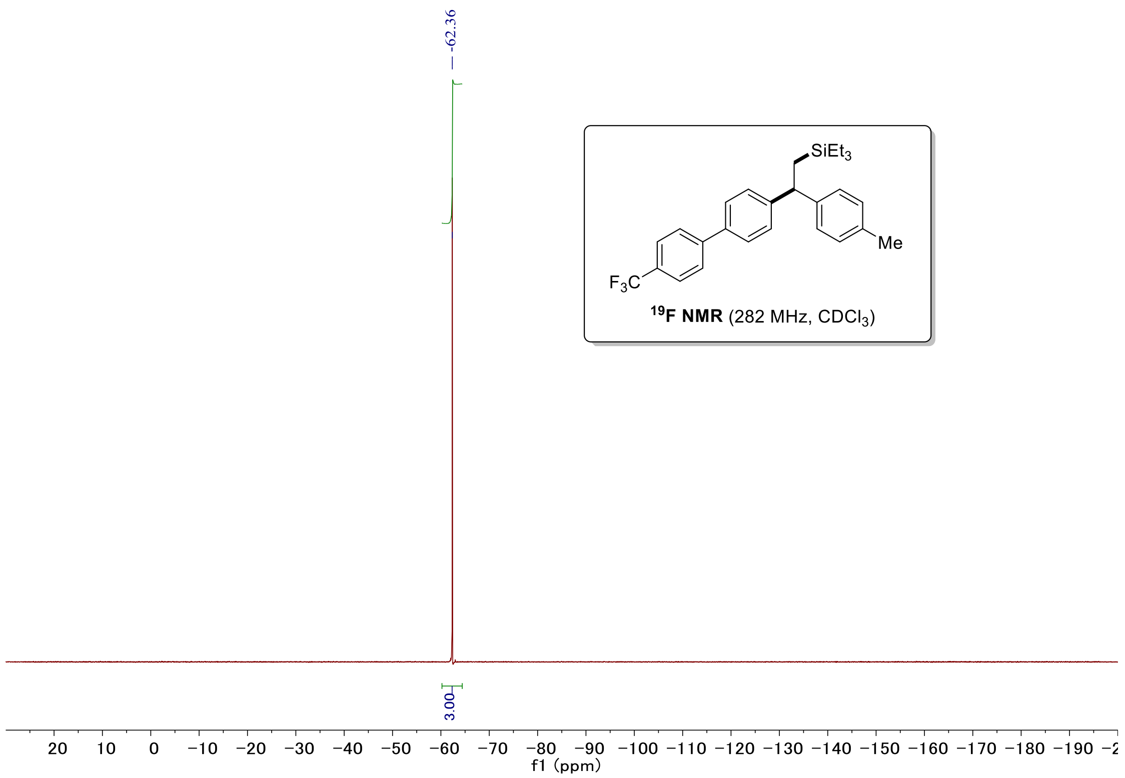
-62.36
SiEt3
Me
F3C
19F NMR (282 MHz, CDCl3)
3.00
20 10 0 −10 −20 −30 −40 −50 −60 −70 −80 −90 −100 −110 −120 −130 −140 −150 −160 −170 −180 −190
f1 (ppm)

Triethyl(2-(*p*-tolyl)-2-(4'-(chloro)-biphenyl-4-yl)ethyl)silane (4mc)

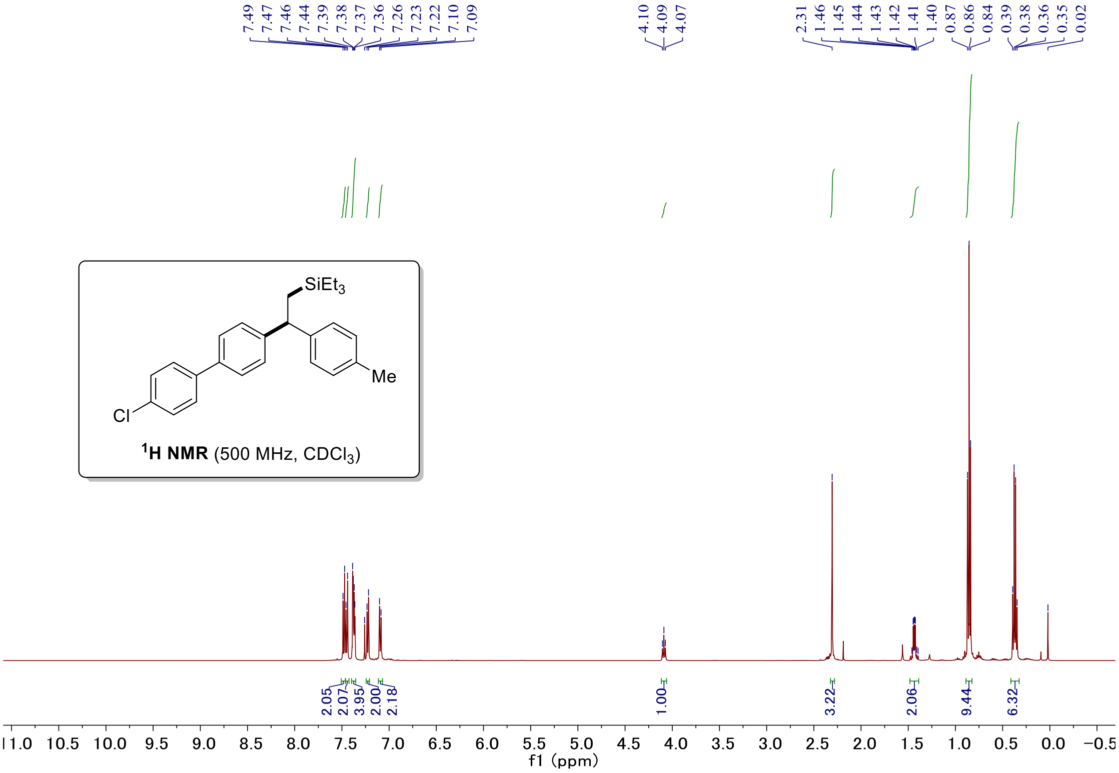

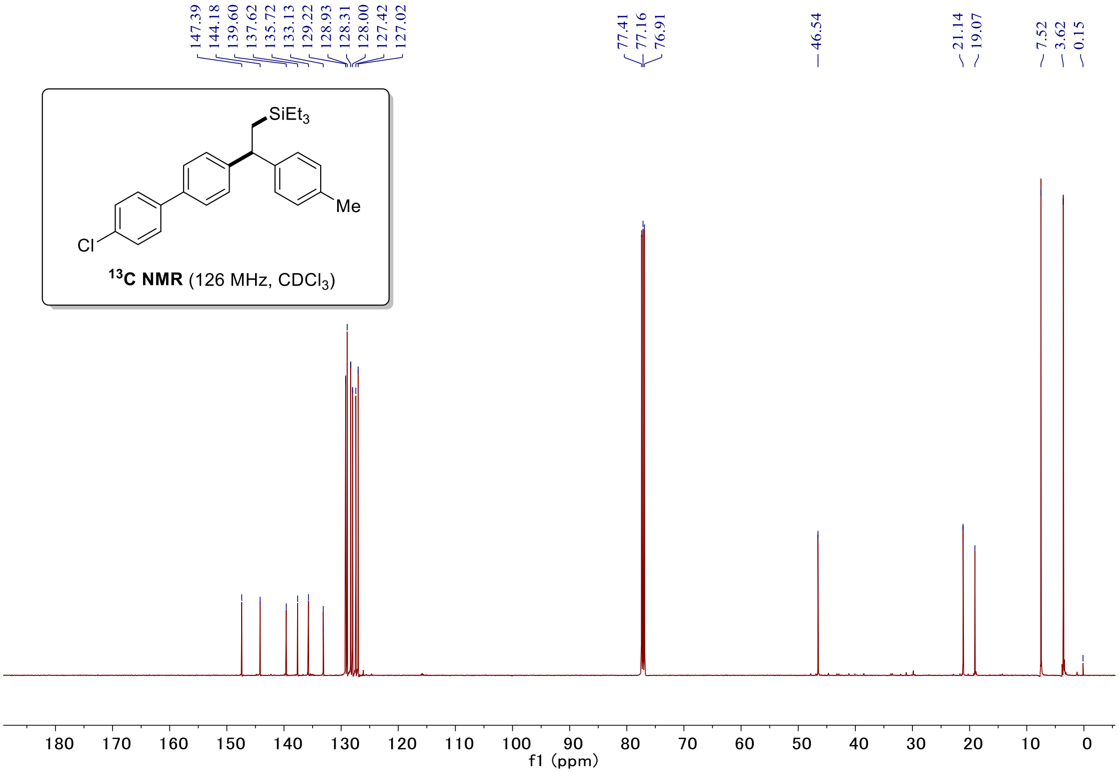

Triethyl(2-(3-(3-methoxypropyl)phenyl)-2-(4-(methylthio)phenyl)ethyl)silane (4vi)

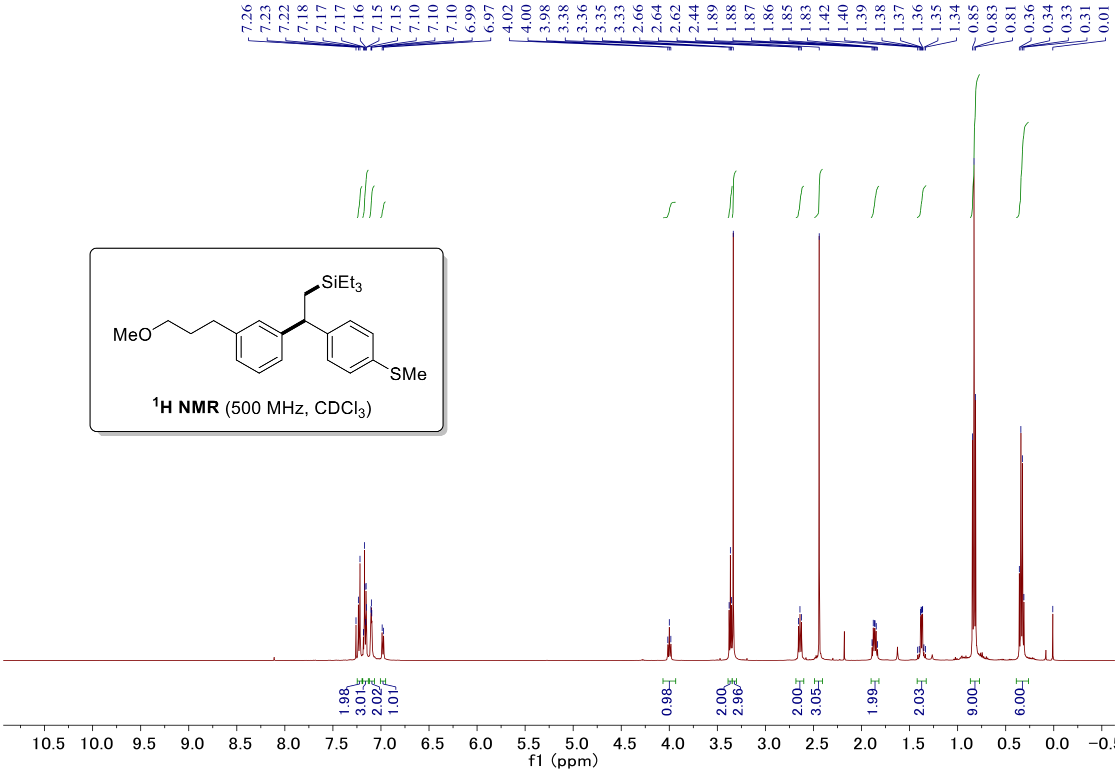

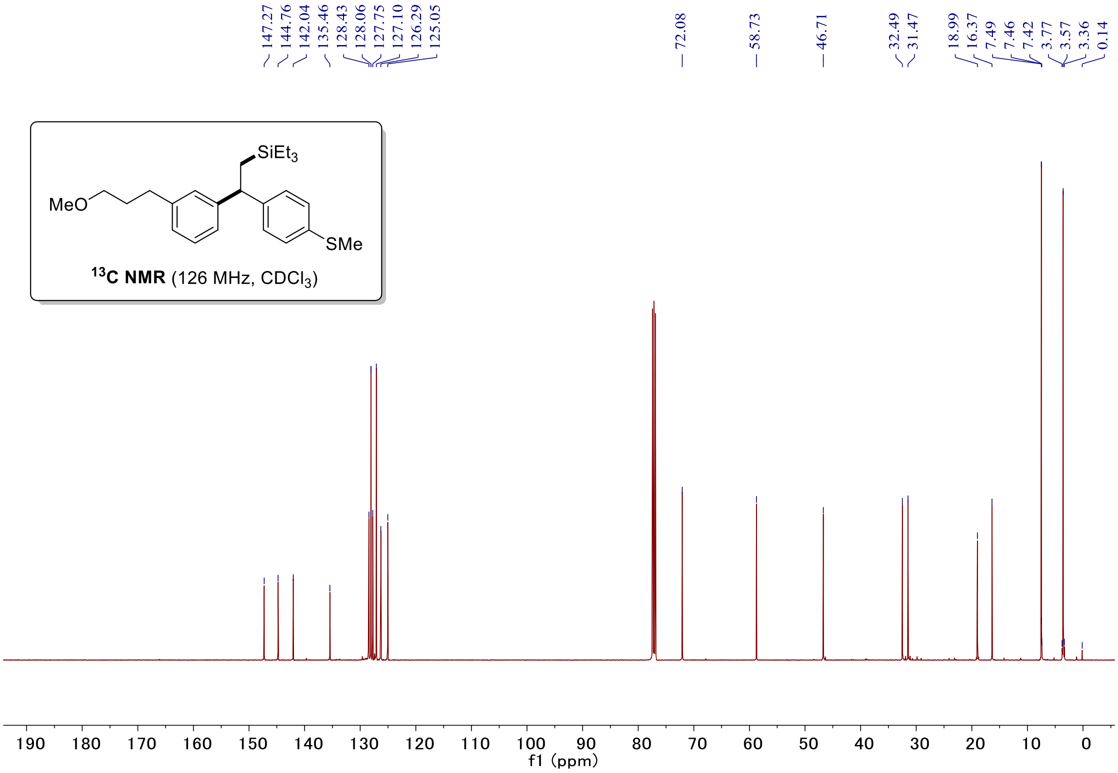

(2-(Biphenyl-4-yl)-2-(naphthalen-2-yl)ethyl)dimethyl(phenyl)silane (4ab`)

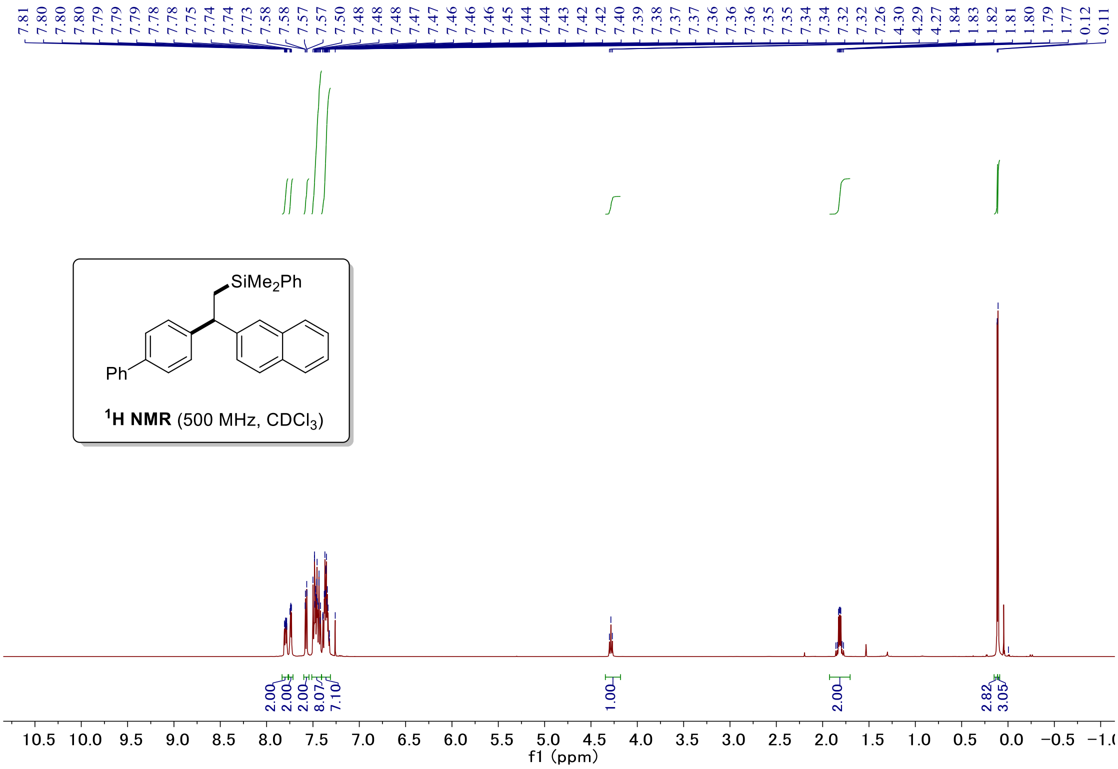

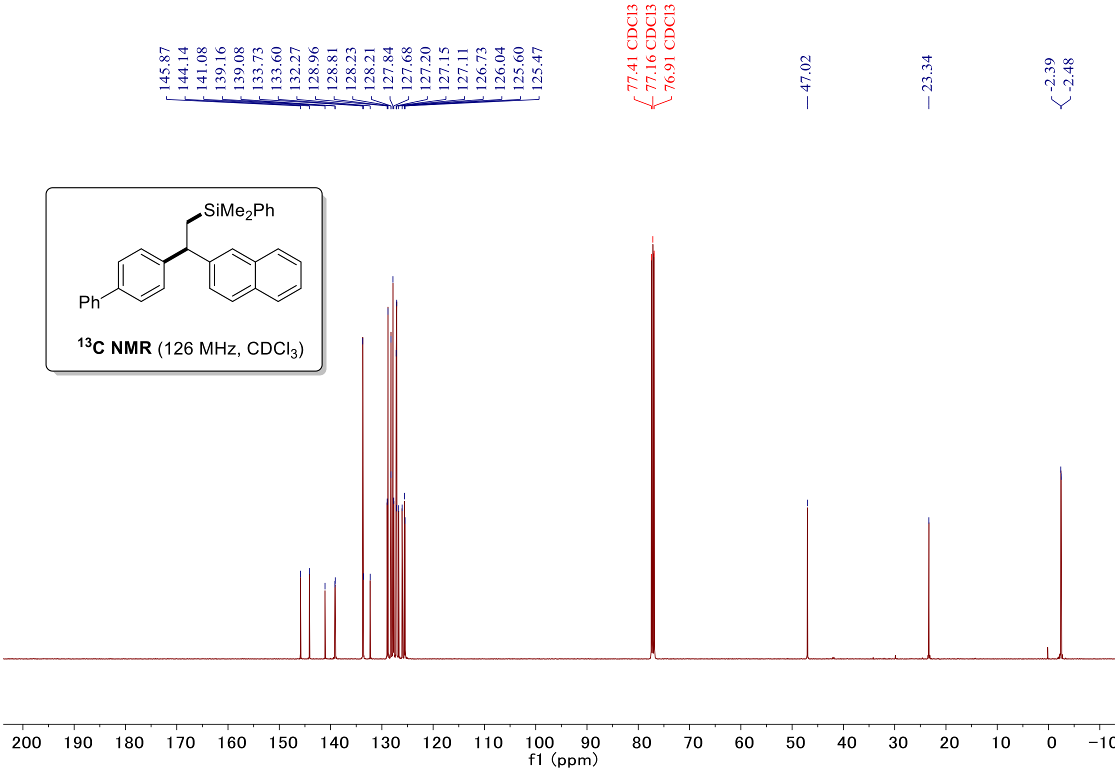

(2-(Biphenyl-4-yl)-2,2-diphenylethyl)triethylsilane (4aj)

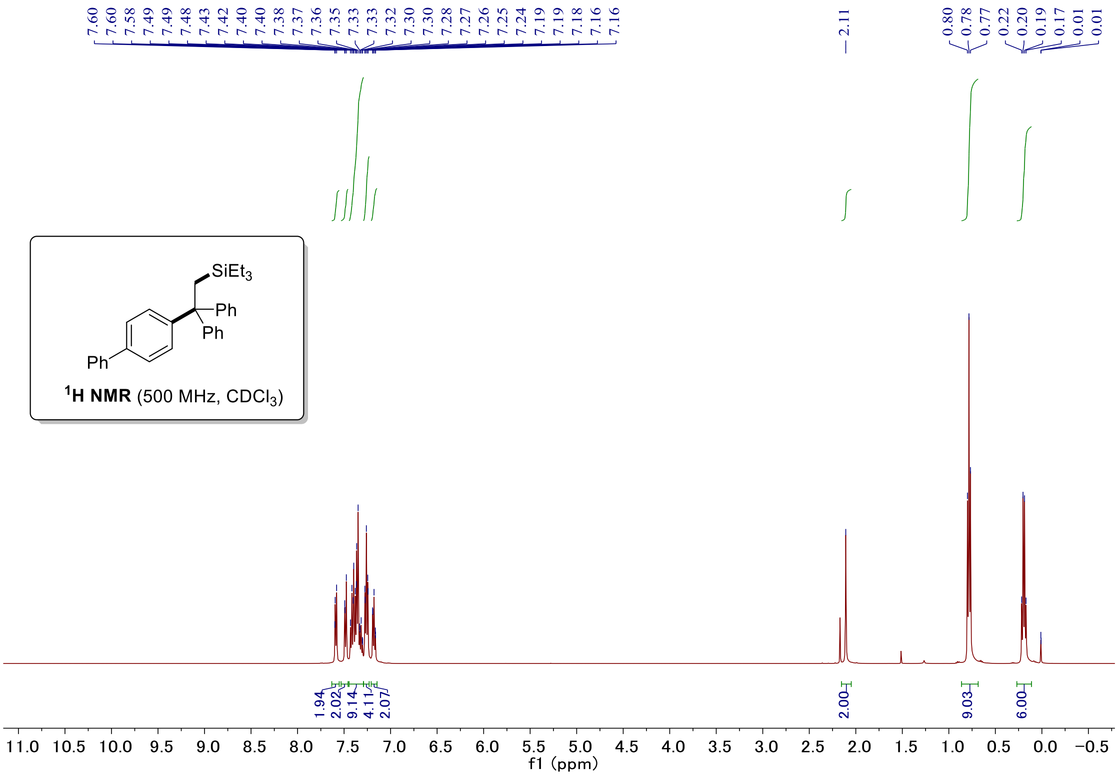

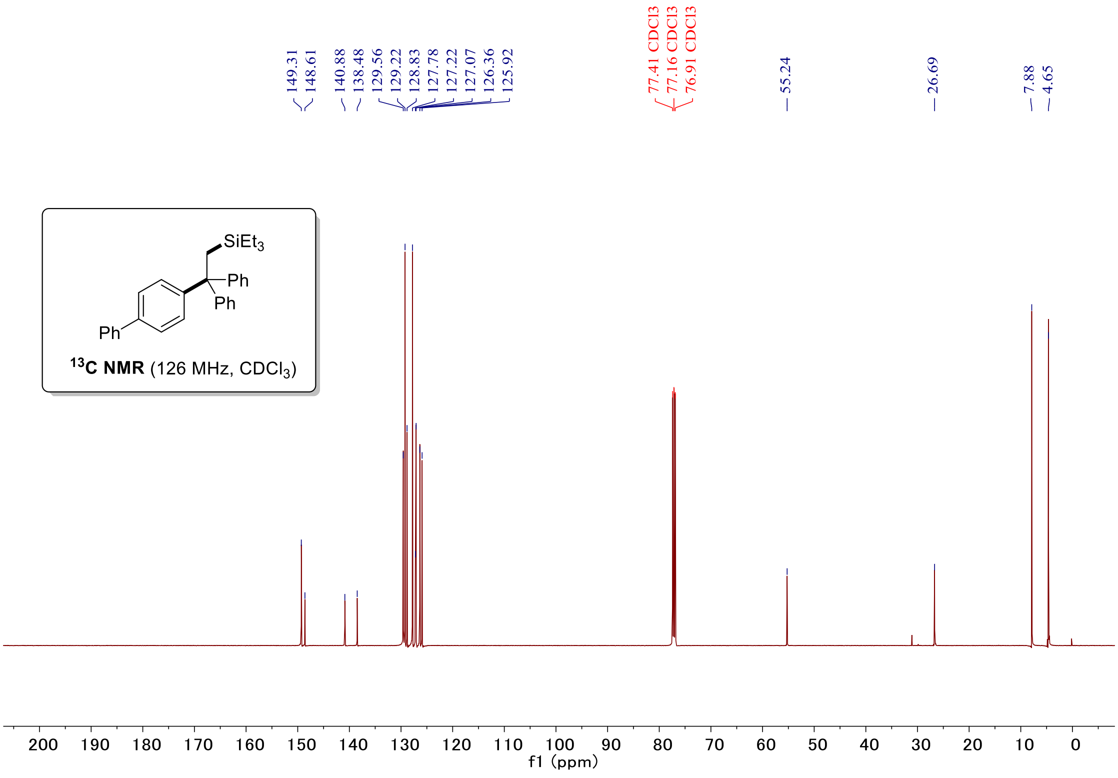

(2-(Biphenyl-4-yl)-2-phenylpropyl)triethylsilane (4ak)

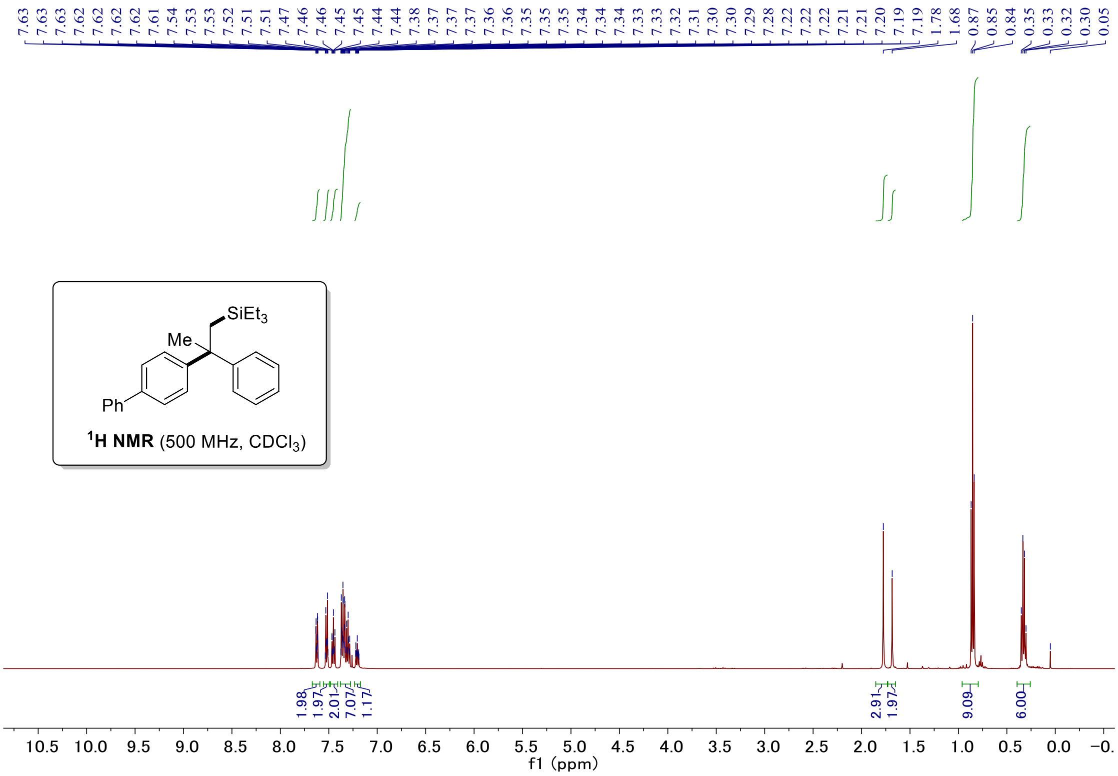

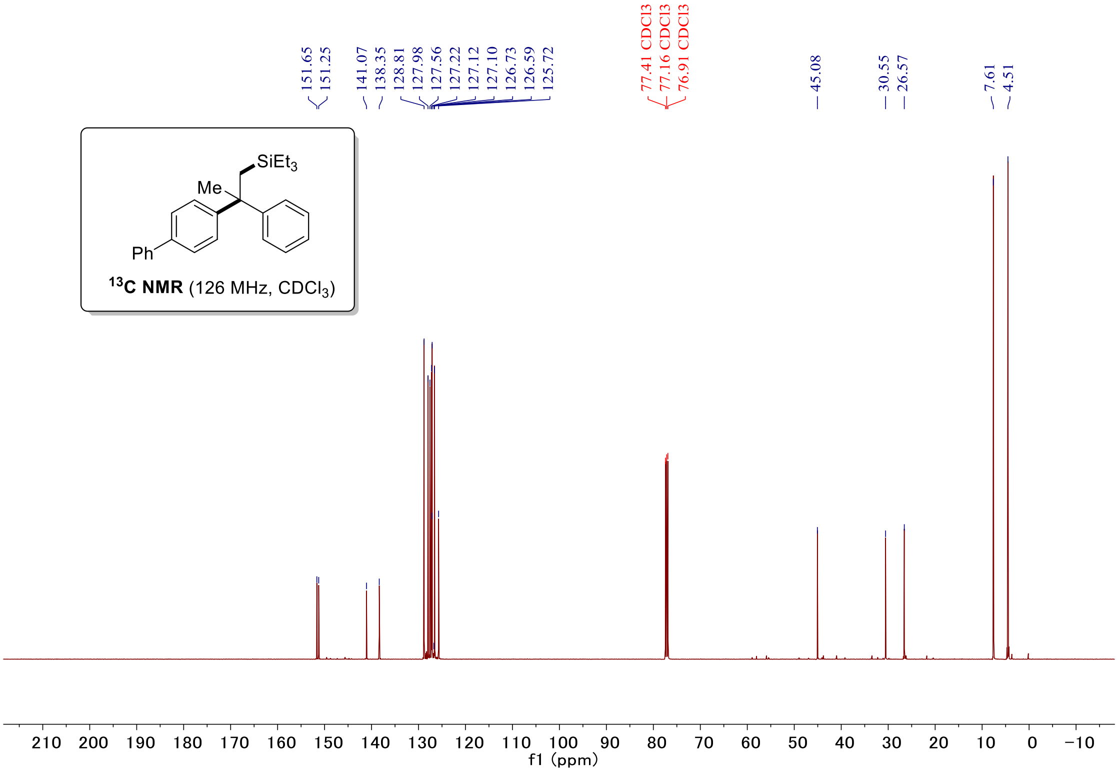

(2-(Biphenyl-4-yl)-4-methoxy-2-phenylbutyl)triethylsilane (4al)

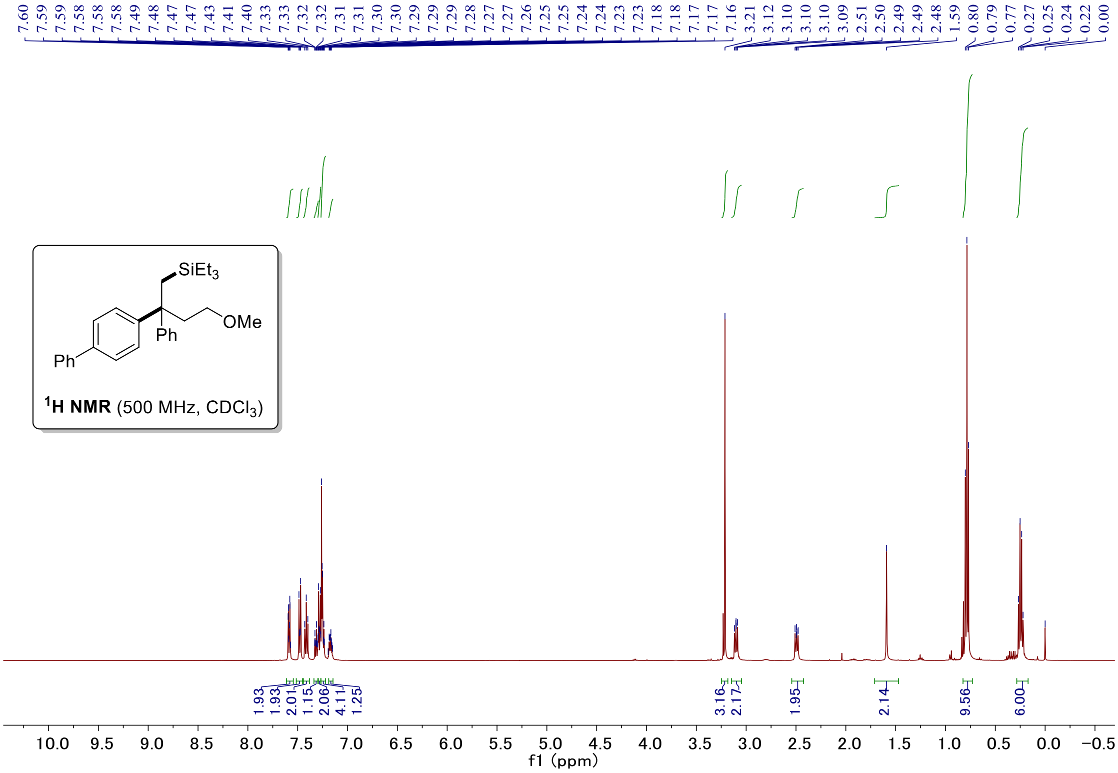

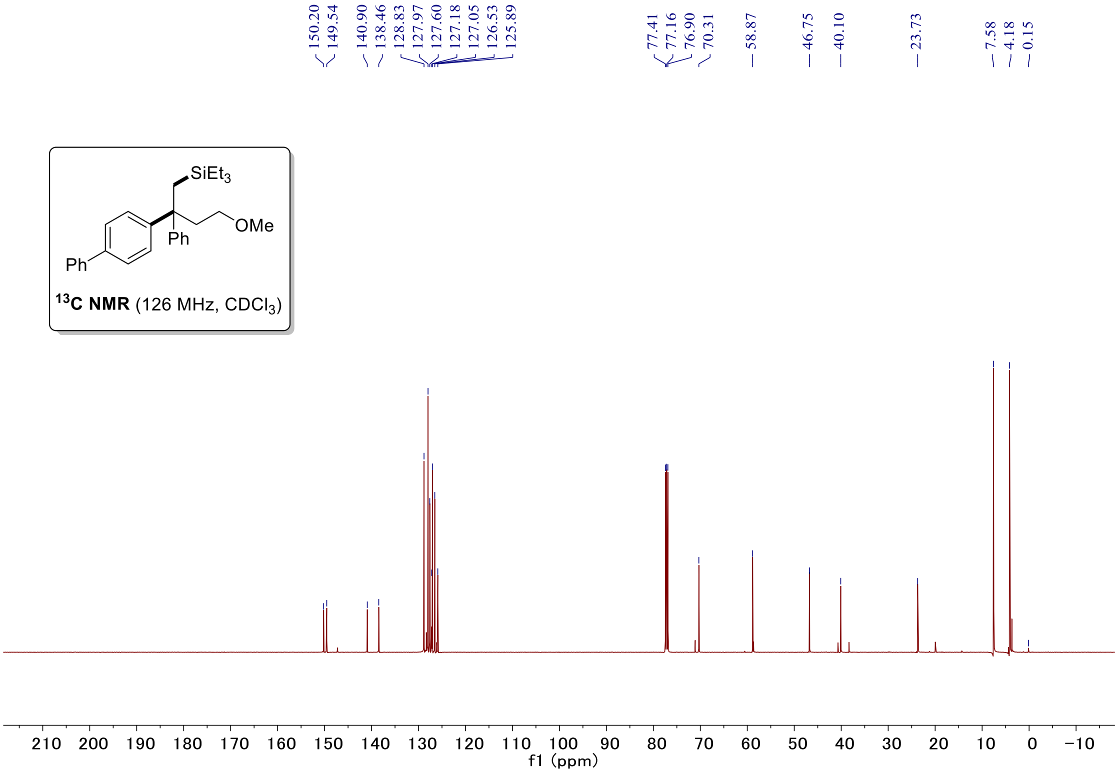

(2,2-Diphenylpropyl)triethylsilane (4fk)

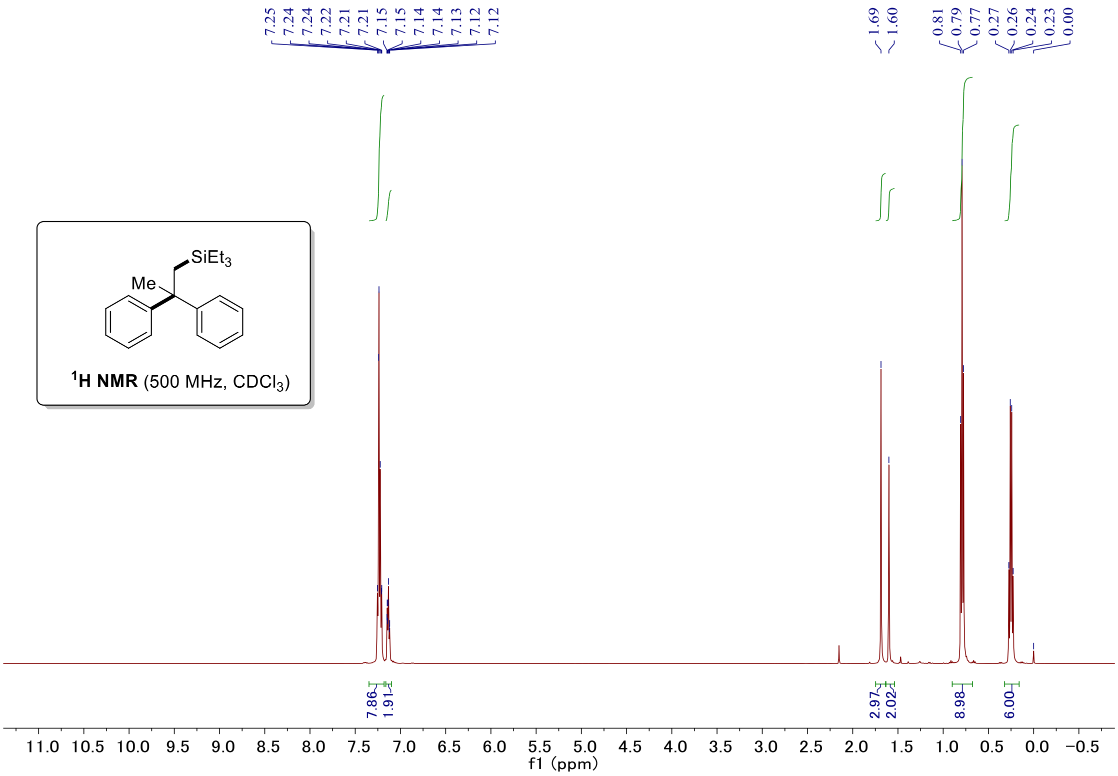

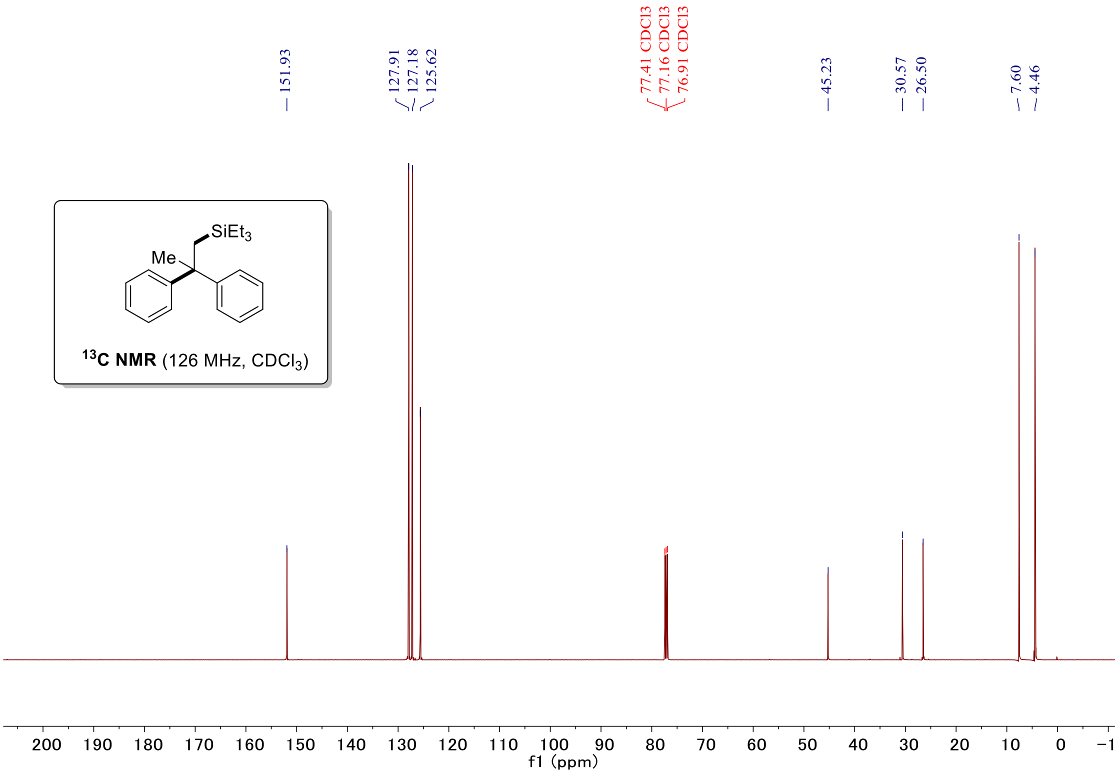

(2-(4'-Chloro-biphenyl-4-yl)-2-phenylpropyl)triethylsilane (4mk)

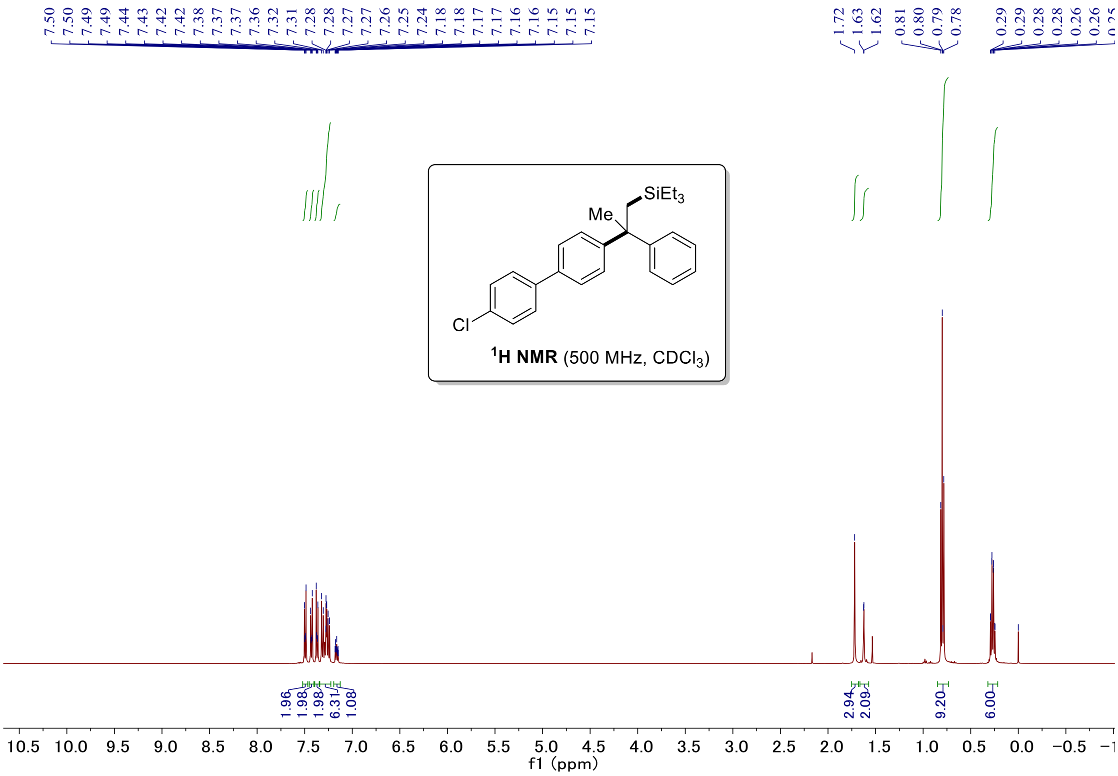

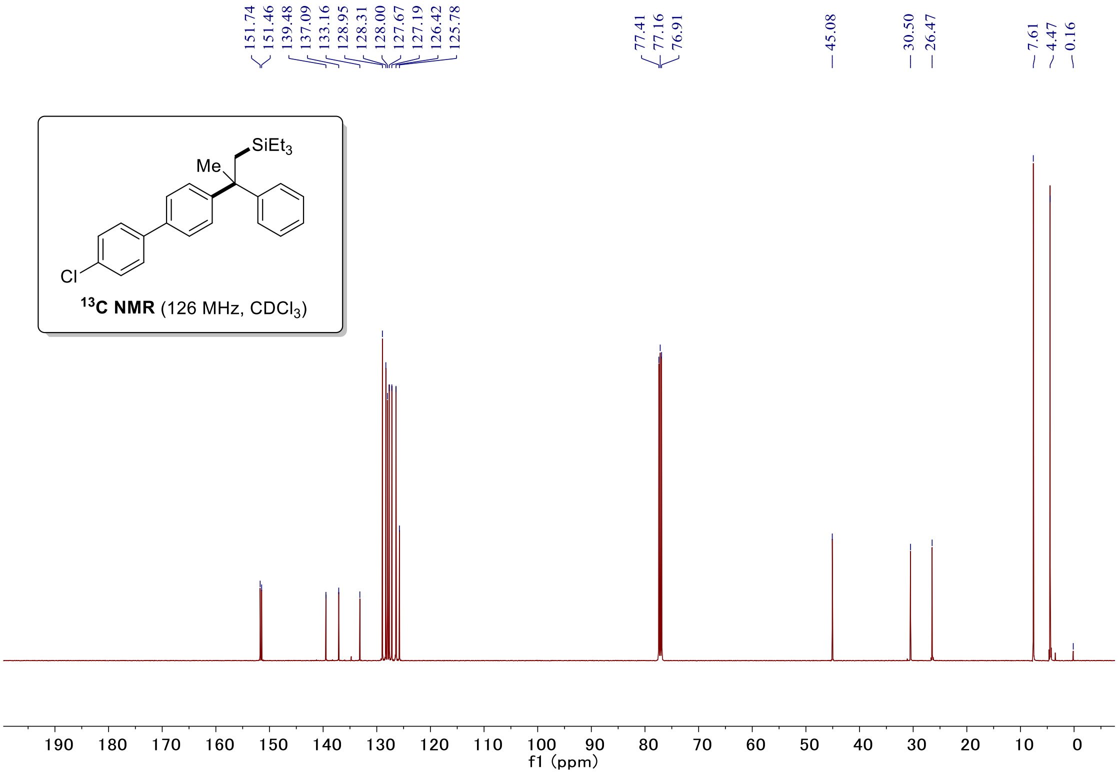

(2,6-Diphenylhexyl)triethylsilane (5aa)

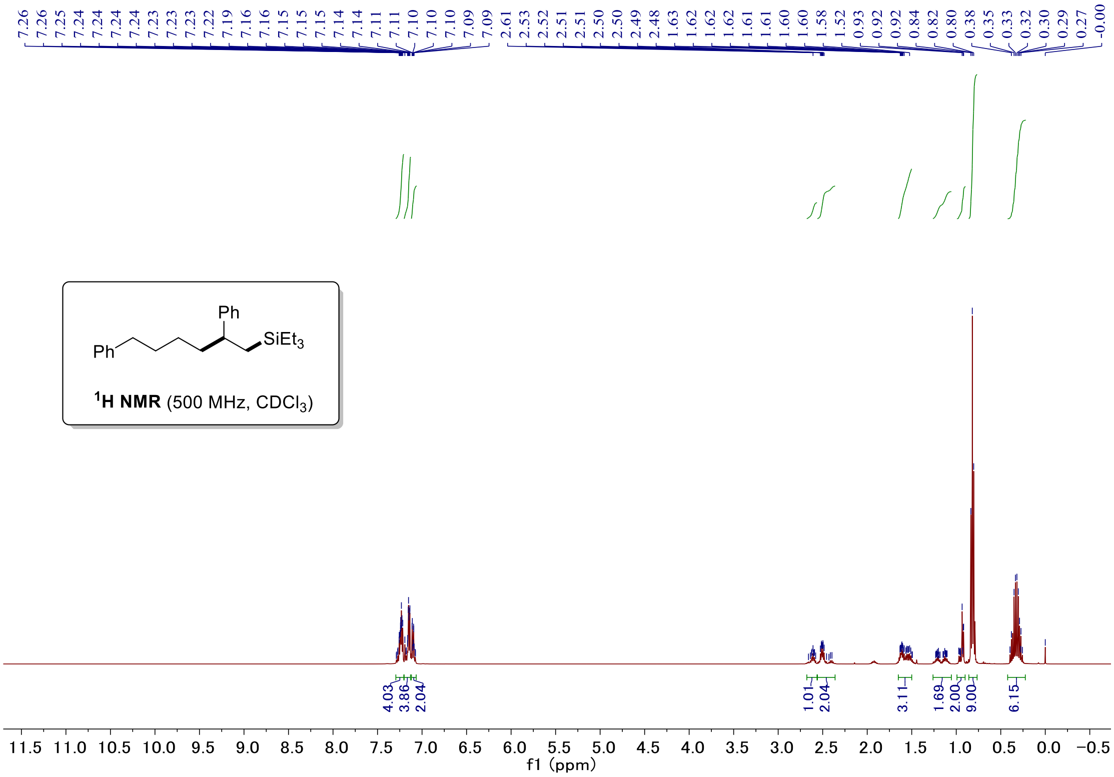

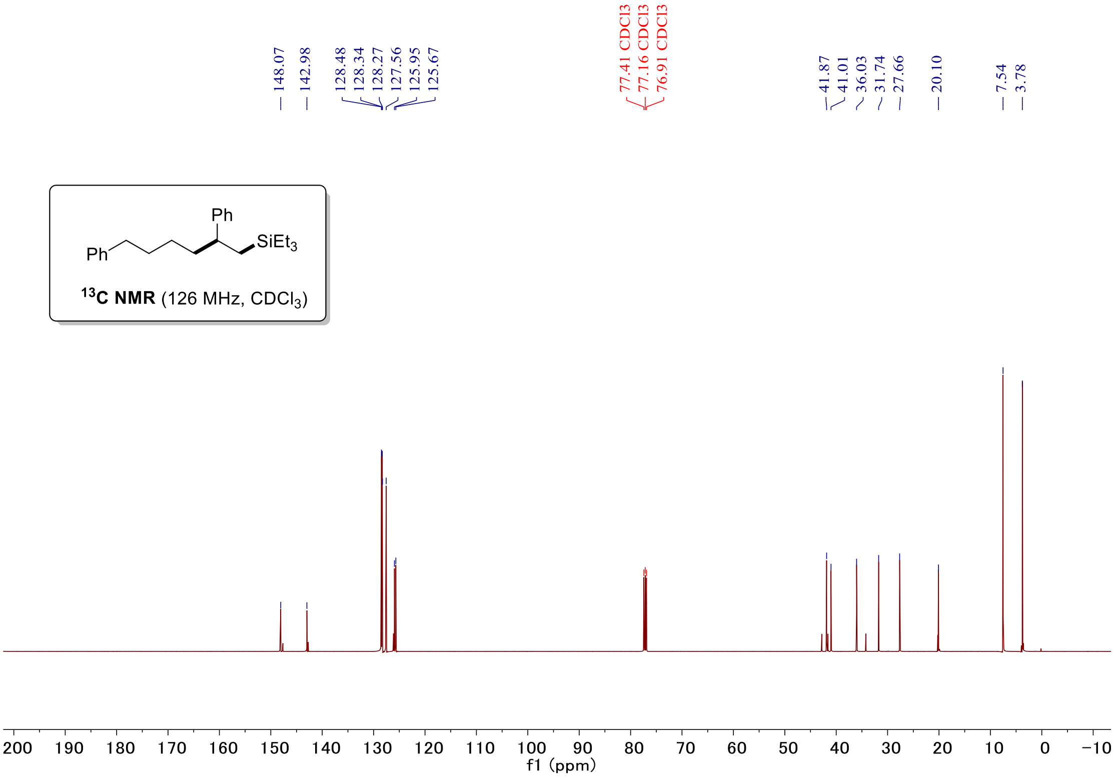

(3-(Biphenyl-4-yl)-2-phenylpropyl)triethylsilane (5ba)

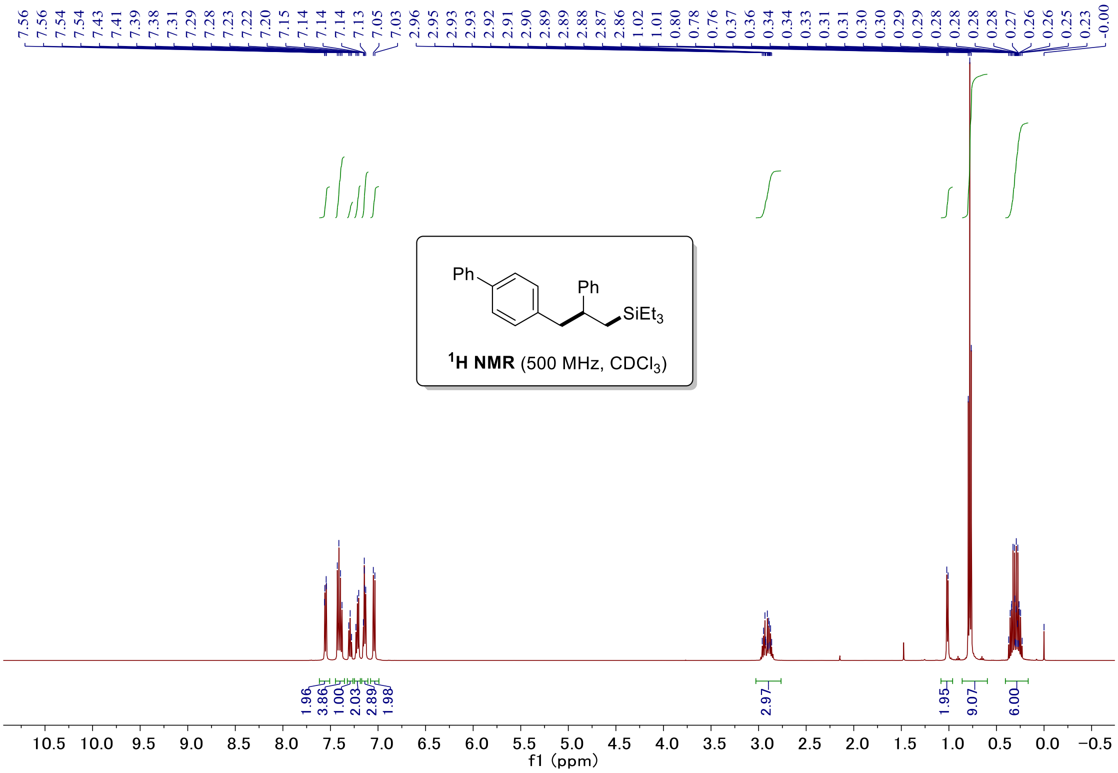

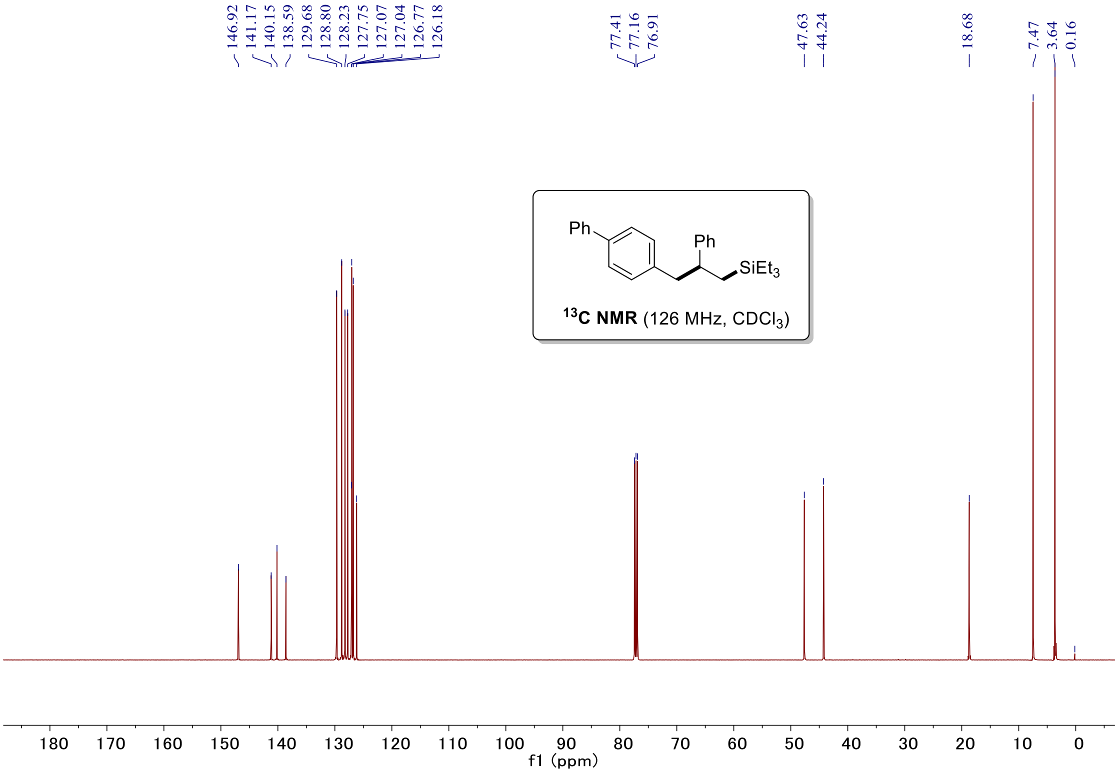

Triethyl(3-(naphthalen-1-yl)-2-phenylpropyl)silane (5ca)

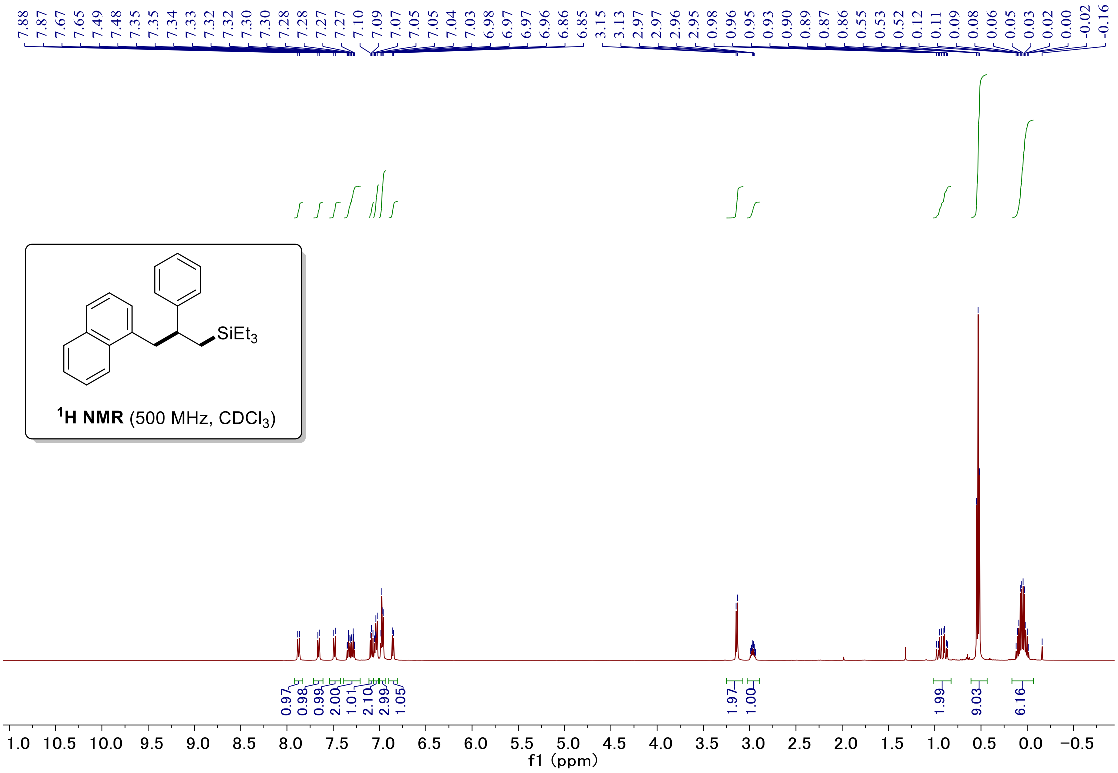

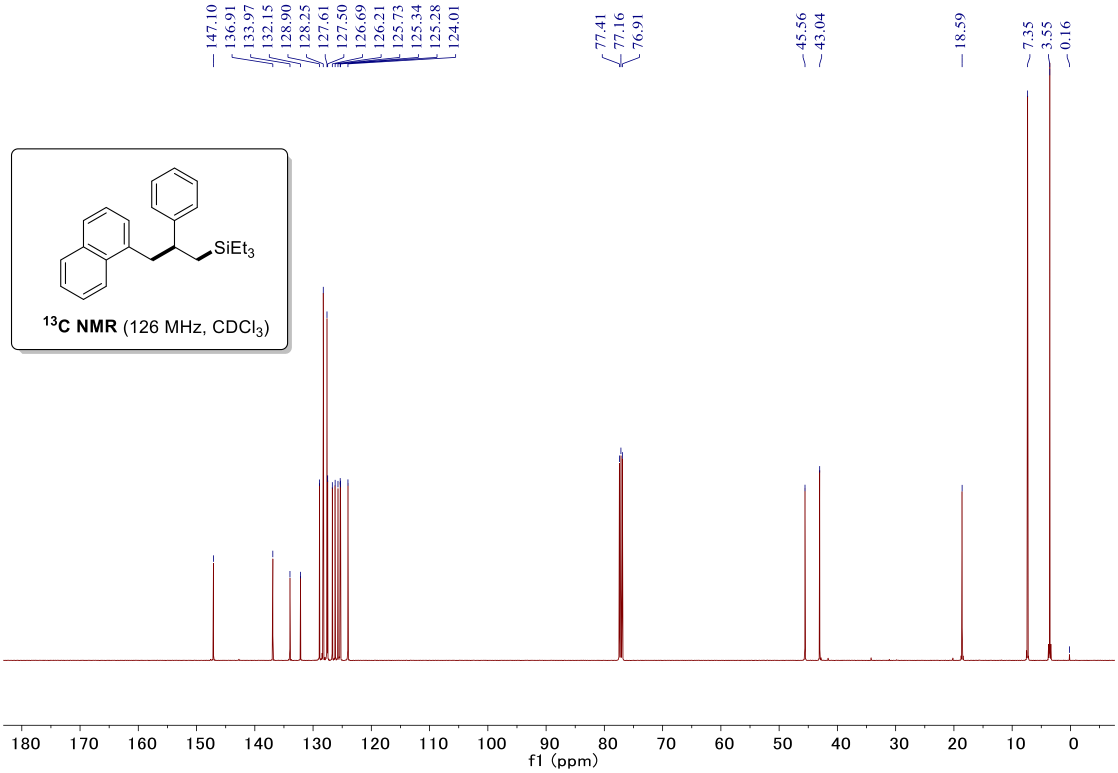

Triethyl(2-phenyl-3-(4-(trifluoromethoxy)phenyl)propyl)silane (5da)

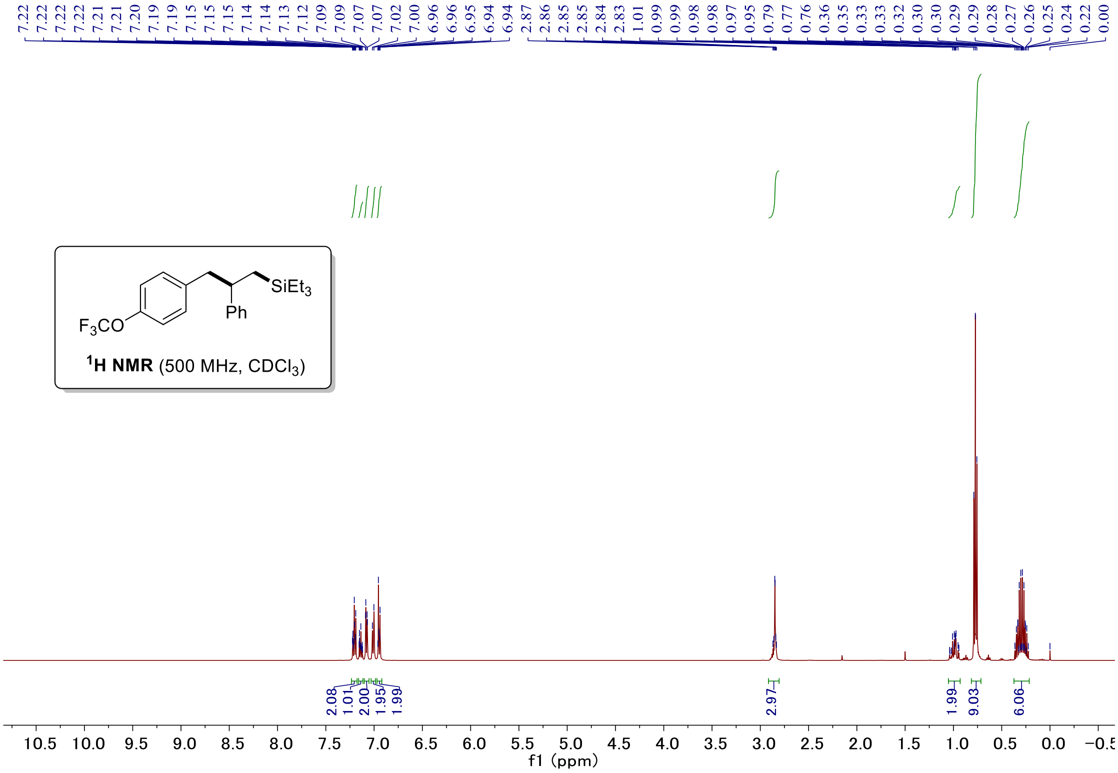

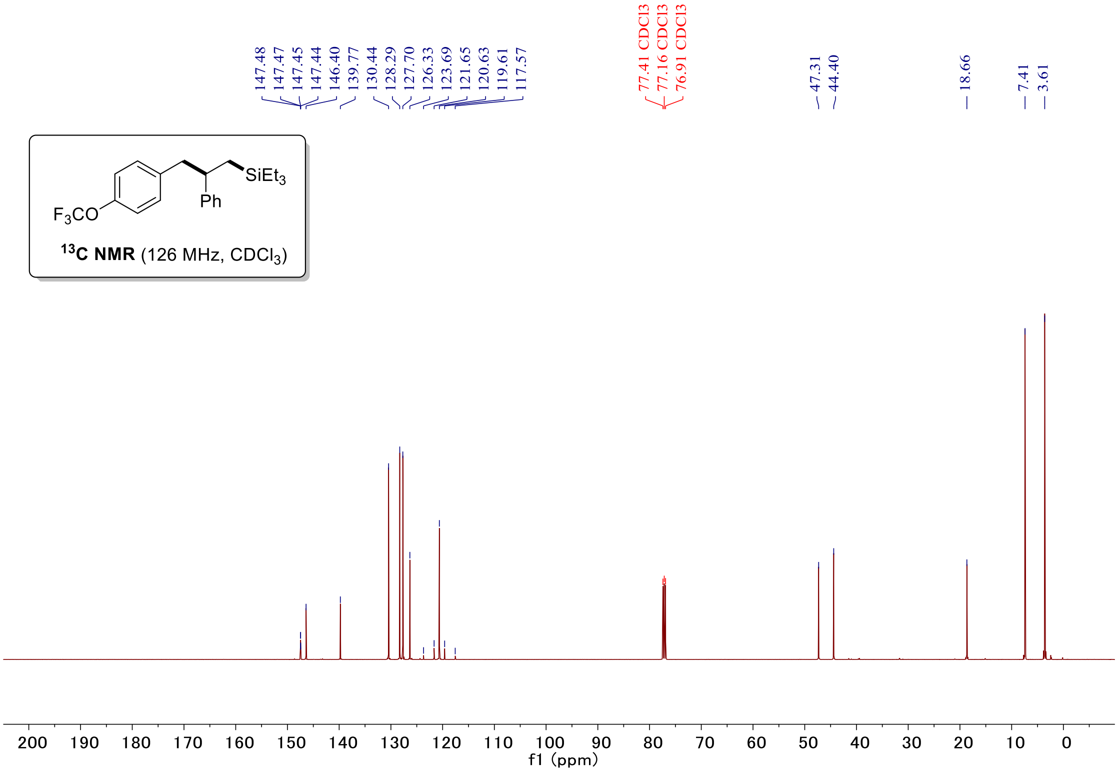

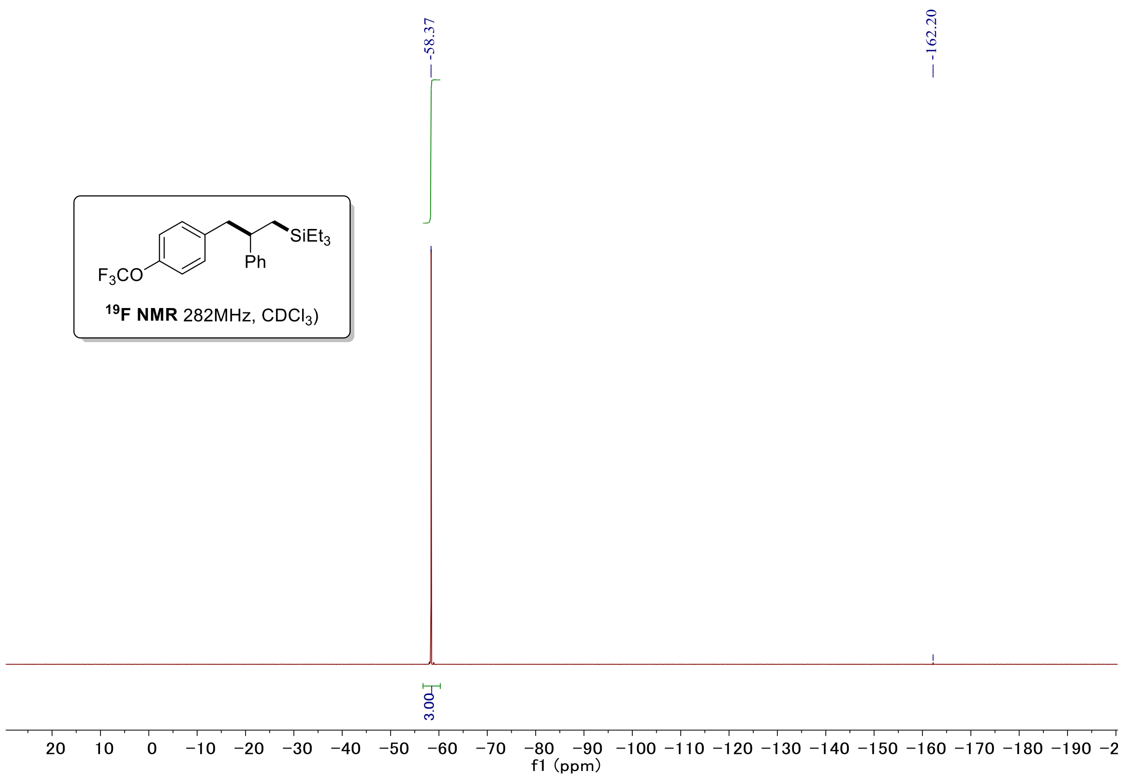
-58.37
-162.20
SiEt3
Ph
F3CO
19F NMR 282MHz, CDCl3)
3.00
20 10 0 −10 −20 −30 −40 −50 −60 −70 −80 −90 −100 −110 −120 −130 −140 −150 −160 −170 −180 −190 −2
f1 (ppm)

Triethyl(2-(4-(methylthio)phenyl)dodecyl)silane (5ei)

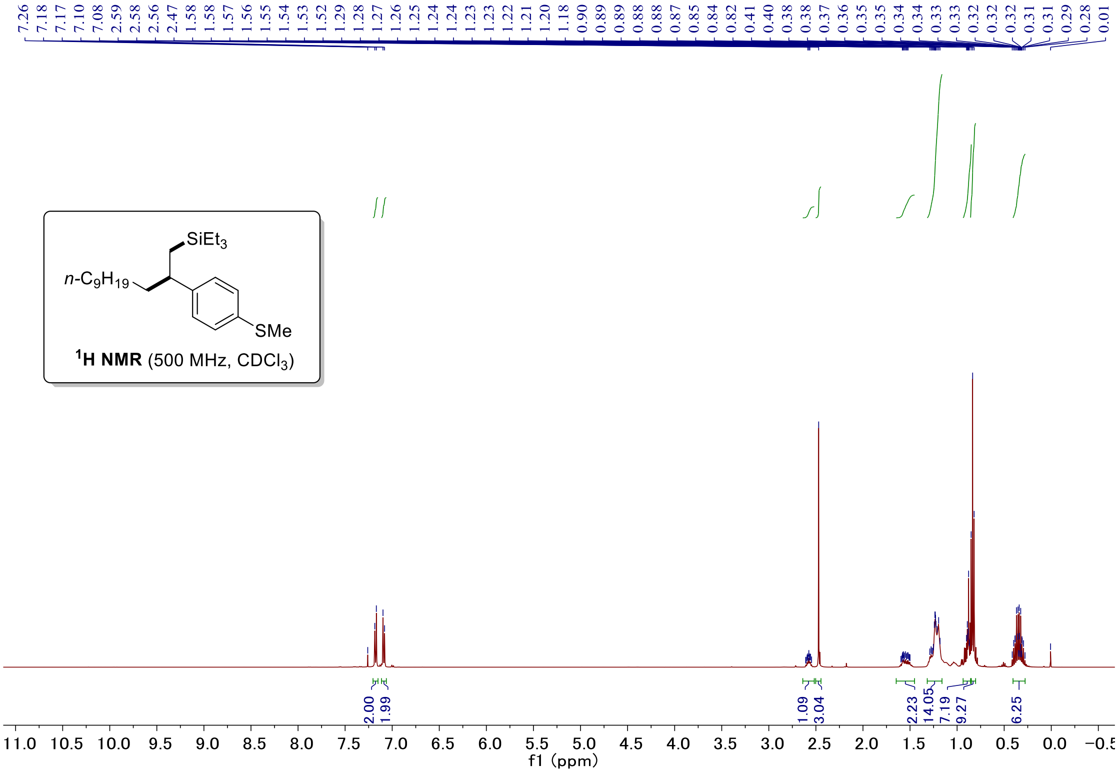

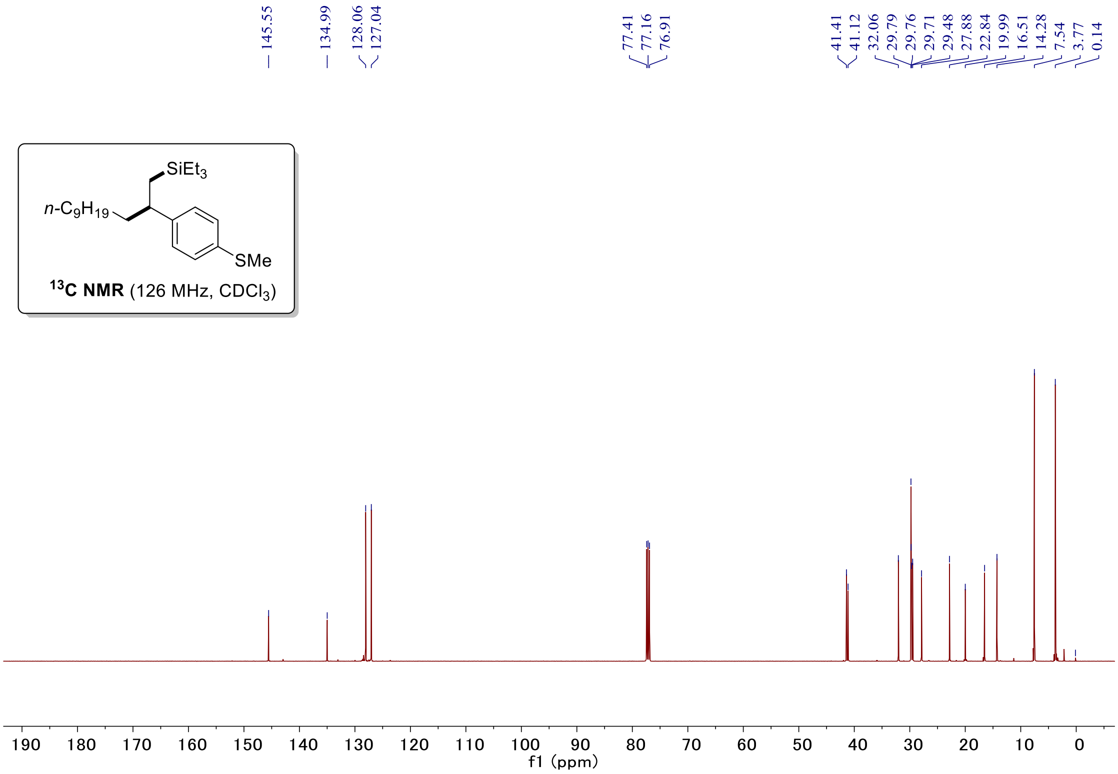

Triethyl(2-(4-(methylthio)phenyl)-5-phenylpentyl)silane (5fi)

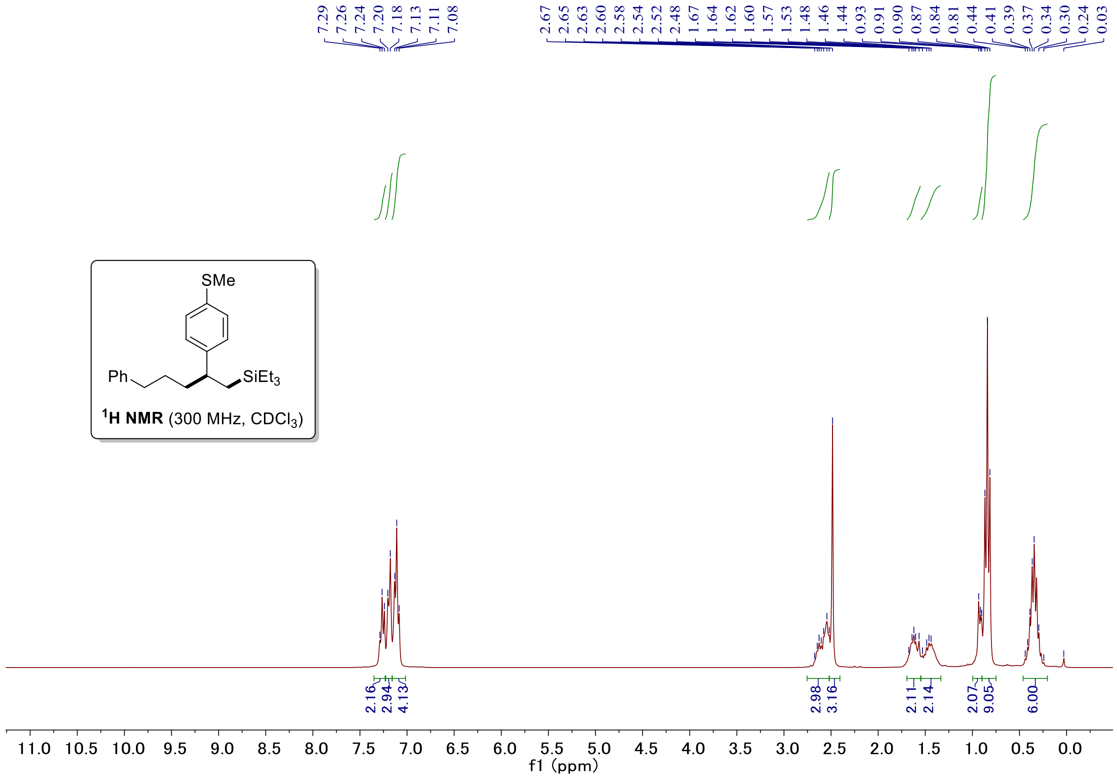

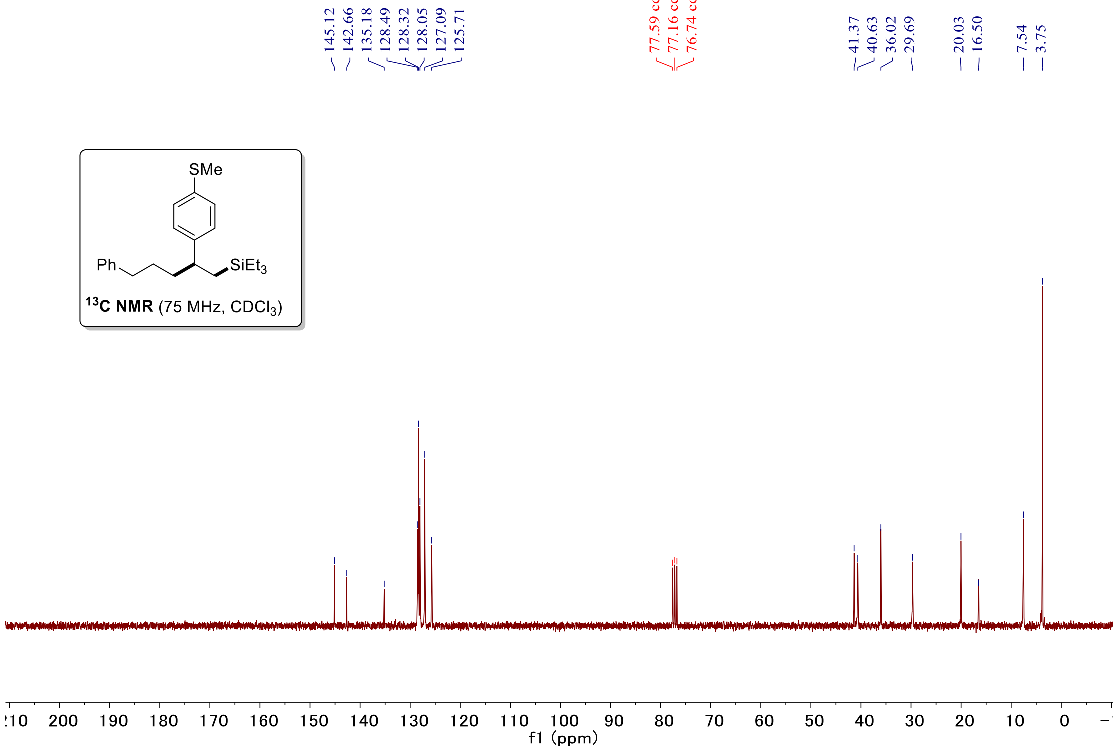

Triethyl(2-(4-(methylthio)phenyl)-4-phenylbutyl)silane (5gi)

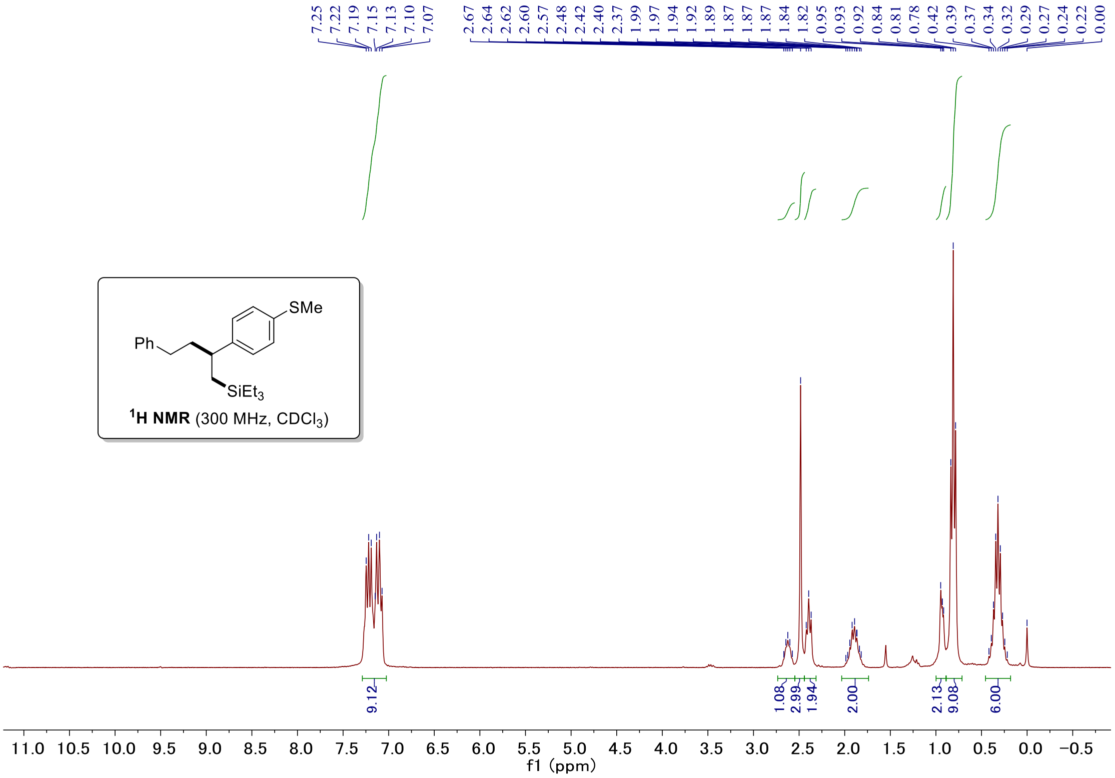

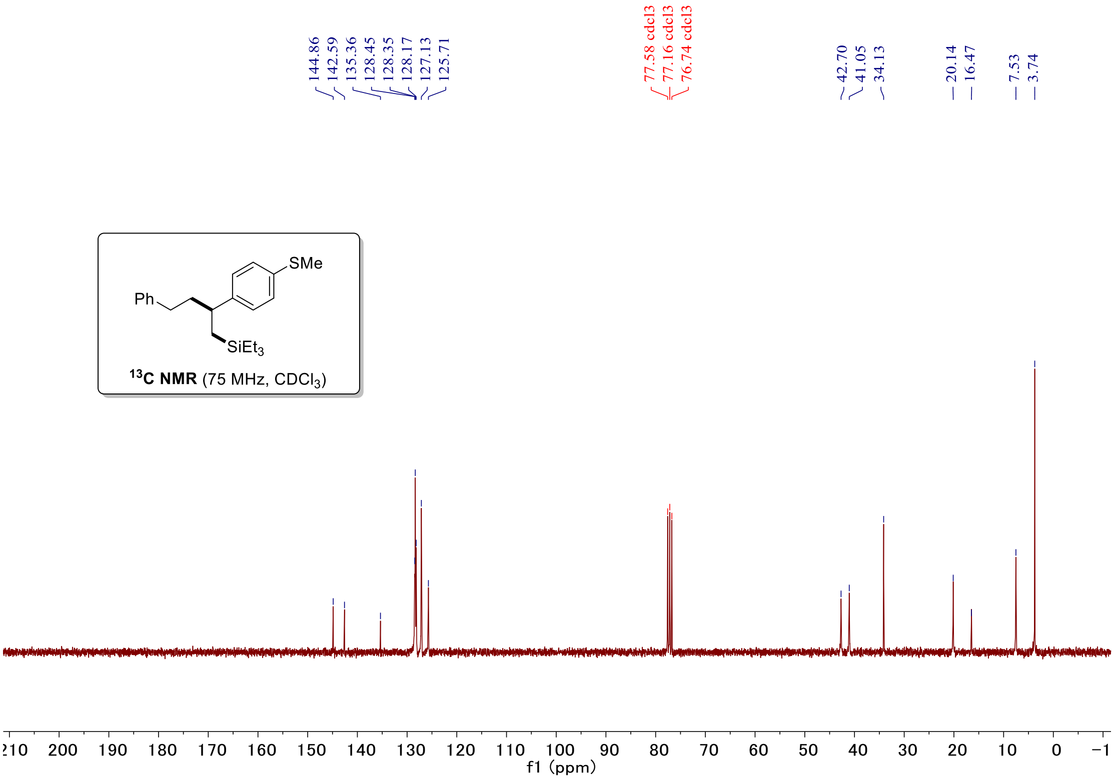

(5-(Adamantan-1-yl)-2-(*p*-tolyl)pentyl)triethylsilane (5hc)

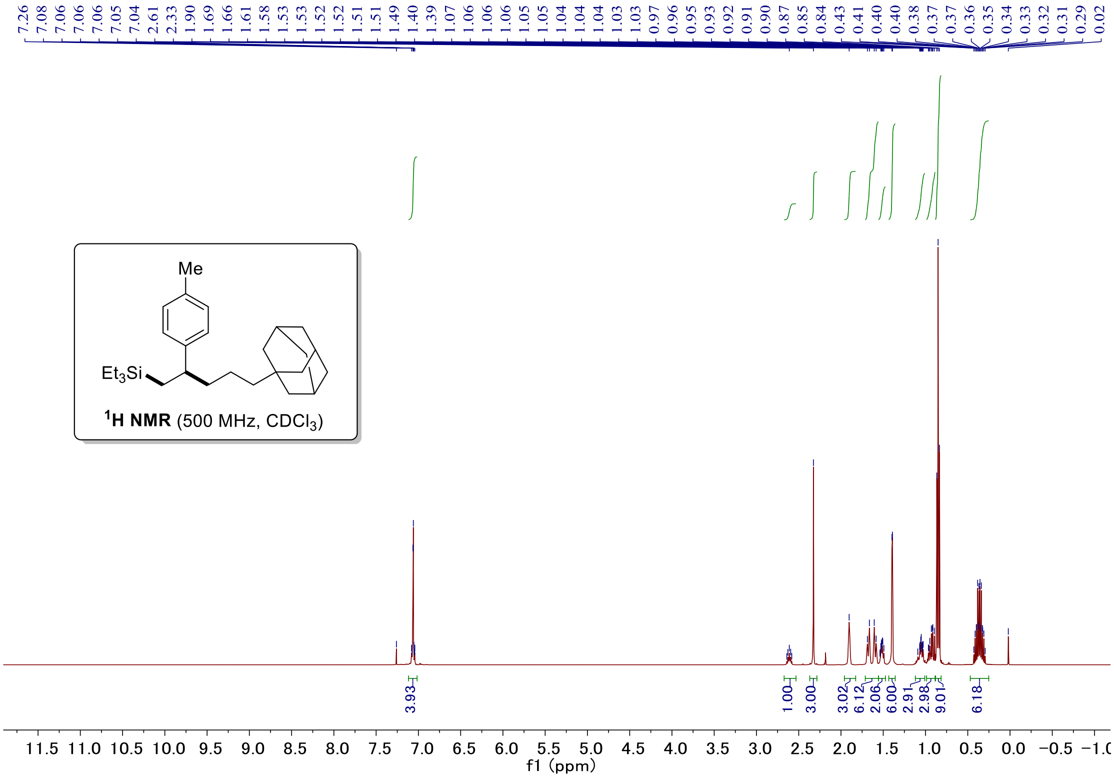

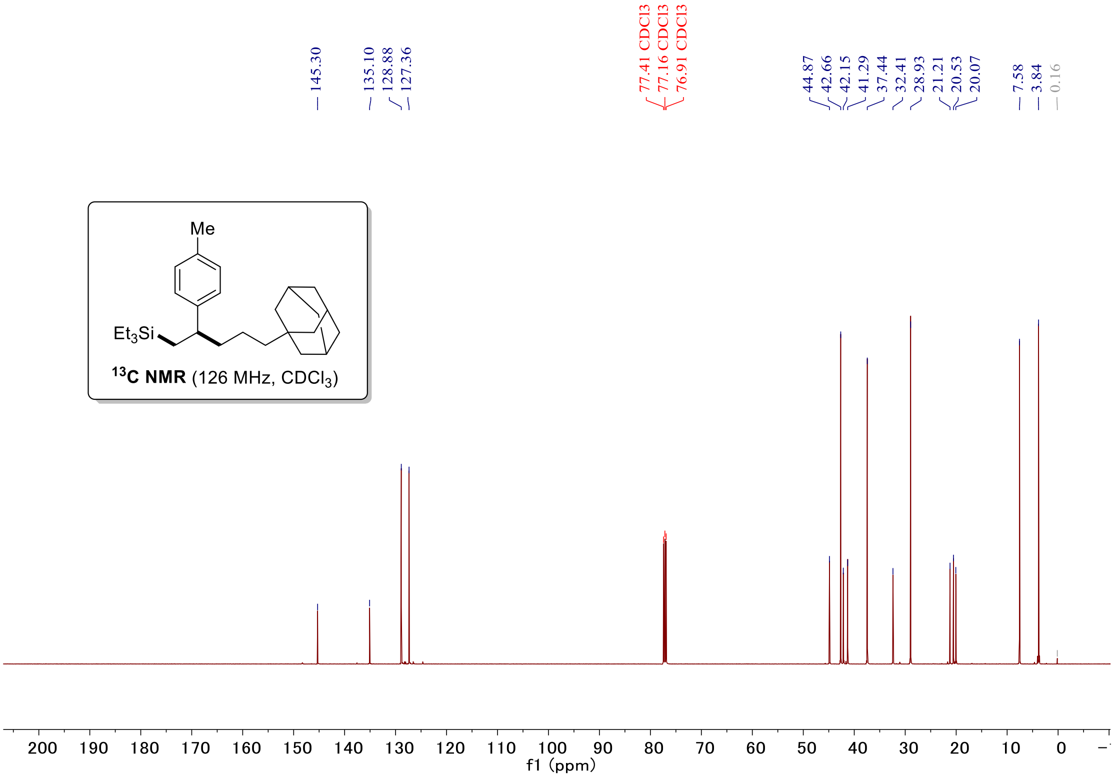

3-Cyclohexyl-2,3-diphenylpropyl)triethylsilane (5ia)

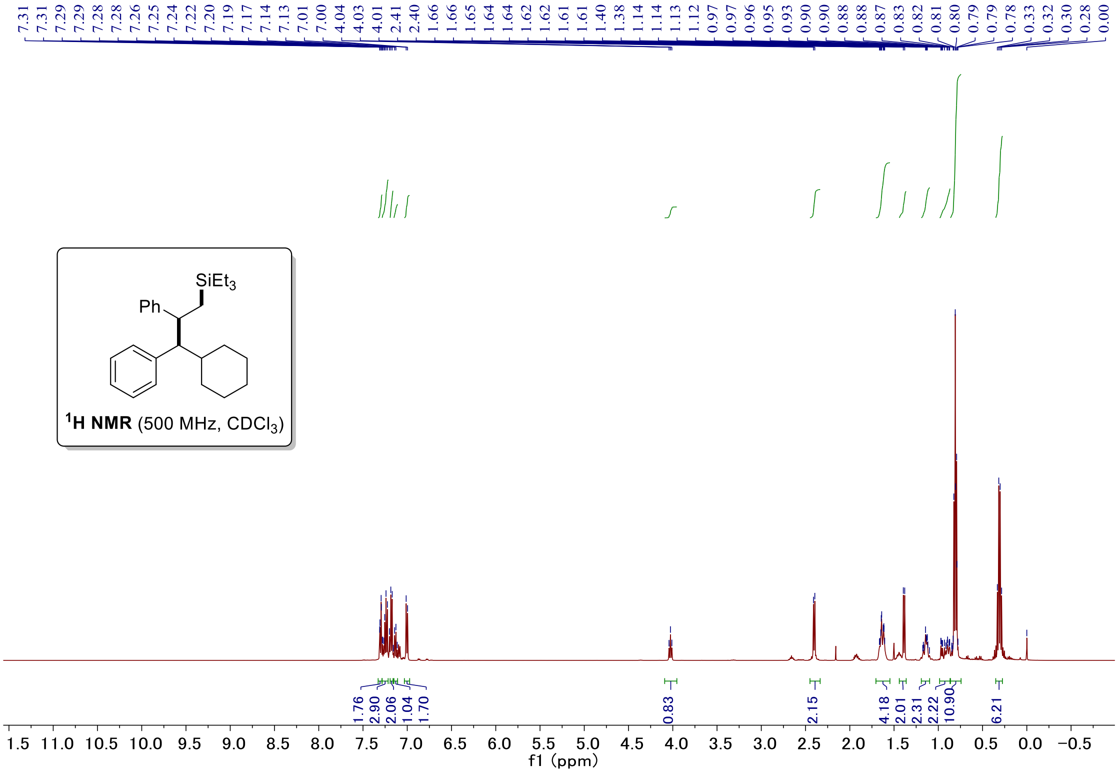

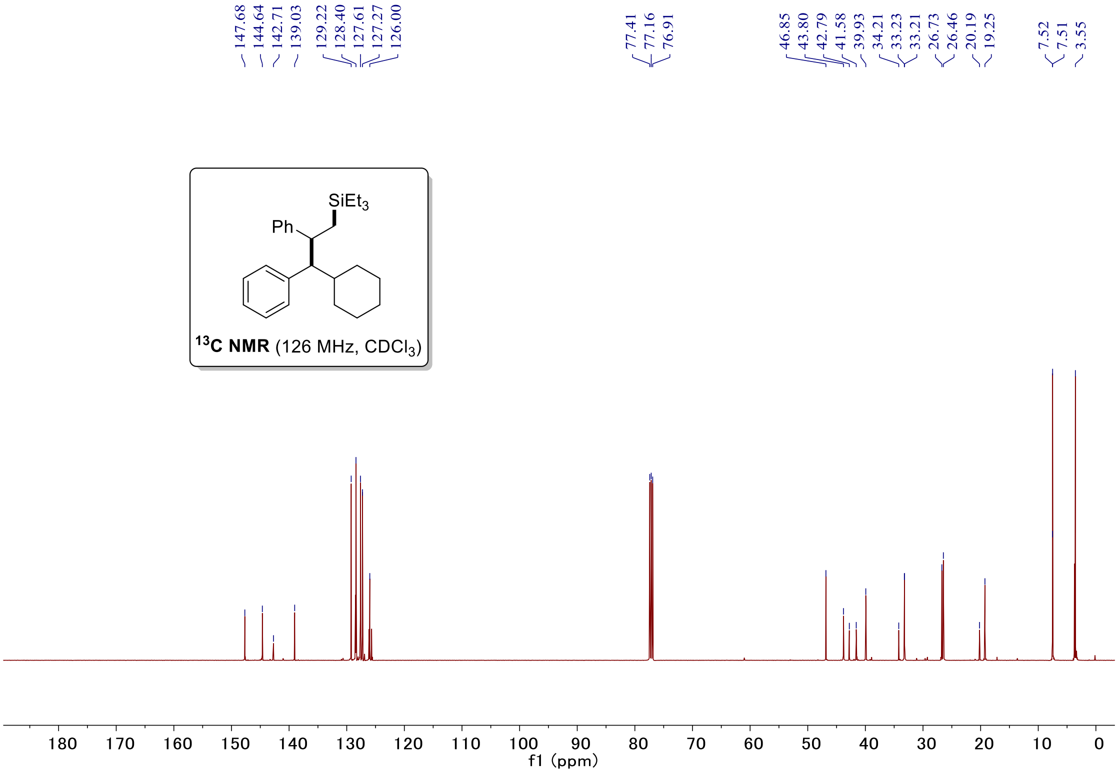

Triethyl(2,3,5-triphenylpentyl)silane (5ja)

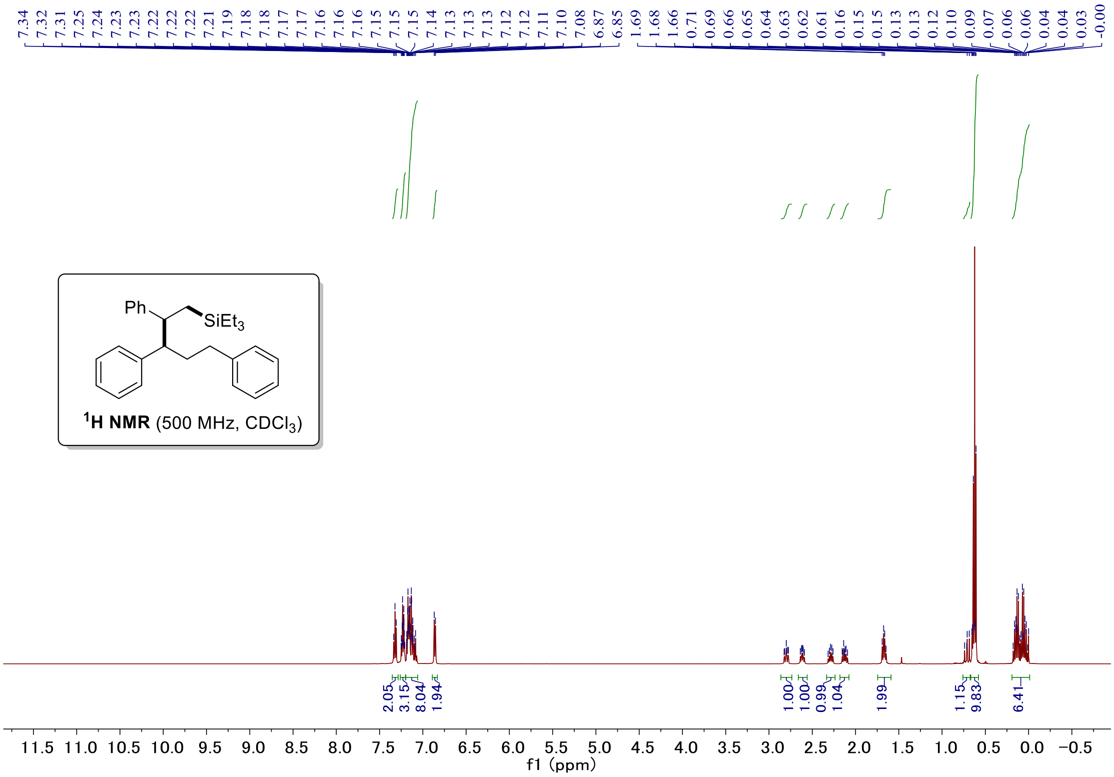

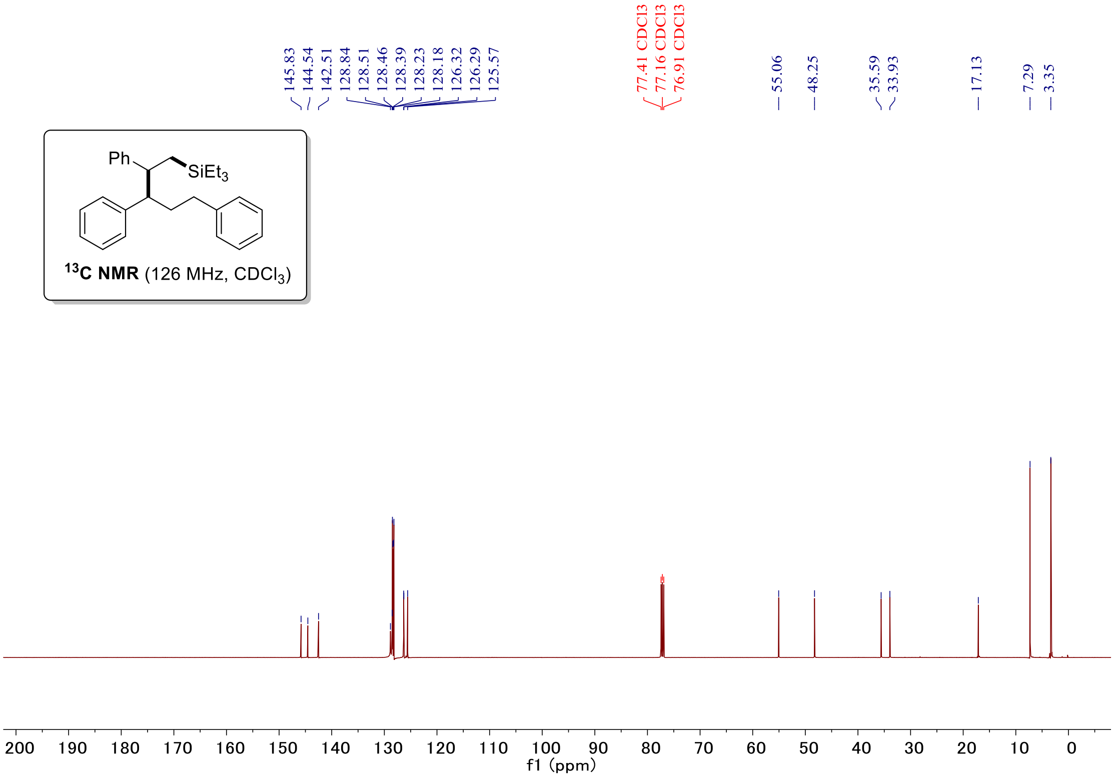

Triethyl((2*S*)-2-phenyl-2-(4-(((2,5,7,8-tetramethyl-2-(4,8,12-trimethyltridecyl)chroman-6-yl)oxy)methyl)phenyl)ethyl)silane (4wa)

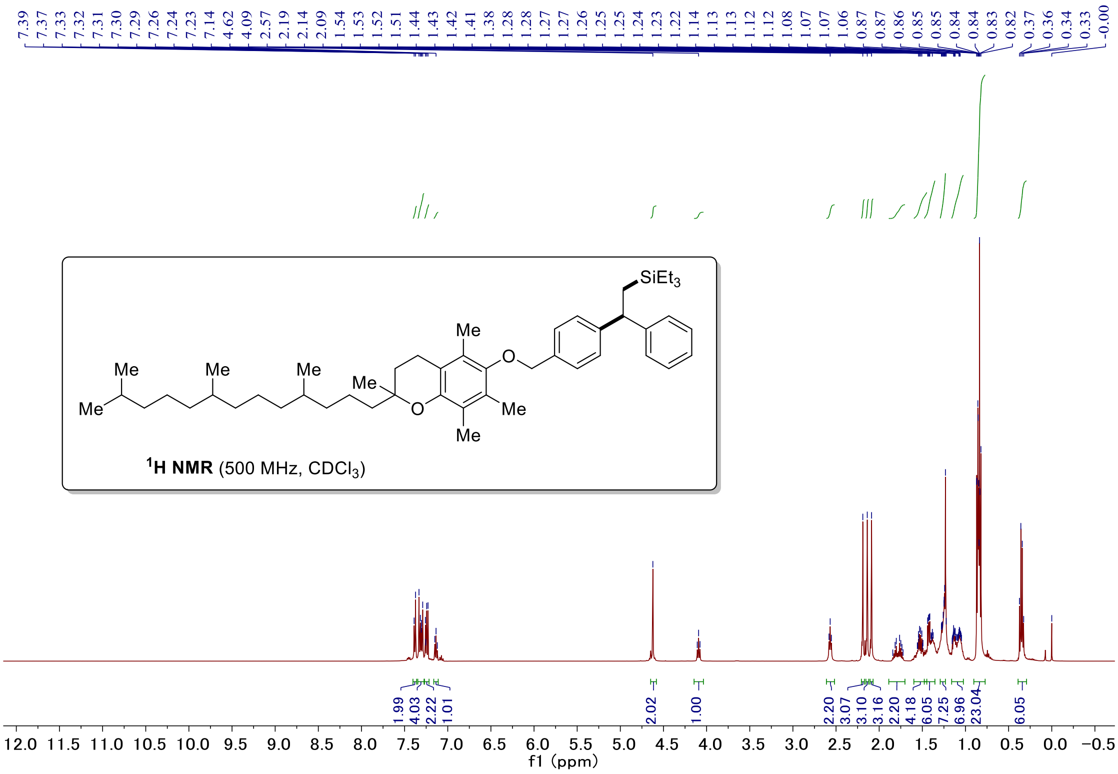

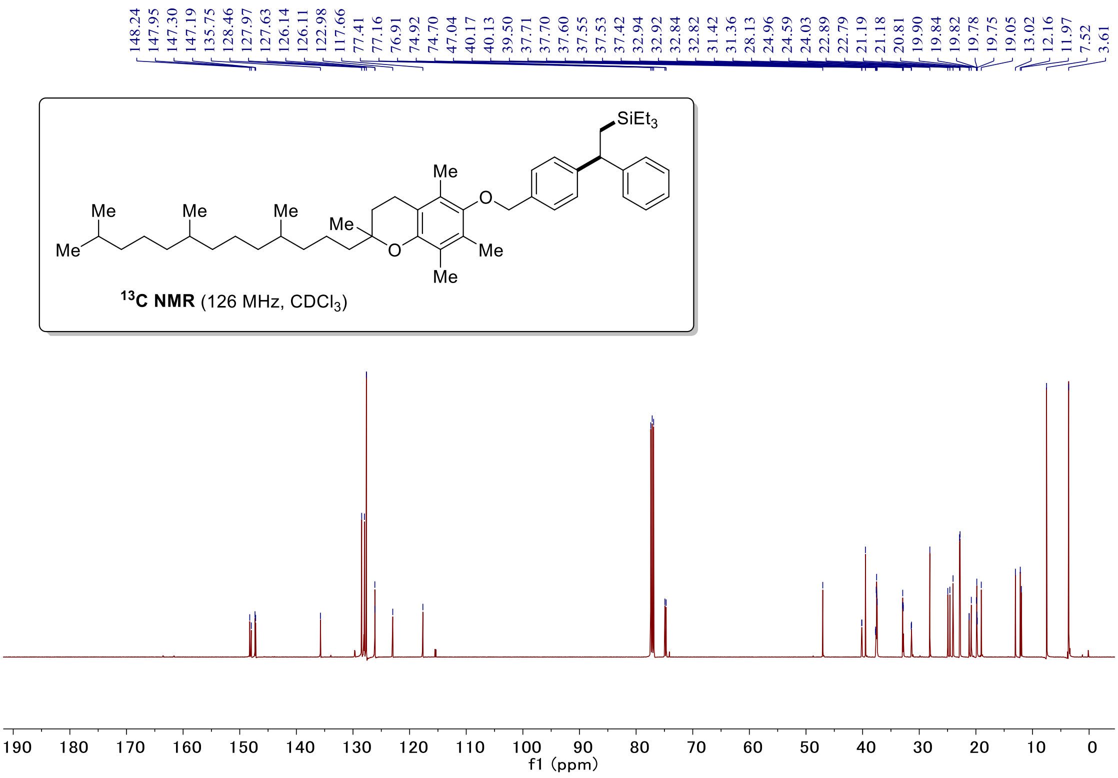

Menthol derivative (4xa)

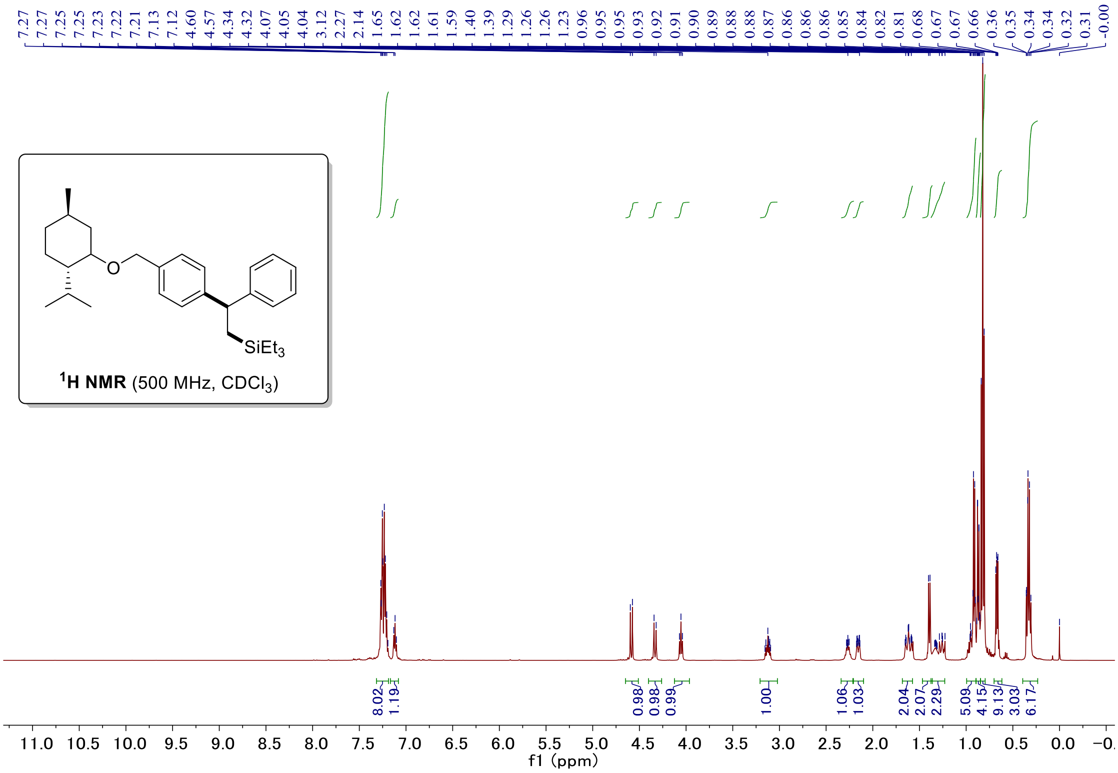

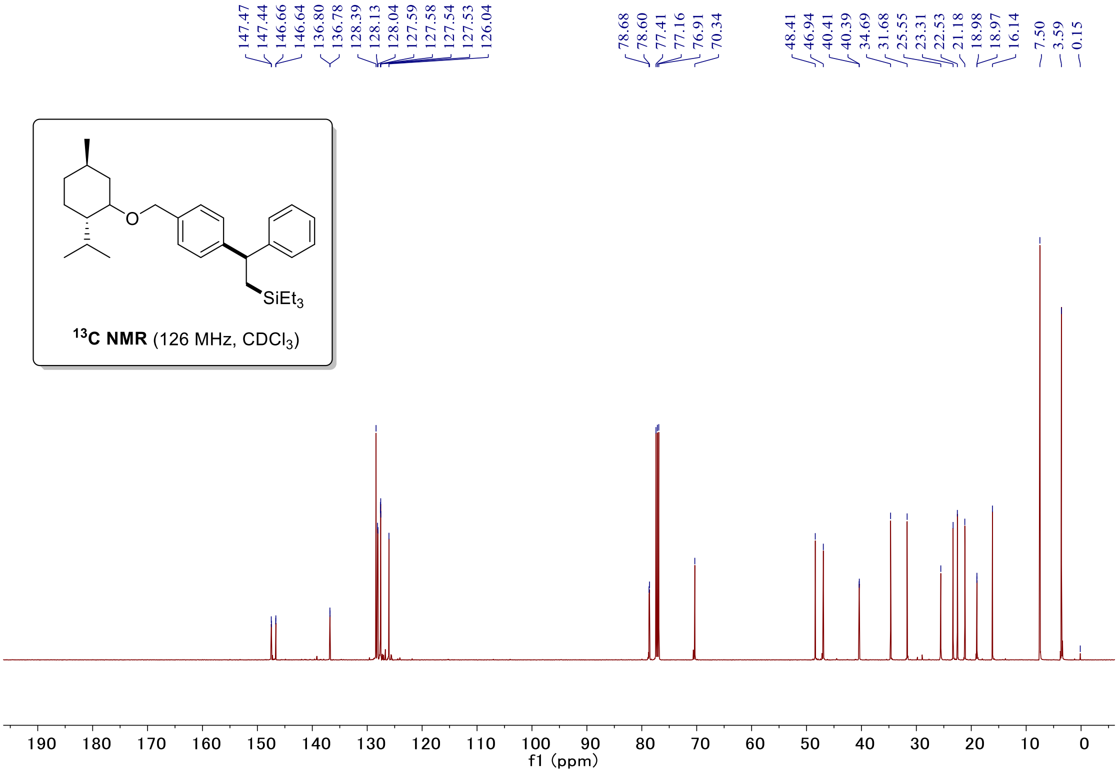

Estrone derivative (4ya)

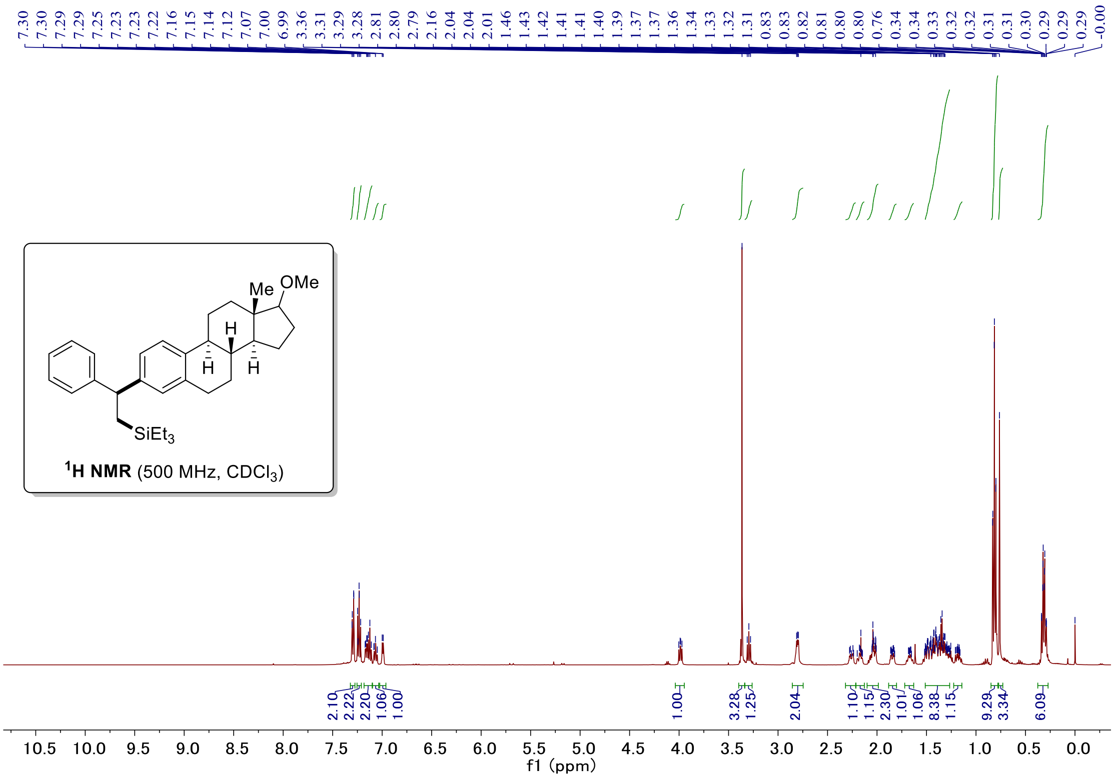

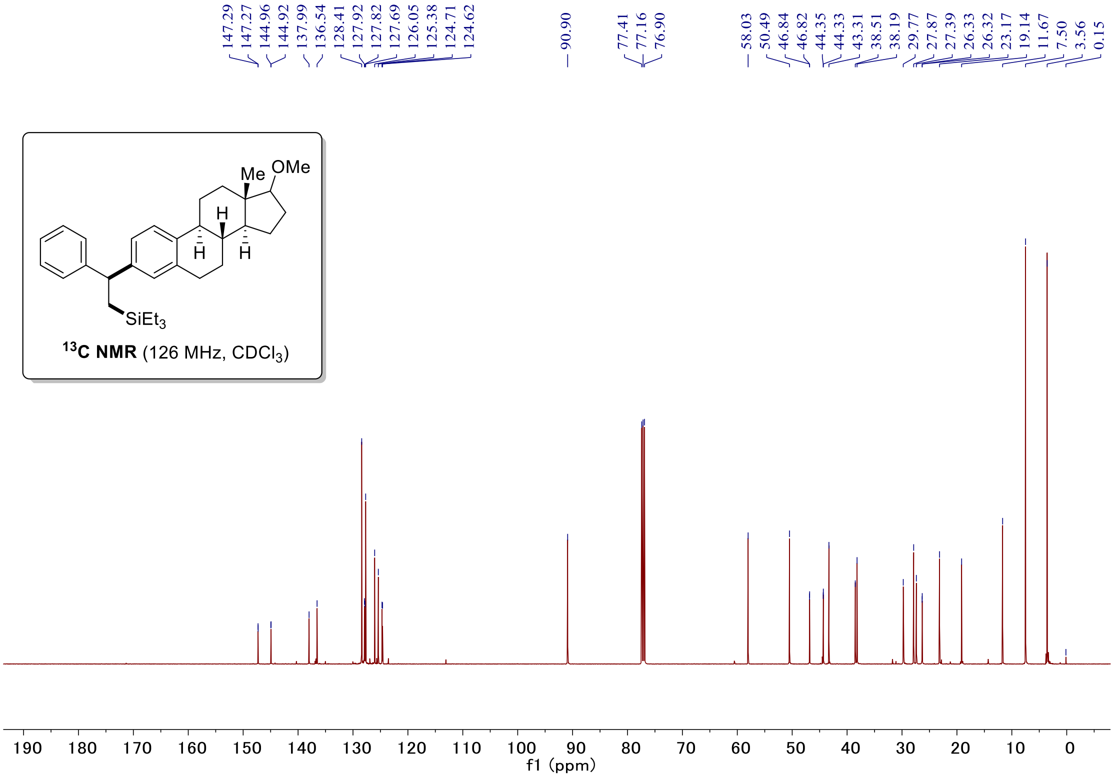

Estrone derivative (4am)

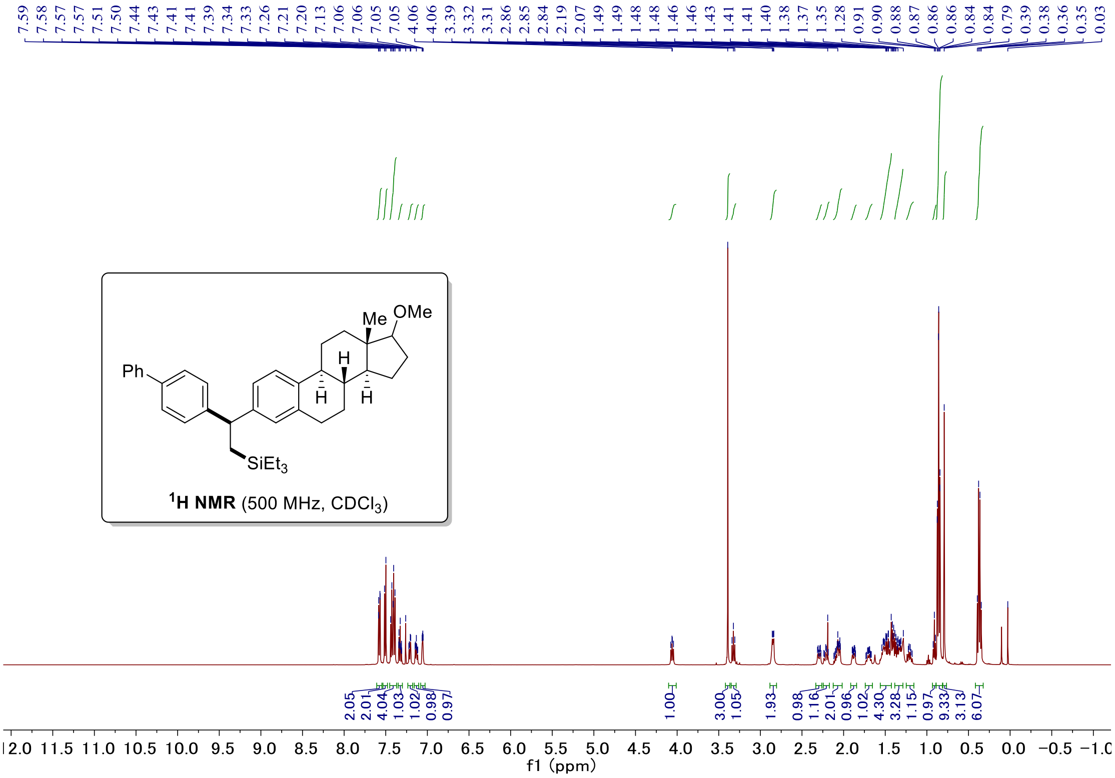

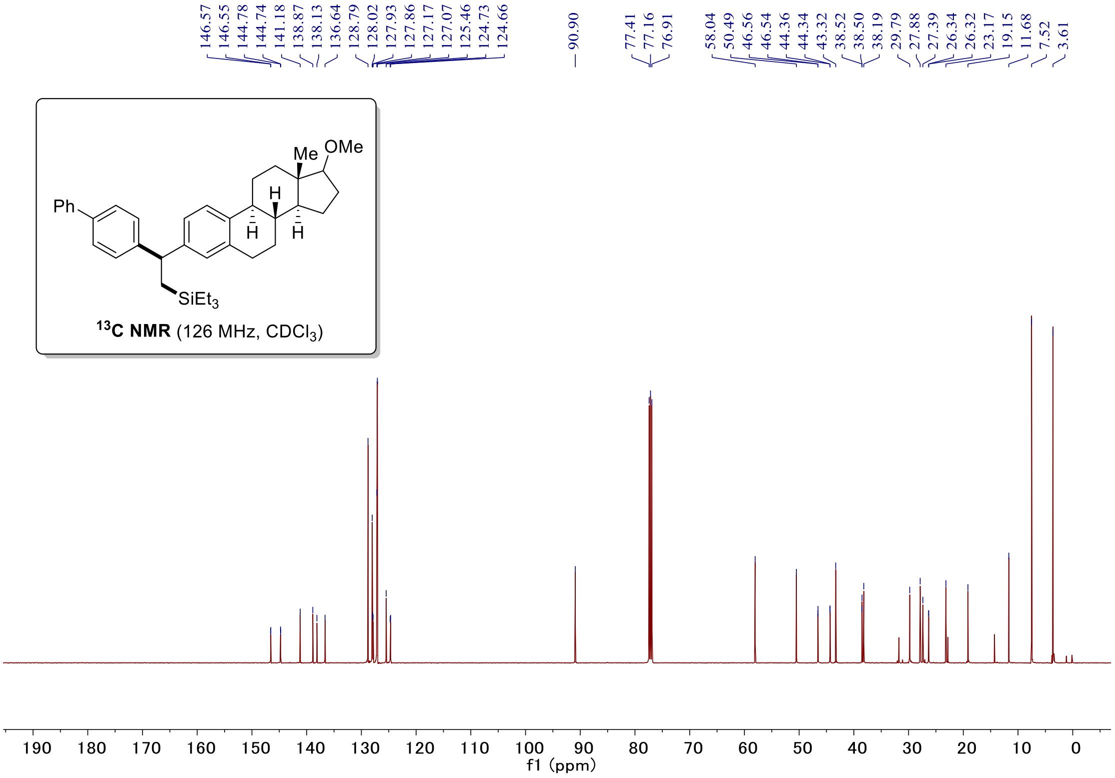

Liquid crystal material derivative (4zi)

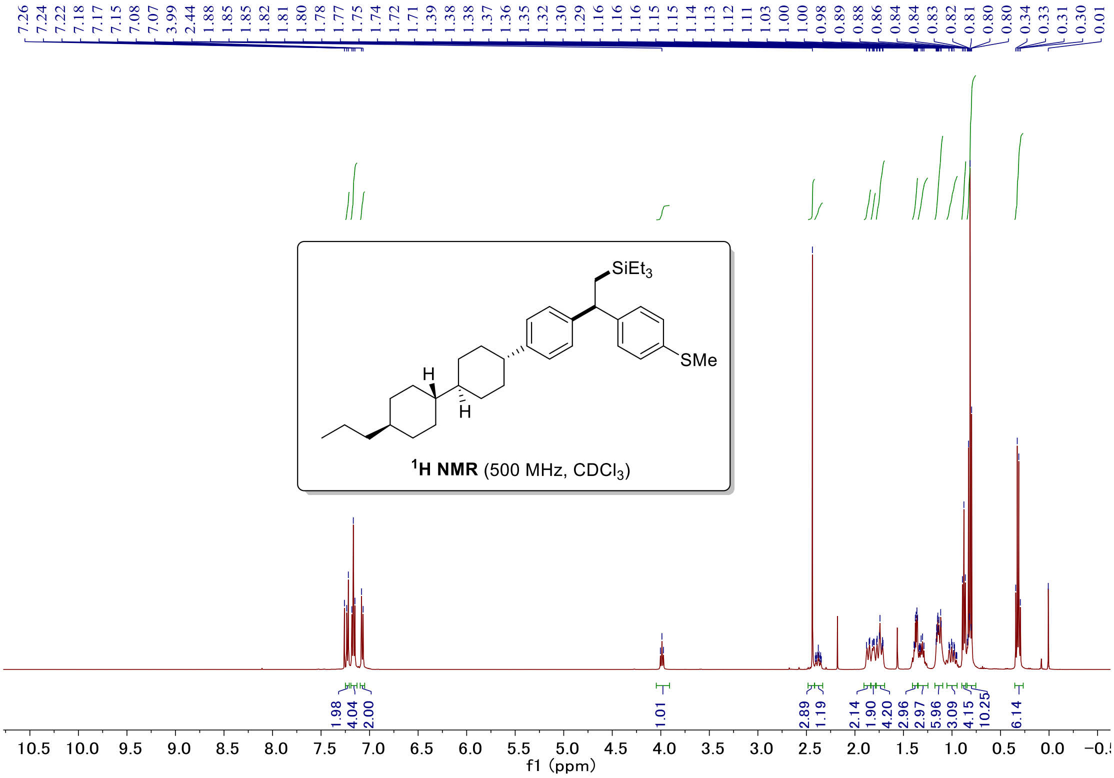

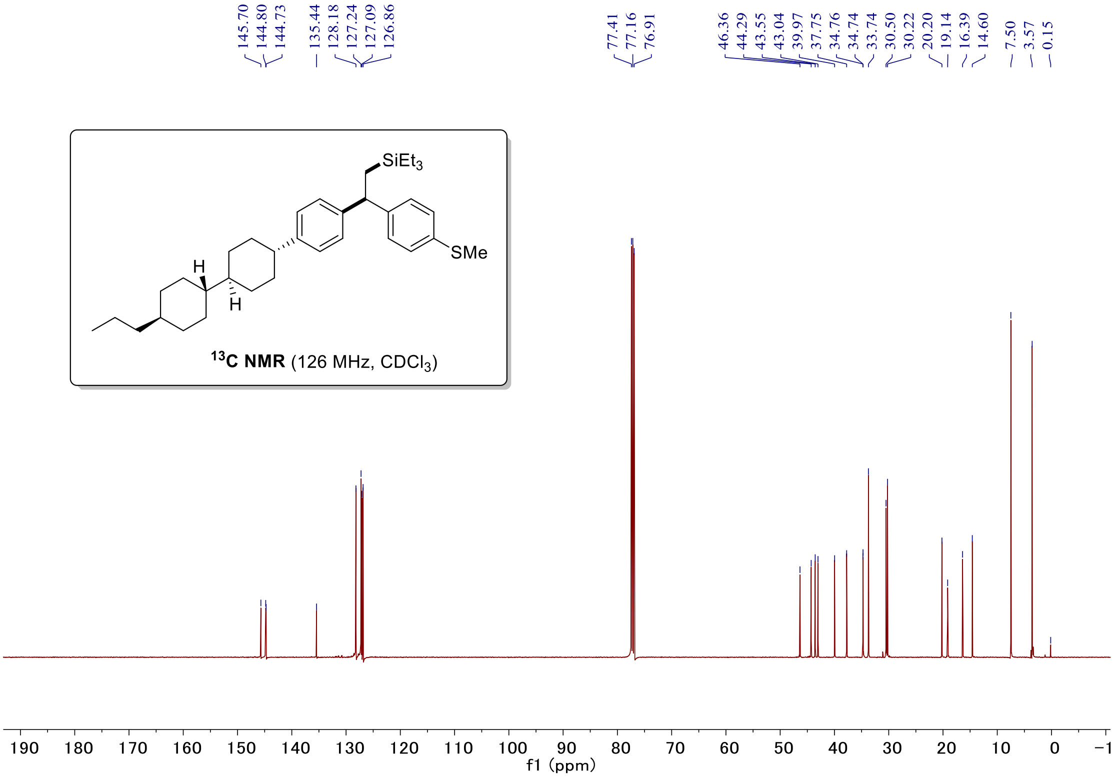

(4-(Biphenyl-4-yl)-4-phenylbut-2-en-1-yl)triethylsilane (4an)

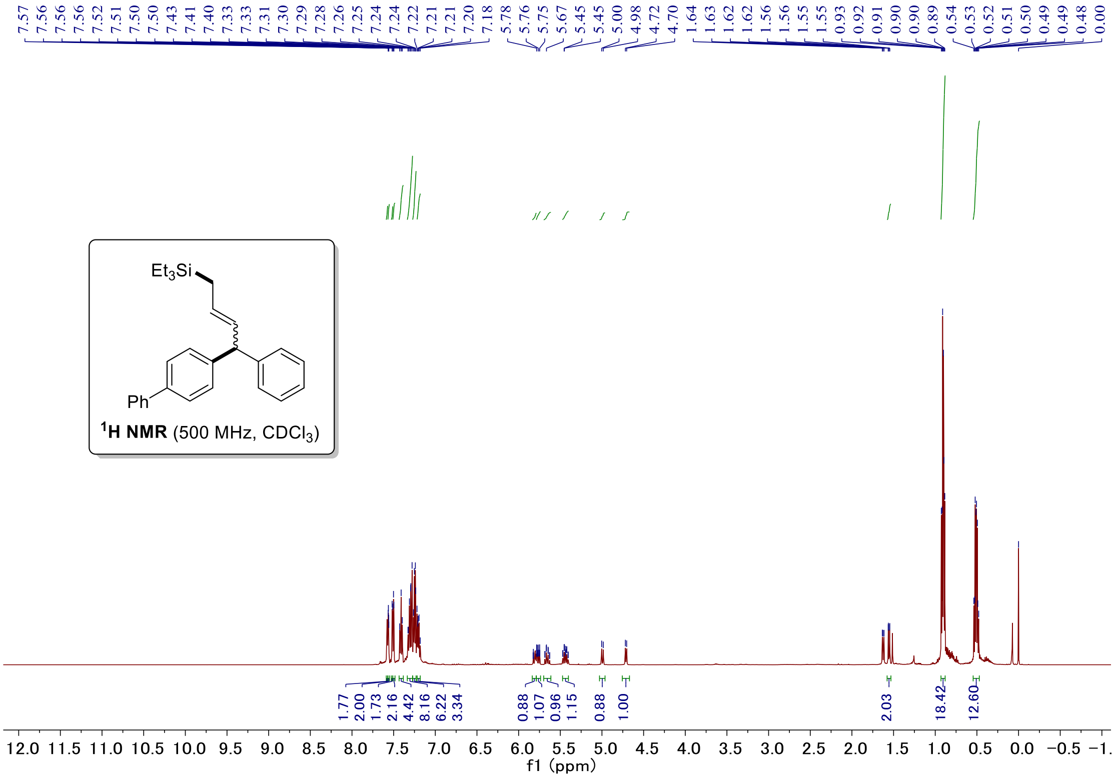

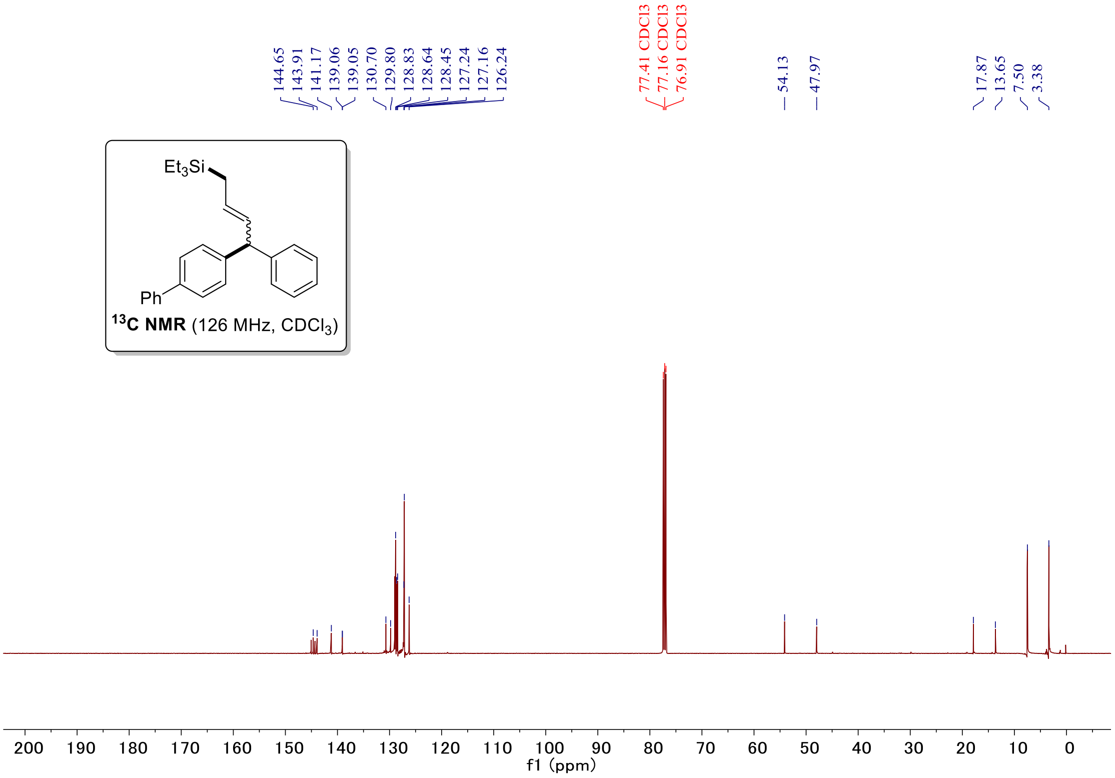

(2-(Biphenyl-4-yl)-2-phenylbut-3-en-1-yl)triethylsilane (4ao)

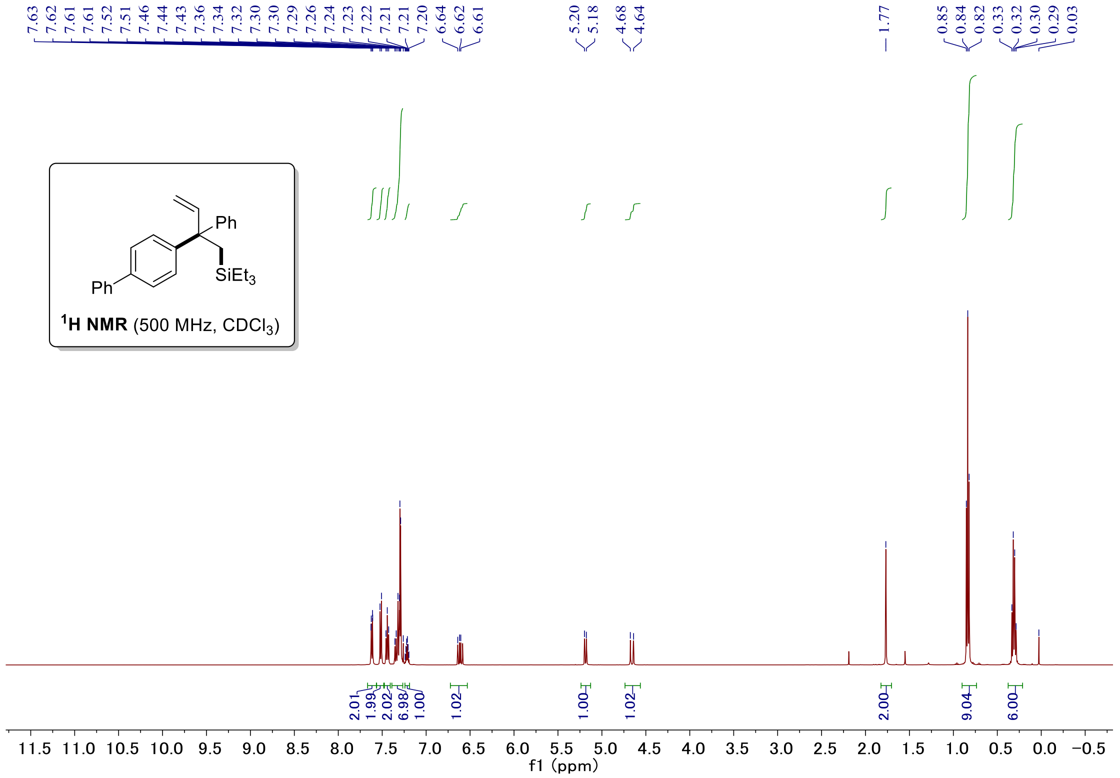

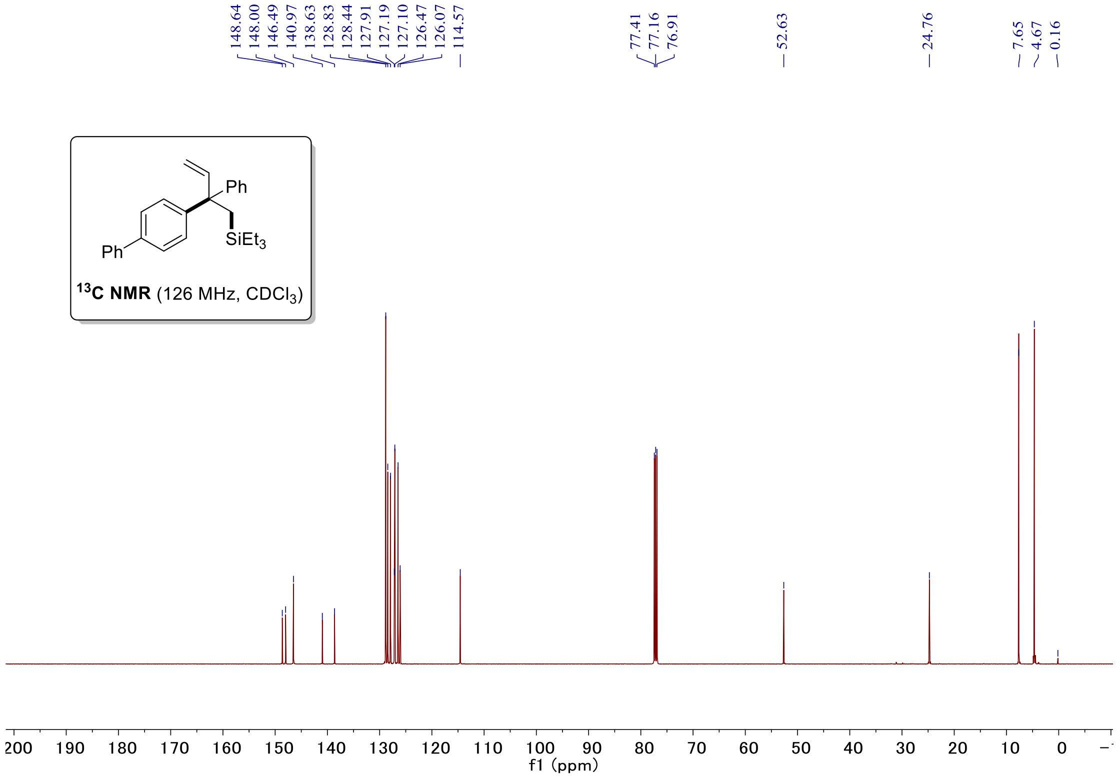

1-(But-3-en-1-yl)-2-fluorobenzene (6a)

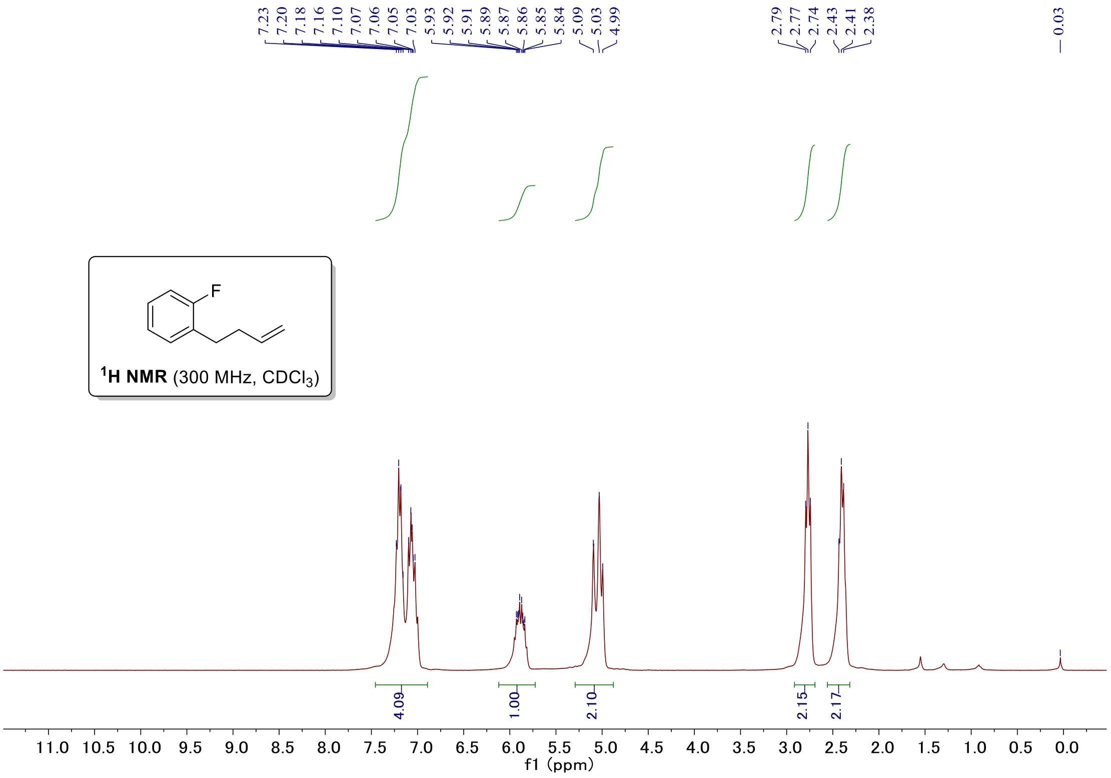

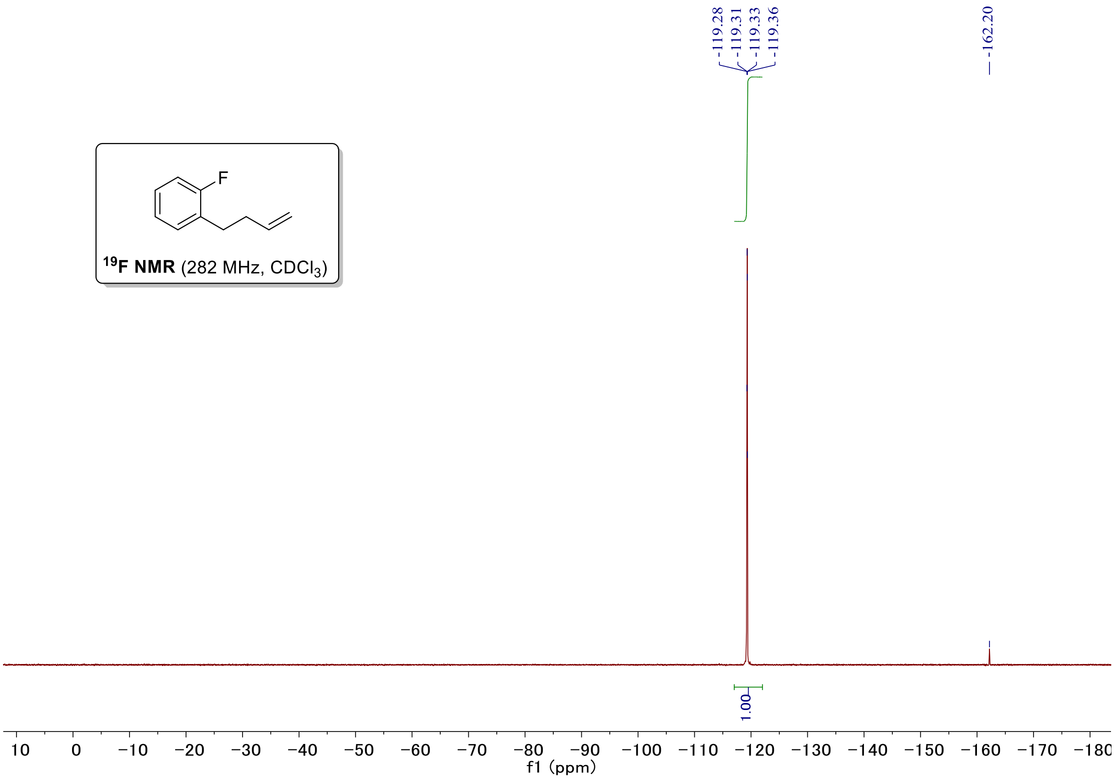

1-Fluoro-2-(3-phenylbut-3-en-1-yl)benzene (6b)

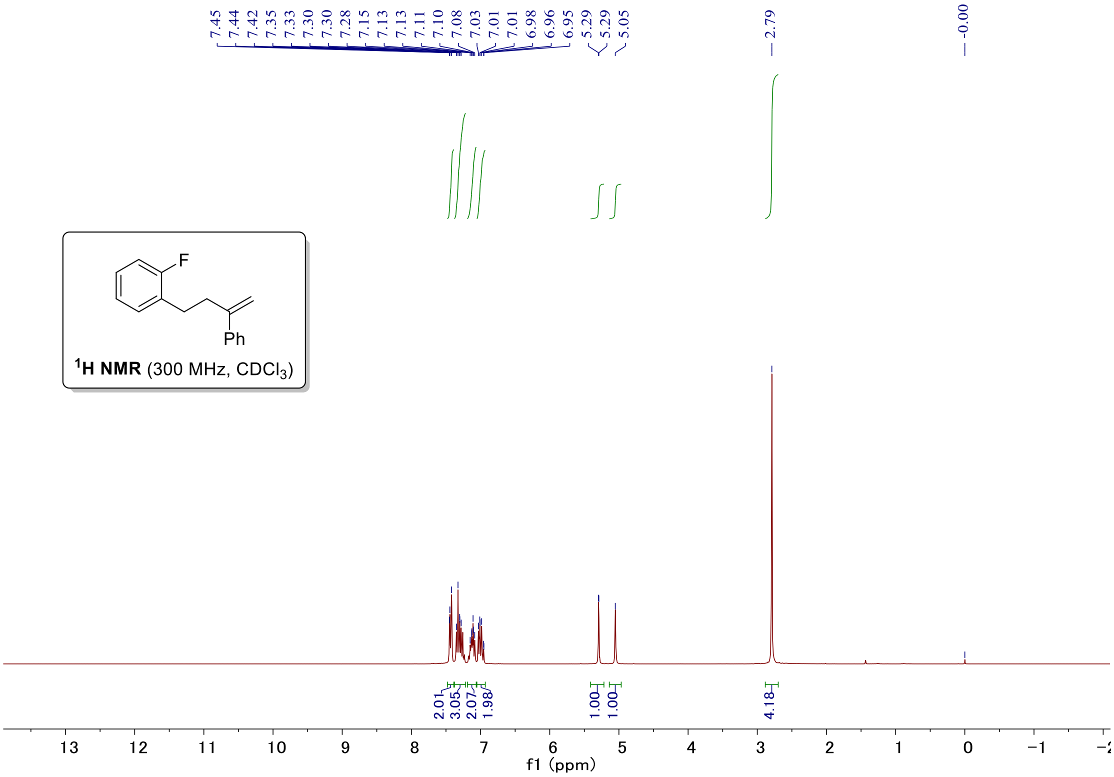

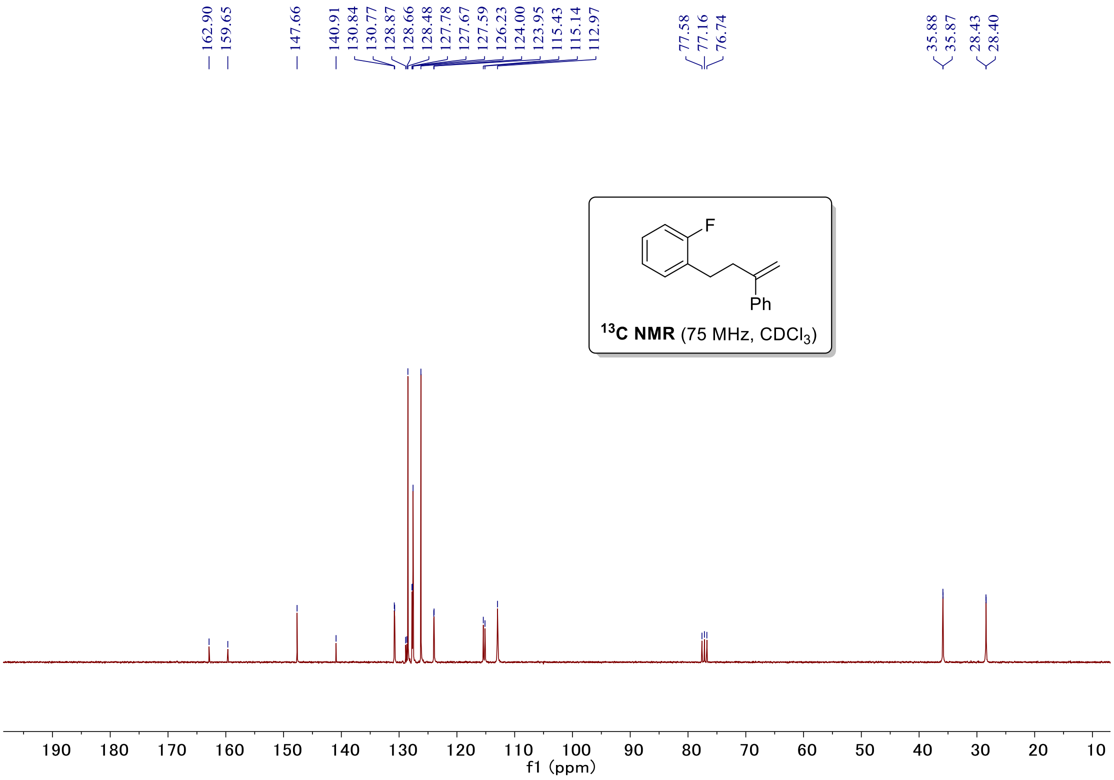

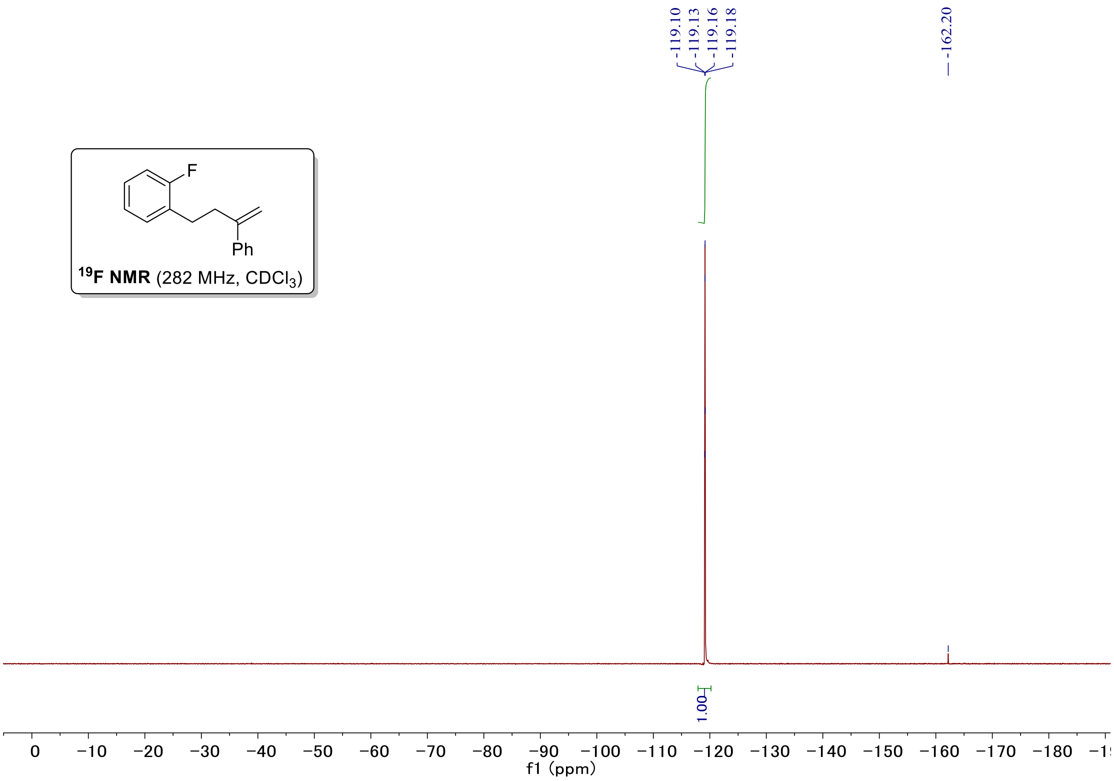

4-Fluoro-3-(3-phenylbut-3-en-1-yl)-1,1'-biphenyl (6c)

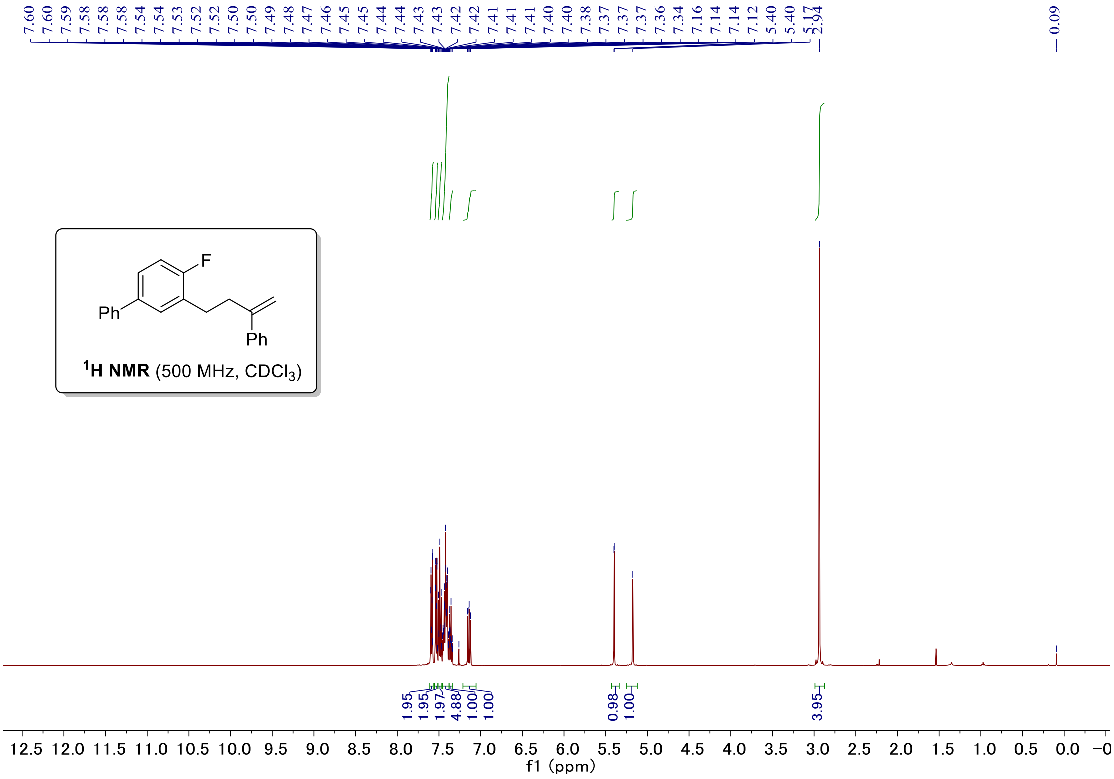

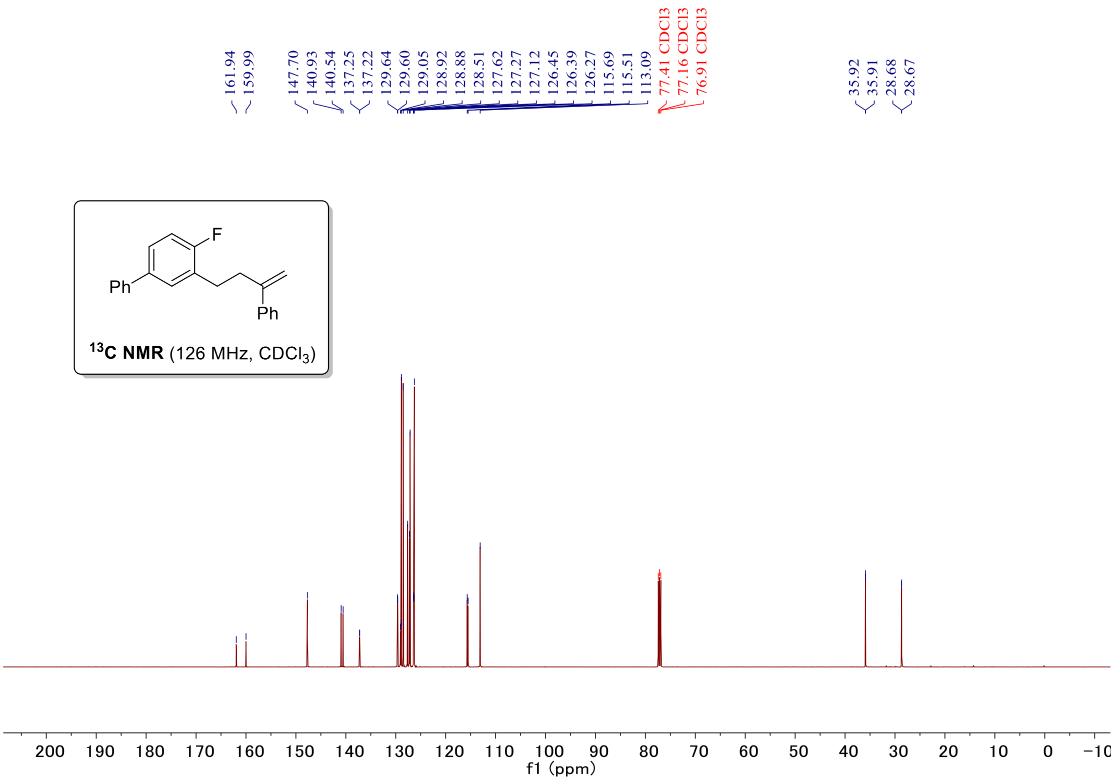
161.94
159.99
147.70
140.93
140.54
137.25
137.22
129.64
129.60
129.05
128.92
128.88
128.51
127.62
127.27
127.12
126.45
126.39
126.27
115.69
115.51
113.09
77.41 CDCl3
77.16 CDCl3
76.91 CDCl3
35.92
35.91
28.68
28.67
F
Ph
Ph
13C NMR (126 MHz, CDCl3)
f1 (ppm)

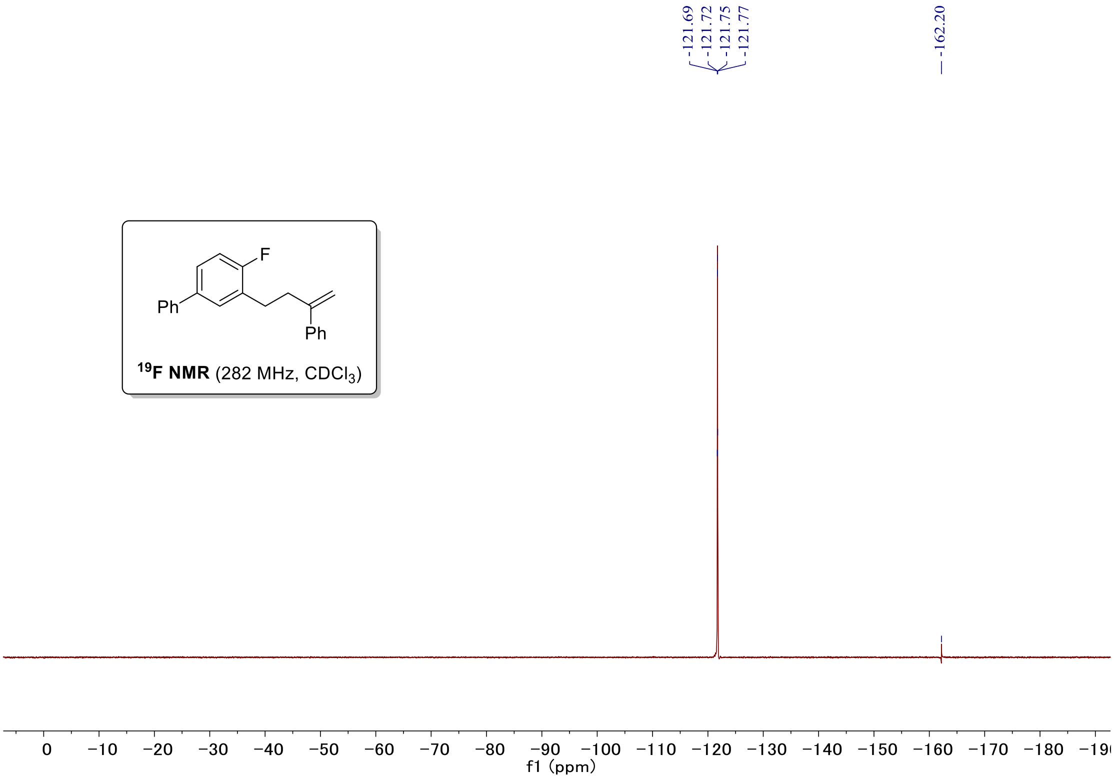
-121.69
-121.72
-121.75
-121.77
-162.20
F
Ph
Ph
19F NMR (282 MHz, CDCl3)
f1 (ppm)

5-(4-(2-Fluoro-5-methoxyphenyl)but-1-en-2-yl)benzo[*d*][1,3]dioxole (6d)

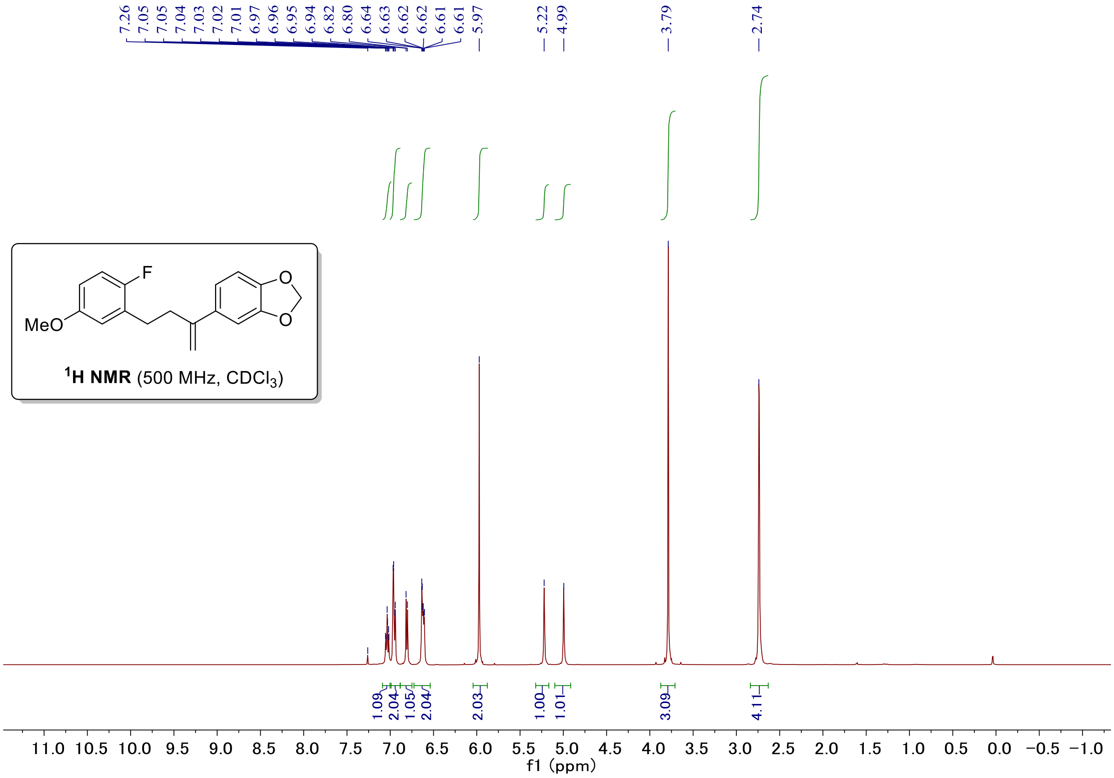

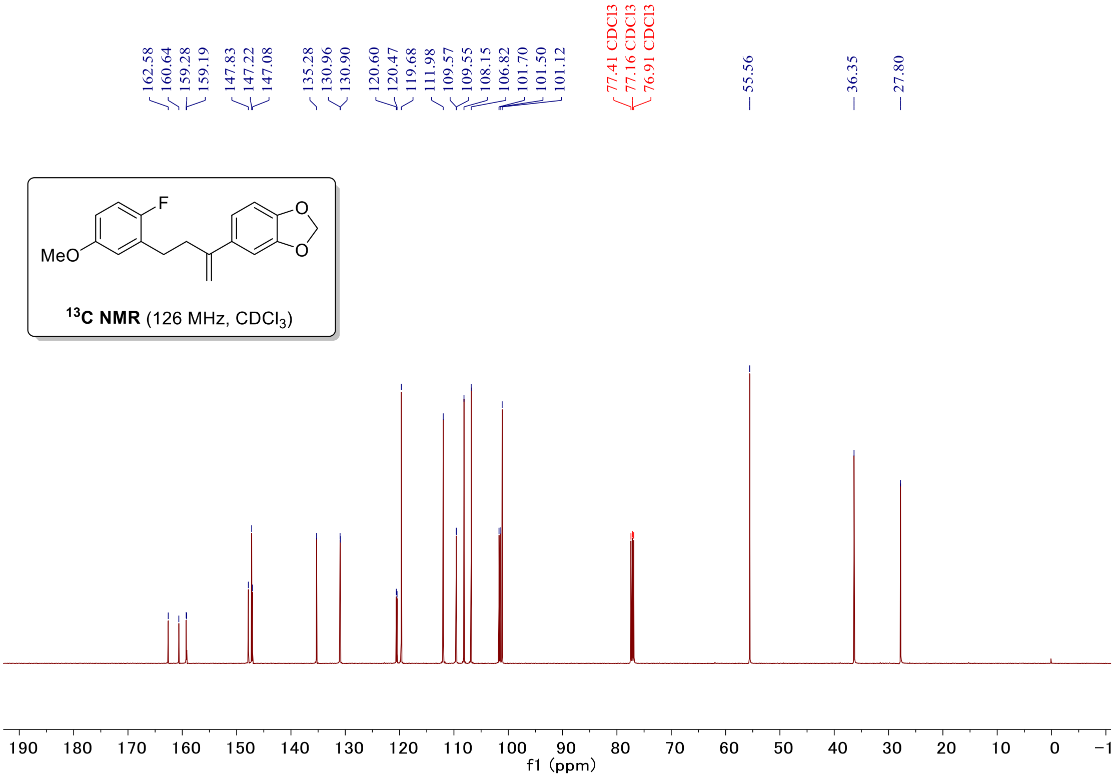

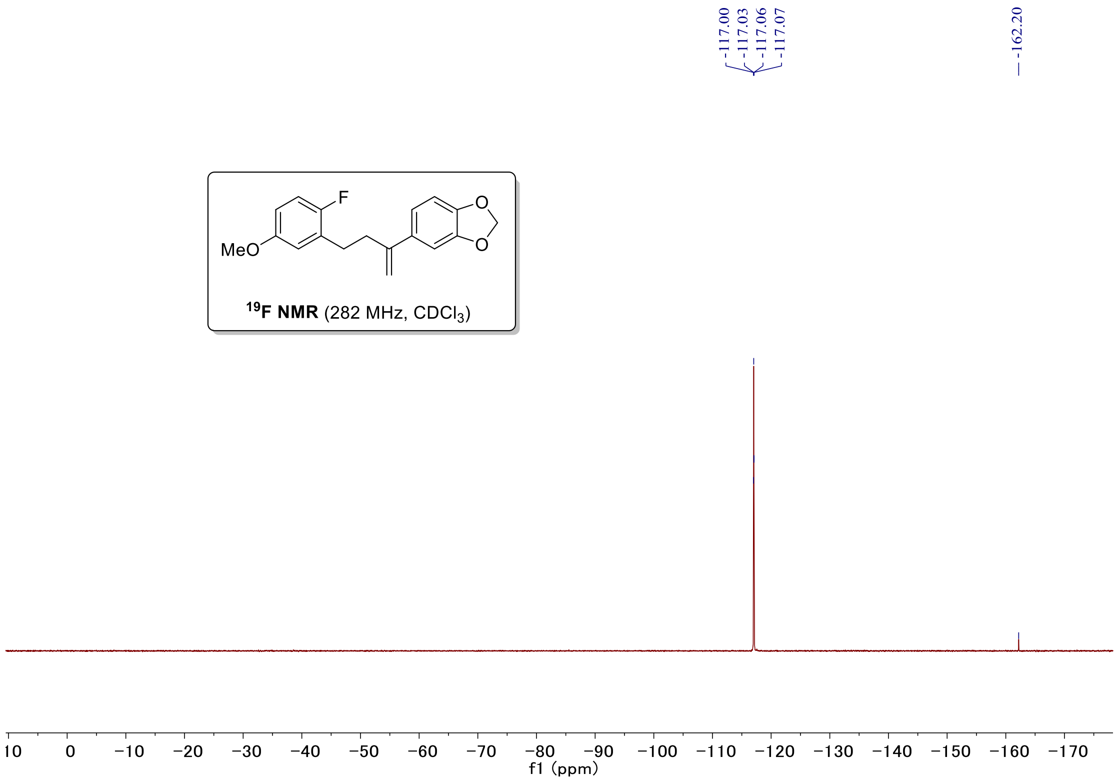

1-Fluoro-2-((3-phenylbut-3-en-1-yl)oxy)benzene (6e)

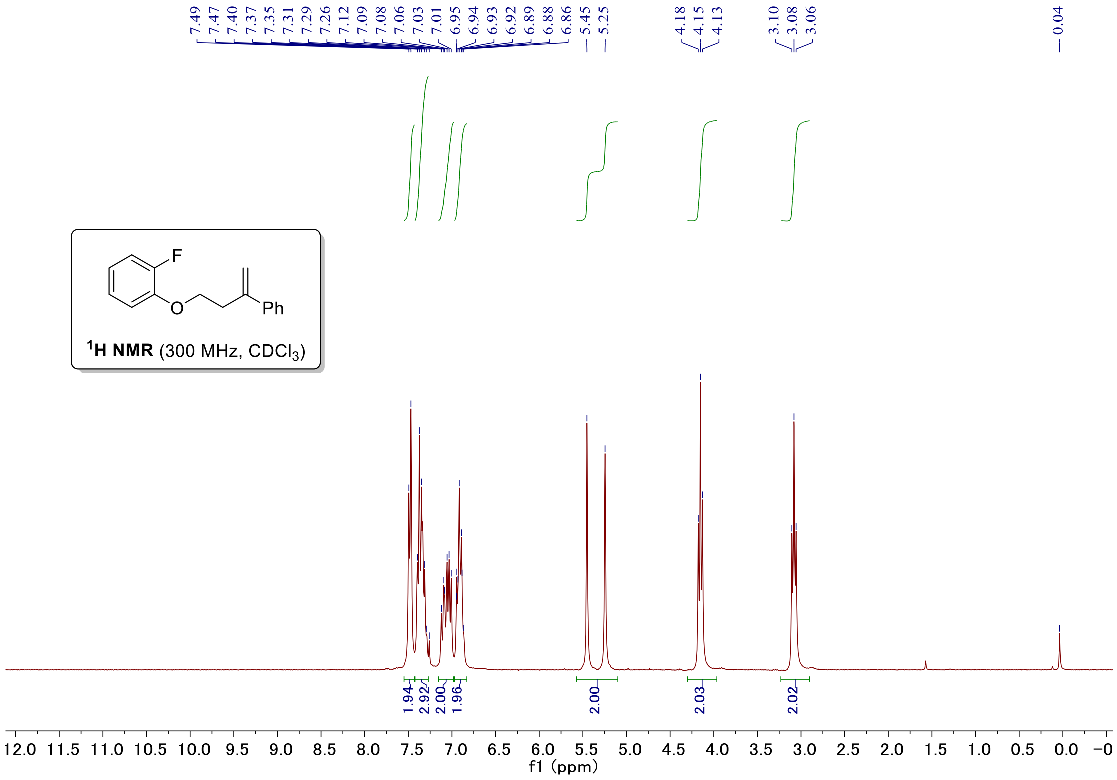

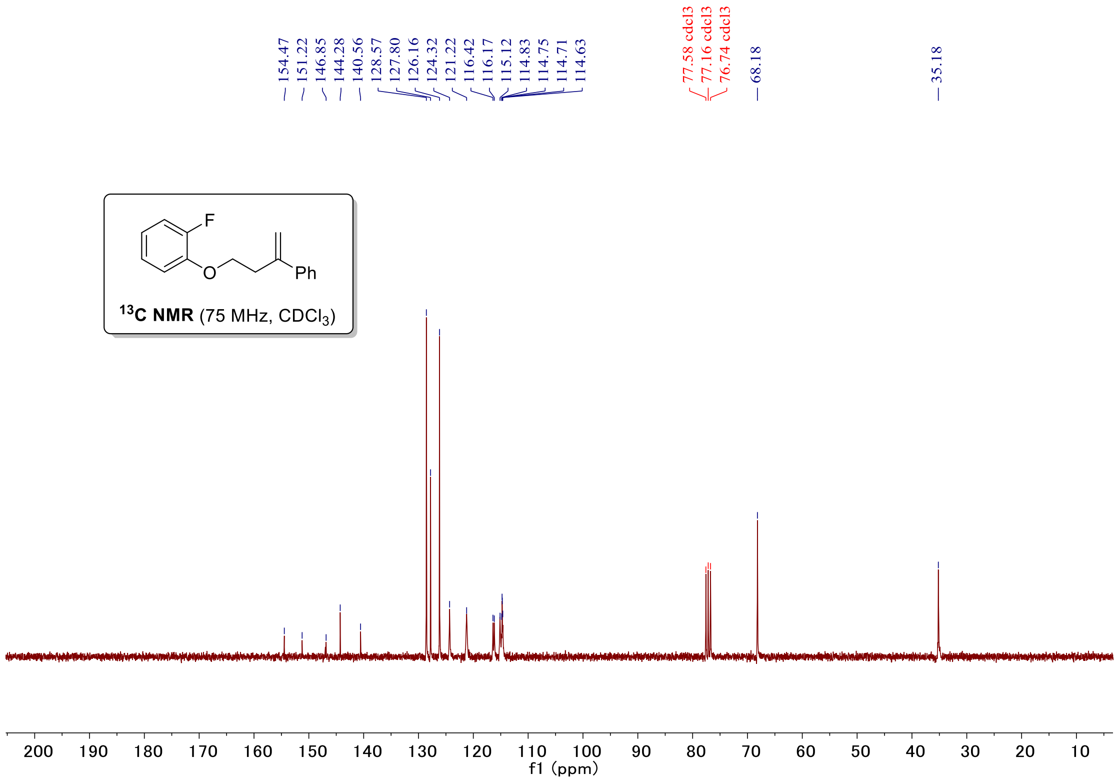
154.47
151.22
146.85
144.28
140.56
128.57
127.80
126.16
124.32
121.22
116.42
116.17
115.12
114.83
114.75
114.71
114.63
77.58 cdcl3
77.16 cdcl3
76.74 cdcl3
68.18
35.18
F
O
Ph
13C NMR (75 MHz, CDCl3)
200 190 180 170 160 150 140 130 120 110 100 90 80 70 60 50 40 30 20 10
f1 (ppm)

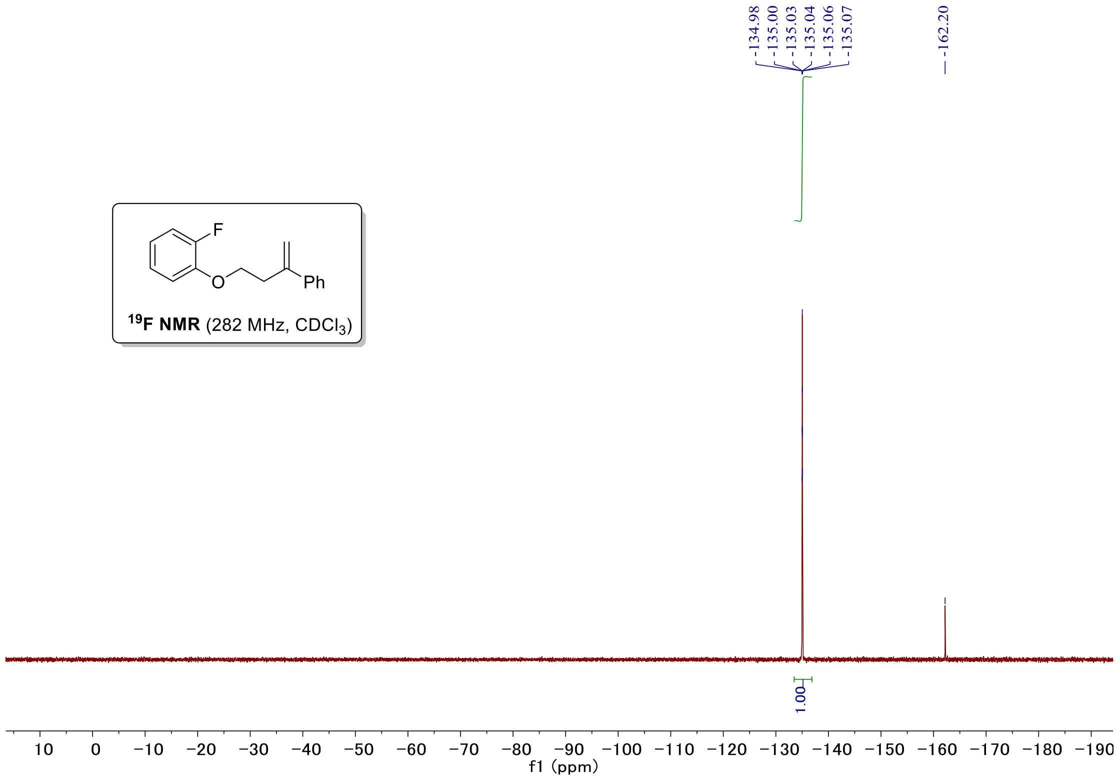
-134.98
-135.00
-135.03
-135.04
-135.06
-135.07
-162.20
F
O
Ph
19F NMR (282 MHz, CDCl3)
1.00
10 0 −10 −20 −30 −40 −50 −60 −70 −80 −90 −100 −110 −120 −130 −140 −150 −160 −170 −180 −190
f1 (ppm)

(2-(2-(But-3-en-1-yl)phenyl)-2-phenylethyl)triethylsilane (7aa)

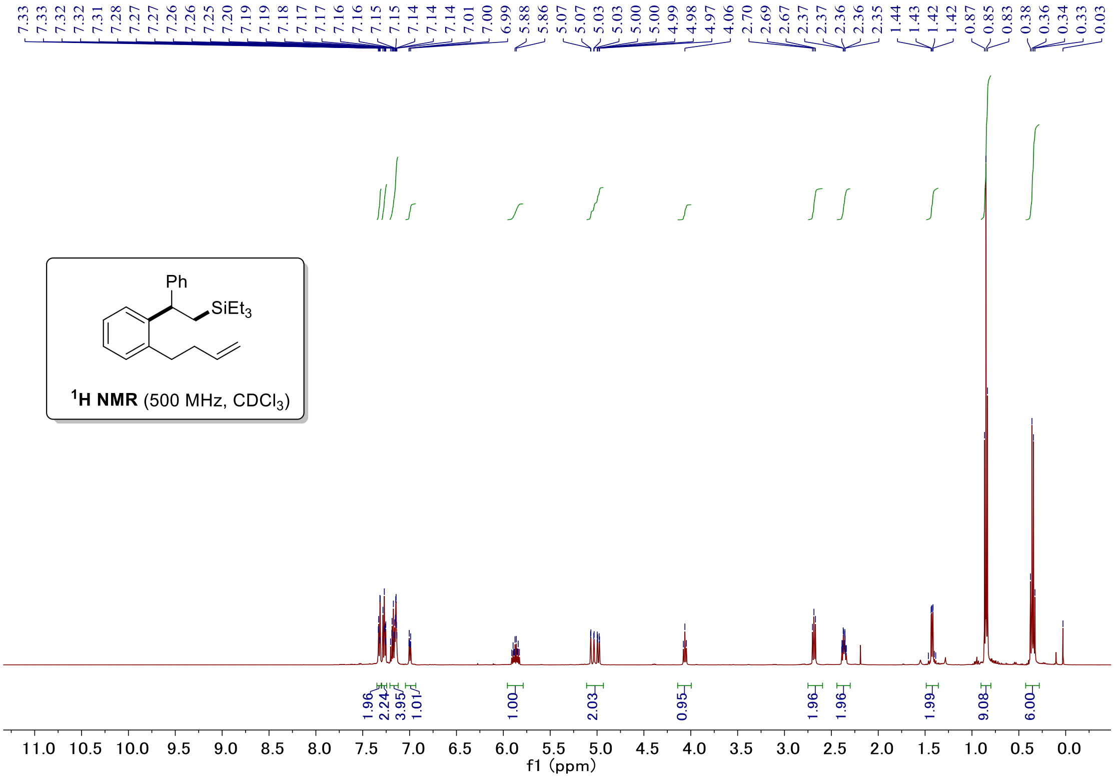

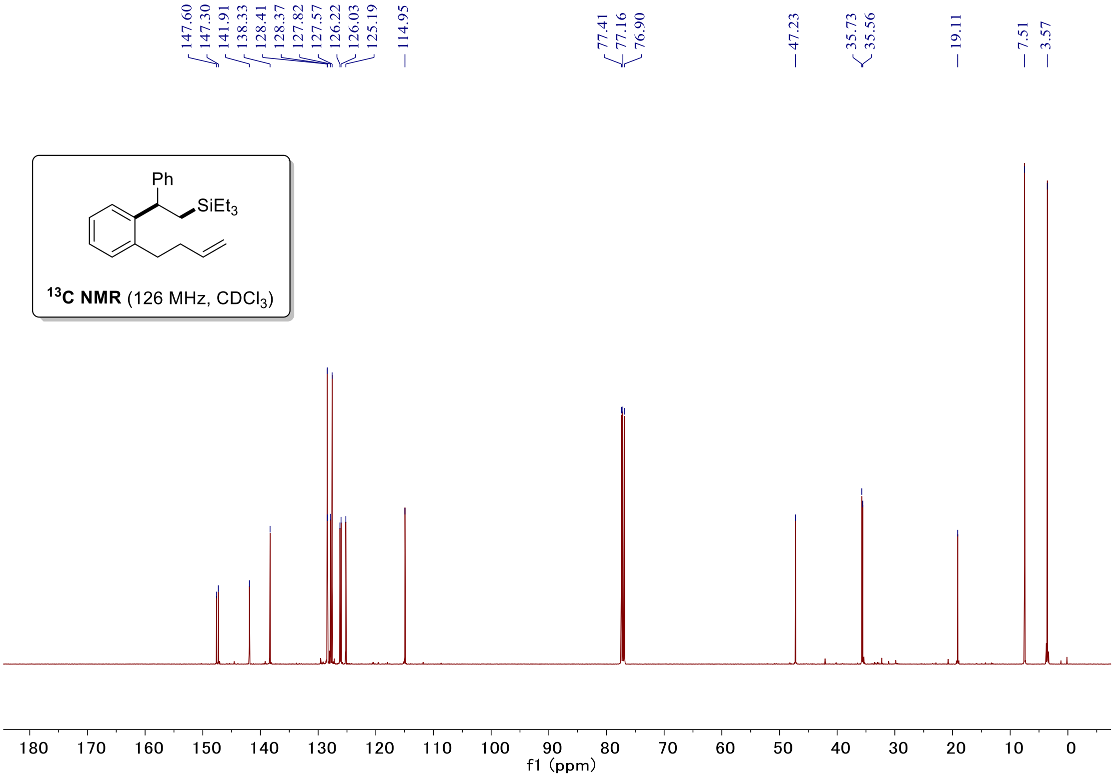

Triethyl(2-phenyloct-7-en-1-yl)silane (5ka)

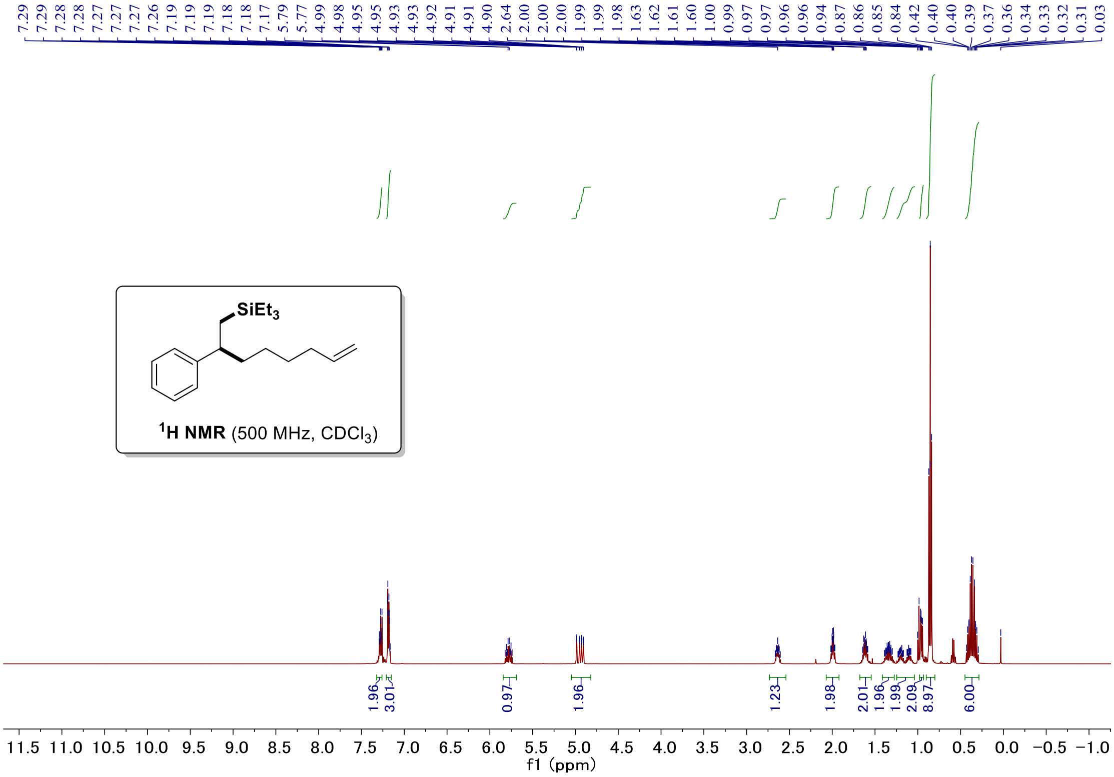

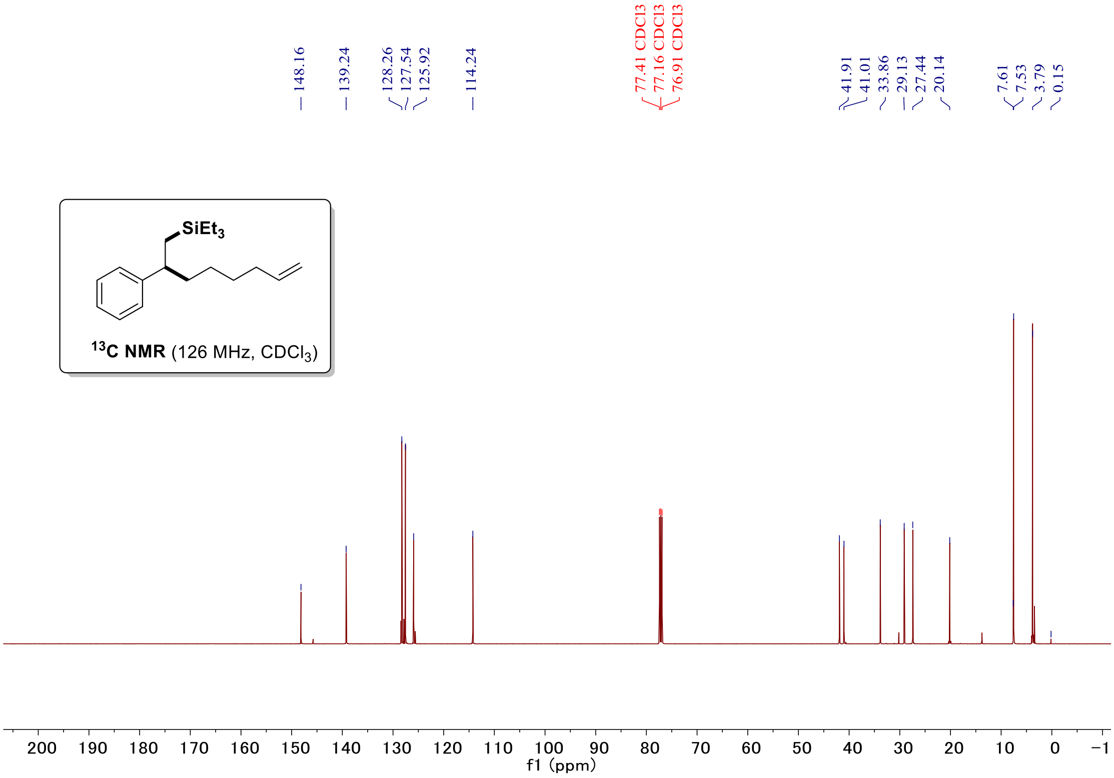

Triethyl(2-phenyloct-7-en-1-yl)silane (5li)

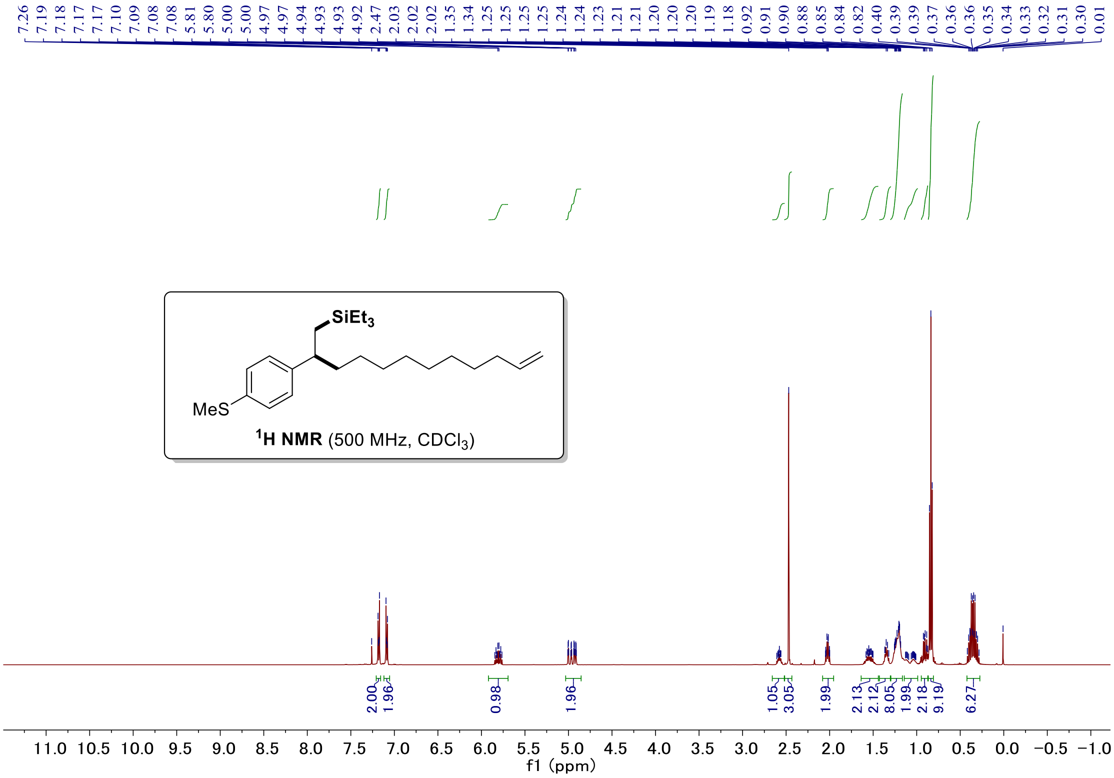

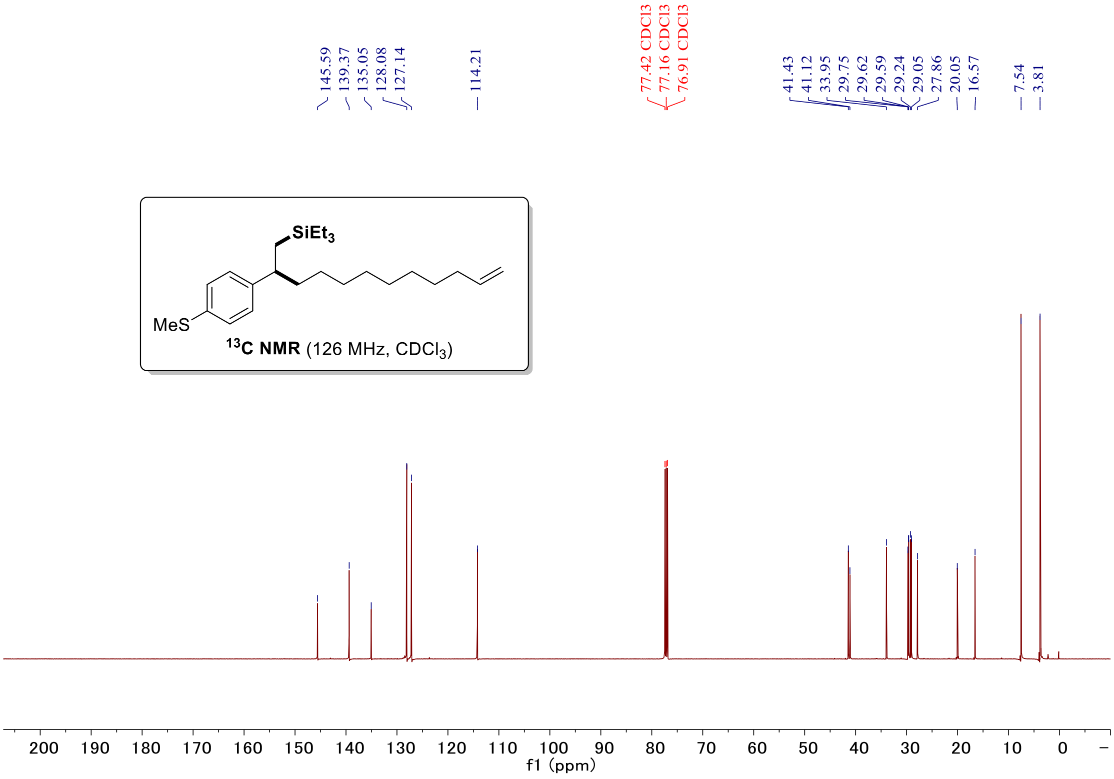

Triethyl((1-phenyl-2,3-dihydro-1*H*-inden-1-yl)methyl)silane (8b)

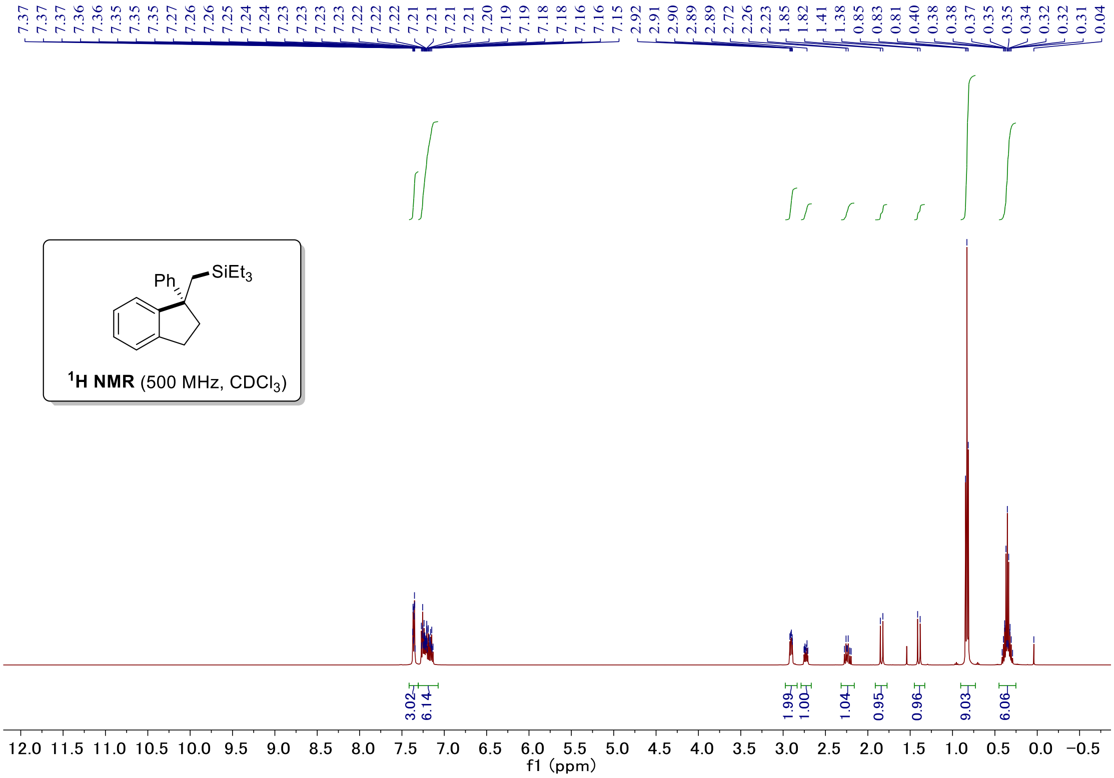

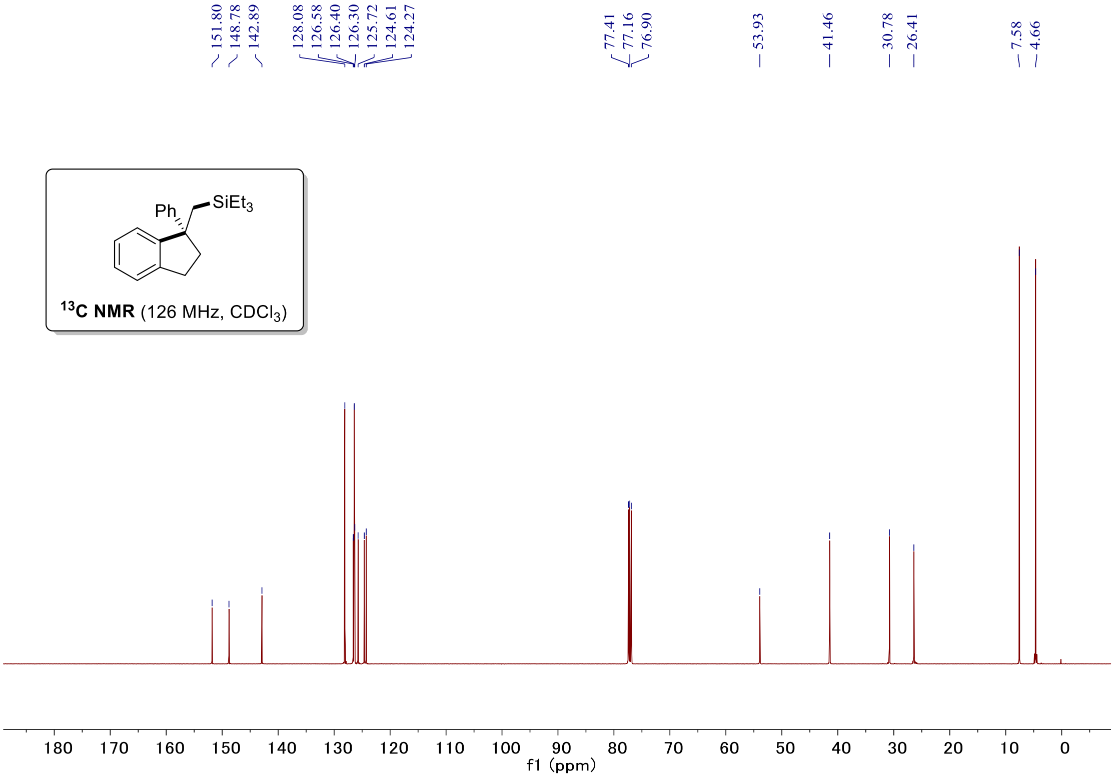

((1,5-Diphenyl-2,3-dihydro-1*H*-inden-1-yl)methyl)triethylsilane (8c)

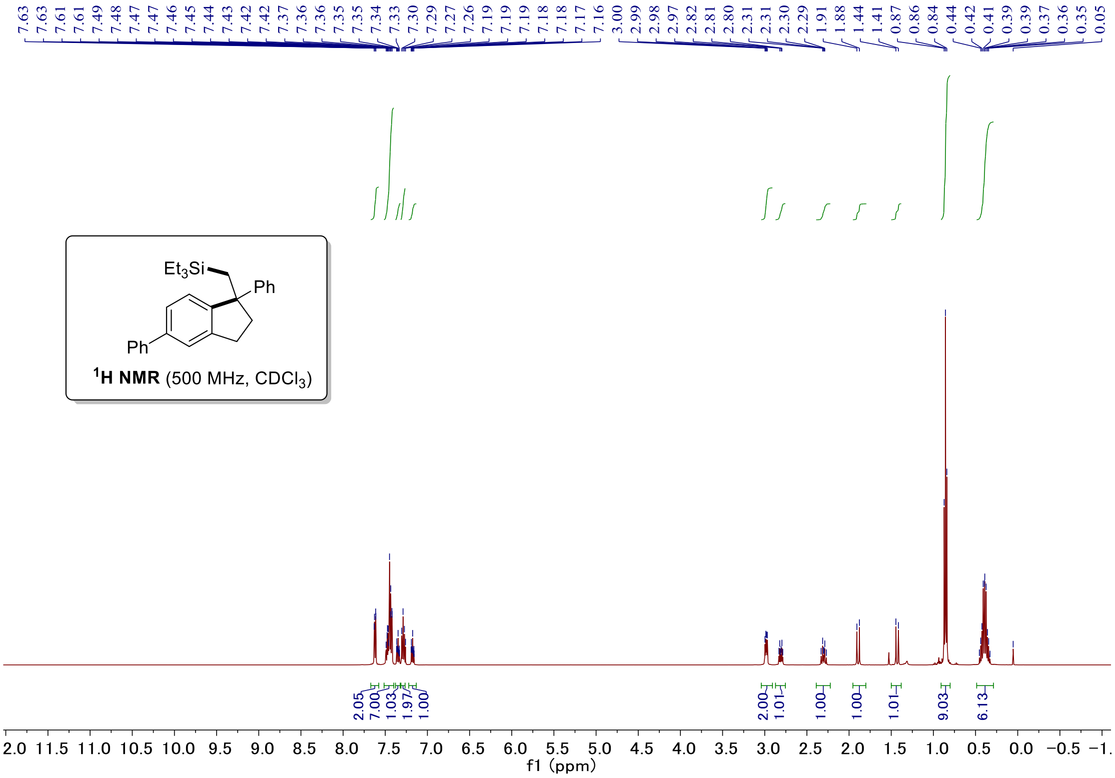

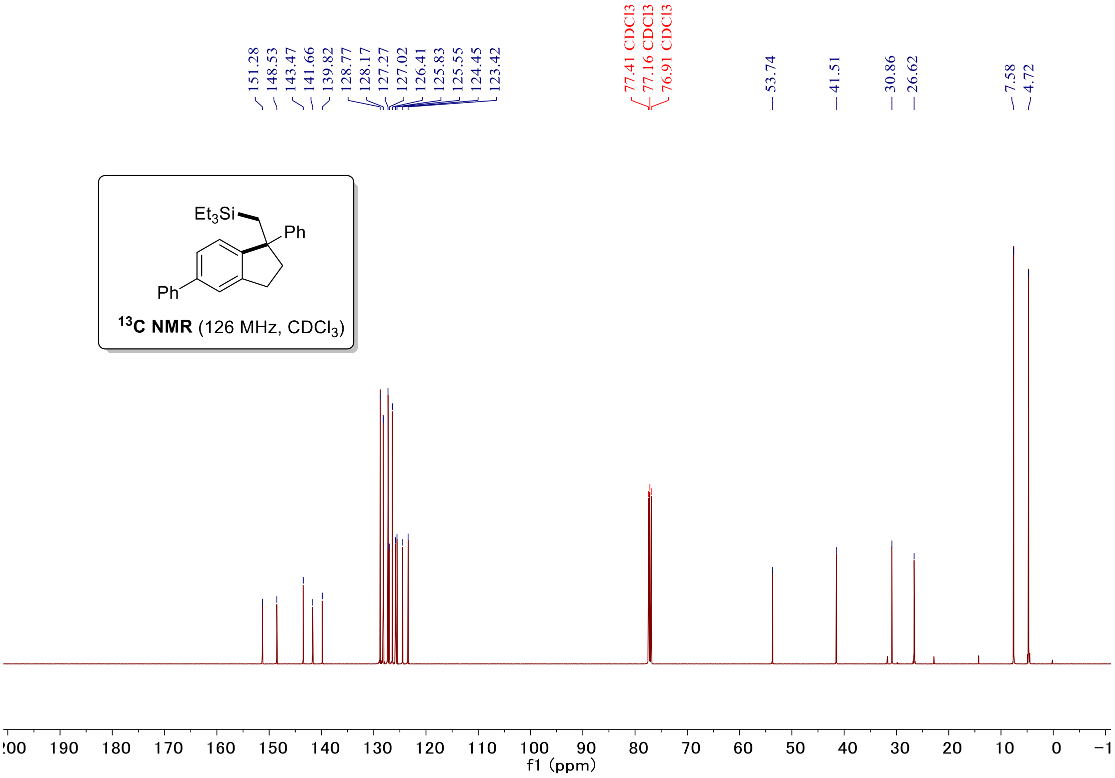

5-(4-(2-Fluoro-5-methoxyphenyl)but-1-en-2-yl)benzo[*d*][1,3]dioxole (8d)

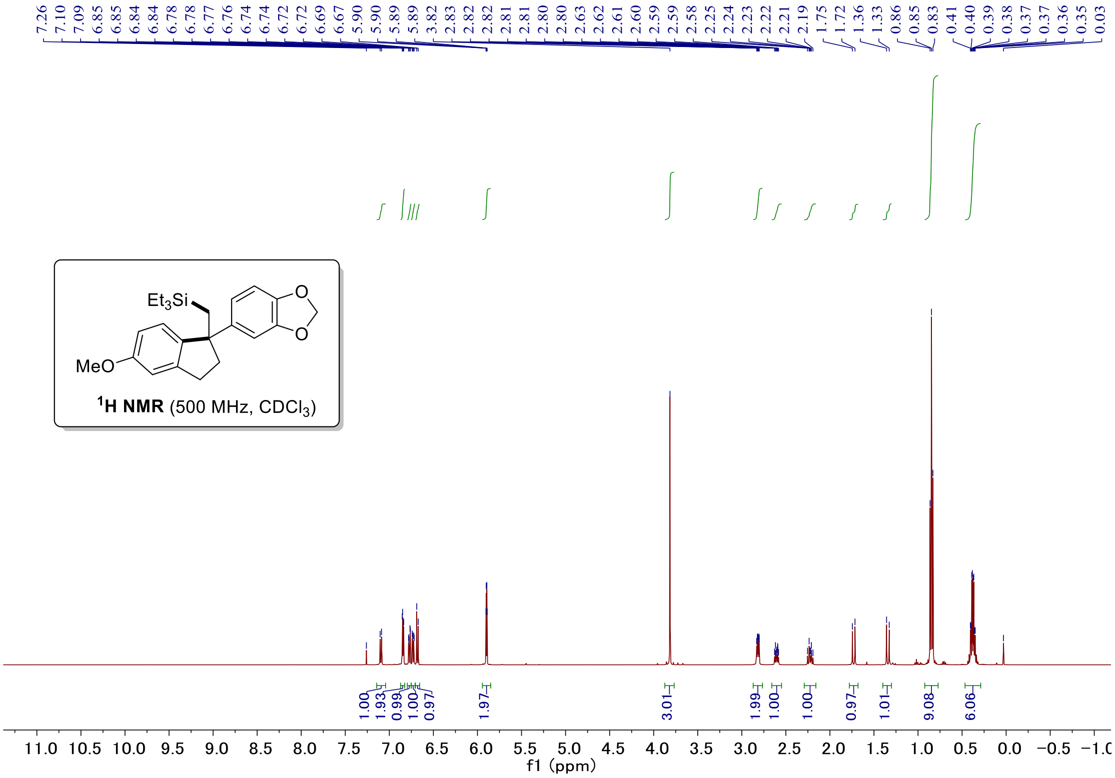

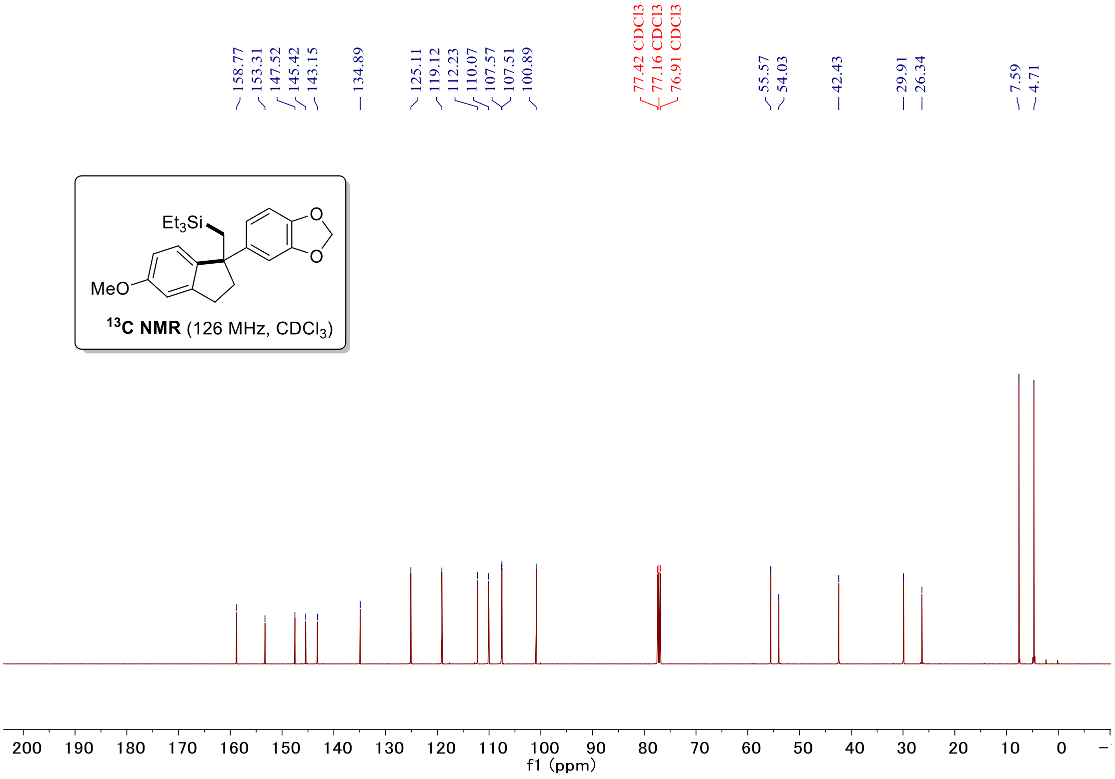

2,11-Di-*p*-tolyldodecane-1,12-diyl)bis(triethylsilane) (5mc)

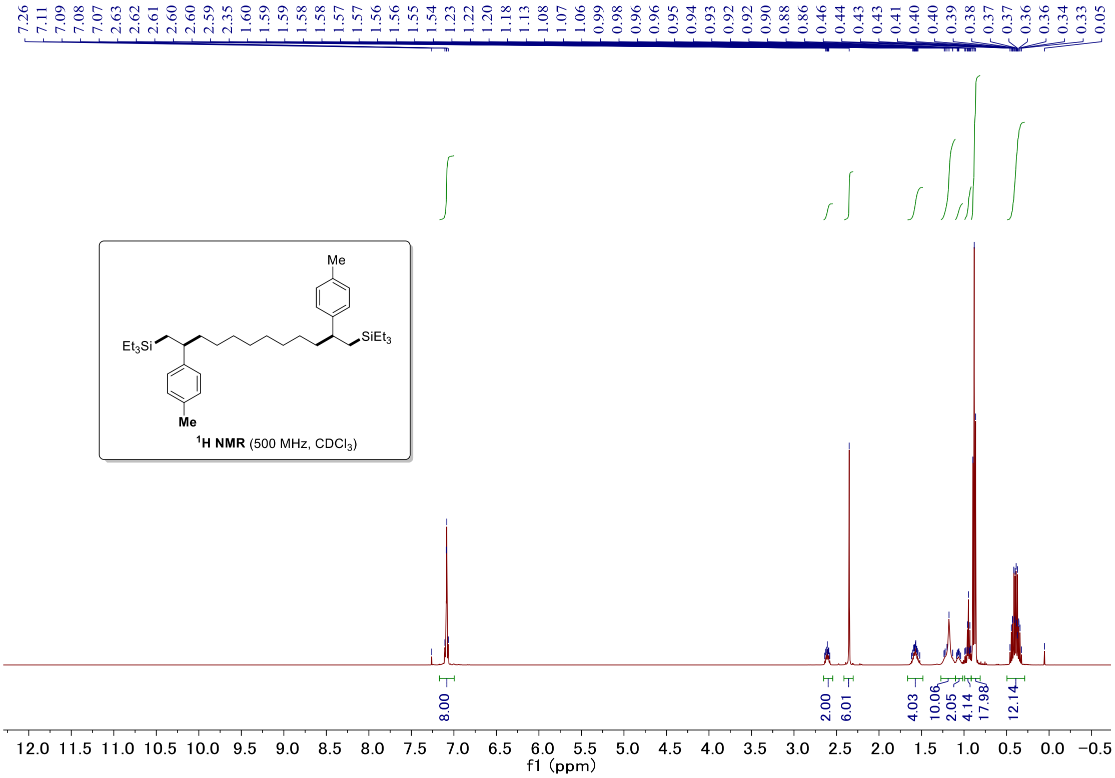

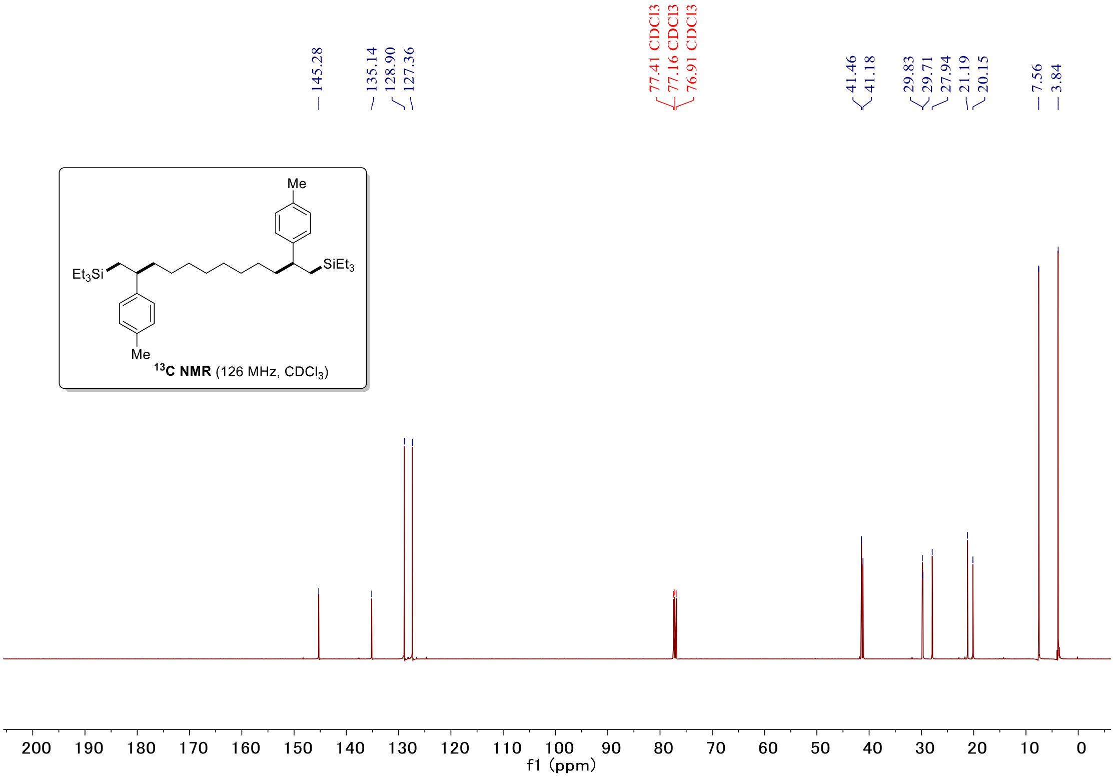

Biphenyl-4-yltriethylsilane (11)

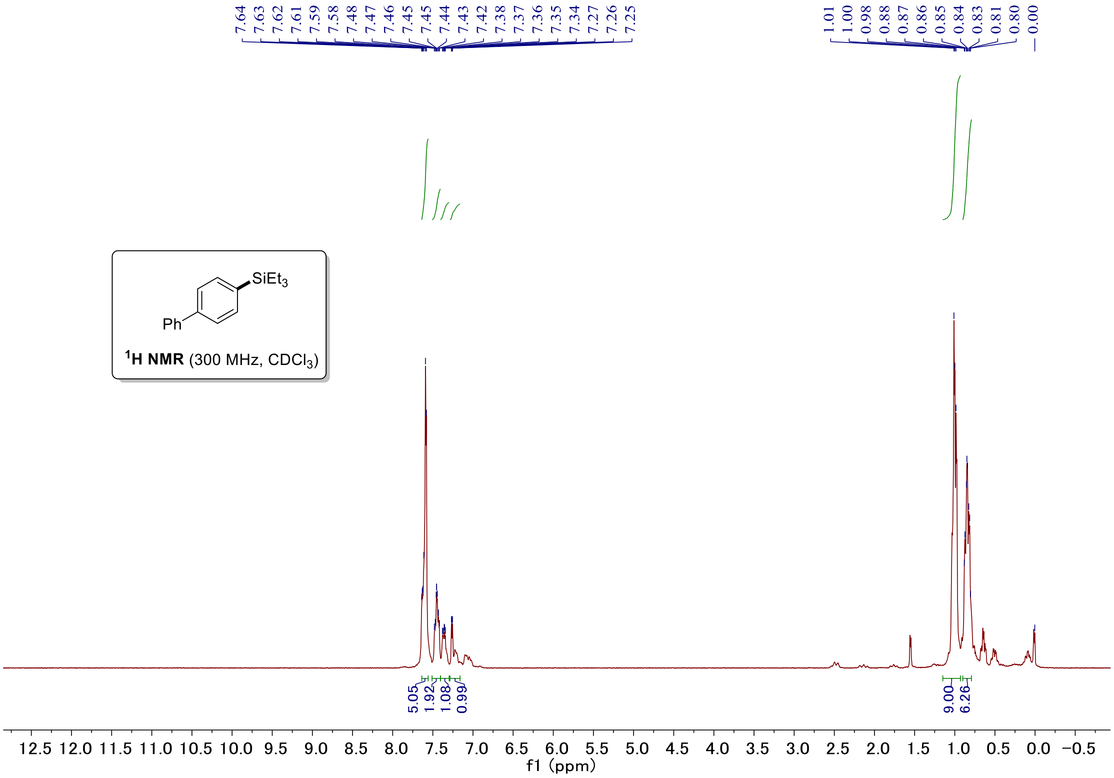

2-(Biphenyl-4-yl)-4,4,5,5-tetramethyl-1,3,2-dioxaborolane (12)

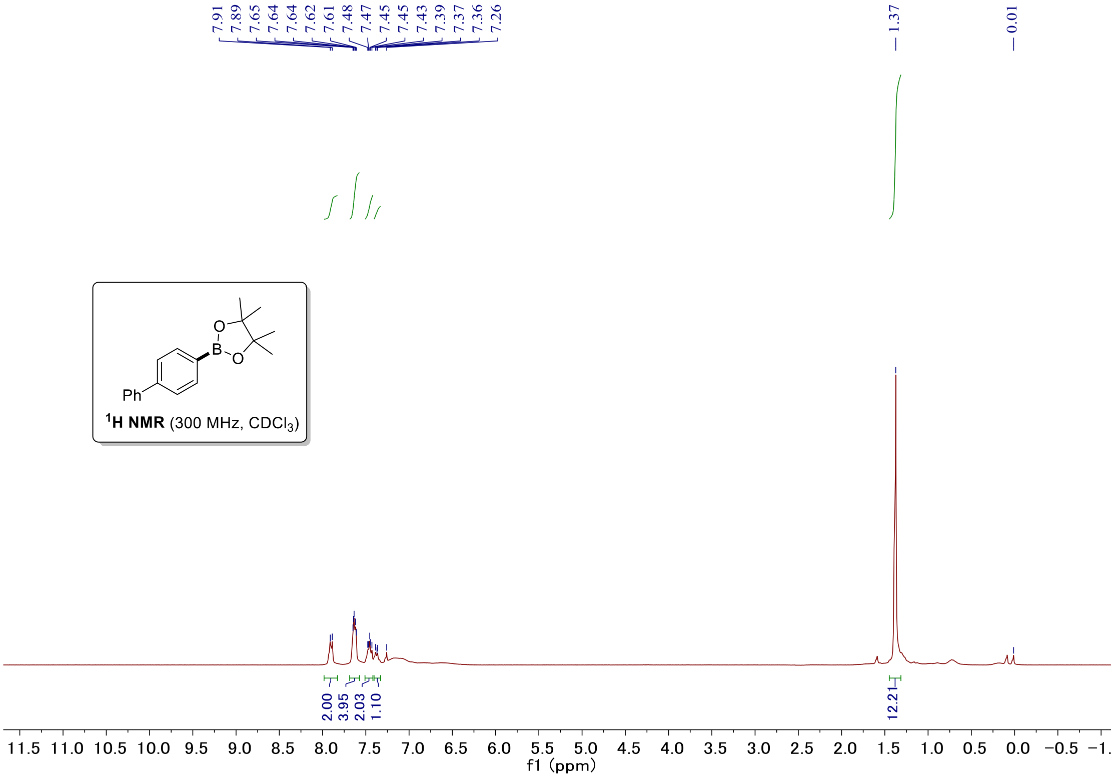

Triethyl(phenethyl)silane (14)

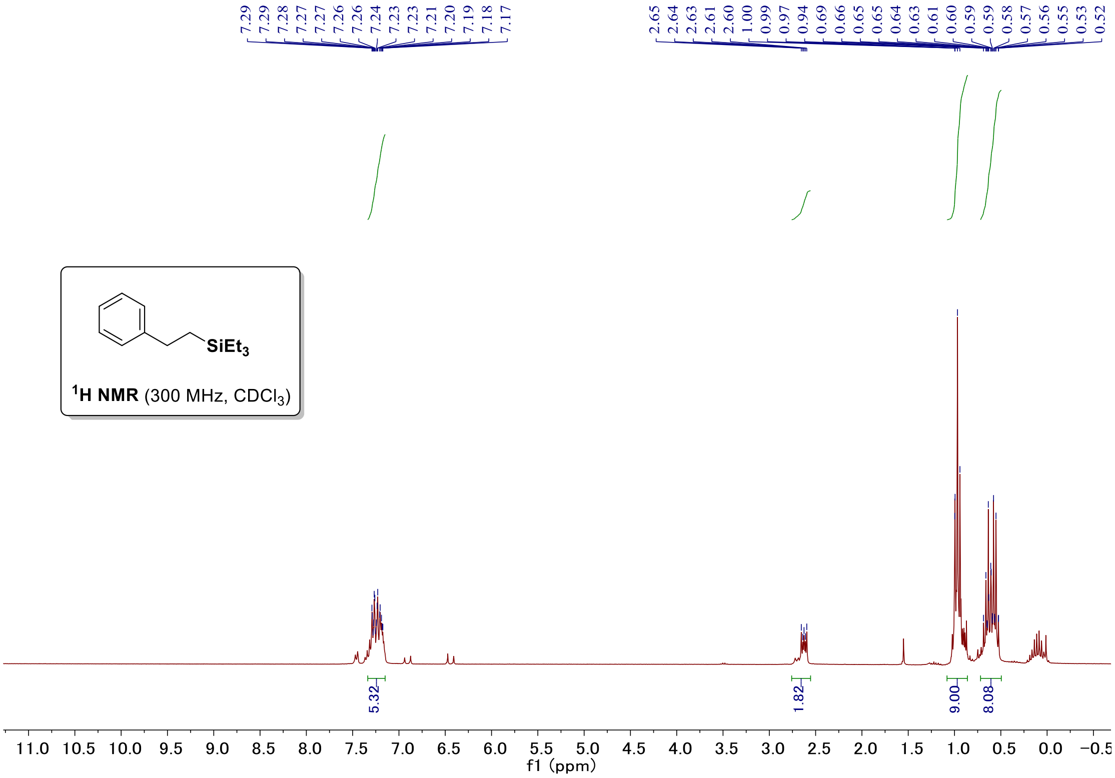

(*Z*)-Triethyl(2-phenylpent-2-en-1-yl)silane (15)

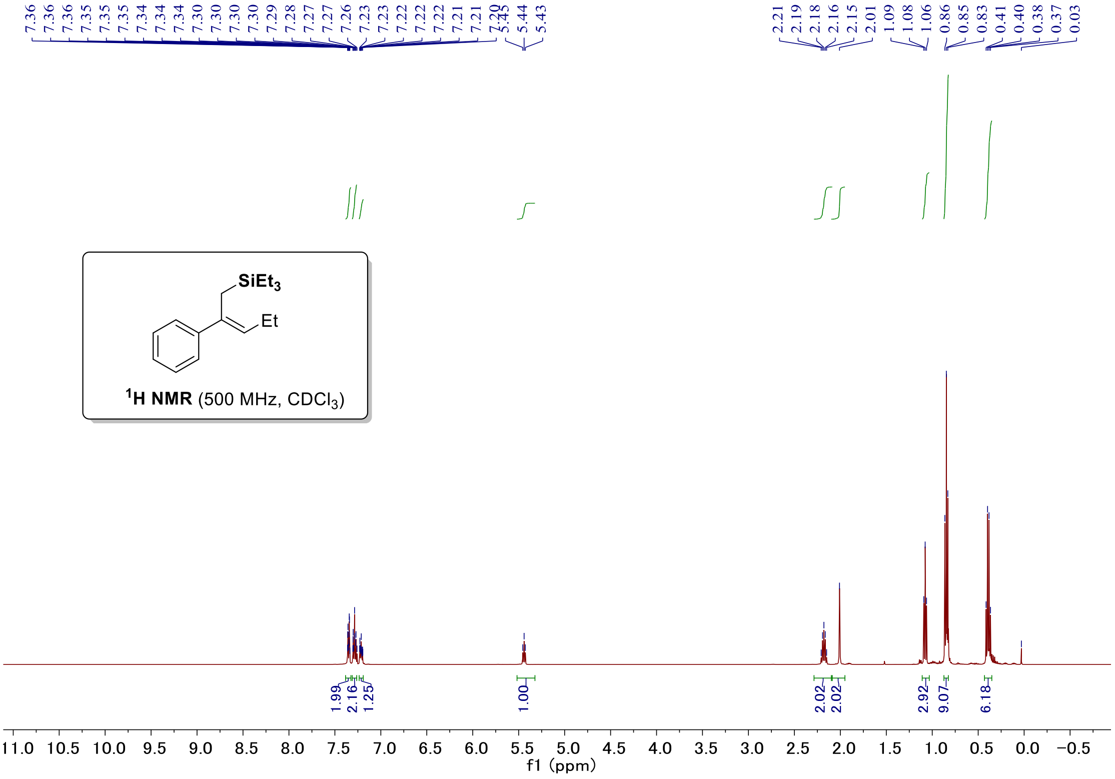

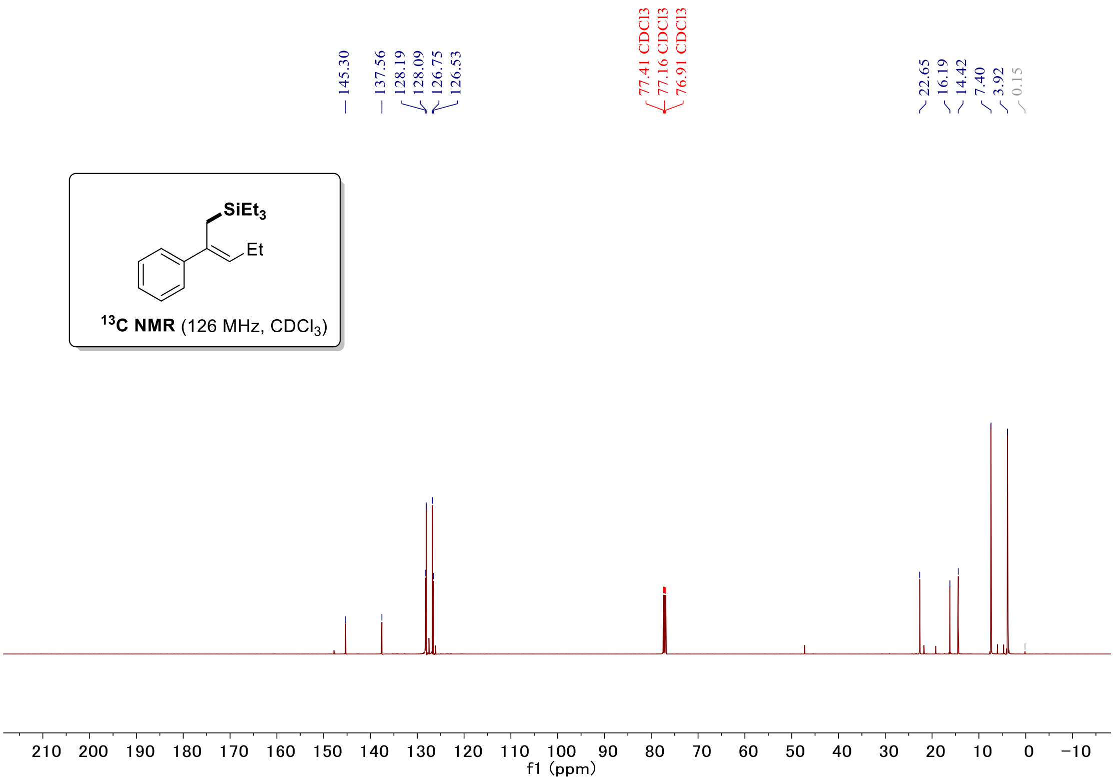

Triethyl((1-phenylcyclopropyl)methyl)silane (16)

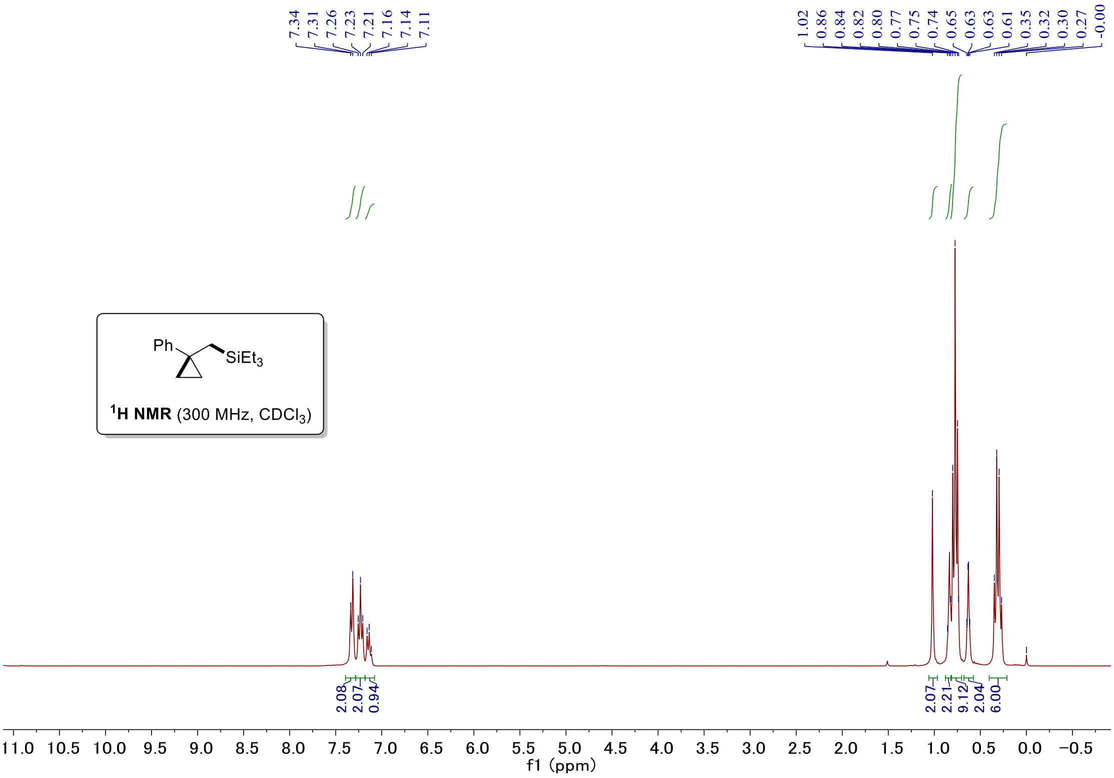

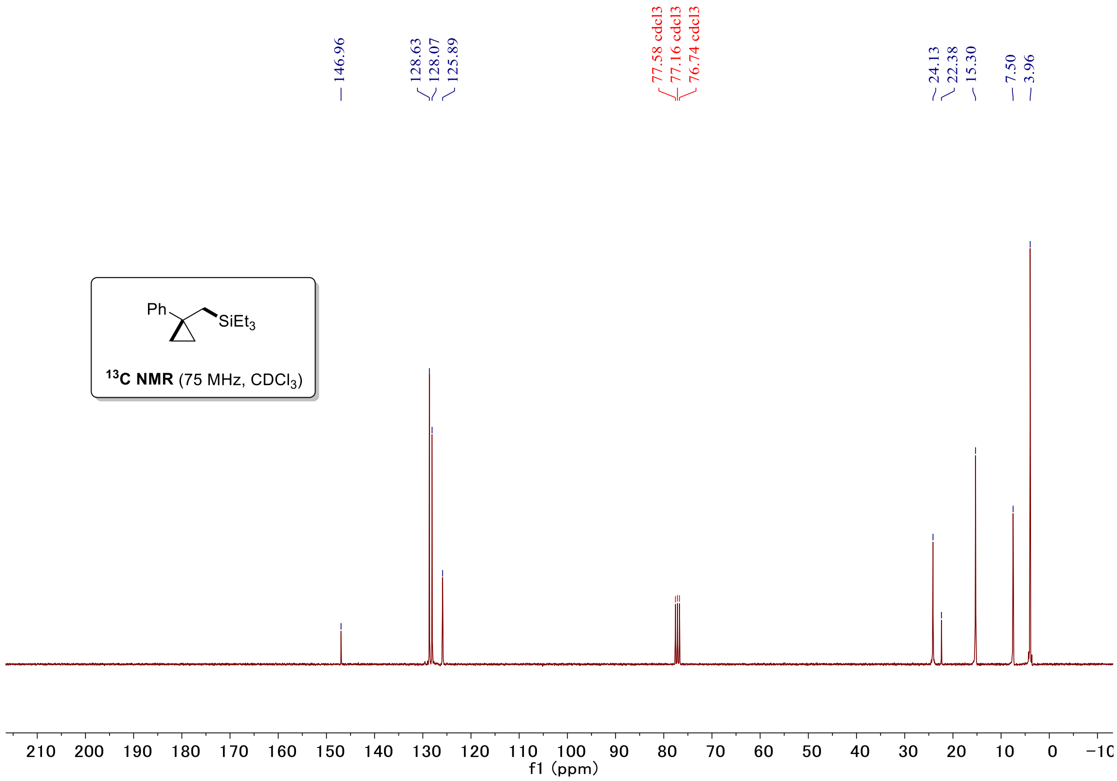

Triethyl(hex-5-en-1-yl)silane (17)

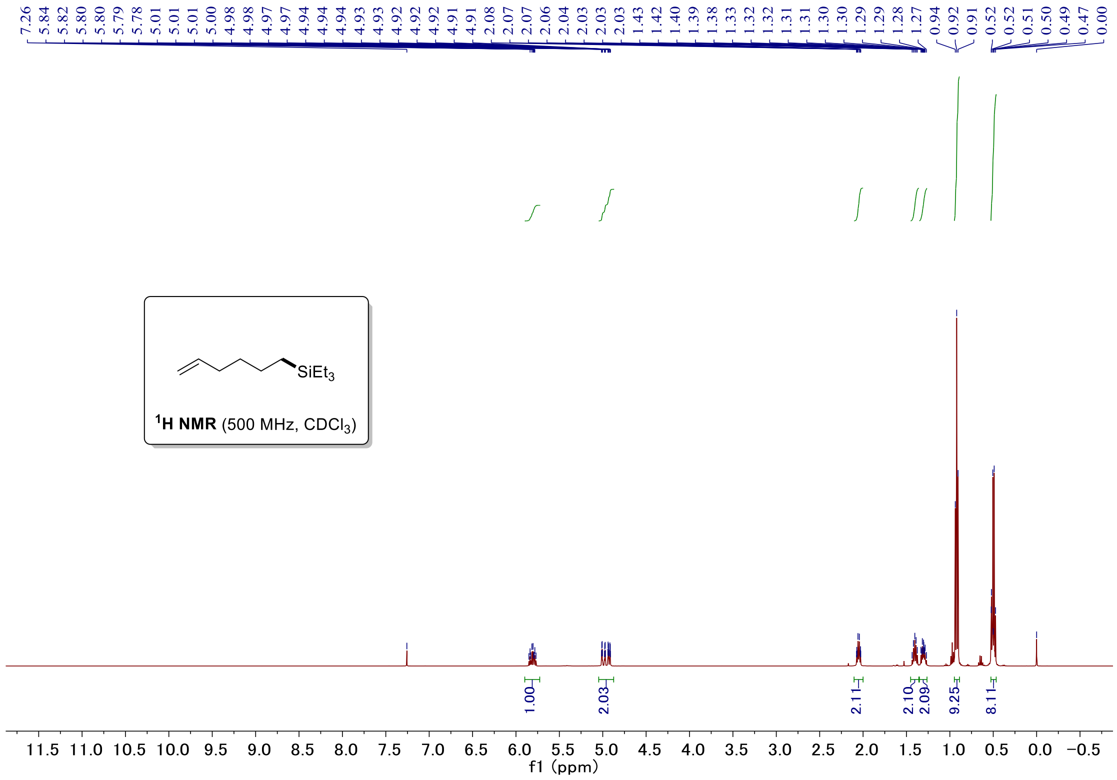

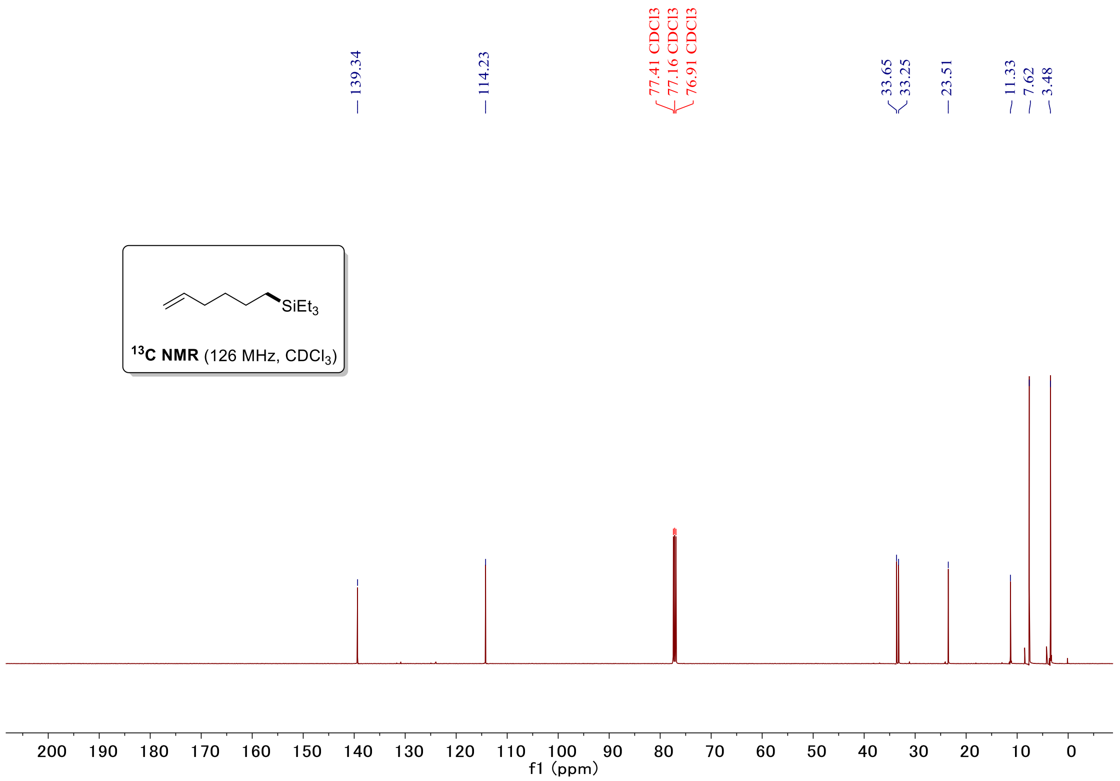

(4-(Biphenyl-4-yl)-2,4-diphenylbutyl)triethylsilane (18)

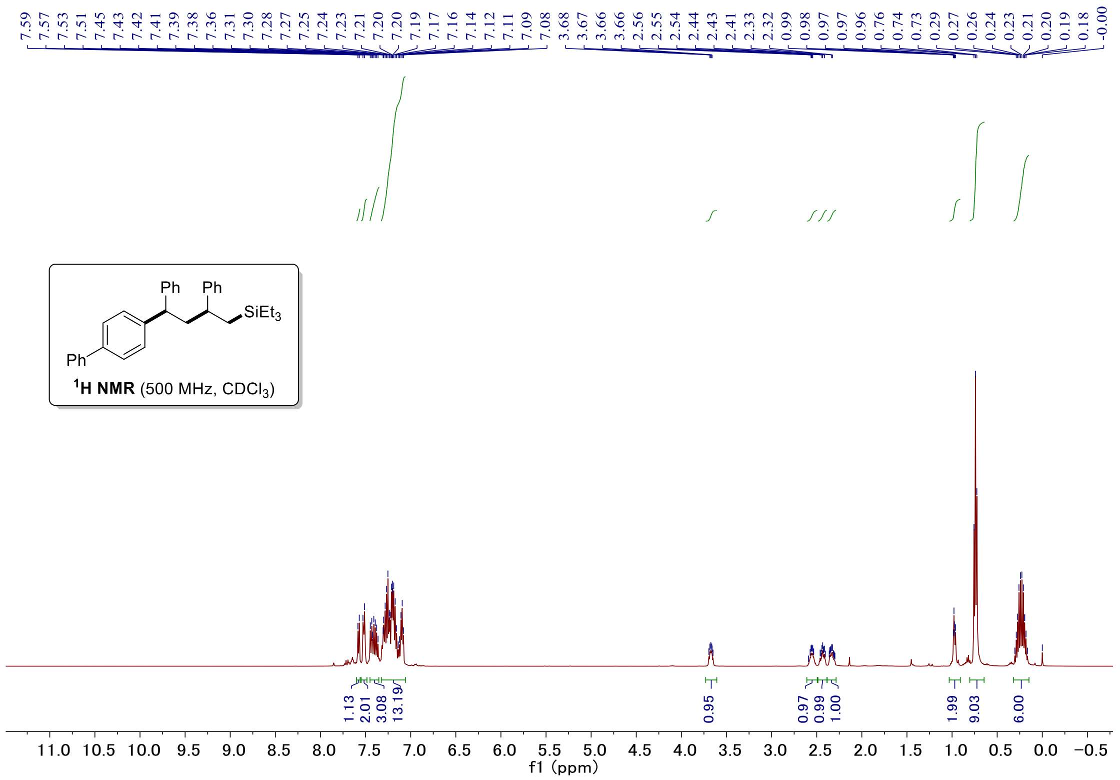

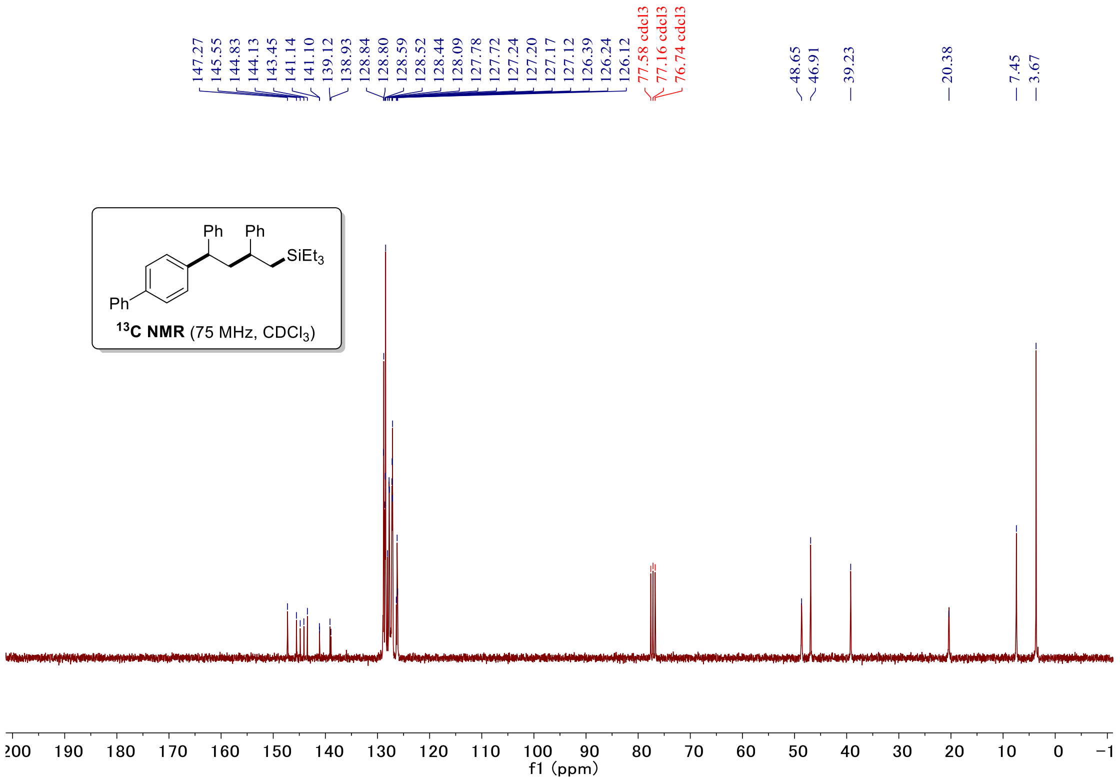

Hexaethyldisilane

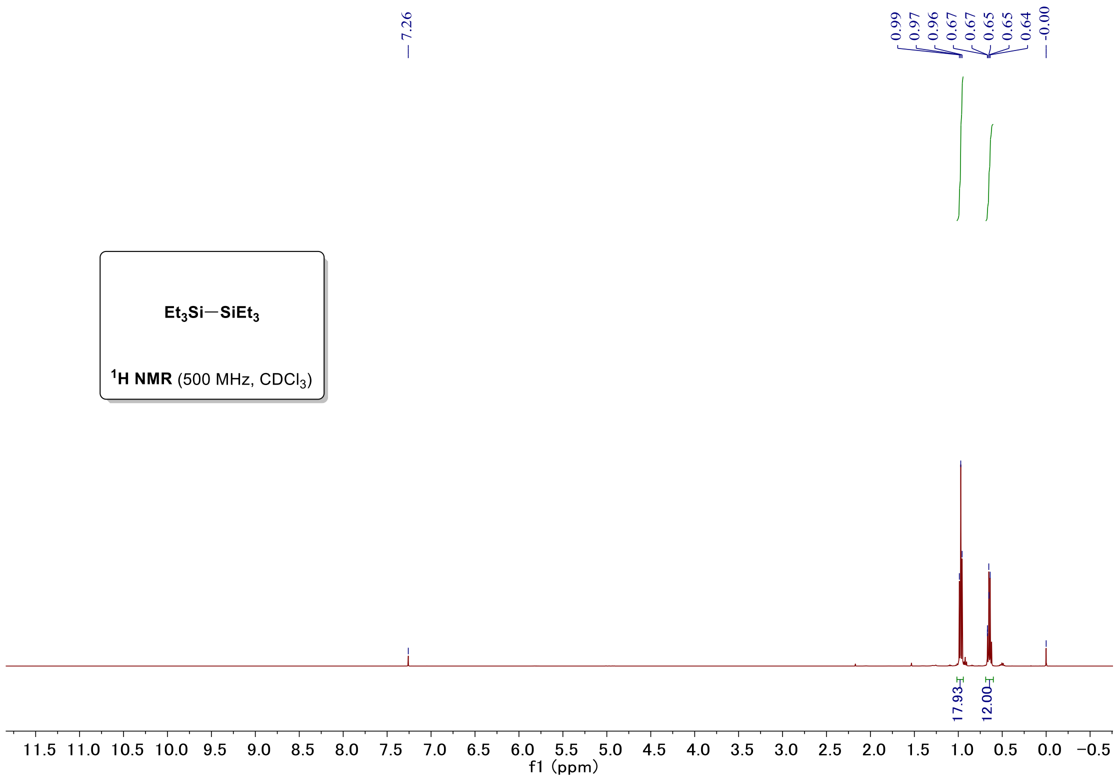

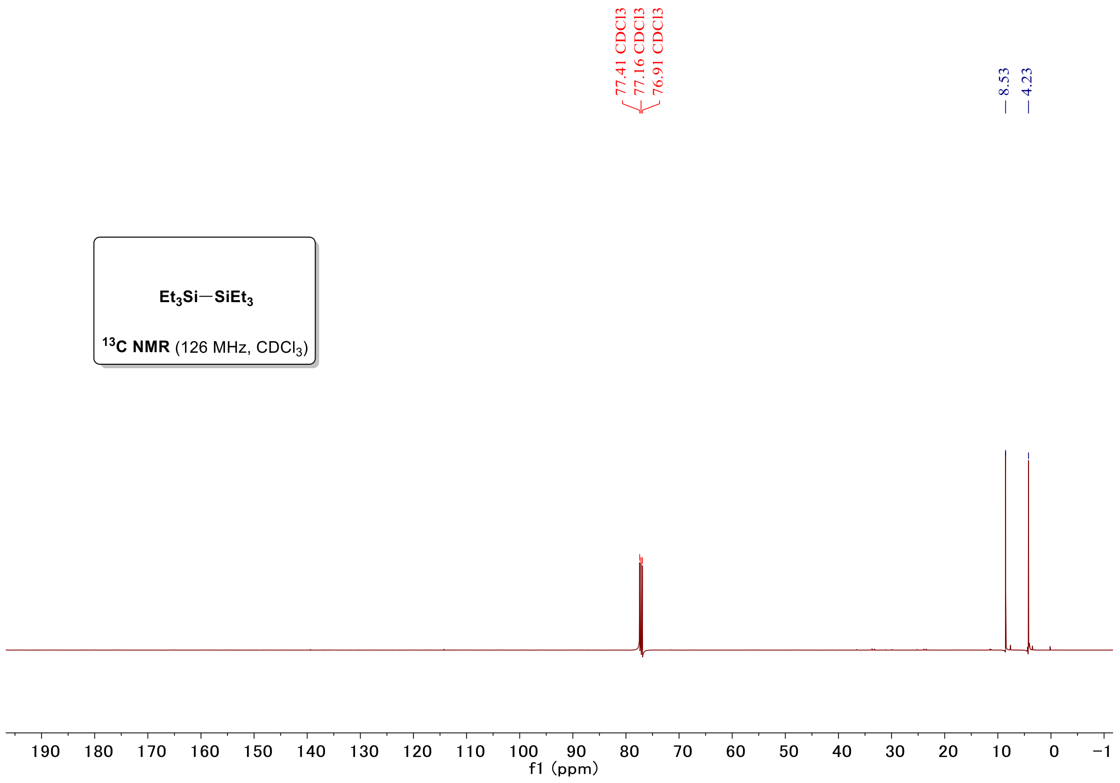
77.41 CDCl3
77.16 CDCl3
76.91 CDCl3
8.53
4.23
Et3Si—SiEt3
13C NMR (126 MHz, CDCl3)
190 180 170 160 150 140 130 120 110 100 90 80 70 60 50 40 30 20 10 0 −1
f1 (ppm)

10. Supplementary References

1. Niwa, T. et al. Ni/Cu-catalyzed defluoroborylation of fluoroarenes for diverse C–F bond functionalizations. *J. Am. Chem. Soc.* **137,** 14313–14318 (2015).
2. Baxendale, I. R. et al Microwave-assisted Suzuki coupling reactions with an encapsulated palladium catalyst for batch and continuous-flow transformations. *Chem. Eur. J.* **12,** 4407–4416 (2006).
3. Manzoor, A. et al. Catalysis of cross-coupling and homocoupling reactions of aryl halides utilizing Ni(0), Ni(I), and Ni(II) precursors; Ni(0) compounds as the probable catalytic species but Ni(I) compounds as intermediates and products. *Organometallics* **36,** 3508–3519 (2017).
4. Antelo Miguez, J. M. et al. A practical and general synthesis of unsymmetrical terphenyls. *J. Org. Chem.* **72,** 7771–7774 (2007).
5. Czompa, A. et al. Scope and limitation of propylene carbonate as a sustainable solvent in the Suzuki–Miyaura reaction. *RSC Advances*, **9,** 37818–37824 (2019).
6. Chen, Y., Liu, C. & Wang, L. Effects of fluorine substituent on properties of cyclometalated iridium(III) complexes with a 2,2′-bipyridine ancillary ligand. *Tetrahedron* **75,** 130686 (2019).
7. Sander, K. et al. Sulfonium salts as leaving groups for aromatic labelling of drug-like small molecules with fluorine-18. *Sci. Rep.* **5,** 9941 (2015).
8. Rocaboy, R., Anastasiou, I. & Baudoin, O. Redox-neutral coupling between two C(sp^3)−H bonds enabled by 1,4-palladium shift for the synthesis of fused heterocycles. *Angew. Chem. Int. Ed.* **58,** 14625–14628 (2019).
9. Yang, L. Q. et al. Electrochemically enabled C3-formylation and -acylation of indoles with aldehydes, *Org. Lett.* **21,** 7702–7707 (2019).
10. Cui, B. Q., Jia, S. C., Tokunaga, E. & Shibata, N. Defluorosilylation of fluoroarenes and fluoroalkanes. *Nature Commun.* **9,** 4393 (2018).
11. Punna, N., Harada, K., Zhou, J. & Shibata, N. Pd-catalyzed decarboxylative cyclization of trifluoromethyl vinyl benzoxazinanones with sulfur ylides: access to trifluoromethyl dihydroquinolines. *Org. Lett.* **21,** 1515–1520 (2019).
12. Russo, A. T. et al. A Simple borohydride-based method for selective 1,4-conjugate reduction of α, β-unsaturated carbonyl compounds. *Tetrahedron Lett.* **52,** 6823–6826 (2011).
13. Yang, F. Y., Jin, Y. X. & Wang, C. Nickel-catalyzed asymmetric intramolecular reductive Heck reaction of unactivated alkenes. *Org. Lett.* **21,** 6989–6994 (2019).
14. Niwa, T., Ochiai, H. & Hosoya, T. Copper-catalyzed *ipso*-borylation of fluoroarenes. *ACS Catal.* **7,** 4535–4541 (2017).
15. Wang, J. D., Ogawa, Y. & Shibata, N. Activation of saturated fluorocarbons to synthesize spirobiindanes, monofluoroalkenes, and indane derivatives. *iScience* **17,** 132–143 (2019).
16. Bosch, P., Camps, F., Chamorro, E., Gasol, V., Guerrero, A. Tetrabutylammonium biflouride: a versatile and efficient flourinating agent. *Tetrahedron Lett.* **28,** 4733–4736 (1987).
17. Beaulieu, F. Aminodifluorosulfinium tetrafluoroborate salts as stable and crystalline deoxofluorinating reagents. *Org. Lett.* **11,** 5050–5053 (2009).
18. Koroniak, H. *et al.* 1,1,3,3,3-Pentafluoropropene secondary amine adducts new selective fluorinating agents. *J. Fluorine Chem.* **127,** 1245–1251 (2006).

19. Melero, C. et al. New modes of reactivity in the threshold of the reduction potential in solution. alkylation of lithium PAH (polycyclic aromatic hydrocarbon) dianions by primary fluoroalkanes: a reaction pathway complementing the classical birch reductive alkylation. *Chem. Eur. J.* **13,** 10096–10107 (2007).
20. Ma, X. & Herzon, S. B. Intermolecular hydropyridylation of unactivated aAlkenes. *J. Am. Chem. Soc.* **138,** 8718–8721 (2016).
21. Wu, L. L. & Yu, Steve S. F. Tuning the regio- and stereoselectivity of C–H activation in *n*-octanes by cytochrome P450 BM-3 with fluorine substituents: evidence for interactions between a C–F bond and aromatic π systems. *Chem. Eur. J.* **17,** 4774–4787 (2011).
22. Nicolaou, K. C. Design, synthesis, and biological evaluation of platensimycin analogues with varying degrees of molecular complexity. *J. Am. Chem. Soc.* **130,** 13110–13119 (2008).
23. Symmers, P. R. Non-equilibrium cobalt(III) "click" capsules. *Chem. Sci.* **6,** 756–760 (2015).
24. Cao, H. Y. et al. Copper-catalyzed selective semihydrogenation of terminal alkynes with hypophosphorous acid. *Adv. Synth. Catal.* **356,** 765–769 (2014).
25. Shirakawa, E. Cross-coupling reaction of organostannanes with aryl halides catalyzed by nickel-triphenylphosphine or nickel-lithium halide complex. *Synthesis* **10,** 1544–1549 (1998)
26. Barbasiewicz, M., Makosza, M. Intermolecular reactions of chlorohydrine anions: acetalization of carbonyl compounds under basic conditions. *Org. Lett.* **8,** 3745–3748 (2006).
27. Aslam, S. N. et al. Synthesis of cicerfuran, an antifungal benzofuran, and some related analogues. *Tetrahedron* **62,** 4214– 4226 (2006).
28. Seferos, D. S. et al. α,ω-Bis(thioacetyl)oligophenylenevinylene Chromophores from Thioanisol Precursors. *J. Org. Chem.* **69,** 1110–1119 (2004).
29. Yasukawa, N.et al. Highly-functionalized arene synthesis based on palladium on carbon-catalyzed aqueous dehydrogenation of cyclohexadienes and cyclohexenes. *Green Chem.* **20,** 1213–1217 (2018).
30. Liu, J. *et al*. Organocatalytic 1,5-trifluoromethylthio-sulfonylation of vinylcyclopropane mediated by visible light in the water phase. *Org. Chem. Front.* **7,** 1314–1320 (2020).
31. Köpfer, A. et al. Regiodivergent reductive coupling of 2-substituted dienes to formaldehyde employing ruthenium or nickel catalyst: hydrohydroxymethylation *via* transfer hydrogenation. *Chem. Sci.* **4,** 1876–1880 (2013).
32. Shu, C. et al. Synthesis of functionalized cyclopropanes from carboxylic acids by a radical addition–polar cyclization cascade. *Angew. Chem. Int. Ed.* **57,** 15430–15434 (2018).
33. Thompson, H. W. & Rashid, S. Y. Stereochemical control of reductions. 9. haptophilicity studies with 1,1-disubstituted 2-methyleneacenaphthenes. *J. Org. Chem.* **67,** 2813–2825 (2002).
34. Furuya, T., Strom, A. E. & Ritter, T. Silver-mediated fluorination of functionalized aryl stannanes. *J. Am. Chem. Soc.* **131,** 1662–1663 (2009).
35. Tian, M. Q. et al. Divergent C–H oxidative radical functionalization of olefins to install tertiary alkyl motifs enabled by copper catalysis. *Org. Lett.* **21,** 1607–1611 (2019).
36. Boebel, T. A. & Hartwig, J. F. Iridium-catalyzed preparation of silylboranes by silane borylation and their use in the catalytic borylation of arenes. *Organometallics* **27,** 6013–6019 (2008).
37. Shishido, R. et al. General synthesis of trialkyl- and dialkylarylsilylboranes: versatile silicon nucleophiles in organic synthesis. *J. Am. Chem. Soc.* **142,** 14125–14133 (2020).
38. Ito, H., Horita, Y. & Yamamoto, E. Potassium *tert*-butoxide-mediated regioselective silaboration of aromatic alkenes. *Chem.Commun.* **48,** 8006–8008 (2012).

39. Gao, P. et al. Transition-metal-free defluorosilylation of fluoroalkenes with silylboronates. *Chin. J. Chem.* **37,** 1009–1014 (2019).
40. Kleeberg, C. & Borner, C. On the reactivity of silylboranes toward Lewis bases: heterolytic B-Si cleavage vs. adduct formation. *Eur. J. Inorg. Chem.* **2013,** 2799–2806 (2013).
41. Wrackmeyer, B. Applications of ^{29}Si NMR parameters. *Annual Reports on NMR Spectroscopy* **57,** 1−49 (2006).
42. Simonneau, A. & Oestreich, M. 3-Silylated cyclohexa-1,4-dienes as precursors for gaseous hydrosilanes: the $B(C_6F_5)_3$-catalyzed transfer hydrosilylation of alkenes. *Angew. Chem. Int. Ed.* **52,** 11905–11907 (2013).
43. Pu, X., Hu, J., Zhao, Y. & Shi, Z. Nickel-catalyzed decarbonylative borylation and silylation of esters. *ACS Catal.* **6,** 6692–6698 (2016).
44. Fyfe, J. W. B., Seath, C. P. & Watson, A. J. B. Chemoselective boronic ester synthesis by controlled speciation. *Angew. Chem. Int. Ed.* **53,** 12077–12080 (2014).
45. Yang, X. & Wang, C. Diverse fates of β-silyl radical under manganese catalysis: hydrosilylation and dehydrogenative silylation of alkenes. *Chin. J. Chem.* **36,** 1047–1051 (2018).
46. Fukumoto, K. et al. Desulfurization of *N*, *N*-dimethylthioformamide by hydrosilane with the help of an iron complex. Isolation and characterization of an iron–carbene complex as an intermediate of C–S double bond cleavage. *Chem. Commun.* **48,** 3809 (2012).
